# Genome Wide Association Study Pinpoints Key Agronomic QTLs in African Rice *Oryza glaberrima*

**DOI:** 10.1186/s12284-020-00424-1

**Published:** 2020-09-16

**Authors:** Philippe Cubry, Hélène Pidon, Kim Nhung Ta, Christine Tranchant-Dubreuil, Anne-Céline Thuillet, Maria Holzinger, Hélène Adam, Honoré Kam, Harold Chrestin, Alain Ghesquière, Olivier François, François Sabot, Yves Vigouroux, Laurence Albar, Stefan Jouannic

**Affiliations:** 1 DIADE, Univ Montpellier, IRD, Montpellier, France; 2 Present address: Leibniz Institute of Plant Genetics and Crop Plant Research (IPK) Gatersleben, Seeland, Germany; 3 LMI RICE, AGI, IRD, Univ Montpellier, CIRAD, USTH, Hanoi, Vietnam; 4 Present address: National Institute of Genetics, Mishima, Shizuoka Japan; 5 INERA, Bobo Dioulasso, Burkina Faso; 6 Université Grenoble-Alpes, Centre National de la Recherche Scientifique, Grenoble, France

**Keywords:** African rice, Genome wide association study, Flowering time, Panicle architecture, RYMV, Climate variation

## Abstract

**Background:**

African rice, ***Oryza glaberrima***, is an invaluable resource for rice cultivation and for the improvement of biotic and abiotic resistance properties. Since its domestication in the inner Niger delta ca. 2500 years BP, African rice has colonized a variety of ecologically and climatically diverse regions. However, little is known about the genetic basis of quantitative traits and adaptive variation of agricultural interest for this species.

**Results:**

Using a reference set of 163 fully re-sequenced accessions, we report the results of a Genome Wide Association Study carried out for African rice. We investigated a diverse panel of traits, including flowering date, panicle architecture and resistance to *Rice yellow mottle virus*. For this, we devised a pipeline using complementary statistical association methods. First, using flowering time as a target trait, we found several association peaks, one of which co-localised with a well described gene in the Asian rice flowering pathway, *OsGi*, and identified new genomic regions that would deserve more study. Then we applied our pipeline to panicle- and resistance-related traits, highlighting some interesting genomic regions and candidate genes. Lastly, using a high-resolution climate database, we performed an association analysis based on climatic variables, searching for genomic regions that might be involved in adaptation to climatic variations.

**Conclusion:**

Our results collectively provide insights into the extent to which adaptive variation is governed by sequence diversity within the ***O. glaberrima*** genome, paving the way for in-depth studies of the genetic basis of traits of interest that might be useful to the rice breeding community.

## Background

African rice, *Oryza glaberrima* Steud., was domesticated independently of Asian rice *Oryza sativa* L. (Wang et al. 2014; Meyer et al. 2016; Cubry et al. 2018; Choi et al. 2019). Its domestication took place in the inner delta of the Niger river (Cubry et al. 2018), from a wild relative species, *Oryza barthii* A. Chev.. Its origin from this wild Sahelian species explains its strong tolerance or resistance to biotic and abiotic stresses (Sarla and Swamy 2005). In the context of increasing temperatures and a more variable climate, strong tolerance to such stresses is an important objective for rice agriculture worldwide. However, knowledge of the genetic basis of phenotypic variation in African rice remains very limited. With the exception of salinity tolerance (Meyer et al. 2016), few association studies have been performed for traits of agricultural interest in this species. Genome wide association studies (GWAS) have successfully identified genes of functional importance associated with flowering time in Asian rice (Zhao et al. 2011; Huang et al. 2012; Yano et al. 2016). For Asian rice, the genetic determination of this trait is well understood (Lee and An 2015), whereas we have no information about the variation of this trait for African rice. Another trait of broad interest for rice farmers and breeder communities is the architecture of the panicle. This trait is one of the main components of yield potential, because the number of seeds per panicle is directly related to the branching complexity of the inflorescence (Xing and Zhang 2010). With increasing global movement of plant material and climate change, biotic threats to rice agriculture continue to evolve and the search for new sources of resistance to pathogens is therefore a challenging research field. *Rice yellow mottle virus* (RYMV) is responsible for one of the most damaging diseases of rice in Africa (Kouassi et al. 2005; Issaka et al. 2012; Kam et al. 2013). Resistance genes against RYMV are mostly found in *O. glaberrima* (Pidon et al. 2020), and this species may be an interesting source of quantitative trait loci (QTLs) for global rice breeding strategies (Thiémélé et al. 2010).

To better assess the functional variation present in African rice, we developed a genome-wide association panel and corresponding phenotypic datasets for flowering time, inflorescence architecture, and resistance to RYMV. Using several complementary statistical methods for association genetics, we identified key genomic regions for flowering time variation, panicle architecture, quantitative resistance to RYMV and climatic variation.

## Results

We took opportunity of previously built genomic resources for a panel of 163 African rice genotypes (Cubry et al. 2018) to address the genetic determinant of important agronomic traits using several GWAS methods.

The phenotypic data were obtained from infield experiments (for panicle architecture and flowering time), greenhouse experiments (RYMV resistance) or from available public databases for environmental data (Table 1; details in Additional file 1: Table S1). A Box-Cox transformation was applied to all variables except early flowering (DFT2012a, DFT2014a) and primary branch internode average length (PBIntL2012, PBIntl2014), as for these variables both data from 2012 and 2014 fitted a normal distribution (Additional file 2: Table S2; Additional file 3: Fig. S1). Heritability ranged from 0.52 to 0.89 for the different experimental variables, showing the phenotype was strongly linked to genetic variation (Additional file 4: Table S3).

**Table 1:** List of traits and variables used for the association study

Category	Trait	Source	Variables	Transformation used?
**Flowering**	Early sowing	Field	DFT2012a	no
			DFT2014a	no
	Late sowing	Field	DFT2012b	yes
			DFT2014b	yes
**Panicle**	Rachis length (RL)	Field	RL2012	yes
			RL2014	yes
	Spikelet number (SpN)	Field	SpN2012	yes
			SpN2014	yes
	Primary branch number (PBN)	Field	PBN2012	yes
			PBN2014	yes
	Secondary branch number (SBN)	Field	SBN2012	yes
			SBN2014	yes
	Primary branch average length (PBL)	Field	PBL2012	yes
			PBL2014	yes
	Secondary branch average length (SBL)	Field	SBL2012	yes
			SBL2014	yes
	Primary branch internode average length (PBintL)	Field	PBintL2012	no
			PBintL2014	no
	Secondary branch internode average length (SBintL)	Field	SBintL2012	yes
			SBintL2014	yes
**Resistance**	Resistance to Rice yellow mottle virus (RYMV)	Greenhouse	RYMV1	yes
			RYMV2	yes
			RYMV3	yes
**Environment**	Climate-related variables principal component (bioPC)	Database	bioPC1	yes
			bioPC2	yes
	Monthly maximum temperature principal component (Tmax)	Database	tmaxPC1	yes
			tmaxPC2	yes

A total of 892,539 SNPs previously identified (Cubry et al. 2018) from 163 different *O. glaberrima* accessions was used in this study. The genome-wide linkage disequilibrium was high at short distance and slowly decayed with increasing genomic distance. It remained above 0.2 for at least 150 kb (Additional file 5: Fig. S2). This is in accordance with previously published results (Cubry et al. 2018).

Genetic structure is rather subtle, with a cross-entropy criterion that decreased slowly with increasing K (Additional file 6: Fig. S3a). However, we assumed that the number of ancestry groups that best explained our data was four, with a subtle elbow in the cross-entropy curve at this point. Ancestral population membership estimated for the genotypes allowed to identify four groups but with a lot of mixture (Additional file 6: Fig. S3b). The retained number of four was subsequently used as an input for some genetic association methods to correct for population structure.

The geographic spanning of phenotypic variables and correlation with genetic structure was determined. No clear geographic clustering or strong correlation between phenotypic data and genetic structure was observed for any variable (Additional file 7: Fig. S4; Additional file 8: Fig. S5).

Three GWAS methods, namely efficient mixed model analysis (EMMA, Kang et al. 2008), mixed-linear model (MLM, Zhang et al. 2010) and latent factor mixed model (LFMM, Frichot et al. 2013) were applied to identify associations between genomic polymorphisms and phenotypic variables. Using a non-corrected analysis of variance (ANOVA) as a benchmark, all methods allowed an efficient correction for false positives linked to genetic structure (QQ-plots, see Additional files 9: Fig. S6). A total of 1976 SNP/trait associations were detected at 10^− 5^ *p*-value threshold for all the traits tested (Table 2). About 25% of them were detected with at least two methods (Table 2; Additional file 12: Table S4). The EMMA method detected a higher number of SNPs than the two others for most of the traits and the MLM method detected only 13% of the associations; however contrasted results were observed depending on the traits. A total of 82 candidate genomic regions associated to 10 different variables was identified, considering a 50 kb genomic window around each significant SNP and retaining only regions consistent with at least two methods.

**Table 2:** Numbers of significant SNPs, regions and genes found to be associated with the different traits. Significant SNPs were detected with the different models using the Fisher combination method and based on a 10^− 5^ *p*-value threshold. The “EMMA”, “MLM” and “LFMM” columns indicate the number of SNPs detected for each method. The “2_met” and “3_met” columns indicate the number of SNPs detected by two or three methods respectively. Fifty kb windows around these SNPs defined independent genomic regions associated with each variable and only regions containing SNPs detected with at least two methods were retained

Trait	Significant SNPs					Regions
	EMMA	MLM	LFMM	2_met	3_met	
**BioPC1**	17	0	0	0	0	0
**BioPC2**	5	0	8	0	0	0
**TmaxPC1**	10	0	0	0	0	0
**TmaxPC2**	211	3	89	89	3	16
**Early Sowing**	24	1	657	17	0	6
**Late Sowing**	191	2	141	105	0	5
**RYMV**	454	124	90	120	65	31
**SpN**	4	0	0	0	0	0
**PBN**	0	7	0	0	0	0
**SBN**	14	14	1	8	0	4
**RL**	37	18	2	3	1	3
**PBL**	37	30	23	30	23	1
**SBL**	66	2	79	63	2	10
**PBintL**	21	4	2	4	2	1
**SBintL**	53	119	0	49	0	5

### Flowering Time

**Fig. 1:**
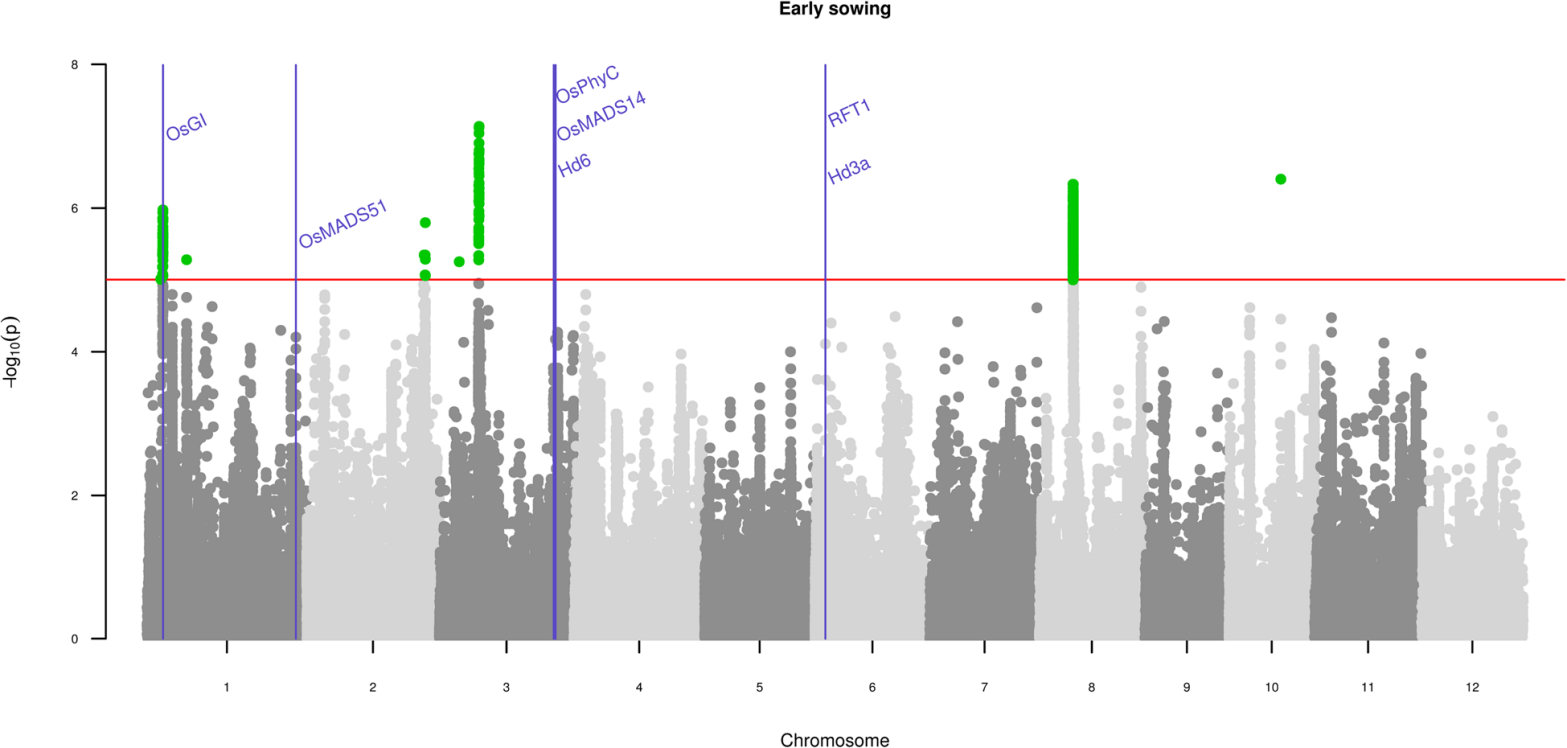
Manhattan plot of LFMM association results for flowering time assessed for early sowing. The red line indicates the 10^−5^ *p*-value threshold. SNPs exhibiting lower *p*-values than the threshold are indicated as green dots. Positions of known Asian rice flowering genes listed in the expert list (see text) are indicated by vertical blue lines

The genome-wide association study of flowering time based on data from the early planting dates allowed us to identify 664 non-redundant SNPs statistically associated with this trait for at least one method (10^− 5^ *p*-value threshold, Table 2; Additional file 12: Table S4). Most of these SNPs were at a distance of less than 25 kb from each other and were clumped into six genomic regions detected by at least two methods (Table 2; Additional file 12: Table S4; Additional file 13: Table S5). Corresponding analyses performed using data from the later planting date revealed a lower number of significant associations: 229 non-redundant significant SNPs resulting in five genomic regions detected with at least two methods (Table 2; Additional file 12: Table S4; Additional file 13: Table S5). Taken together, these two variables helped to define nine regions associated with variation in flowering time (Additional file 13: Table S5). These regions encompassed 79 genes (Additional file 14: Table S6). One GWAS peak for both early and late sowing co-localized with a known Asian rice flowering time gene (Table 2; Fig. 1): *OsGI* on chromosome 1.

Using an expert list based on bibliography, we checked the enrichment in genes known to be involved in flowering among the 79 candidate genes (Additional file 9: Table S5). Considering that we detected one gene from this list, this resulted in a twelve-fold enrichment, not significant using a G-test. Considering a 10^− 4^ *p*-value less stringent threshold and only one method for the detection of association resulted in the identification of 1025 genes. Six of them belonged to our expert list, corresponding to a five-fold significant enrichment (Additional file 15: Table S7).

### Panicle Morphological Traits

A total of 344 non-redundant significant SNPs was detected for at least one of the panicle morphological traits at 10^− 5^ *p*-value threshold for any of the three methods used (Table 2; Additional file 12: Table S4). Twenty-three unique genomic regions associated with one or more morphological traits were identified (Table 2; Additional file 13: Table S5). One of them (rOg-PAN-14) was associated with two traits: secondary branch number (SBN) and secondary branch average length (SBL). The 22 remaining regions were each associated with a single trait: 3 with SBN, 3 with Rachis length (RL), 1 with primary branch average length (PBL), 9 with SBL, 1 with PBintL and 5 with secondary branch internode average length (SBintL). No region associated with spikelet number (SpN) and primary branch number (PBN) traits was selected, as significative SNPs for these traits were detected with a single method only (Table 2). Over the 23 associated regions, 283 annotated genes were identified (Additional file 14: Table S6). Among those genes, two were already known to be associated to panicle development in *O. sativa* (i.e. spikelet number and secondary branch number): *NARROW LEAF8* (*NAL8*) which co-localized with the rOg-PAN-15 region (Chen et al. 2019) and *FACTOR OF DNA METHYLATION LIKE1* (*OsFDML1*) with the rOg-PAN-3 region (Tao et al. 2018), both associated with SBL trait in our study (Additional file 14: Table S6).

### RYMV Resistance

**Fig. 2:**
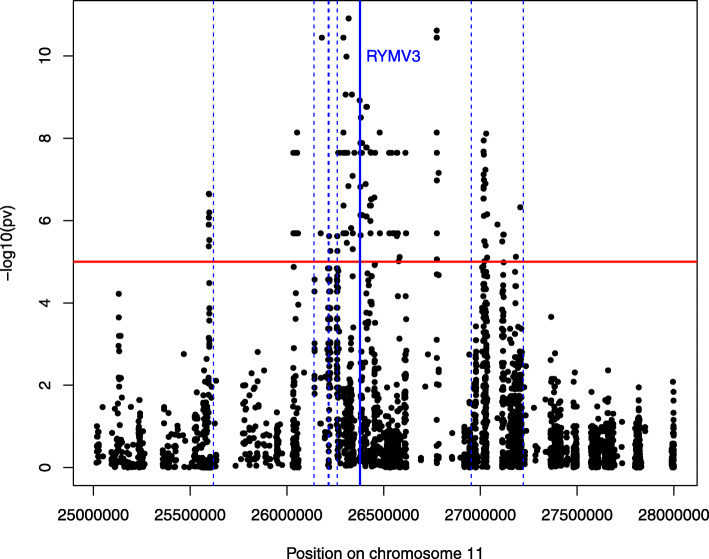
Details of the Manhattan plot obtained with EMMA on the region associated to *RYMV* resistance on chromosome 11. The 10^− 5^ *p*-value threshold is represented by a red horizontal line. Positions of the major resistance gene *RYMV3* is indicated by plain vertical blue lines and positions of other *NLR* genes on chromosome 11 are indicated by dotted blue lines

Quantitative resistance to RYMV was found to be associated with 483 SNPs detected with any of the three methods and delimiting 31 regions detected with at least two methods (Table 2; Additional file 12: Table S4; Additional file 13: Table S5). Two thirds of these SNPs were clustered in six regions defining a 1.8 Mb interval around position 26,5 Mb on chromosome 11, which underlined the major role of this genomic interval in RYMV resistance. A total of 287 candidate genes were identified in the candidate regions (Table 2; Additional file 14: Table S6). Interestingly, the high resistance gene *RYMV3* is located at position 26,4 Mb on chromosome 11 (Pidon et al. 2017), in the cluster of candidate regions identified on this chromosome (Fig. 2).

### Environment-Related Variables

In order to study association with environmental variables, we downloaded climatic variables for 107 geolocalised accessions (Cubry et al. 2018) from the worldclim v1.4 database (Hijmans et al. 2005). The first two axes of a PCA on bioclimatic data (BioPC1 and BioPC2) explained 49.91% and 26.32% of the variance of all variables. BioPC1 was mainly explained by temperature seasonality (bio4) and precipitation of driest quarter (bio17) while BioPC2 was mainly explained by mean temperature of driest quarter (bio9) and mean temperature of coldest quarter (bio11). We evaluated the statistical association of genetic polymorphisms with the first and second axes of the PCA. Using our threshold, we detected 17 and 13 significant associations for BioPC1 and BioPC2 respectively (Table 2). However, no genomic region was detected with at least two methods.

When considering maximum temperature variables (Tmax), 73.56% and 17.90% of the variance was explained by the first PCA axis (TmaxPC1) and second PCA axis (TmaxPC2) respectively. Ten associations were found considering TmaxPC1, while TmaxPC2 allowed us to detect 211 SNPs. Considering genomic regions detected by at least two methods, we found 16 regions for TmaxPC2 only (Table 2; Additional file 13: Table S5). When intersected with genome annotation, these regions encompassed 167 genes (Additional File 14: Table S6).

## Discussion

The *O. glaberrima* collection used in this study comprised 163 accessions, of which not all were included for some traits. This limited number of accessions likely do not allow the detection of associations involving low frequency variants. It could also have impaired the detection of any association for traits strongly correlated to genetic structure. However, thanks to the weak structure of this population, and more generally the *O. glaberrima* species (Cubry et al. 2018; Additional file 6: Fig. S3), it still allowed the detection of significant associations. We retained a 10^− 5^ *p*-value cutoff which is more relaxed than a Bonferroni threshold. However, we selected only genomic regions consistent with two methods, which limits false positives and give a broad scale idea about polymorphisms associated with important phenotypic variables. Considering this threshold, seventy-nine distinct genomic regions were found to be associated to phenotypic traits related to flowering date, panicle architecture, virus resistance and environmental traits. A false discovery rate (FDR) 5% threshold frequently used in GWAS would have retrieved a far larger number of SNPs and regions. Most of the regions identified in this study would also have been identified with a FDR 5% threshold, confirming the stringency of our approach. Given the extent of the LD within our sample, we lack power to address fine dissection of quantitative traits. We chose a rather conservative approach by defining large genomic windows around the SNPs we detected as significantly associated with one of our traits of interest. The lists of genes we identified likely contain a large number of non-associated ones and therefore should be considered with caution. More studies, including functional ones, will be needed to precise which genes are good candidates for the phenotypic traits variation.

### Limited Overlap of Flowering Time and Panicle Architecture Genetic Networks Between African and Asian Crop Species

The flowering pathway is a well described pathway in Asian rice *O. sativa*, with several known key genes (Tsuji et al. 2011; Hori et al. 2016). Based on our expert list of known flowering genes, we were able to identify a peak that co-localized with a known Asian rice flowering gene, *OsGI*. However, using a G-test, this enrichment was not significant, certainly due to the fact that we had only one gene in our test. Using a less stringent GWAS threshold enabled to achieve a significant enrichment, suggesting our method could significantly retrieve genes involved in the variability of this trait. *OsGI* is an ortholog of the *Arabidopsis thaliana GIGANTEA* gene and its over-expression in rice leads to late flowering under both short day and long day conditions (Hayama et al. 2003). Eight additional peaks were detected with our analysis. These peaks are good candidates for further analysis in order to identify novel genetic diversity relating to the flowering pathway of African rice. Among those peaks, four were specific to early sowing and three to late sowing. This is not surprising as those two conditions likely address different flowering regulation pathways. A study in *A. thaliana* found for example less than 10 overlapping QTLs over 37 in total between two different conditions (Brachi et al. 2010). The peak common to both sowing conditions encompassed a total of 13 genes. Among those, a *CO-like* family gene is present (*LOC_Os08g15050*). This gene might be of interest as several genes from this family are involved in flowering control in long day or short day conditions in rice, as well as several homologs in *A. thaliana,* especially *CONSTANS* (*CO*) gene known to promote flowering in long day conditions (Putterill et al. 1995; Zhang et al. 2017).

Spikelet number per panicle and primary branch number were the main traits contributing to the diversity of panicle architecture observed in this population. However, only few SNPs were identified for the SpN and PBN traits over the 3 methods used. A possible explanation for this result is that these traits might be associated with a large number of QTLs of low effect sizes, and may consequently be difficult to assess using the present GWAS panel. Several genes implicated in the regulation of panicle development and/or architecture were previously reported in *O. sativa* (Xing and Zhang 2010; Wang and Li 2011; Teo et al. 2014). Only two genes recently characterized, namely *NAL8* and *OsFMDL1,* which are associated to spikelet number and the development of leaf and flower, respectively, (Chen et al. 2019; Tao et al. 2018) were associated to panicle morphological trait diversity in *O. glaberrima* using our cutoff. Several association studies of panicle morphological trait diversity have been recently conducted for *O. sativa* (Bai et al. 2016; Crowell et al. 2016; Rebolledo et al. 2016; Ta et al. 2018; Yano et al. 2019). Only a few overlaps of GWAS regions were observed between the two rice crop species, including a cluster of GWAS sites related to panicle and yield traits reported on chromosome 4 in *O. sativa* (Crowell et al. 2016).

This would suggest that the intra-specific variation in the African rice species for flowering time and panicle architecture might rely more on specific factors, without excluding the fact that the orthologs of genes identified in *O. sativa* are also important for the control of these traits in *O. glaberrima*. For the specific genomic regions identified in this study, further work should lead to the precise identification of genetic elements governing diversity in African rice.

### Quantitative Resistance to RYMV in *O. glaberrima* and Major Resistance Genes

The regions identified as being associated with resistance against RYMV did not overlap with QTLs of partial resistance against RYMV previously identified in *O. sativa* (Boisnard et al. 2007), suggesting that different genes and pathways may lead to resistance. However, *RYMV3,* a major resistance gene against RYMV identified in *O. glaberrima* species, is located in the main cluster of regions found to be associated with quantitative resistance in this study. The main candidate resistance gene for *RYMV3* belongs to the family of nucleotide-binding domain and leucine-rich repeat containing (NLR) genes (Pidon et al. 2017), many of which are involved in pathogen recognition and effector-triggered immunity (de Ronde et al. 2014). NLR genes frequently act as determinants of high and monogenic resistance (de Ronde et al. 2014) but a role in quantitative resistance has also been clearly established (Wang et al. 1999; Hayashi et al. 2010). NLR genes are known to be frequently organized into clusters and several additional NLR genes, annotated in the close vicinity of the *RYMV3* candidate gene might also be good candidates for quantitative resistance. The *RYMV3* gene, or adjacent NLR genes, might thus harbor both alleles with quantitative effects and alleles with strong effects on RYMV resistance. In addition to the above, genes encoding protein domains implicated previously in plant-virus interaction, such as dnaJ domain containing protein (Lu et al. 2009; Zong et al. 2020) or kelch repeat protein (Thiel et al. 2012), were also found to be located in or close to resistance associated regions (Additional file 14: Table S6). Further studies should be conducted to characterize the different candidate genes and the diversity of resistance pathways to RYMV in African rice.

### Relationship Between Environment-Related Variables and *O. glaberrima* Diversity

We did not find any significant association between the first PCA axis for either the set of bioclimatic variables or the monthly average maximal temperature. This can be due to the highly polygenic determination of these traits and the limited power of our setup to detect small effect variants.

Several candidate regions in relation with temperature variables have been identified that will require a more in-depth study in order to gather variations of interest for breeding purposes (see Additional file 14: Table S6). Such regions might encompass interesting genes for genetic improvement in the context of a changing climate. More studies will have to develop on this basis and we provide here the first list of candidate polymorphisms linked to environment response in the African rice.

## Conclusions

We report on the results of an extensive Genome Wide Association Study carried out for several traits of agronomical interest on African rice. As of interest for farmers and breeders, we also carried out the first GWAS analysis to date of climate variables in relation to African rice.

Our analysis pinpointed some genes already identified as key factors for the different traits studied as candidate genes. For instance, RYMV quantitative resistance may involve the major resistance gene *RYMV3*, and flowering time diversity may also be controlled by the ortholog of *OsGI* gene, as previously reported in *O. sativa*. Identifying adaptive polymorphisms among these candidates and functional validation will be needed to reinforce our results.

Besides, other associated regions did not contain any obvious candidate genes, suggesting that *O. glaberrima* likely harbors an original diversity. Interestingly, for all the characters studied, most of the genomic regions identified were specific to the *O. glaberrima* species compared to *O. sativa*, suggesting that the intra-specific variation in the African rice species for *RYMV* resistance, flowering time and panicle architecture might rely on specific factors. Further studies will lead to the precise identification of genetic elements governing diversity and local adaptation, resistance or tolerance to biotic and abiotic stresses in African rice.

## Material and Methods

### Genotypic Data

Single nucleotide polymorphisms (SNPs) from 163 high-depth re-sequenced *O. glaberrima* accessions were used in this study (Cubry et al. 2018). SNPs were identified based on mapping to the *Oryza sativa japonica* cv. Nipponbare high quality reference genome in terms of assembly and annotation (Kawahara et al. 2013). The bioinformatic mapping pipeline, software and SNP filtering steps that were used are described in Cubry et al. (2018).

SNPs with more than 5% missing data (minor fraction of total SNP set) were filtered out (Cubry et al. 2018). As missing data can reduce the power of association studies (Browning 2008; Marchini and Howie 2010), we imputed the remaining missing data based on a matrix factorization approach using the “impute” function from the R package LEA (Frichot and François 2015). This approach uses the results f ancestry estimation from a sparse non-negative matrix factorization (sNMF) analysis to infer missing genotypes (Frichot et al. 2014). In sNMF, we set K to infer four clusters and kept the best out of 10 runs based on a cross entropy criterion.

### Phenotyping of Flowering Time and Panicle Morphology

Phenotyping of flowering time and panicle morphology was performed near Banfora (Burkina-Faso) under irrigated field conditions at the Institut de l’Environnement et de Recherches Agricoles (INERA) station in 2012 and 2014. Plants were sown at two different periods in the same year: the first at beginning of June (“early sowing”) and second in mid-July (“late sowing”). A total of 15 plants per plot of 0.5 m^2^ were grown. The field trials followed an alpha-lattice design with two replicates (Patterson and Williams 1976) per date of sowing per year. Each single block included 19 accessions (i.e. 19 plots). In total, 87 *O. glaberrima* accessions were planted in 2012 and 155 in 2014.

Flowering date (DFT) was scored when 50% of the plants for a given accession harbored heading panicles for both early and late sowings in 2012 and 2014 (Table 1). Fourteen days after heading date, the three main panicles from three central plants per plot per repeat were collected (i.e. nine panicles/accession/repeat) from the early sowing only, over the 2 years. Each panicle was fixed on a white paper board, photographed and phenotyped using the P-TRAP software allowing the quantification of eight morphological traits (AL-Tam et al. 2013) (see Table 1). All statistical analyses of the dataset were performed using R (R core team 2020) packages ade4 (Dray and Dufour 2007) and corrplot (Wei and Simko 2017) as described in Ta et al. (2018).

### RYMV Resistance Phenotyping

Resistance was evaluated based on ELISA performed on infected plants cultivated in the greenhouse, under controlled conditions. As high resistance to RYMV has been already well studied in African rice, we excluded highly resistant accessions, i.e. in which no virus can be detected with ELISA (Pidon et al. 2020), and we focused only on quantitative resistance. We therefore assessed resistance on a set of 125 accessions. Two varieties were used as susceptibility controls, IR64 (*O. sativa* ssp. *indica*) and Nipponbare (*O. sativa* ssp. *japonica*), and one as a high resistance control, Tog5681 (*O. glaberrima*). Three replicate experiments of all varieties were performed. In each experiment, plants were organized in two complete blocks with four plant replicates per accession.

Plants were mechanically inoculated 3 weeks after sowing with CI4 isolate of RYMV (Pinel et al. 2000). A single batch of inoculum for all replicate was prepared, plants were inoculated with a needleless syringe on two points at the basis of the last emerged leaf. Four discs of 4 mm diameter were cut on the last emerged leaf of each plant 17 and 20 days after inoculation (dai) and discs from the four plants of the same block were pooled. Samples were ground with a QIAGEN TissueLyser II bead mill and resuspended in 750 μL 1X PBST (Phosphate buffer saline with Tween 20). Virus content was estimated by DAS-ELISA (Pinel-Galzi et al. 2018). Preliminary tests on a subset of samples were performed to assess the dilution that best discriminated between samples. ELISA tests were finally performed at dilutions of 1/1000 for 17 dai sampling date and 1/2500 for 20 dai sampling date. Optical density values were normalized according to a standard range of virus dilutions loaded on each ELISA plate in order to correct a putative plate effect and the average of the measures of the two blocks was calculated in each replicate experiment. As virus content was highly correlated between 17 and 20 days after infection (*R*^2^ = 0,81), the resistance level was estimated as the mean of the two sampling dates. Resulting variables were named RYMV1, RYMV2 and RYMV3 for the three different experiments (Table 1).

### Environmental Variables

For accessions with geographical sampling coordinates, we retrieved information for 19 climate-related variables (referred to here as bio) from the WORLDCLIM database at a 2.5 min resolution (Hijmans et al. 2005). We also retrieved the average monthly maximum temperature (referred to here as Tmax). We first performed a Principal Component Analysis (PCA) on each set of variables to build uncorrelated composite variables. PCA were performed using R package LEA (Frichot and François 2015). Association studies were performed using the first two components of each PCA (Table 1).

### Treatment of Phenotypic and Environmental Variables

For each variable (Table 1; Additional file 1: Table S1a), we plotted the histogram of the trait distribution data as well as a quantile-quantile plot to visually assess the normality (Additional file 3: Fig. S1). We additionally performed two tests of normality, the Shapiro-Wilkinson’s and the Anderson-Darling’s statistics (Additional file 2: Table S2a). These analyses were made using the base graphics and nortest (Gross and Ligges 2015) packages for R.

As some of the variables did not fit a normal distribution, we applied a Box-Cox transformation of the data to approximate the normality (Table 1; Additional file 1: Table S1b). To do this transformation, we used the forecast package for R (Hyndman and Khandakar 2008).

The Box-Cox transformation writes as follow:

$$ B\left(x,\lambda \right)=\frac{x^{\lambda }-1}{\lambda }\ \mathrm{if}\ \lambda \ne 0\ \mathrm{and}\ B\left(x,0\right)=\log (x)\ \mathrm{if}\ \lambda =0 $$

We estimated the λ parameter of the transformation using the BoxCox.lambda() function with the « loglik » argument (i.e. using a maximum log likelihood approach). We then applied the transformation using the estimated λ with the BoxCox() function. As some variables (typically the environment variables) had some negative values that could prevent the use of the transformation, we used a translation of the data whenever negative values occurred in the variable with the following formula: *f*(*x*) = *x* + 1 − min(*x*) prior to apply the Box-Cox transformation. The histograms and quantile-quantile plots have been made again, as well as the normality tests for the resulting transformed variables (Additional file 2: Table S2b and Additional file 3: Fig. S1).

Apart from climate-related variables, each trait resulted from the combination of at least two repetitions. If one of the repetitions failed to reach the normality test, we used the transformed dataset for all repetitions. For climate variables, we used the transformation whenever the variable failed to pass the normality test.

Heritability was estimated for the following phenotypic trait: flowering time, panicle morphology and resistance to RYMV virus. We used a mixed model to estimate the inbred line variance, the block, the year and the residual variance. Raw (untransformed) data was used for this specific analysis. Heritability was calculated as the ratio of the line variance divided by the line variance and the residual variance (https://plant-breeding-genomics.extension.org/estimating-heritability-and-blups-for-traits-using-tomato-phenotypic-data/).

### Linkage Disequilibrium

In order to assess the limits of the GWAS analysis, we computed the genome-wide Linkage Disequilibrium (LD) of our sample using the PopLDdecay software (Zhang et al. 2019). We used the imputed VCF as an input and specified the default parameters both for the analysis and the plotting. The genome-wide LD decay was then visually assessed.

### Genetic Structure Assessment

In order to efficiently control for the confounding effect of individual’s relatedness, we assess the population genetic structure of our sample using the sparse non-negative matrix factorization (sNMF) approach implemented in the R package LEA. We assumed a number of ancestral groups (K) between one and 10 and we made five repetitions of the algorithm for each K. In order to evaluate which K best describe our data, we computed the cross-entropy criterion for each K and plotted it. We then selected the run for the considered K which exhibited the lowest cross-entropy and used it to plot the ancestries coefficient of each genotype. The estimated K was subsequently used as an input for some association genetics methods.

### Geographic Mapping of Phenotypic Variables and Link with Genetic Structure

We used the raw data to compute mean values of the quantitative traits under consideration in this study for the accessions having sampling coordinates in their passport data. We then plotted these data using the ggplot2 (Wickham 2016) package for R.

To assess the impact of genetic structure on the phenotypic variables, we computed the Spearman’s rank correlation between the raw phenotypic values and each of the ancestry components retained using the rcorr function of the Hmisc R package (Harrell 2019). We then plotted the resulting matrix as a correlogram using the R package corrplot (Additional file 8: Fig. S5). To assess the significance of the results, we used either a *p*-value < 0.01 threshold (Additional file 8: Fig. S5a) or an FDR approach with a 5% threshold (Additional file 8: Fig. S5b), calculated using the qvalue R package (Storey et al. 2019).

### Association Studies

For each trial, SNPs displaying a minimal allele frequency (frequency of the minor allele) lower than 5% were filtered out. We first adjusted a simple linear model (Analysis of variance, ANOVA) to associate phenotype and genotype. This simple method did not take into account any putative confounding factor and allowed us to assess whether taking into account relatedness and/or population structure could reduce false positive rates. Two classes of methods accounting for confounding factors were used: 1) mixed models using kinship matrix and/or population structure (Yu et al. 2006); and 2) latent factor methods (Frichot et al. 2013). We used both mixed linear models MLM (Zhang et al. 2010) as implemented in GAPIT R package (Lipka et al. 2012) and EMMA (Kang et al. 2008) as implemented in R package EMMA. For EMMA, the kinship matrix was estimated using the emma.kinship function. For MLM (Q + K model), the kinship (K matrix) was computed using the Van Raden method and the first three principal components (PCs) of a PCA of genomic data were used as the Q matrix. The PCs were used to correct for population structure only for the MLM method. Finally, we used latent factor methods (Frichot et al. 2013) that jointly estimated associations between genotype and phenotype and confounding factors. We used the R package LFMM2 (Caye et al. 2019) to perform these analyses. We first made the estimation of the confounding factors by using a subset of SNPs obtained by applying a 20% MAF filter, and we considered four latent factors (Cubry et al. 2018). We then used the resulting confounding matrix for the analysis of genotype/phenotype association. The results of all analyses were graphically represented by using a QQ-plot to assess confounding factor correction and Manhattan plots (R package qqman, Turner 2014). We used a 10^− 5^ *p-value* threshold to select candidate SNPs for each method. An additional false discovery rate (FDR) estimation was realized using the R package qvalue (Storey et al. 2019).

GWAS analysis was performed separately for each year and trial (see Additional file 1: Table S1). *P*-values obtained for the same traits or the same planting data were combined across experiments using Fisher’s method (Sokal and Rohlf 2012). We defined genomic regions for each trait using a genomic window approach, i.e. when two consecutive significant SNPs were distant from less than 50 kb, they were clumped together in the same region. We finally applied a filter on the selected regions by considering as candidate regions those detected at least by two methods. Annotation of retained candidate regions was performed by intersecting the candidate regions with the genome annotation data for MSU7 (Kawahara et al. 2013), considering genes within the defined region and extending 25 kb upstream and 25 kb downstream.

Finally, for flowering traits, we established a list of known genes of particular interest from published data (Tsuji et al. 2011; Hori et al. 2016). This “expert” list was then used to assess the performance of our GWAS approach to retrieve these potential candidates. We used a G-test to assess enrichment of candidates in our list of identified genes.

## Supplementary information

**Additional file 1: Table S1.** Phenotypic data used for genome-wide association analyses. Flowering time (DFT), rachis length (RL), primary branch number (PBN), primary branch average length (PBL), secondary branch average length (SBL), primary branch internode average length (PBintL), secondary branch number (SBN), secondary branch internode average length (SBintL) and spikelet number (SpN) were evaluated in field conditions in 2012 and 2014. Resistance to RYMV was evaluated in greenhouse conditions during three experiments (RYMV1, RYMV2, RYMV3) shifted of about 1–2 months. Environmental data were extracted from the worldclim database at available sampling locations. A Principal Component Analysis was then performed on i) the whole set of variables and ii) only maximal temperature related ones. The two first axes of both these PCA were then used for association analyses and are reported in this table. A Box-Cox transformation was applied to the different data set in order to approximate the normality. The “T_” prefix was added to variables names to distinguish non-transformed and transformed variables. (XLS 161 kb)

**Additional file 2: Table S2.** Statistics of the Shapiro-Wilkinson and Anderson-Darling normality tests, applied to the different non-transformed (a) and transformed variables (b).

**Additional file 3: Figure S1.** Histograms of trait distribution and quantile-quantile plots for each non-transformed and transformed variables.

**Additional file 4: Table S3.** Heritability estimates of the different traits. Heritability was estimated for each phenotypic trait: flowering time, panicule morphology and resistance to RYMV virus. The analysis was done for flowering time (FT), spikelet number (SpN), primary branch number (PBN), secondary branch number (SBN), rachis length (RL), primary branch average length (PBL), secondary branch average length (SBL), primary branch internode average length (PBintL), secondary branch internode average length (SBintL) and RYMV virus content based on the mean of estimation at 17 and 20 days after infection (RYMV). We used a mixed model to estimate the inbred line variance, the bloc, the year and the residual variance. Heritability was calculated as the ratio of the line variance divided by the line variance and the residual variance (https://plant-breeding-genomics.extension.org/estimating-heritability-and-blups-for-traits-using-tomato-phenotypic-data/). (ODS 10 kb)

**Additional file 5: Figure S2.** Genome wide Linkage disequilibrium (LD) decay.

**Additional file 6: Figure S3.** Structure of the population. (a) evolution of the cross-entropy criterion with increasing K, (b) bar plot of ancestries membership considering K = 4 ancestral population.

**Additional file 7: Figure S4.** Geographic distribution of traits. We plotted the mean value of each trait for accessions having sampling location in their passport data.

**Additional file 8: Figure S5.** Correlations between traits and structure. For each ancestry group (A1 to A4), we made a Spearman’s rank correlation test and plotted it as a correlogram. Colors sign the intensity of the correlation and white stars were added when the correlation was significant given the threshold retained, i.e. either a *p*-value cutoff of 0.01 (a) or a False Discovery Rate of 5% (b).

**Additional file 9: Figure S6.** Linear scale QQ-plots corresponding to association analysis performed independently on each trait and repetition. Three different methods (EMMA, LFMM, MLM) taking into account relatedness and/or structure were used for association and ANOVA was used as a benchmark. For a given trait, the transformed data were used if at least one of the replicates failed to reach normality, otherwise non-transformed data were used.

**Additional file 10: Figure S7.** Manhattan plots. Association analysis were performed independently for each trait and repetition and based on three different methods (EMMA, LFMM, MLM). The transformed data were used if at least one of the replicates failed to reach normality. *P*-values obtained for each replicate were then combined using a Fisher combined probability test method to obtain the final *p*-values represented in this Manhattan plots. The 10^− 5^ thresholds are indicated by red lines.

**Additional file 11: Figure S8.** Log scale QQ-plots corresponding to association analysis performed independently on each trait and repetition. Three different models (i.e. EMMA, LFMM, MLM) taking into account relatedness and/or structure were used for association and ANOVA was used as a benchmark. For a given trait, the transformed data were used if at least one of the replicates failed to reach normality, otherwise non-transformed data were used.

**Additional file 12: Table S4.** List of the SNPs associated with fifteen different phenotypic traits. Significant SNPs were identified based on three different methods (EMMA, LFMM and MLM), the Fisher combined probability test method to combine several repetitions of phenotypic data and a 10^− 5^ *p*-value threshold. For each trait, the *p*-values and q-values obtained with the different methods are indicated when the *p*-values were significant. (XLS 349 kb)

**Additional file 13: Table S5.** List of the genomic regions associated with four different categories of phenotypic traits. Regions were defined based on 50 kb windows around the significant SNPs detected with any of the three models. Overlapping regions were combined into a single one. Only regions detected with at least two methods were retained. The chromosome (Chr), the starting (Position 1) and ending (Position 2) positions, the size of the region (Intervals) in base pairs, the number of significant SNPs included (Sign_SNPs_nb) and the lowest *p*-values obtained with the different methods are indicated. Sheets “RYMV” and “Tmax”concerned the regions identified for the resistance to RYMV and the maximum temperature related variables, respectively. The sheet “Flowering” concerned regions identified with the early sowing flowering time (Early) or the late sowing flowering time (Late) traits, as indicated in the columns “Trait 1” and “Trait 2”. The sheet “Panicle” concerned regions identified with rachis length (RL), primary branch average length (PBL), primary branch internode average length (PBintL), secondary branch number (SBN), secondary branch average length (SBL), and secondary branch internode average length (SBintL), as indicated in the columns “Trait 1” and “Trait 2”.

**Additional file 14: Table S6.** List of genes located in each of the regions associated with four categories of phenotypic traits. Gene ID and annotations refer to MSU7 (Kawahara et al. 2013). The region names refer to the names attributed in Table S5. (XLS 105 kb)

**Additional file 15: Table S7.** Expert list of genes previously described as involved in flowering time in Asian rice and test of enrichment of the list of genes detected by our association analysis. Fold-enrichment and G-test associated *p*-value are reported for our retained threshold (*p*-value cutoff of 10^− 5^ and at least two methods to define a region) and three alternative ones (*p*-value cutoff of 10^− 4^ and two methods; *p*-value cutoff of 10^− 5^; *p*-value cutoff of 10^− 4^).

## Data Availability

All customized R scripts are available as a GitHub repository: https://github.com/Africrop/gwas_african_rice. The repository also contains the imputed genotypic data used here to reproduce exactly the same analysis. Phenotypic data are provided as supplemental material.

## Abbreviations

ANOVA: Analysis of Variance
BioPC1: First principal component of PCA on bioclimatic variables
BioPC2: Second principal component of PCA on bioclimatic variables
dai: Days after inoculation
DFT: Days to flowering
ELISA: Enzyme-linked immunosorbent assay
FDR: False discovery rate
GWAS: Genome wide association study
INERA: Institut de l’environnement et de recherches agricoles
LD: Linkage disequilibrium
NLR: Nucleotide binding leucine rich repeat
PbintL: Primary branch internode average length
PBL: Primary branch average length
PBN: Primary branch number
PCA: Principle component analysis
QTL: Quantitative trait locus
QQ-plot: quantile-quantile plot
RL: Rachis length
RYMV: Rice yellow mottle virus
SbintL: Secondary branch internode average length
SBL: Secondary branch average length
SBN: Secondary branch number
SpN: Spikelet number
SNP: Single nucleotide polymorphism
TF: Transcription factor
TmaxPC1: First principal component of PCA on maximal temperature variables
TmaxPC2: Second principal component of PCA on maximal temperature variables
