# Supplementary material for: Genome Wide Association Study Pinpoints Key Agronomic QTLs in African Rice Oryza glaberrima
Source: Rice (N Y). 2020 Sep 16;13:66. doi: 10.1186/s12284-020-00424-1 (PMC7494698; doi:10.1186/s12284-020-00424-1)
Supplement: Supplementary file 3 — Additional file 3: Figure S1. Histograms of trait distribution and quantile-quantile plots for each non-transformed and transformed variables. [file 12284_2020_424_MOESM3_ESM.pdf]

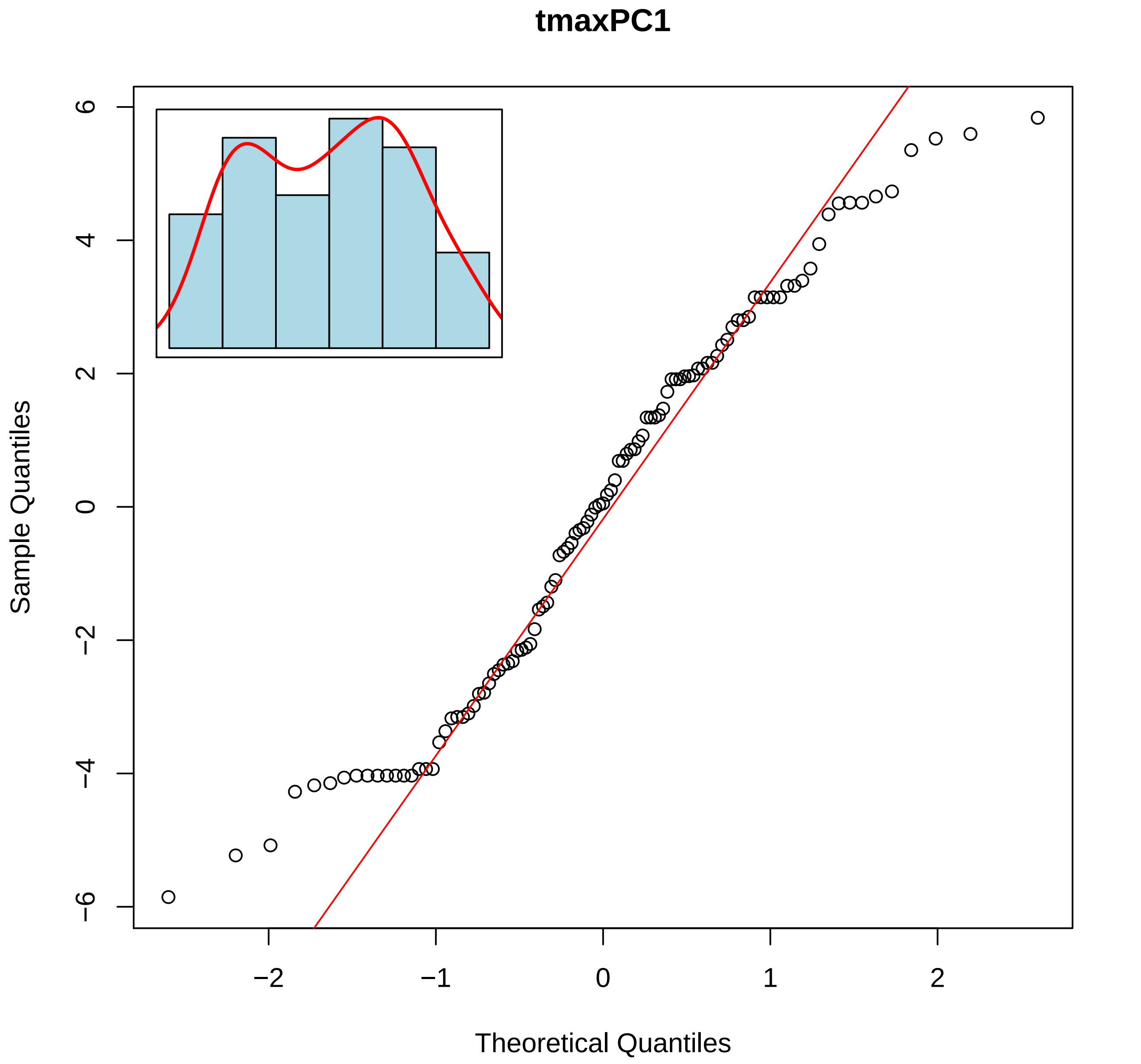
tmaxPC1
Sample Quantiles
Theoretical Quantiles
−6
−4
−2
0
2
4
6
−2
−1
0
1
2

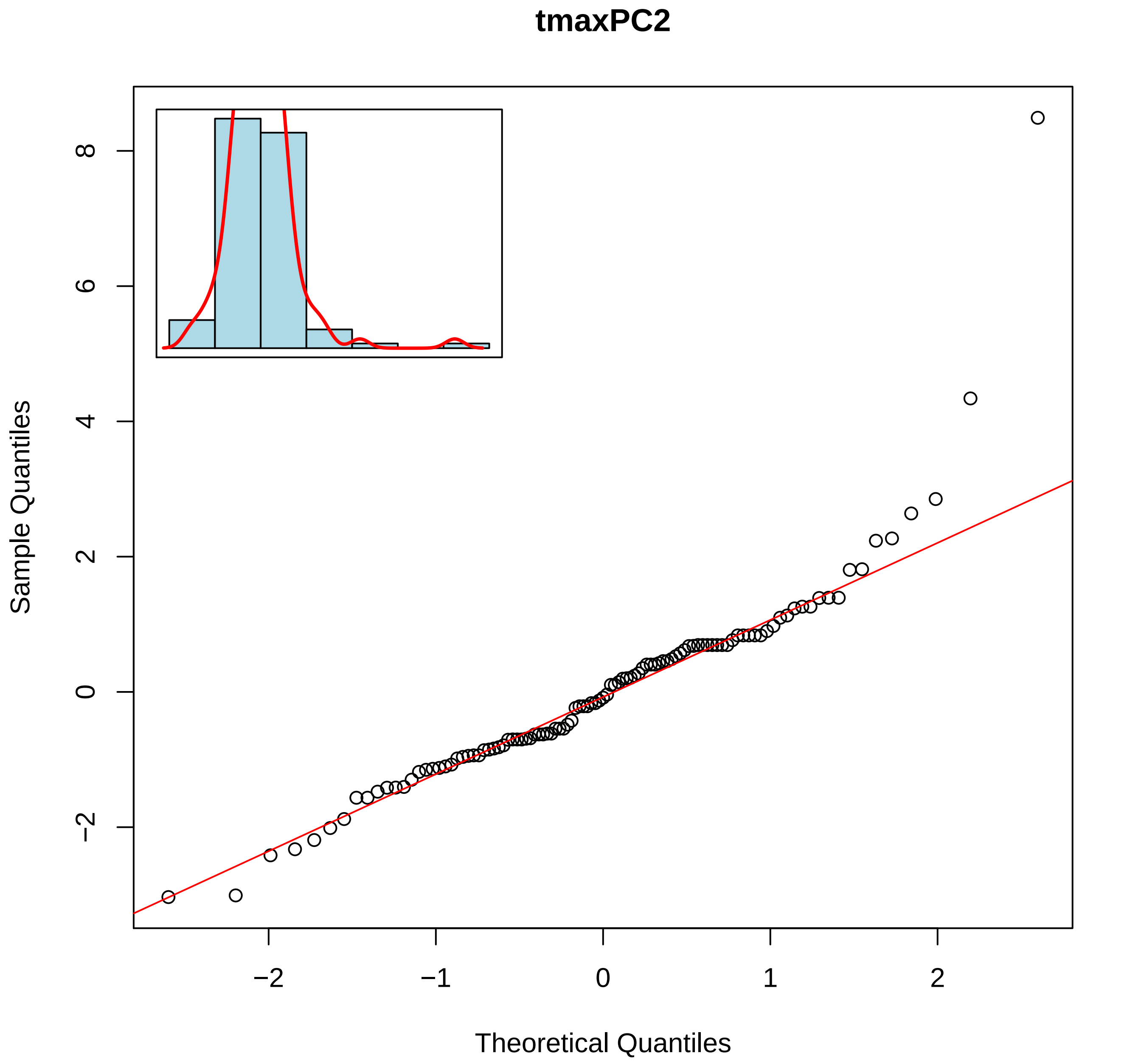
tmaxPC2
Sample Quantiles
Theoretical Quantiles
−2
0
2
4
6
8
−2
−1
0
1
2

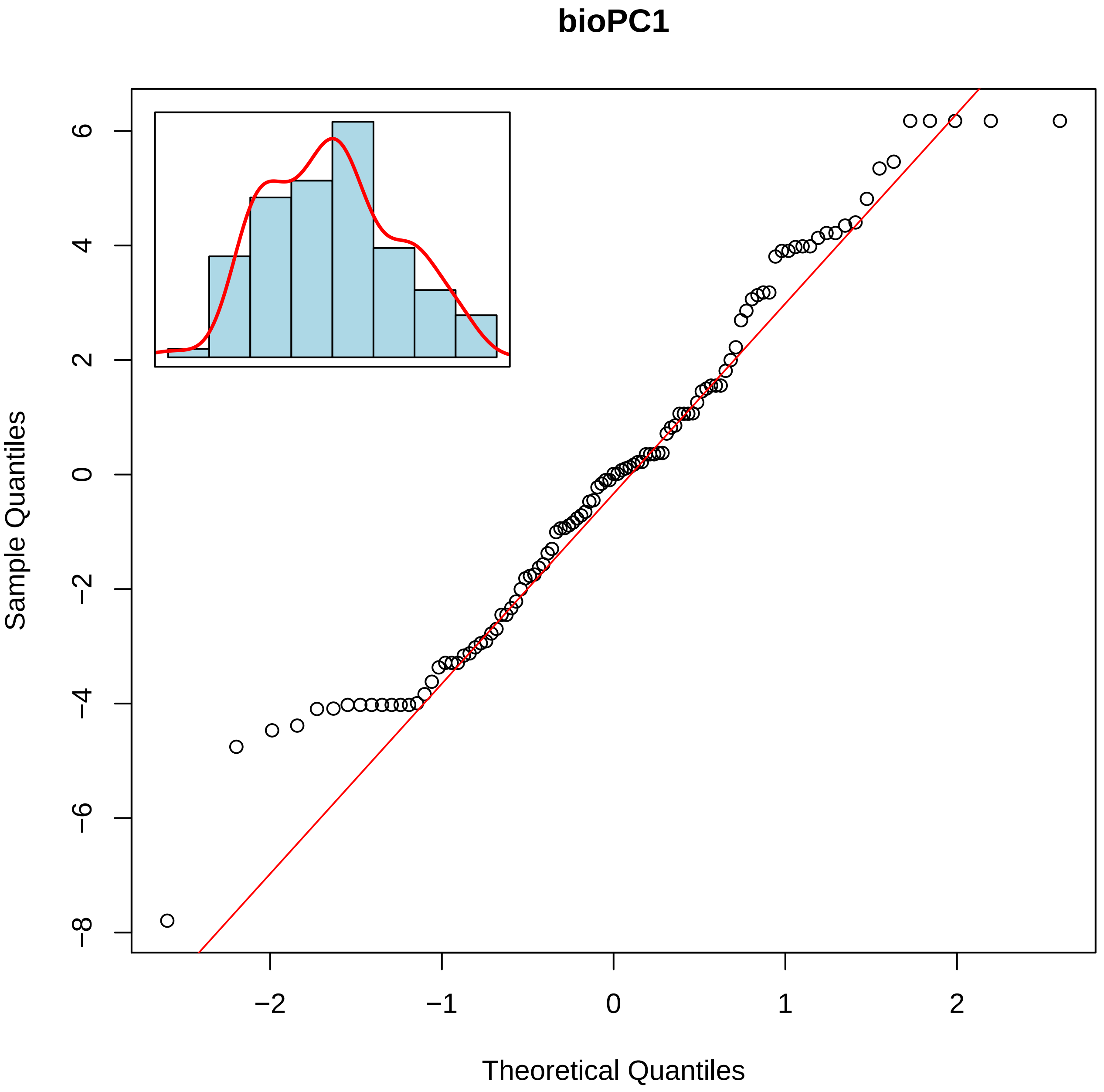
bioPC1
Sample Quantiles
Theoretical Quantiles
6
4
2
0
−2
−4
−6
−8
−2
−1
0
1
2

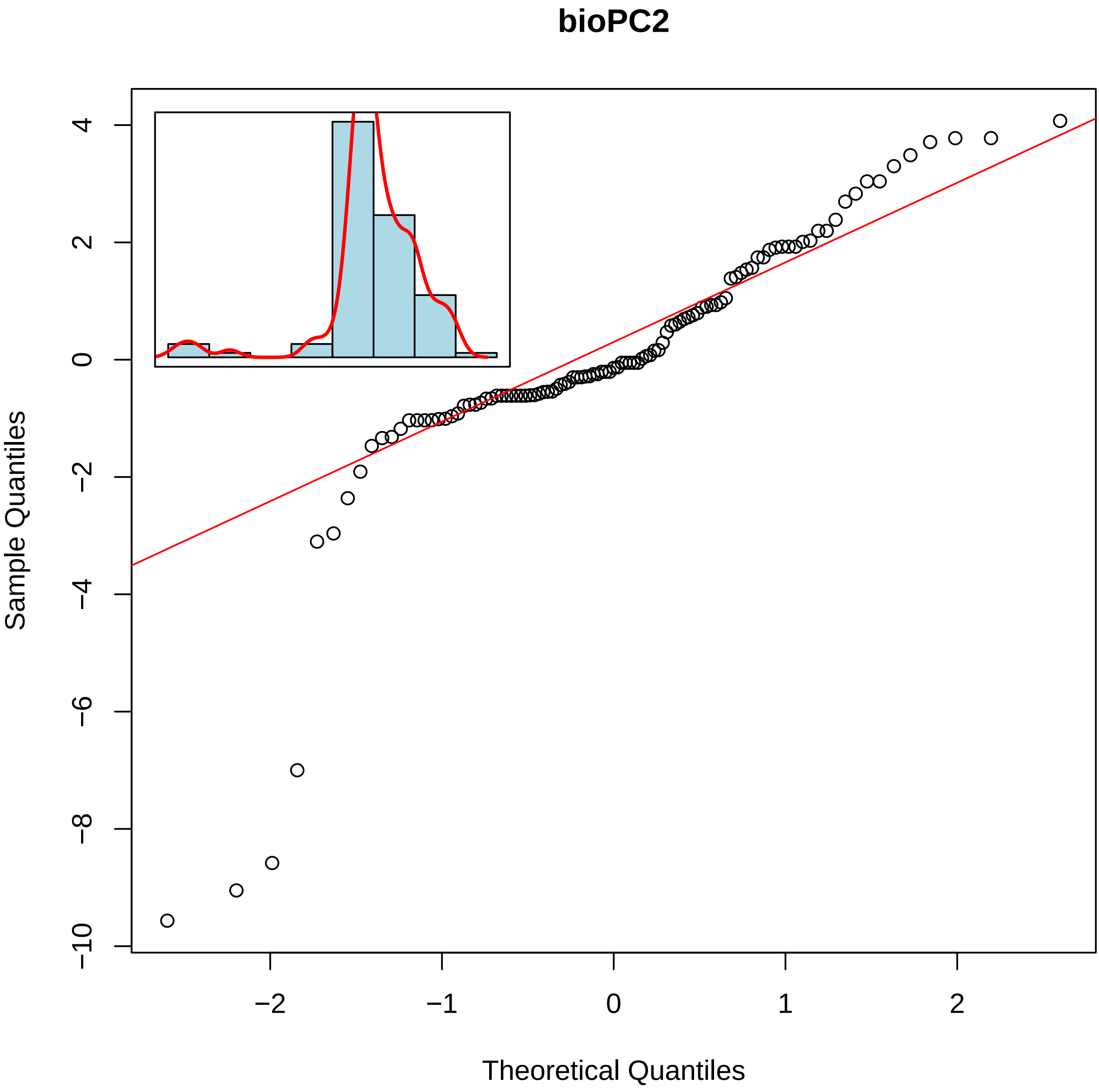

bioPC2
Sample Quantiles
Theoretical Quantiles
4
2
0
−2
−4
−6
−8
−10
−2
−1
0
1
2

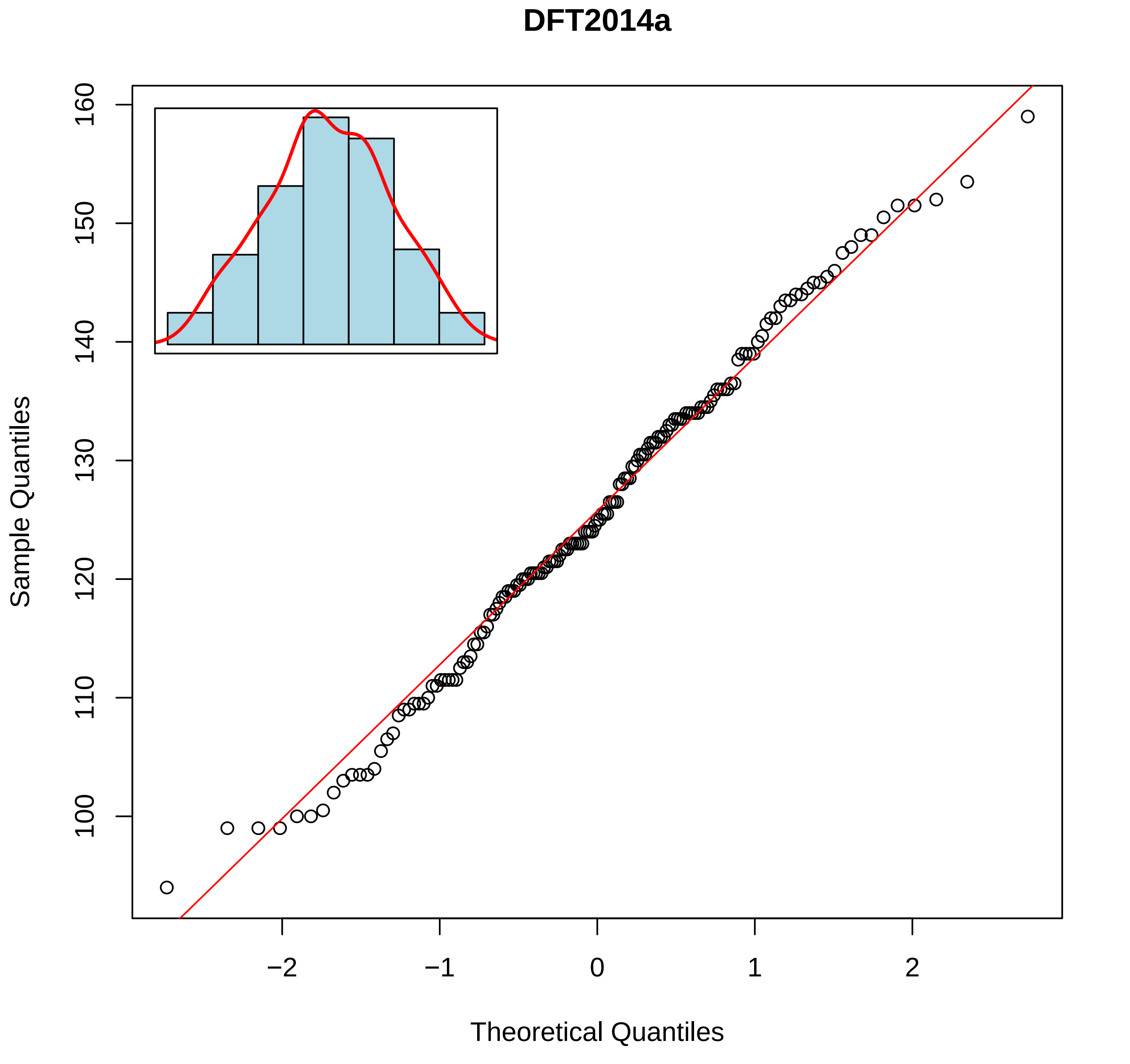

DFT2014a
Sample Quantiles
Theoretical Quantiles
160
150
140
130
120
110
100
−2
−1
0
1
2

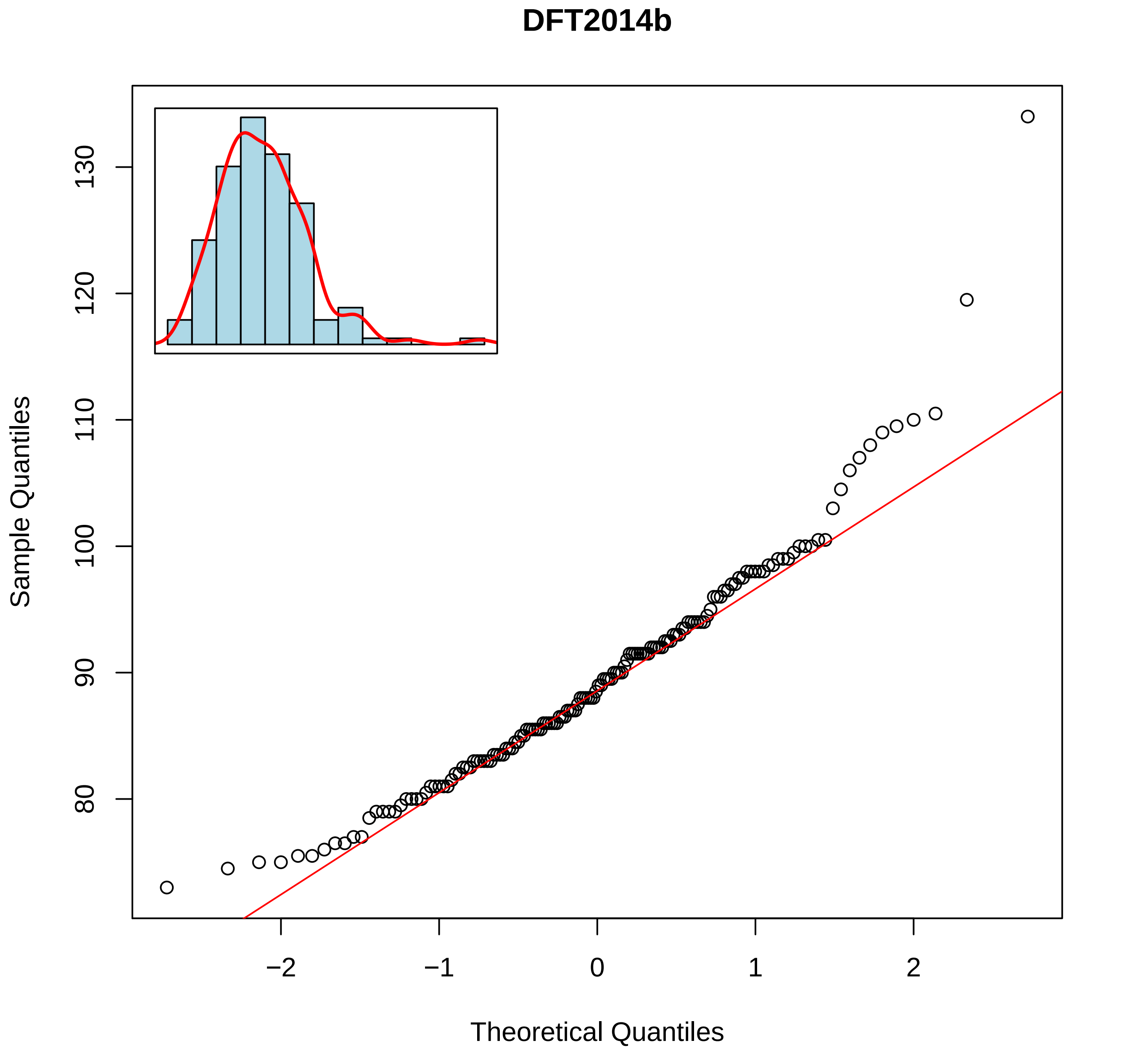
DFT2014b
Sample Quantiles
Theoretical Quantiles
130
120
110
100
90
80
−2
−1
0
1
2

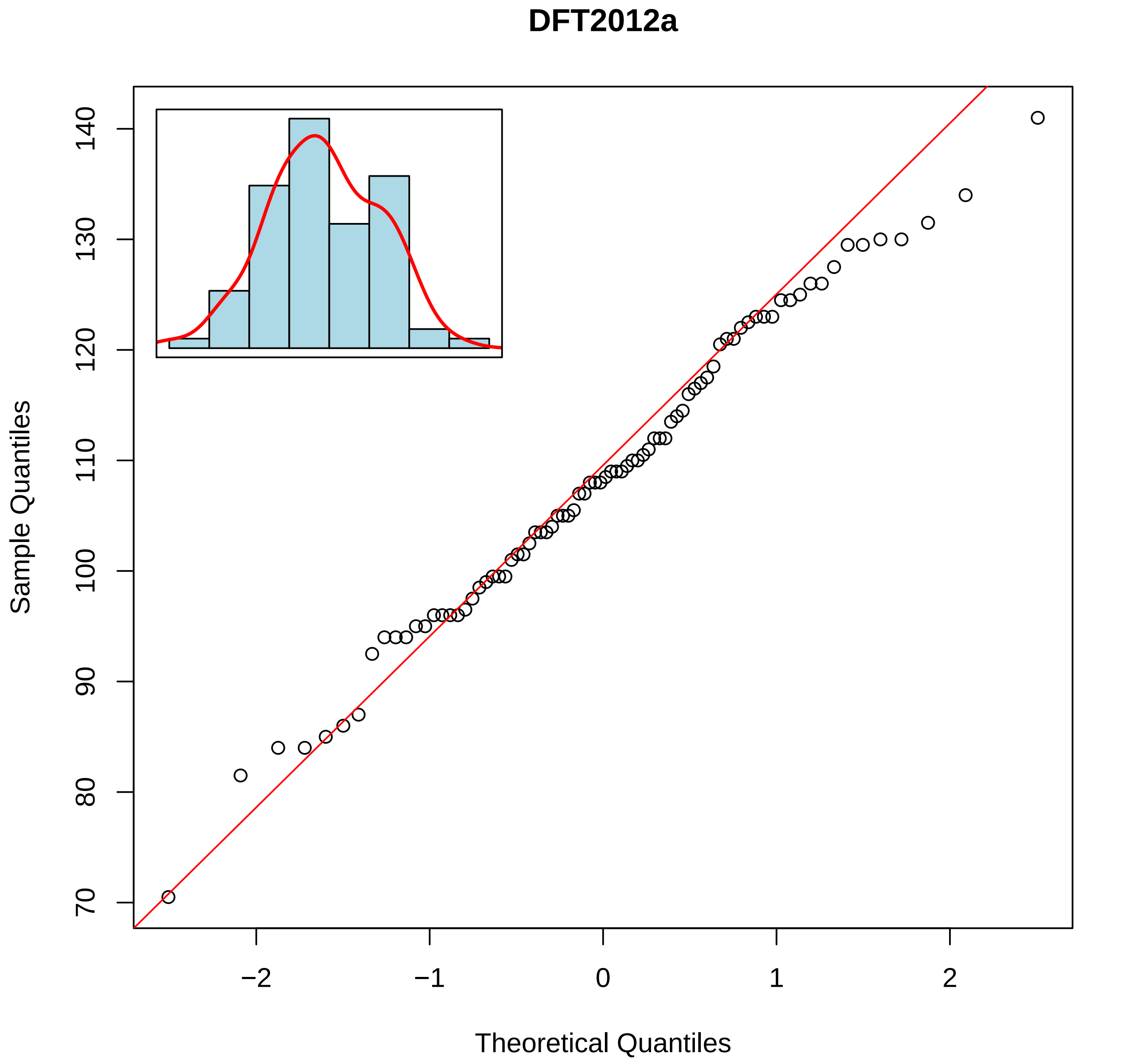

DFT2012a
Sample Quantiles
Theoretical Quantiles
70
80
90
100
110
120
130
140
−2
−1
0
1
2

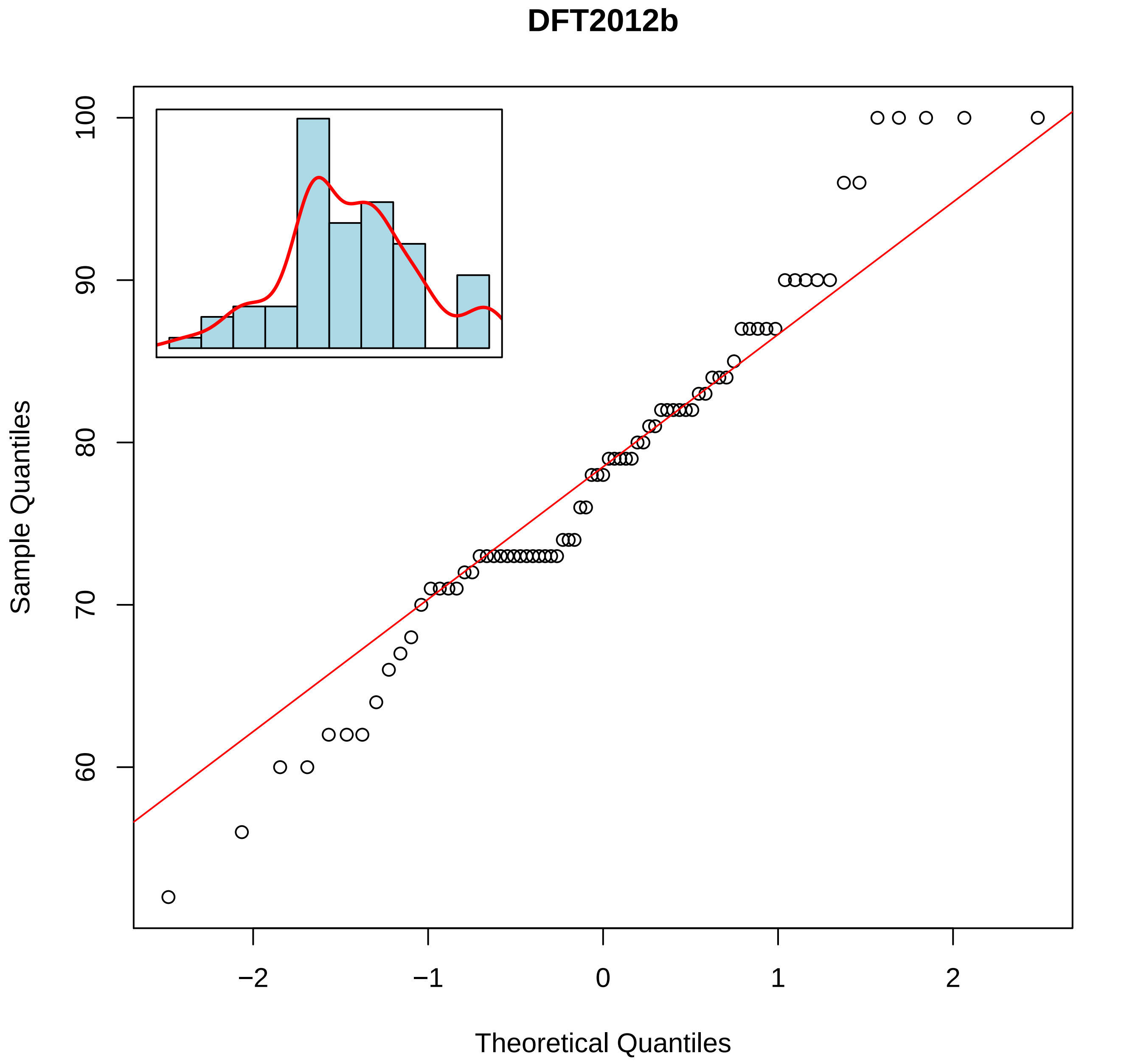
DFT2012b
Sample Quantiles
Theoretical Quantiles
100
90
80
70
60
−2
−1
0
1
2

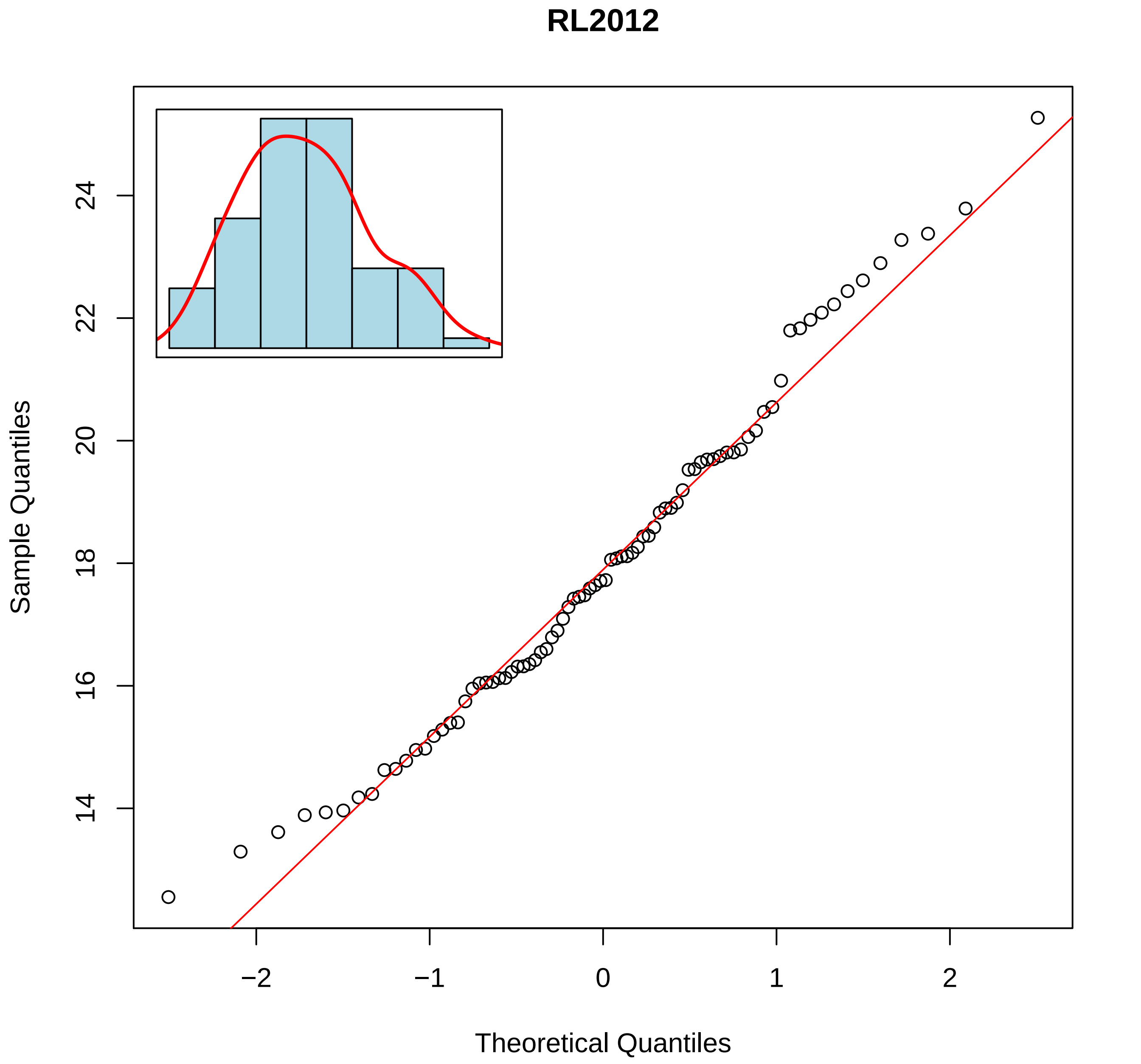
RL2012
Sample Quantiles
Theoretical Quantiles
14
16
18
20
22
24
−2
−1
0
1
2

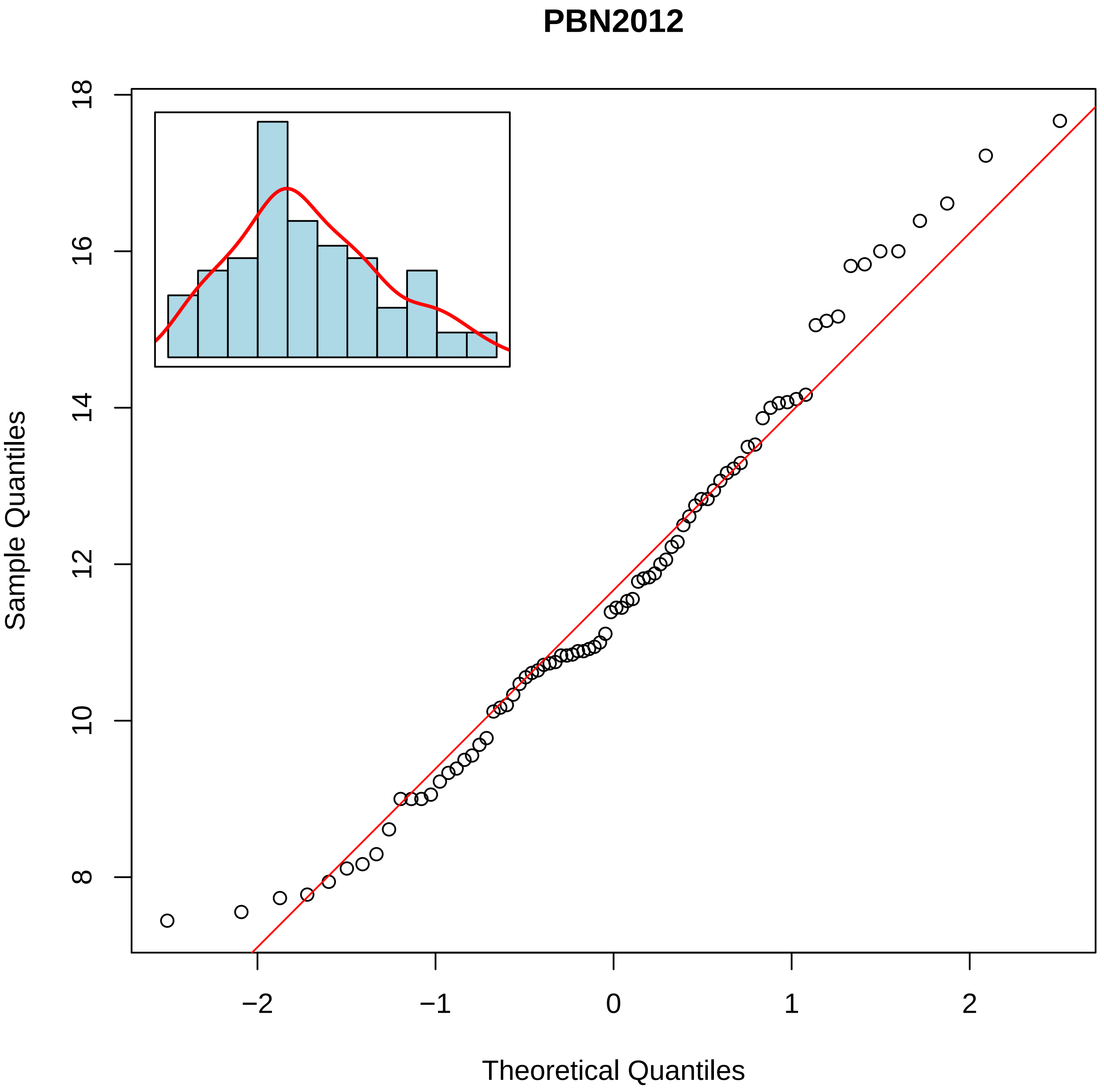
PBN2012
Sample Quantiles
Theoretical Quantiles
8
10
12
14
16
18
−2
−1
0
1
2

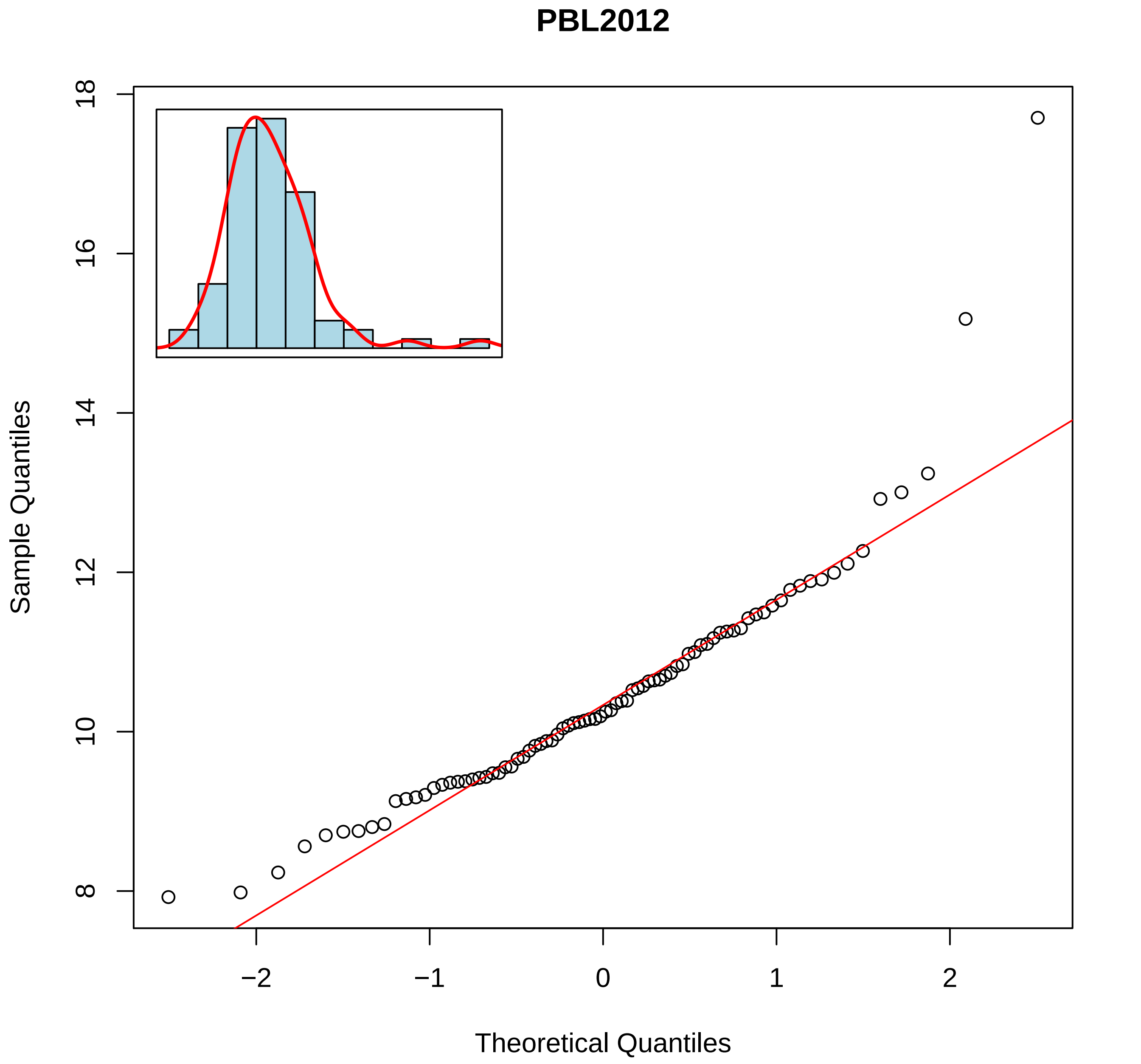
PBL2012
Sample Quantiles
Theoretical Quantiles
18
16
14
12
10
8
−2
−1
0
1
2

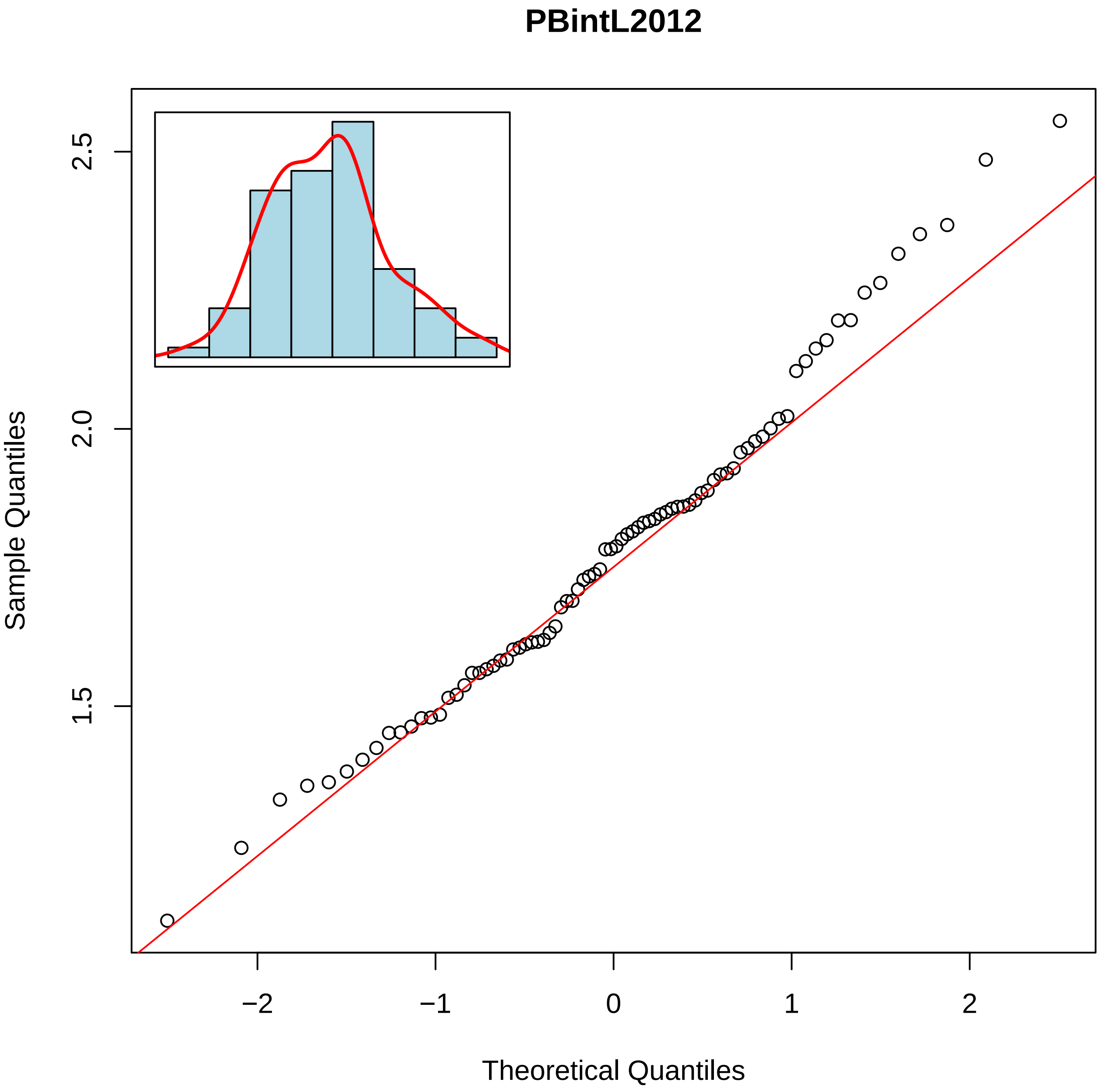
PBintL2012
Sample Quantiles
Theoretical Quantiles
1.5
2.0
2.5
−2
−1
0
1
2

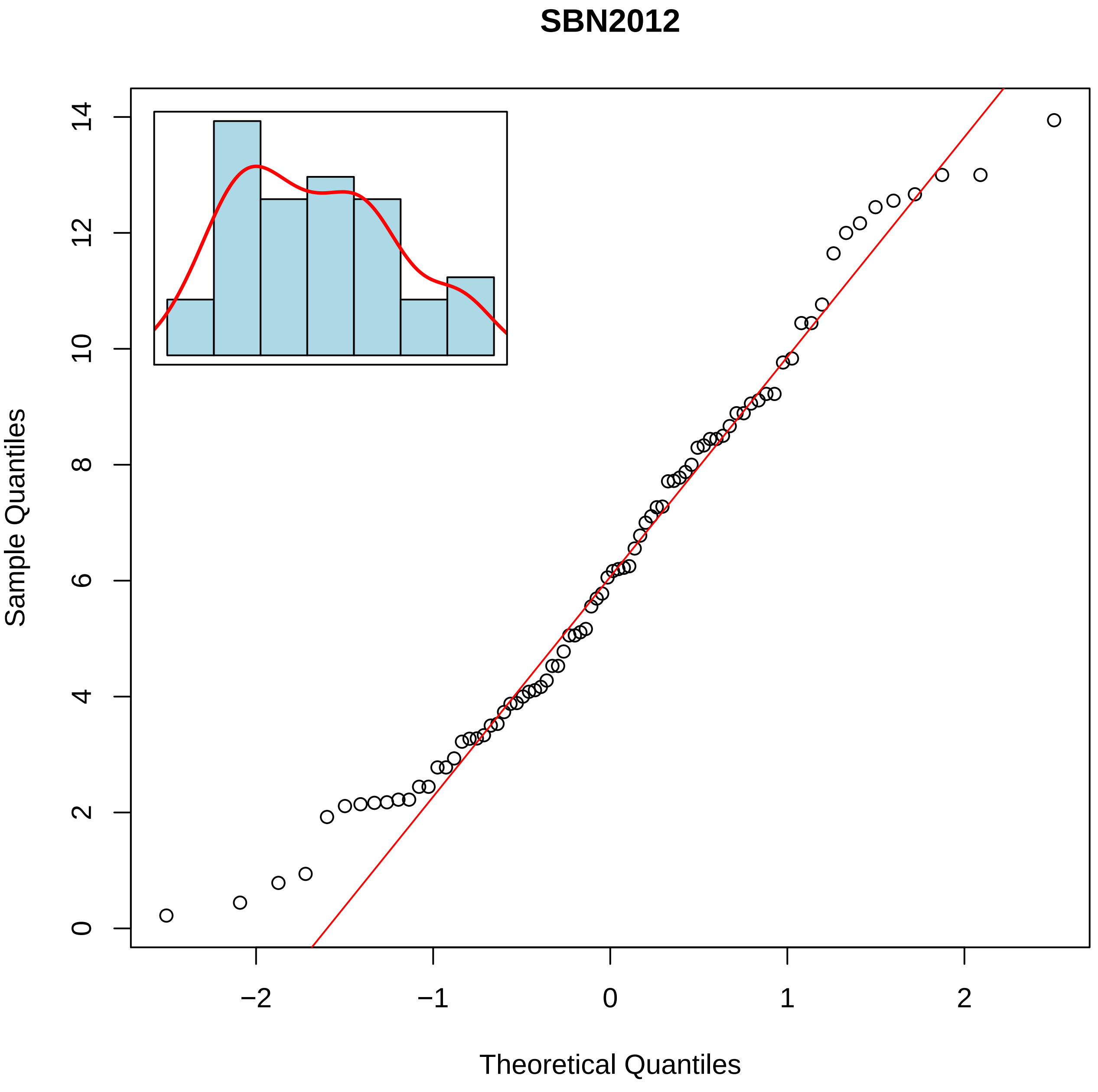
SBN2012
Sample Quantiles
Theoretical Quantiles
0
2
4
6
8
10
12
14
−2
−1
0
1
2

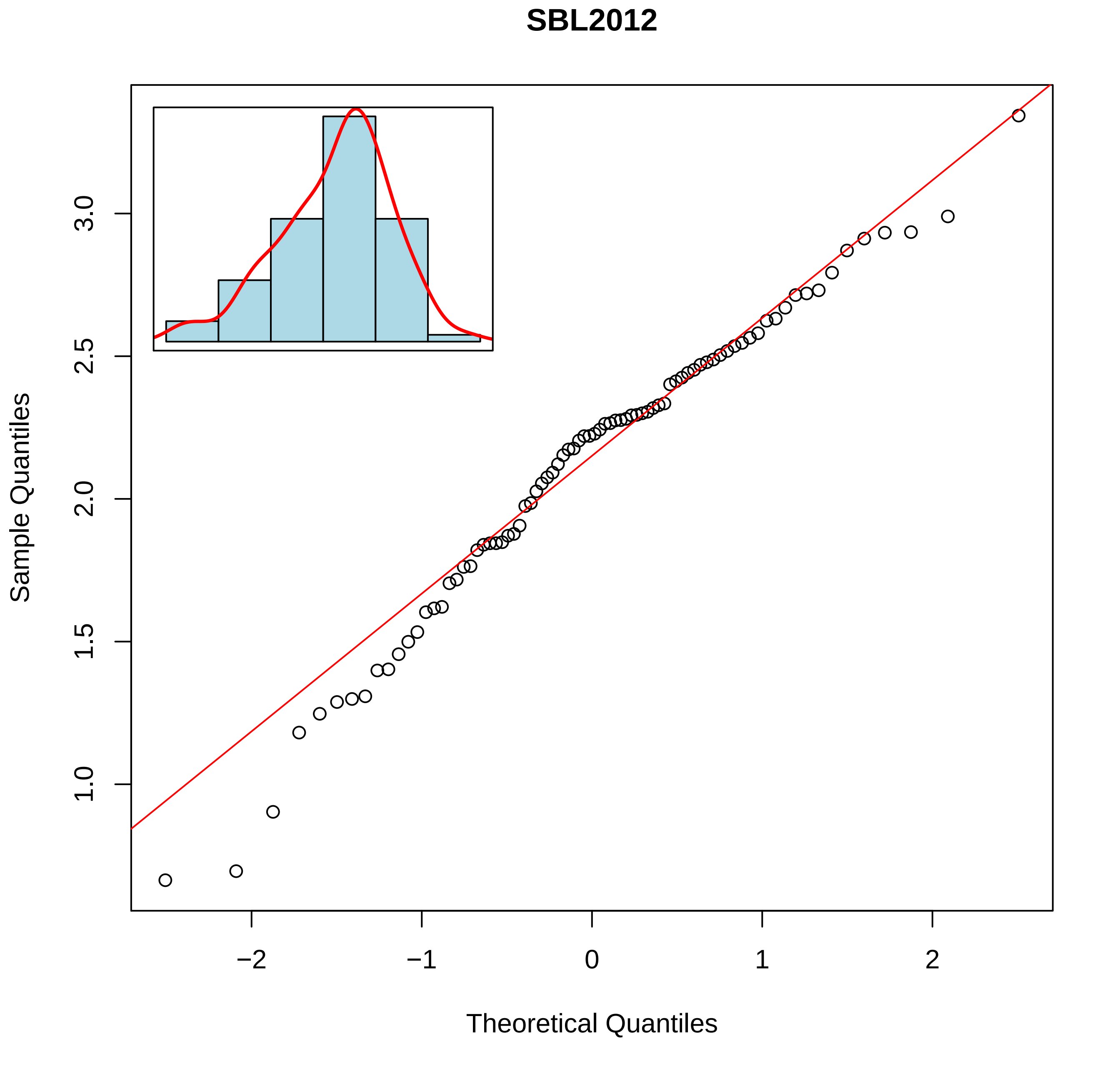
SBL2012
Sample Quantiles
Theoretical Quantiles
1.0
1.5
2.0
2.5
3.0
−2
−1
0
1
2

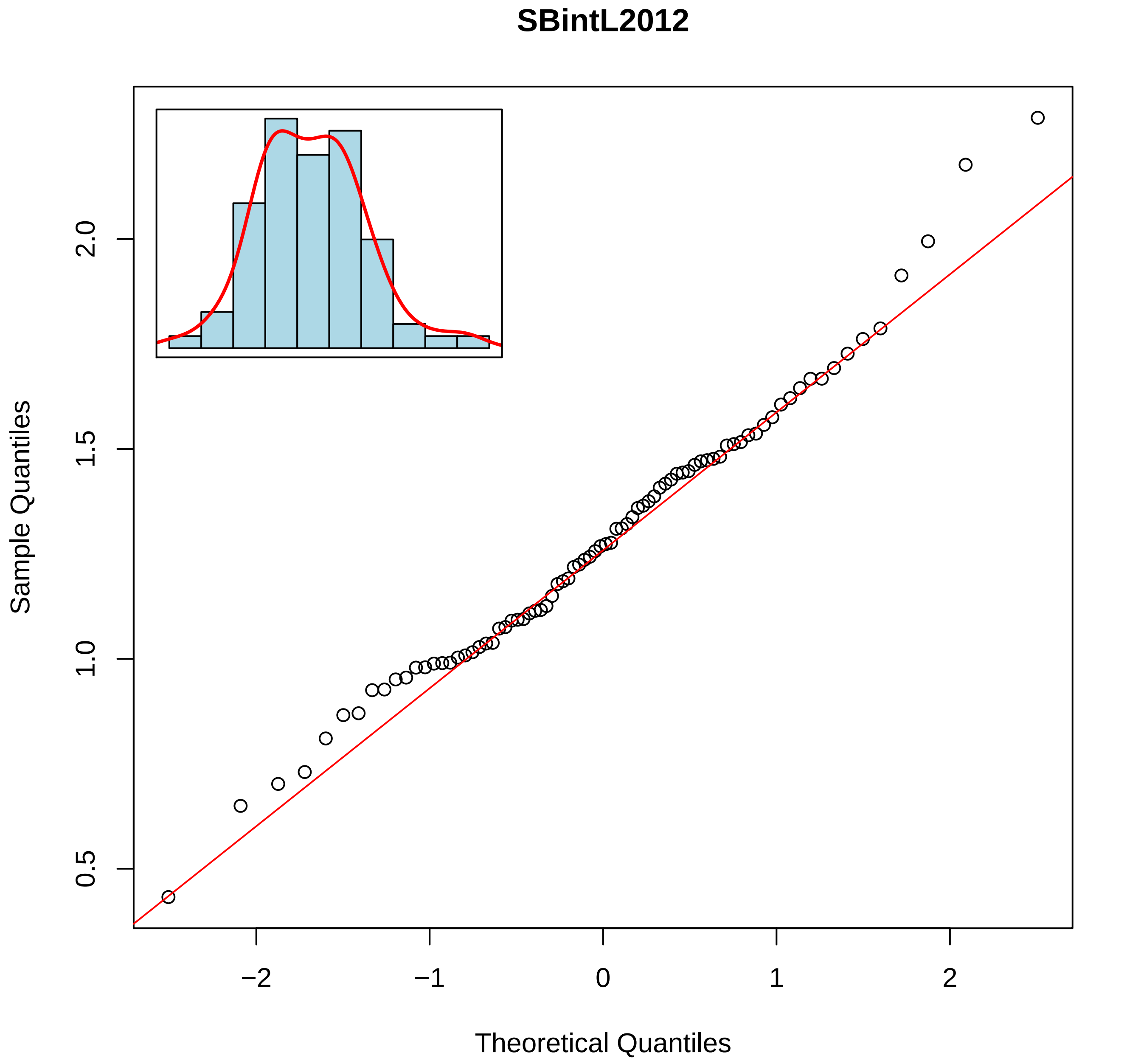

SBintL2012
Sample Quantiles
Theoretical Quantiles
0.5
1.0
1.5
2.0
−2
−1
0
1
2

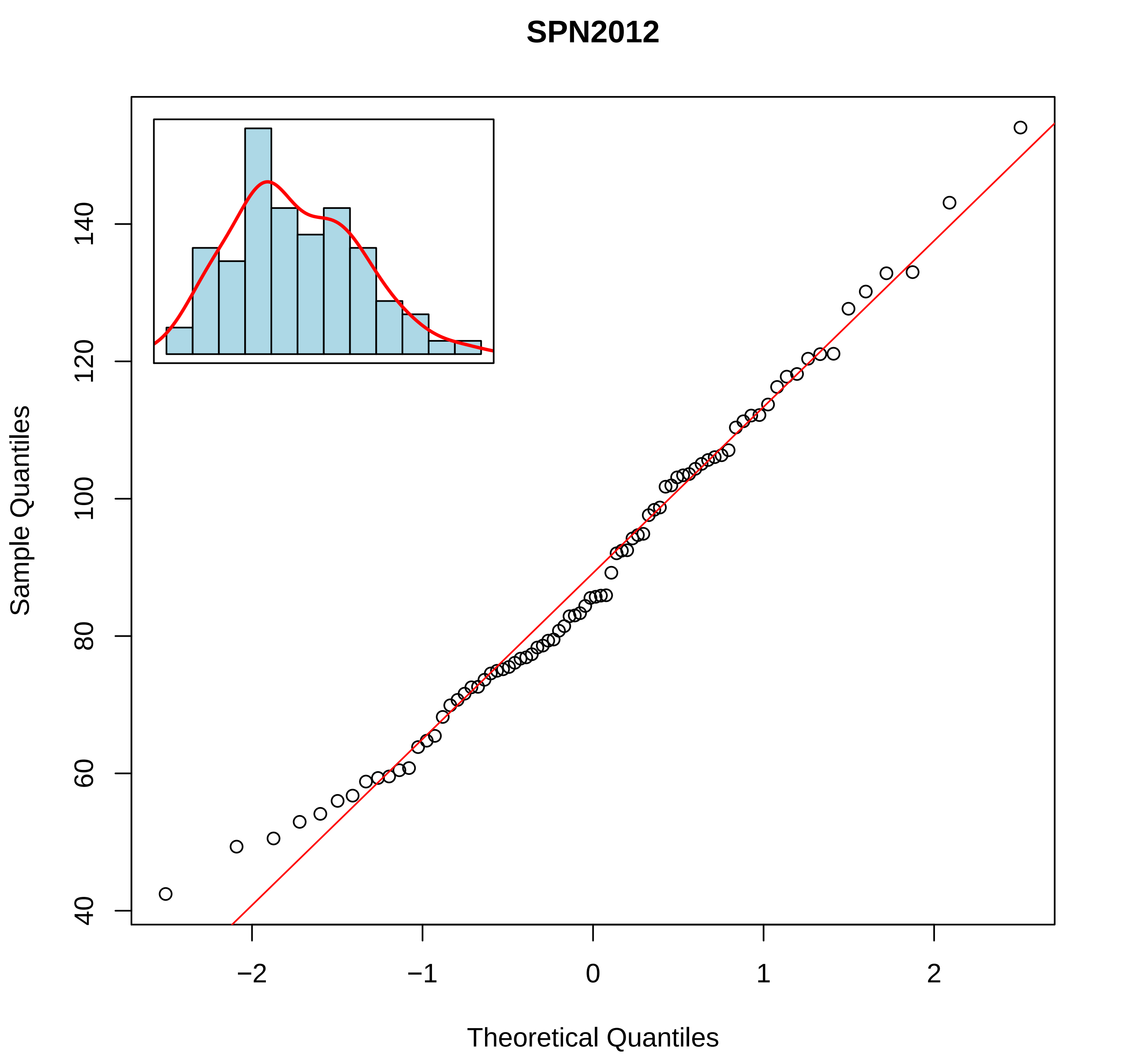

SPN2012
Sample Quantiles
Theoretical Quantiles
40
60
80
100
120
140
−2
−1
0
1
2

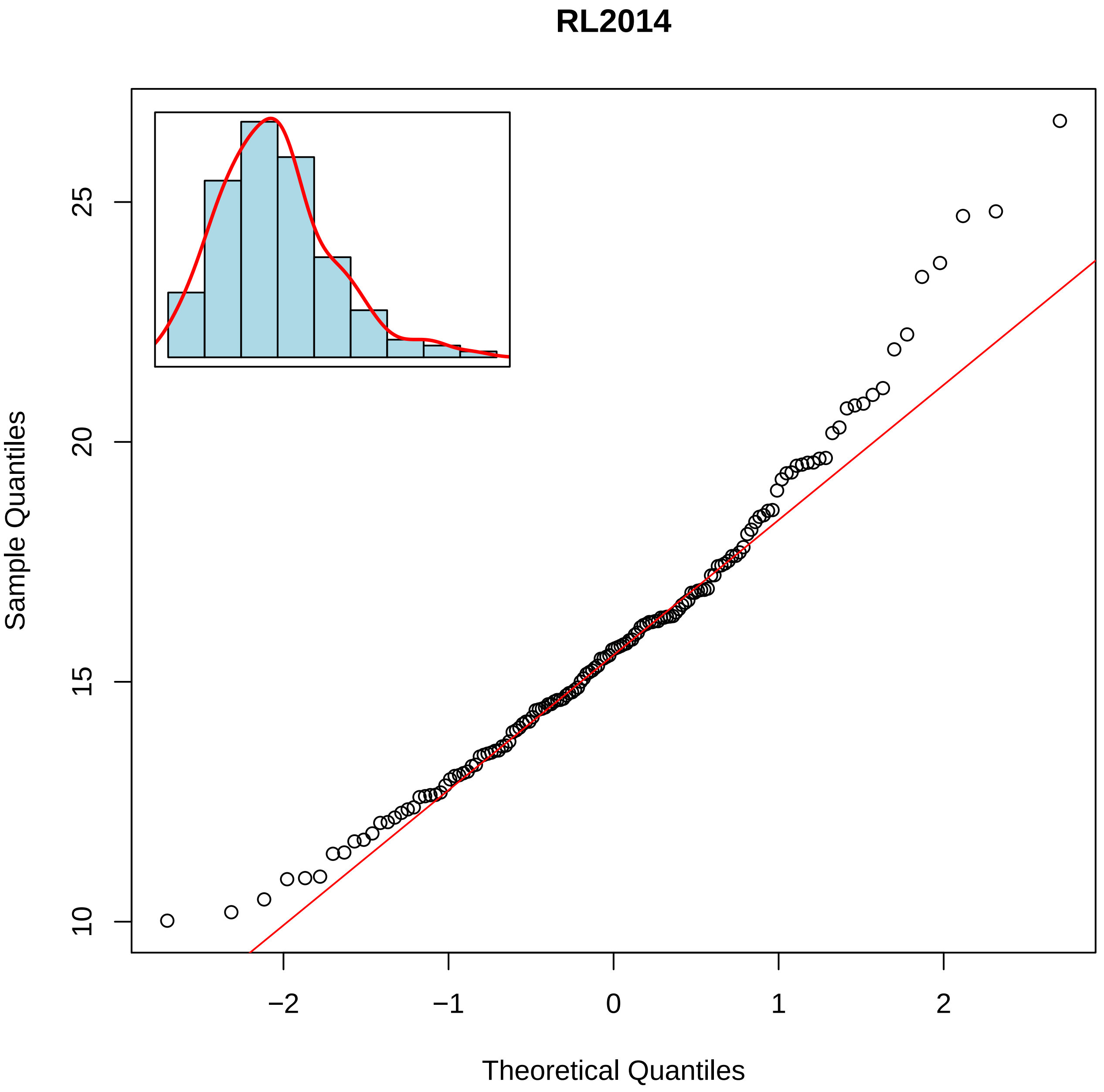
RL2014
Sample Quantiles
Theoretical Quantiles
10
15
20
25
−2
−1
0
1
2

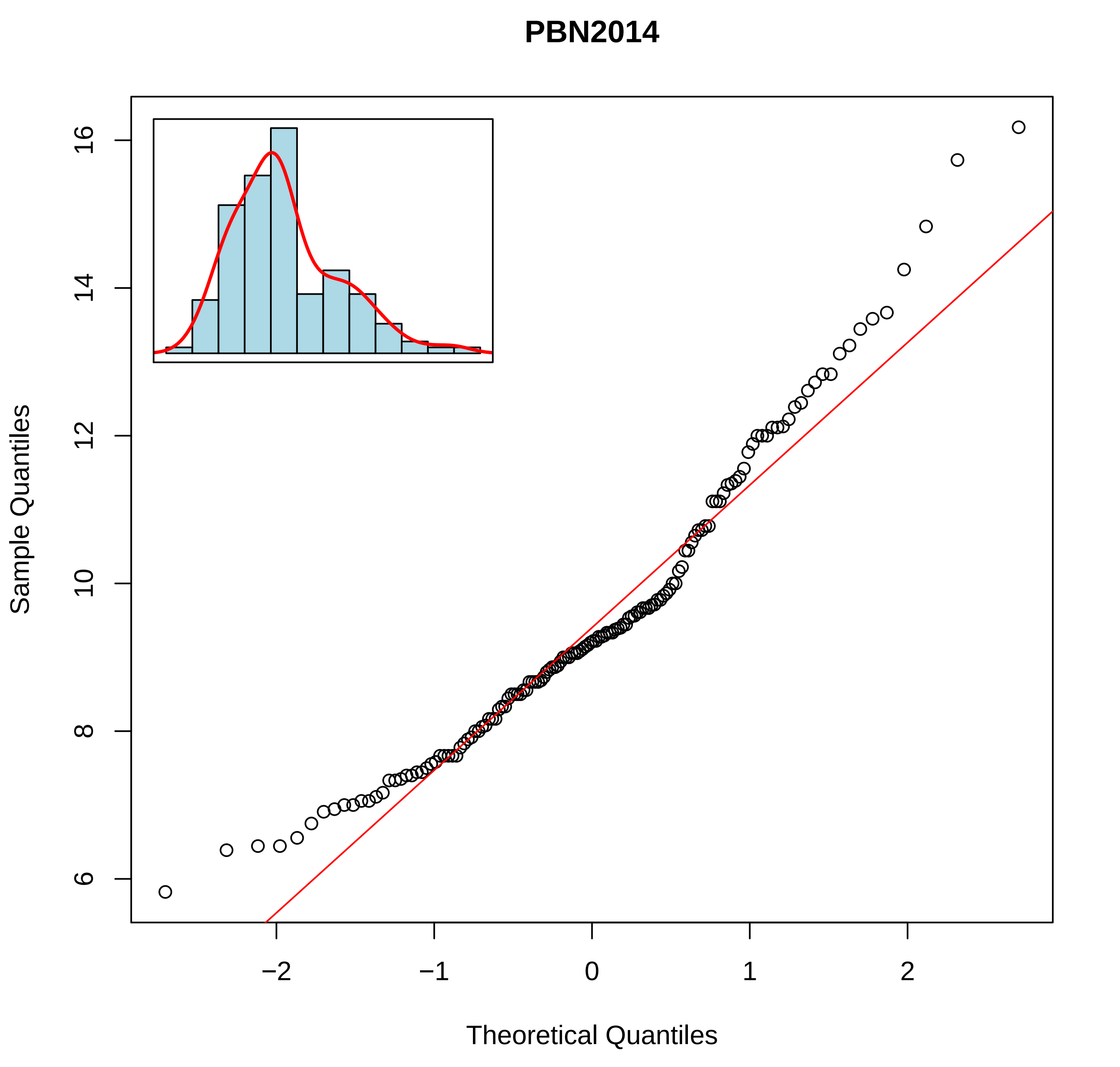

PBN2014
Sample Quantiles
Theoretical Quantiles
6
8
10
12
14
16
−2
−1
0
1
2

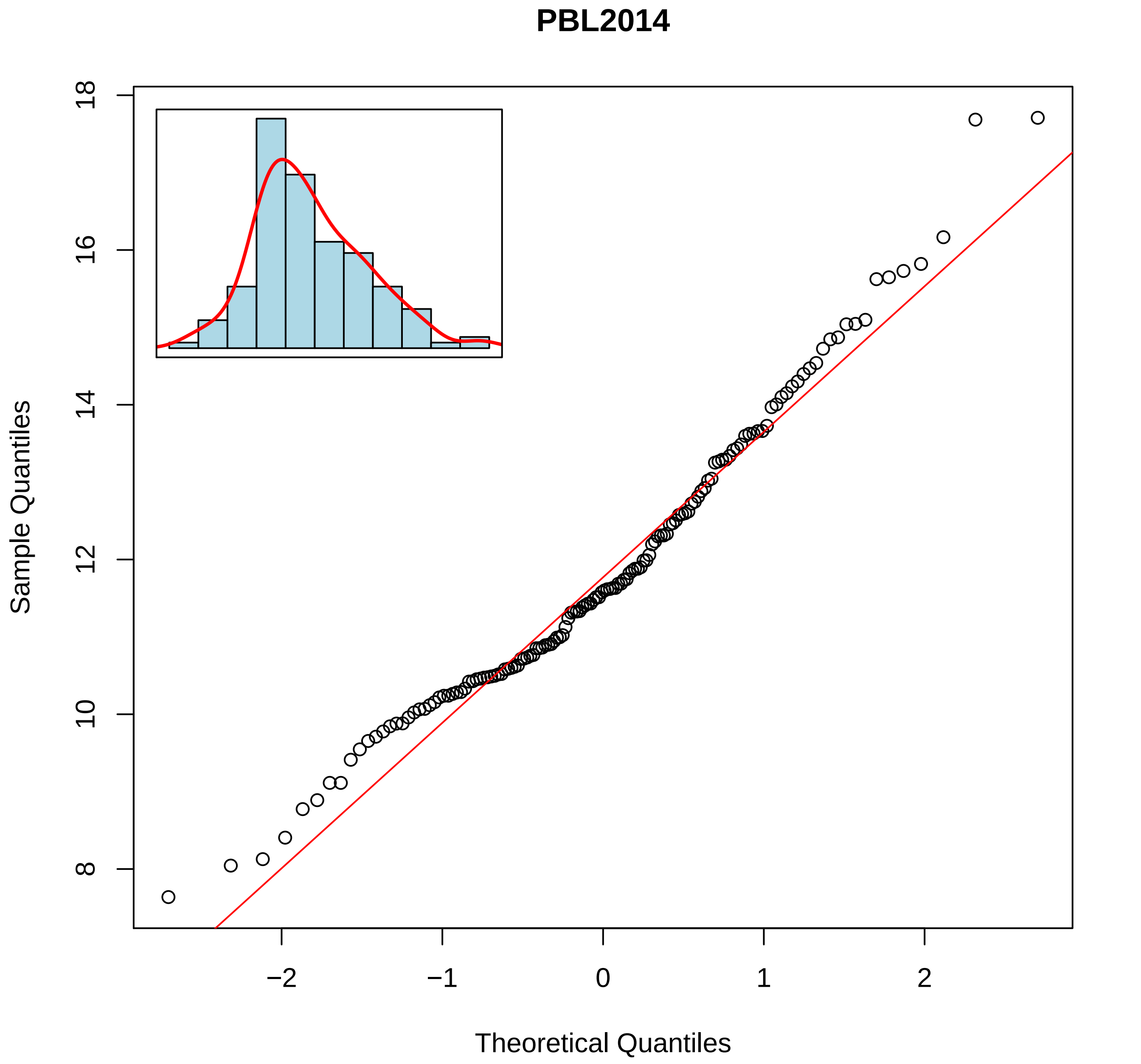

PBL2014
Sample Quantiles
Theoretical Quantiles
8
10
12
14
16
18
−2
−1
0
1
2

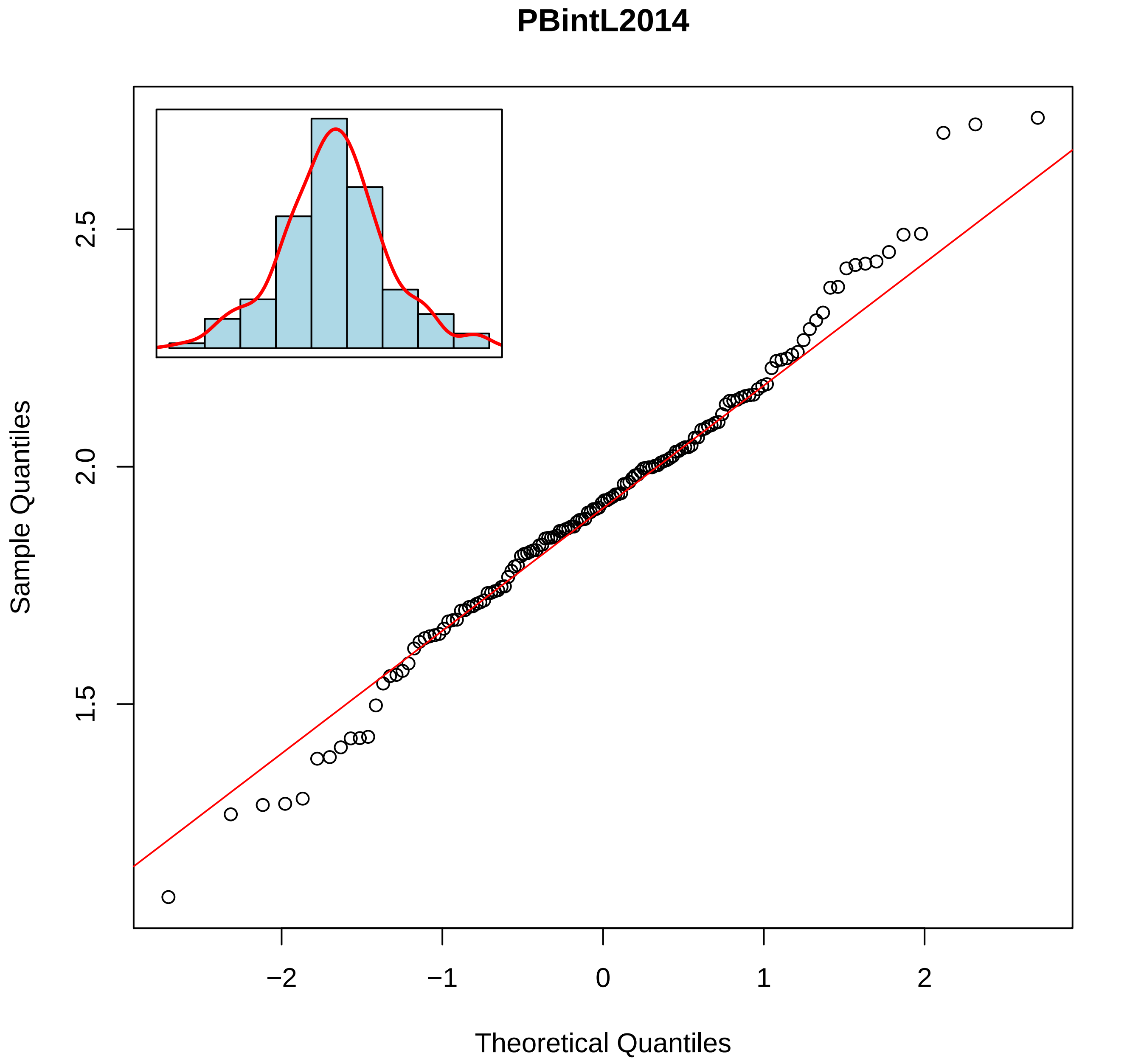

PBintL2014
Sample Quantiles
Theoretical Quantiles
2.5
2.0
1.5
−2
−1
0
1
2

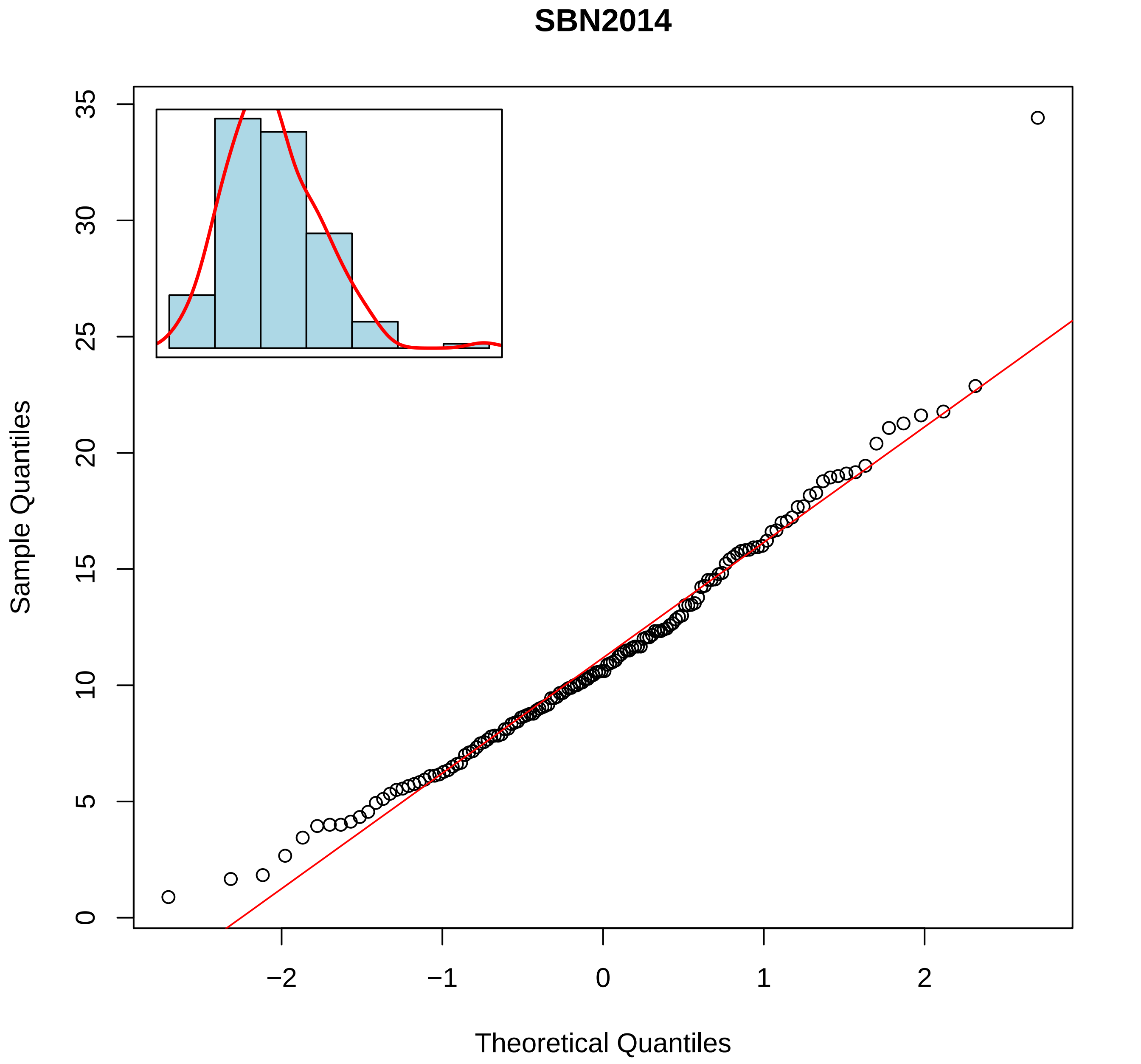
SBN2014
Sample Quantiles
Theoretical Quantiles
0
5
10
15
20
25
30
35
−2
−1
0
1
2

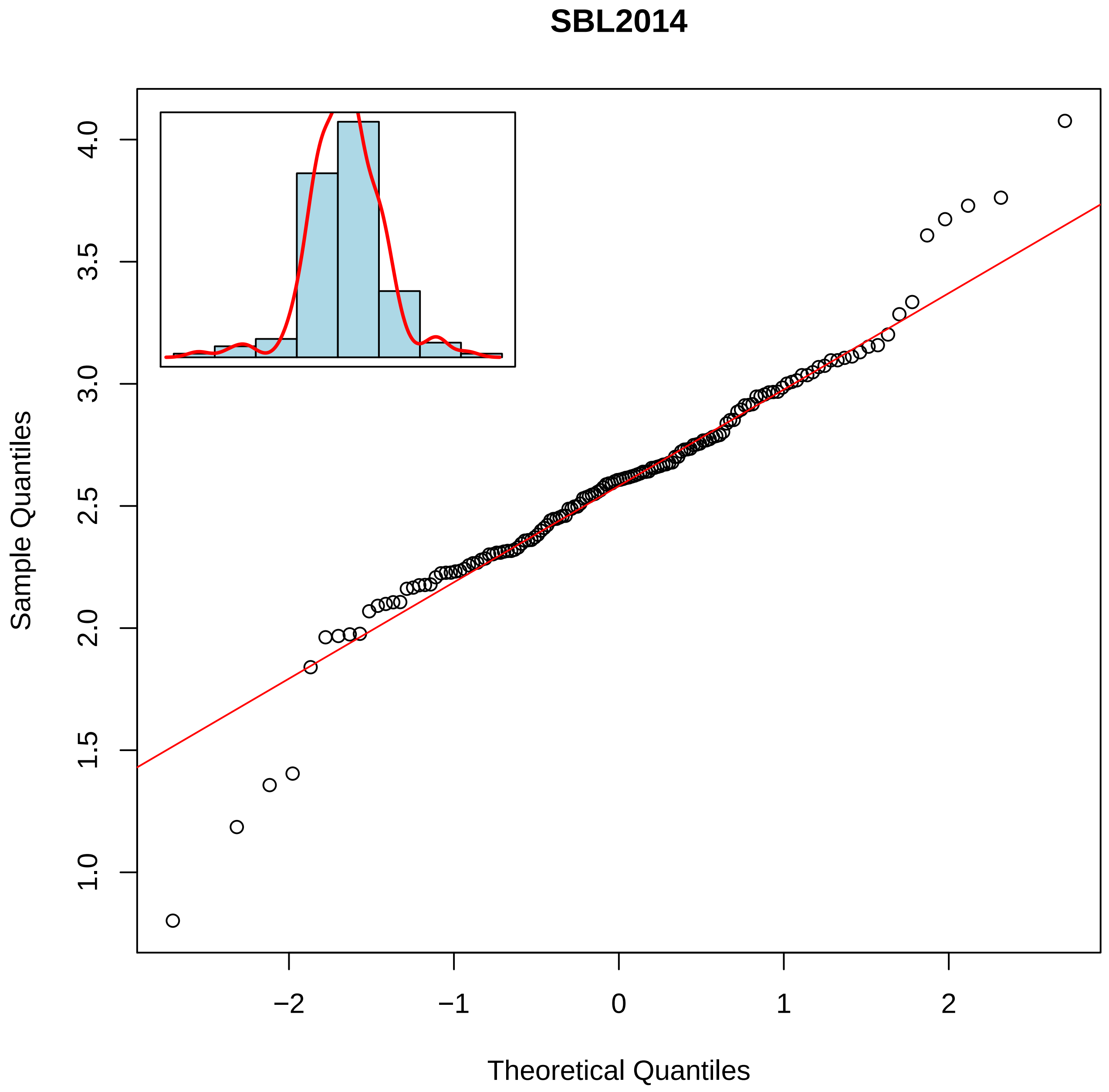
SBL2014
Sample Quantiles
1.0
1.5
2.0
2.5
3.0
3.5
4.0
−2
−1
0
1
2
Theoretical Quantiles

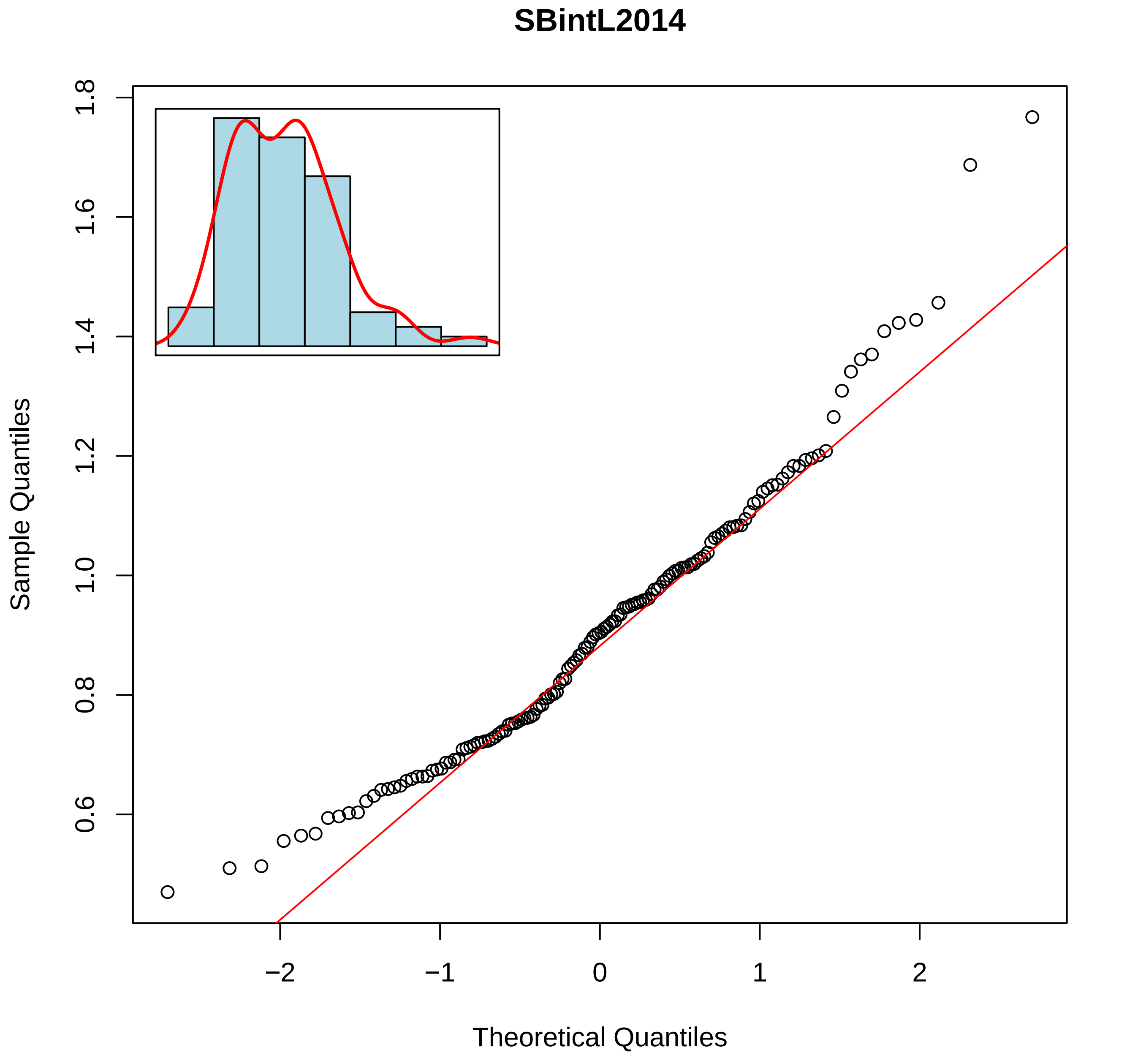
SBintL2014
Sample Quantiles
Theoretical Quantiles
0.6
0.8
1.0
1.2
1.4
1.6
1.8
−2
−1
0
1
2

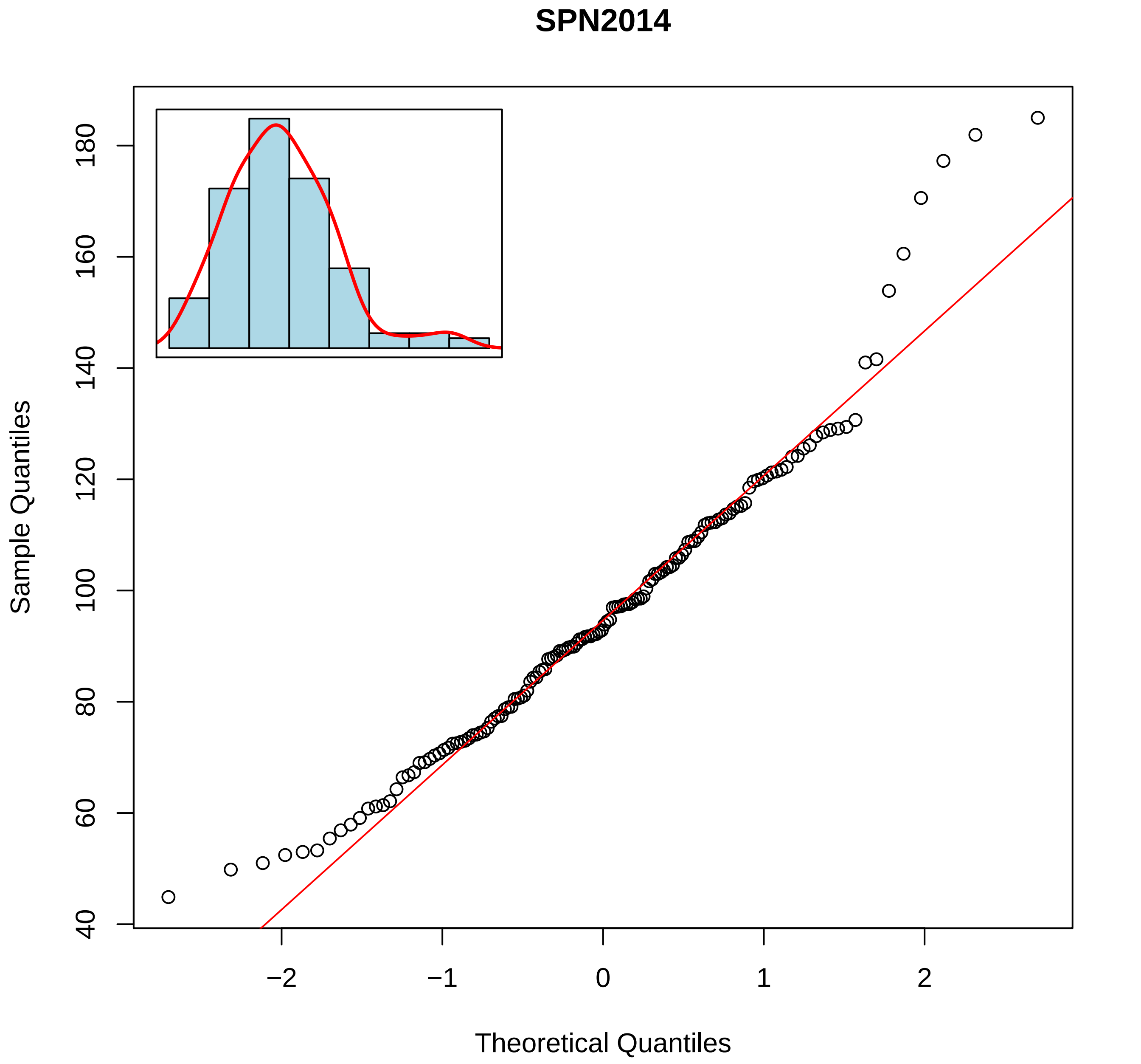

SPN2014
Sample Quantiles
Theoretical Quantiles
40
60
80
100
120
140
160
180
−2
−1
0
1
2

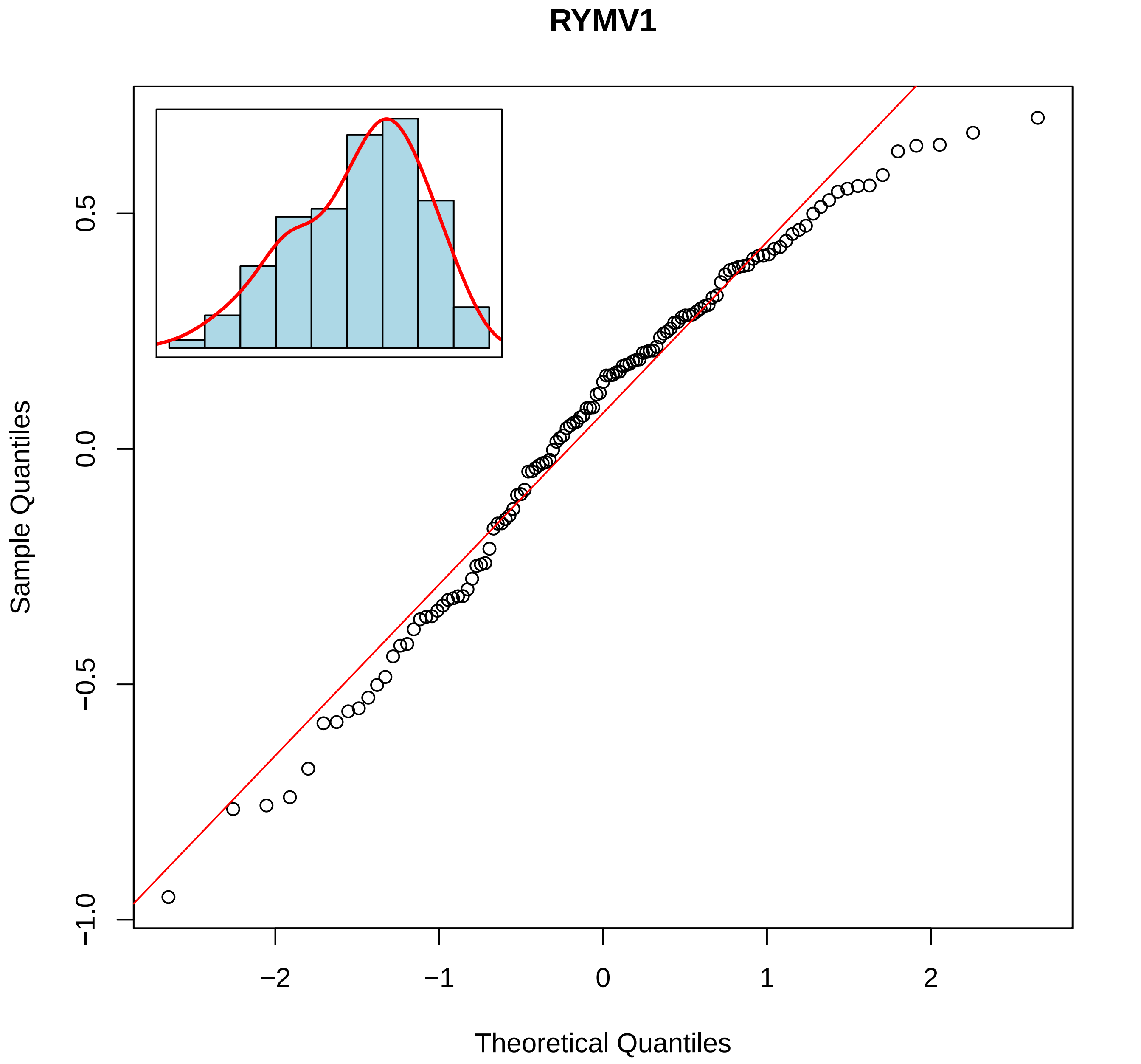
RYMV1
Sample Quantiles
Theoretical Quantiles
0.5
0.0
−0.5
−1.0
−2
−1
0
1
2

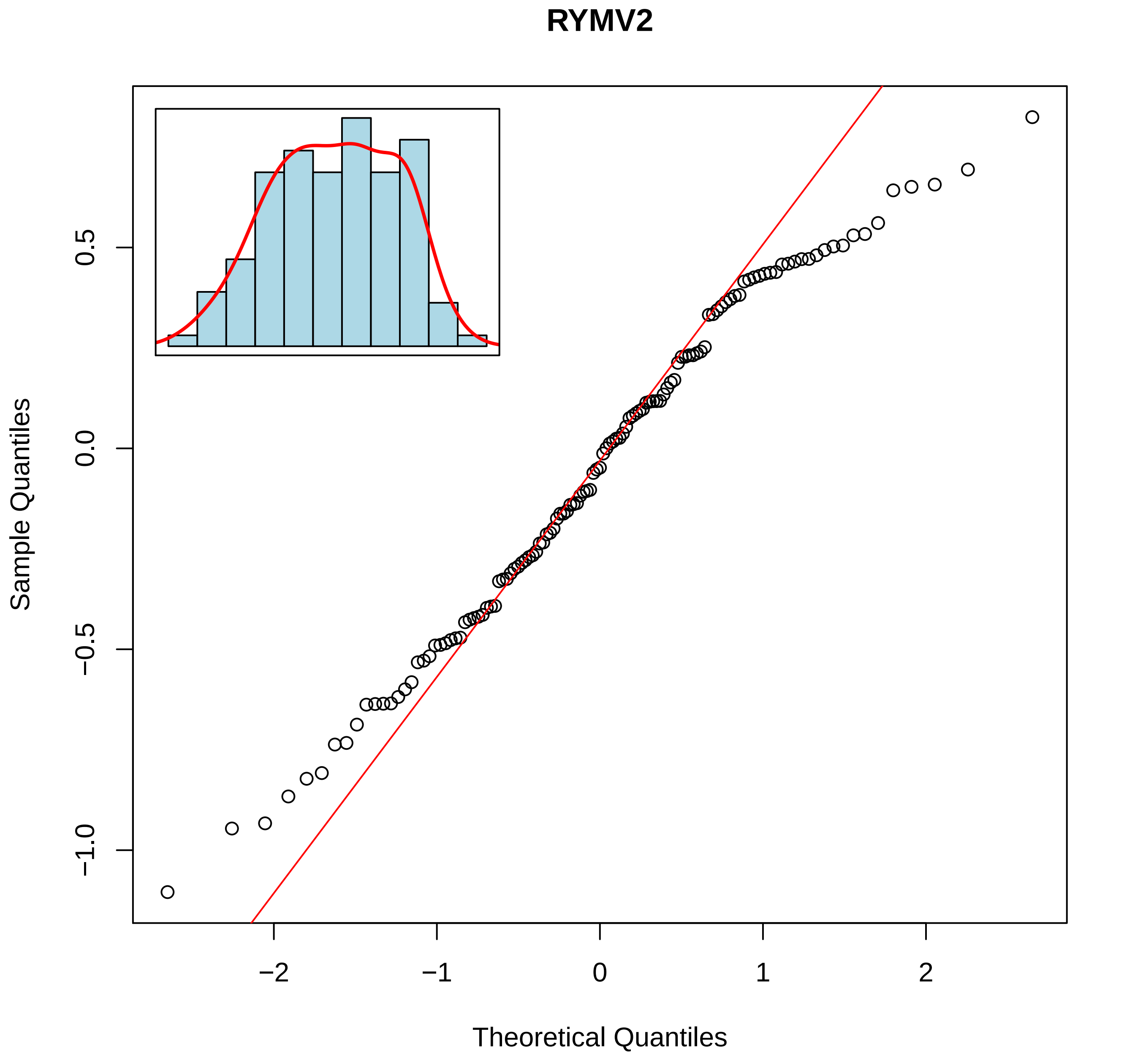
RYMV2
Sample Quantiles
Theoretical Quantiles
−1.0
−0.5
0.0
0.5
−2
−1
0
1
2

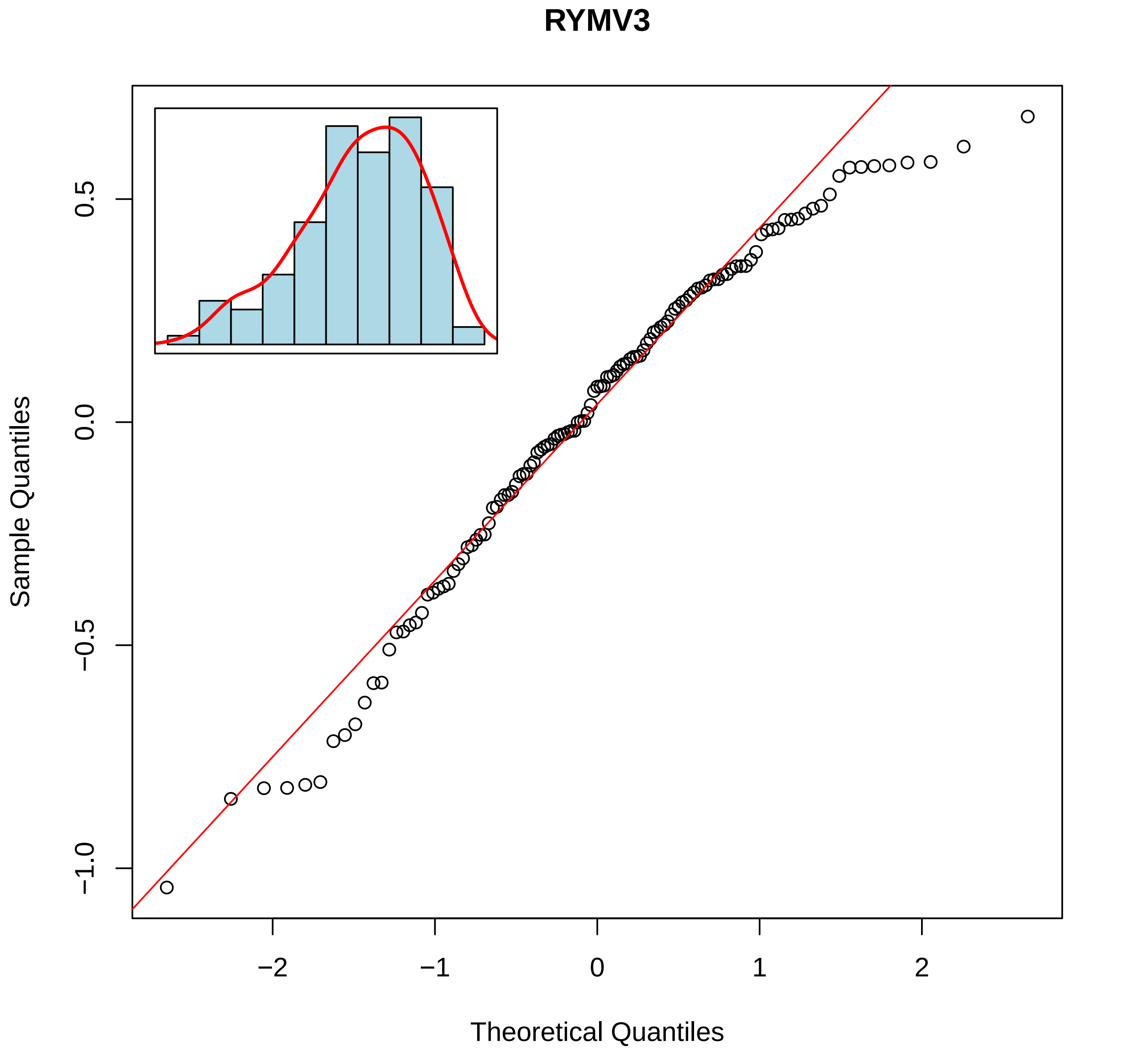
RYMV3
Sample Quantiles
Theoretical Quantiles
0.5
0.0
−0.5
−1.0
−2
−1
0
1
2

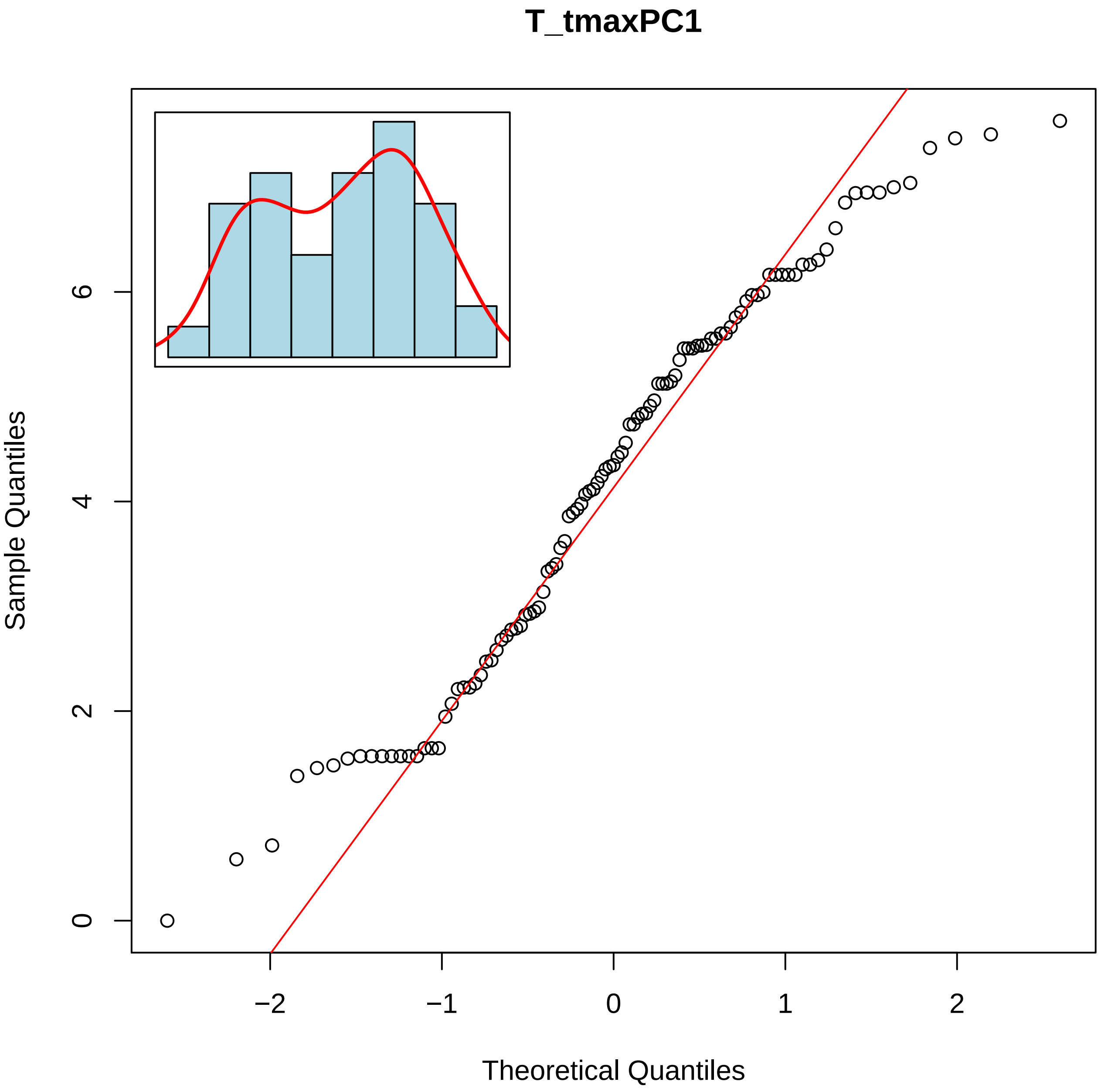
T_tmaxPC1
Sample Quantiles
Theoretical Quantiles
0
2
4
6
−2
−1
0
1
2

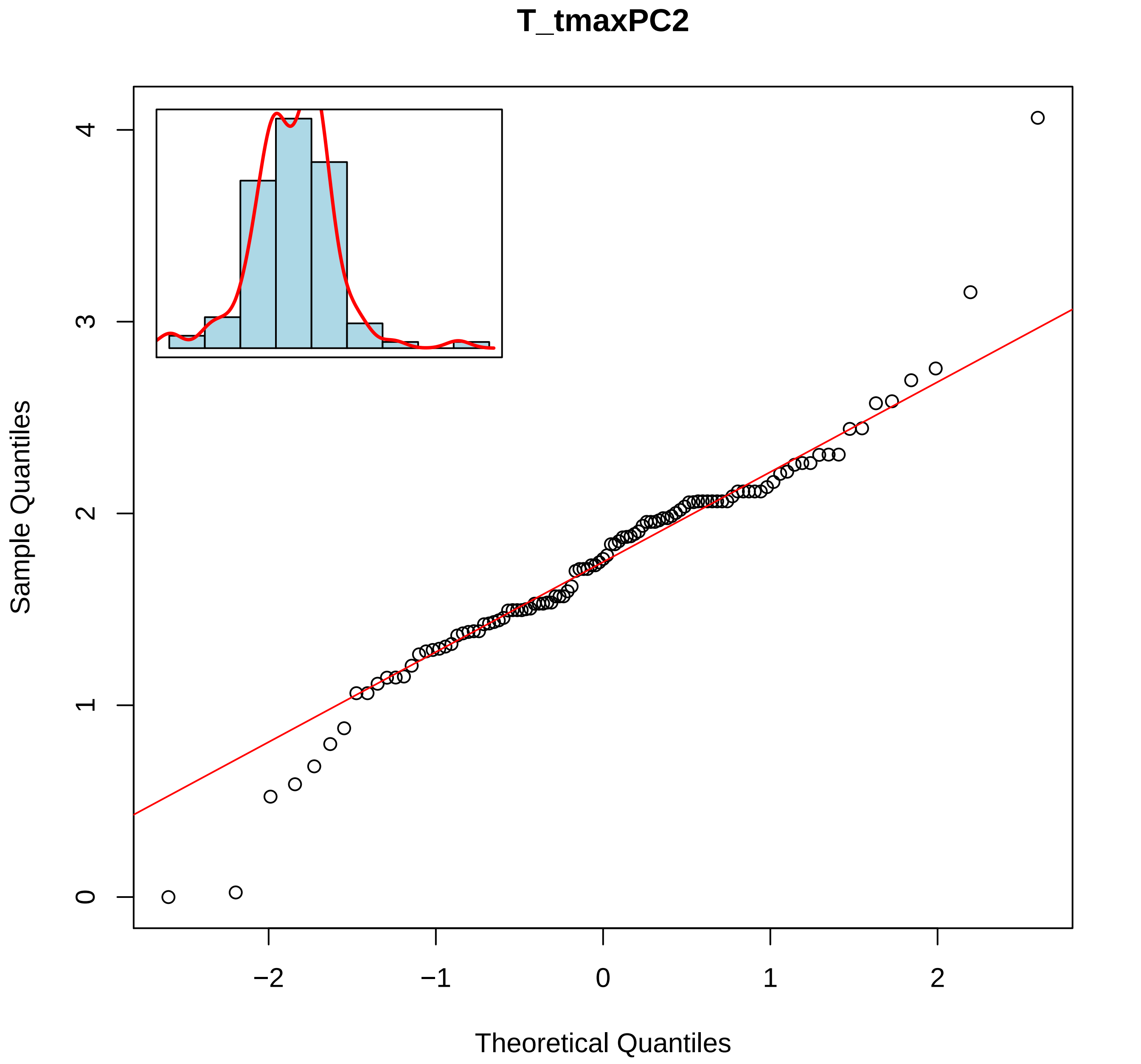
T_tmaxPC2
Sample Quantiles
0
1
2
3
4
Theoretical Quantiles
−2
−1
0
1
2

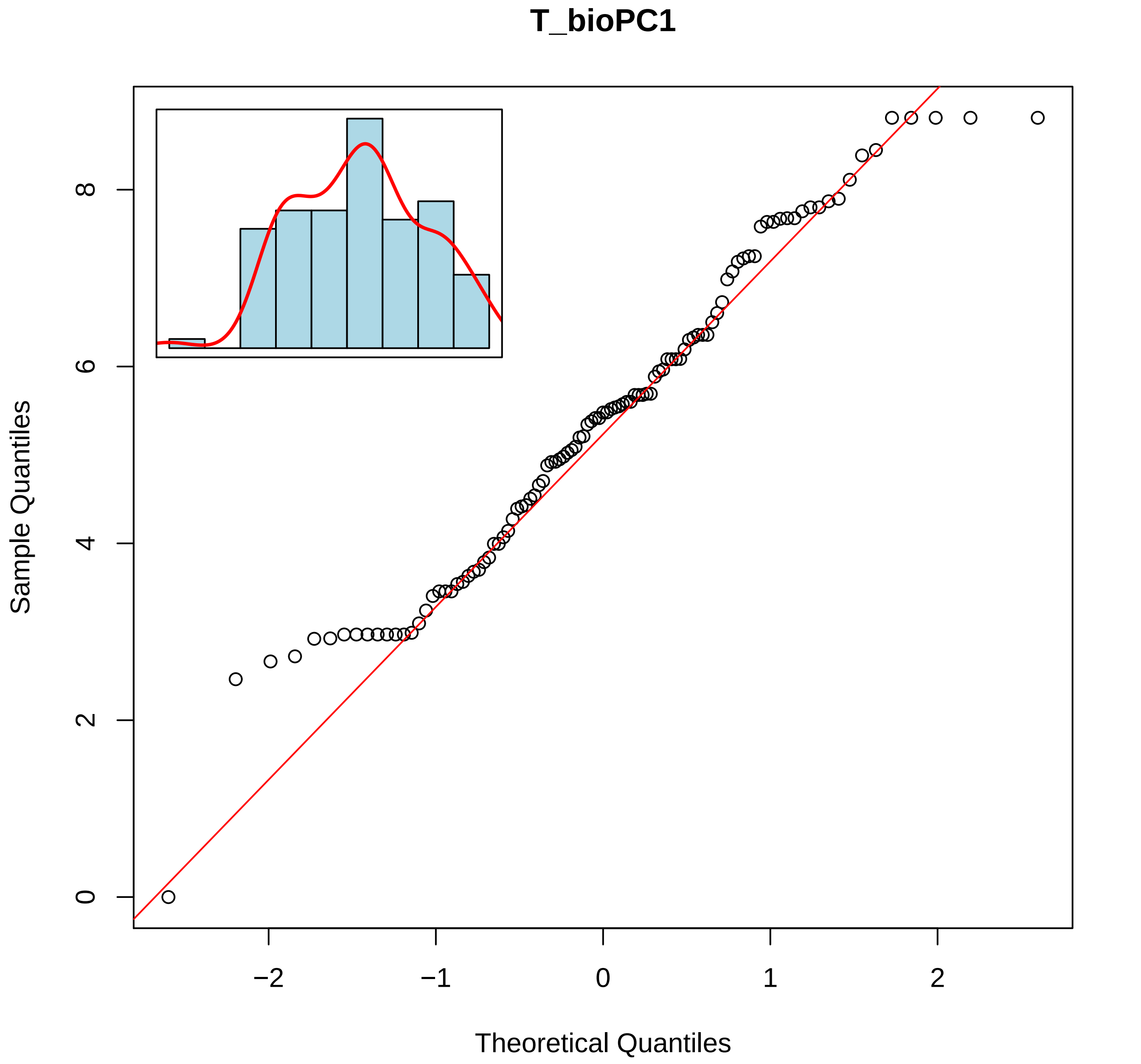
T_bioPC1
Sample Quantiles
Theoretical Quantiles
0
2
4
6
8
−2
−1
0
1
2

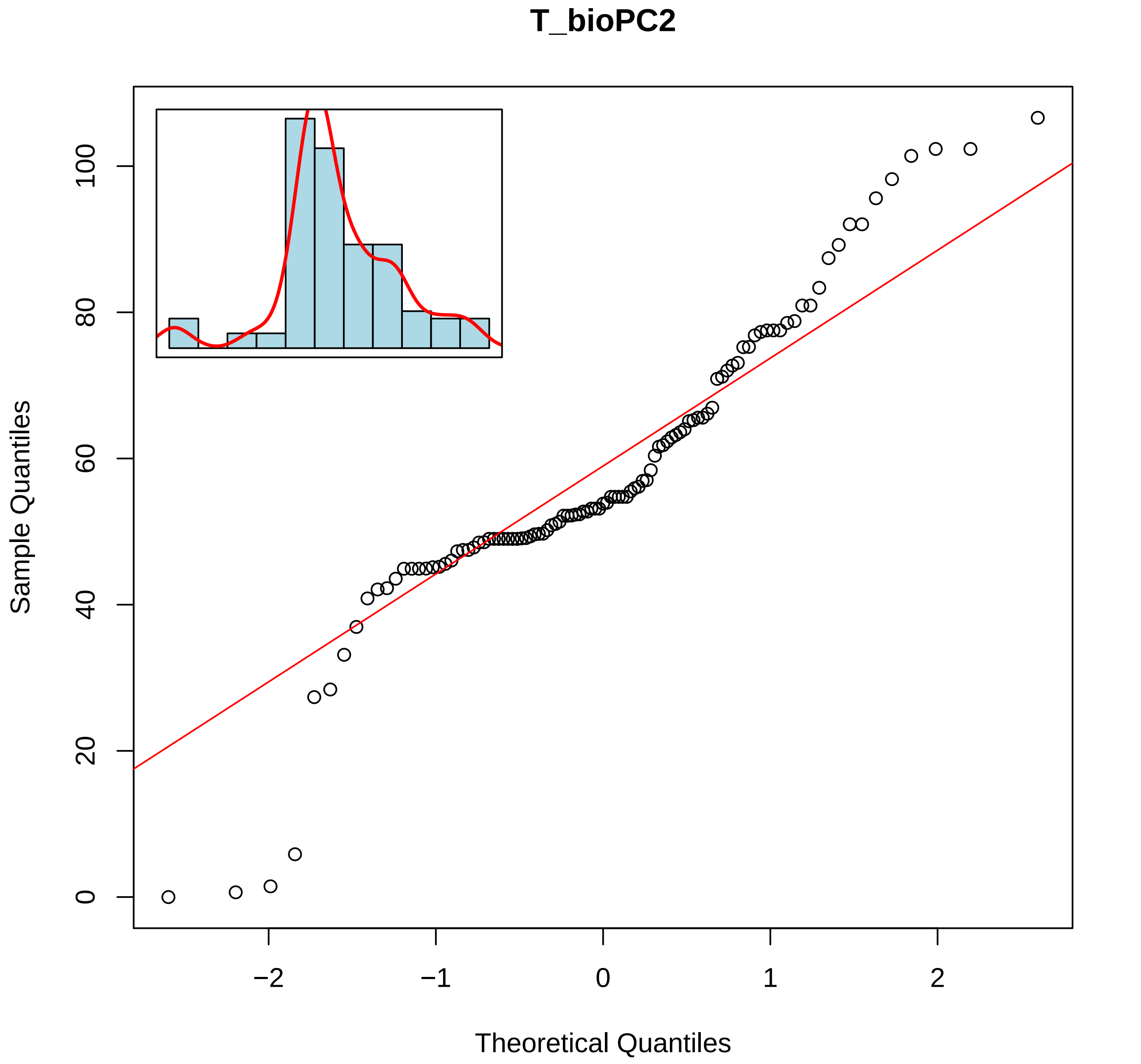
T_bioPC2
Sample Quantiles
Theoretical Quantiles
0
20
40
60
80
100
−2
−1
0
1
2

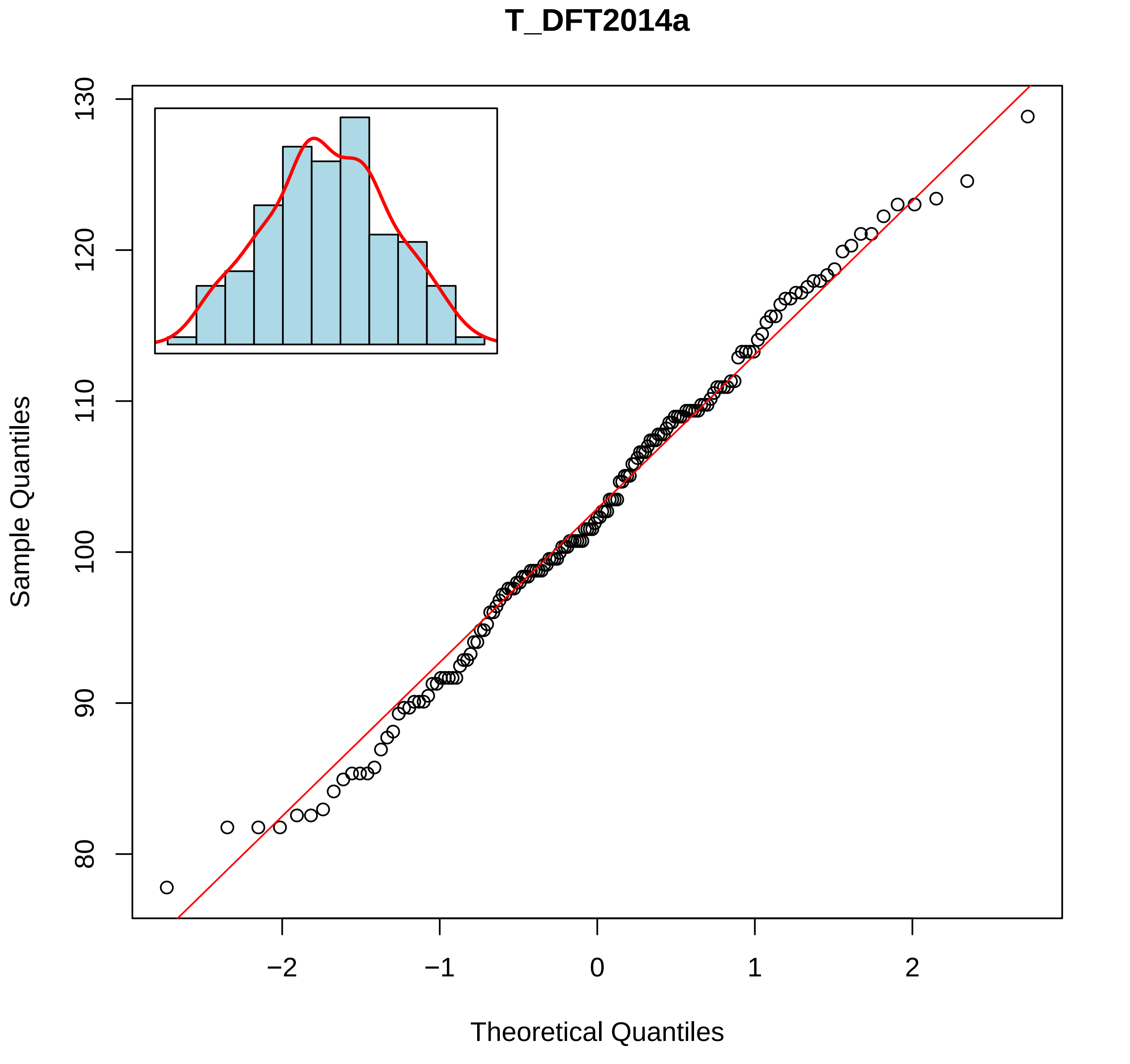
T_DFT2014a
Sample Quantiles
Theoretical Quantiles
130
120
110
100
90
80
−2
−1
0
1
2

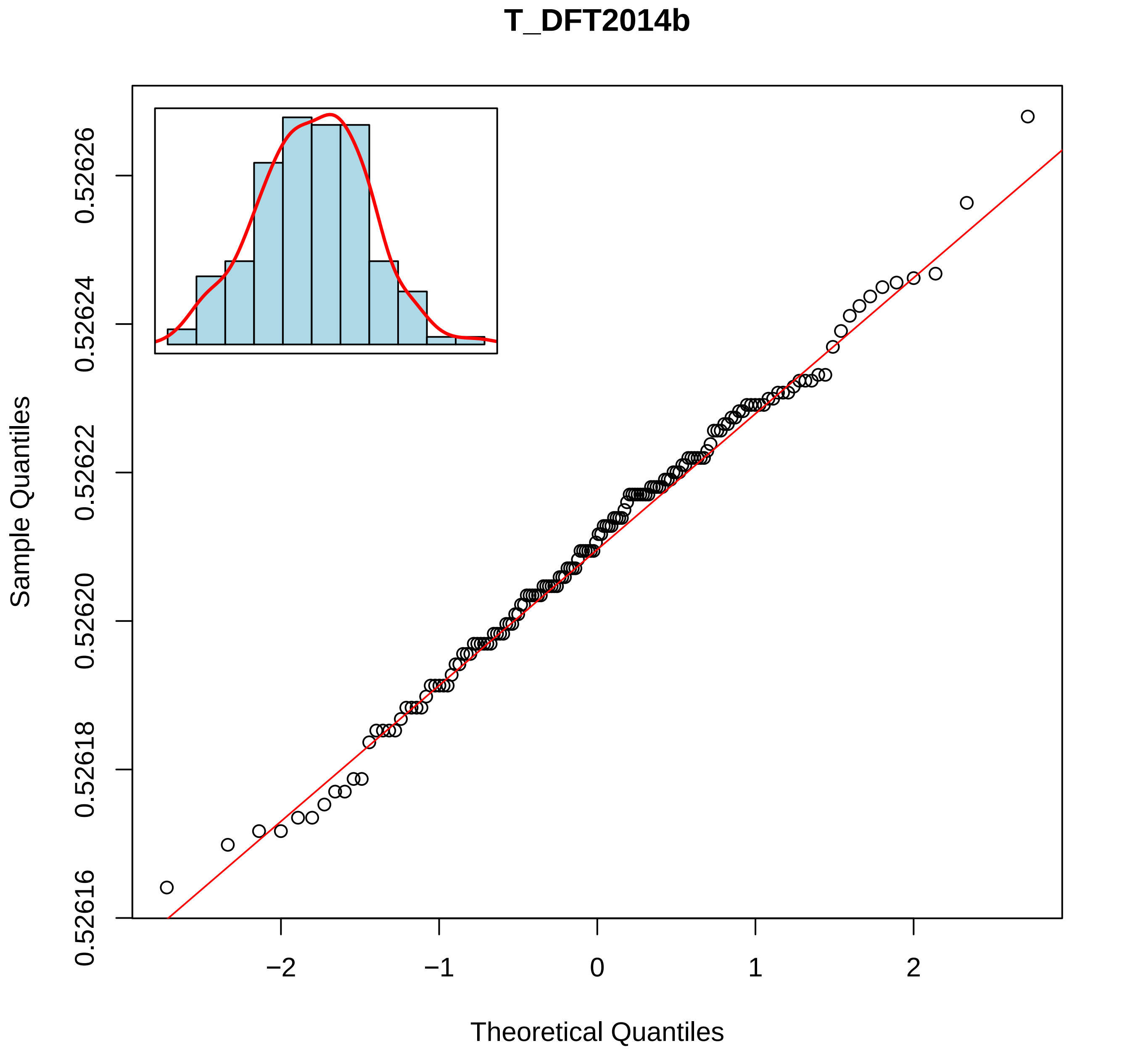

T_DFT2014b
Sample Quantiles
Theoretical Quantiles
0.52616
0.52618
0.52620
0.52622
0.52624
0.52626
−2
−1
0
1
2

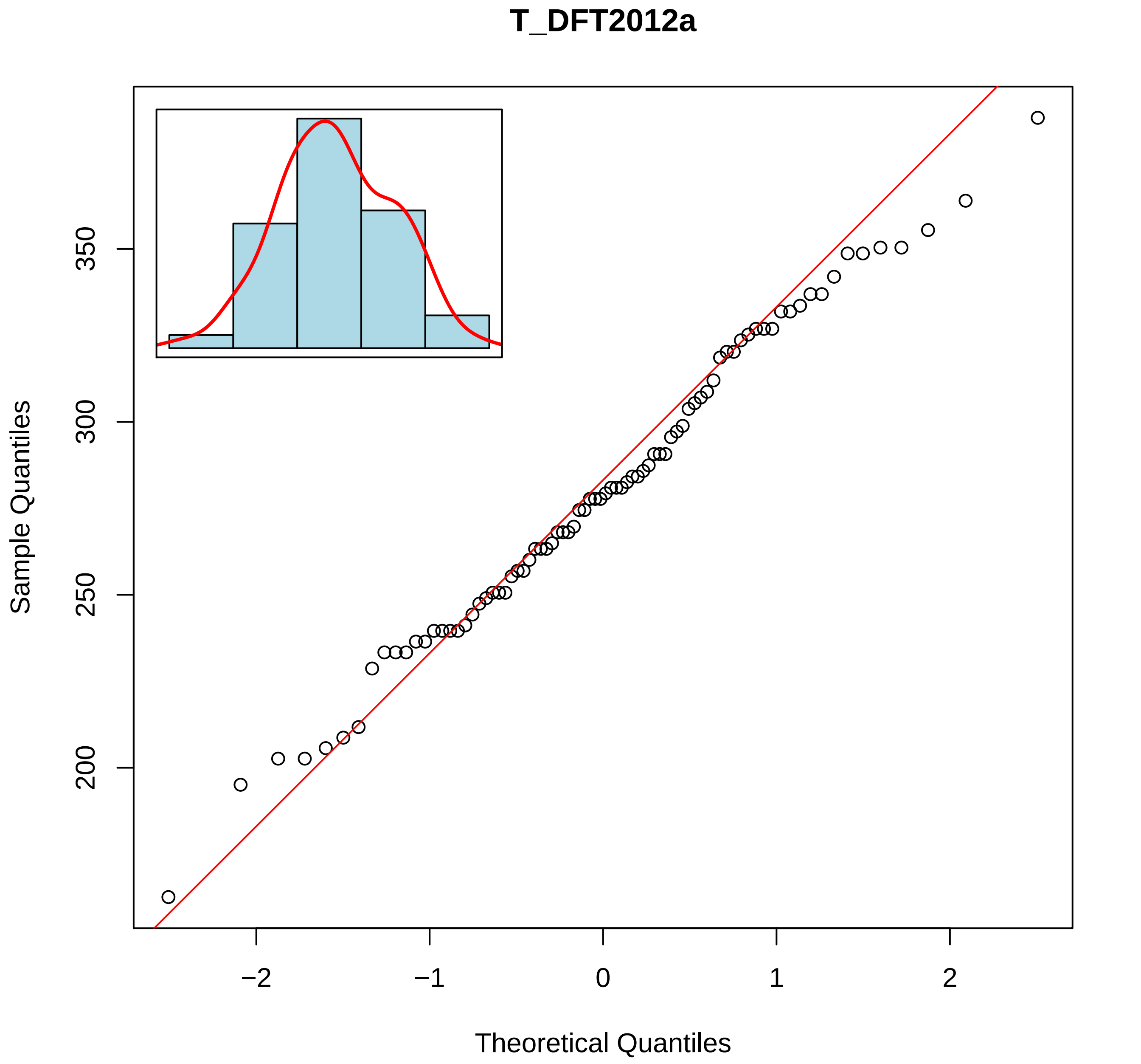
T_DFT2012a
Sample Quantiles
200
250
300
350
Theoretical Quantiles
−2
−1
0
1
2

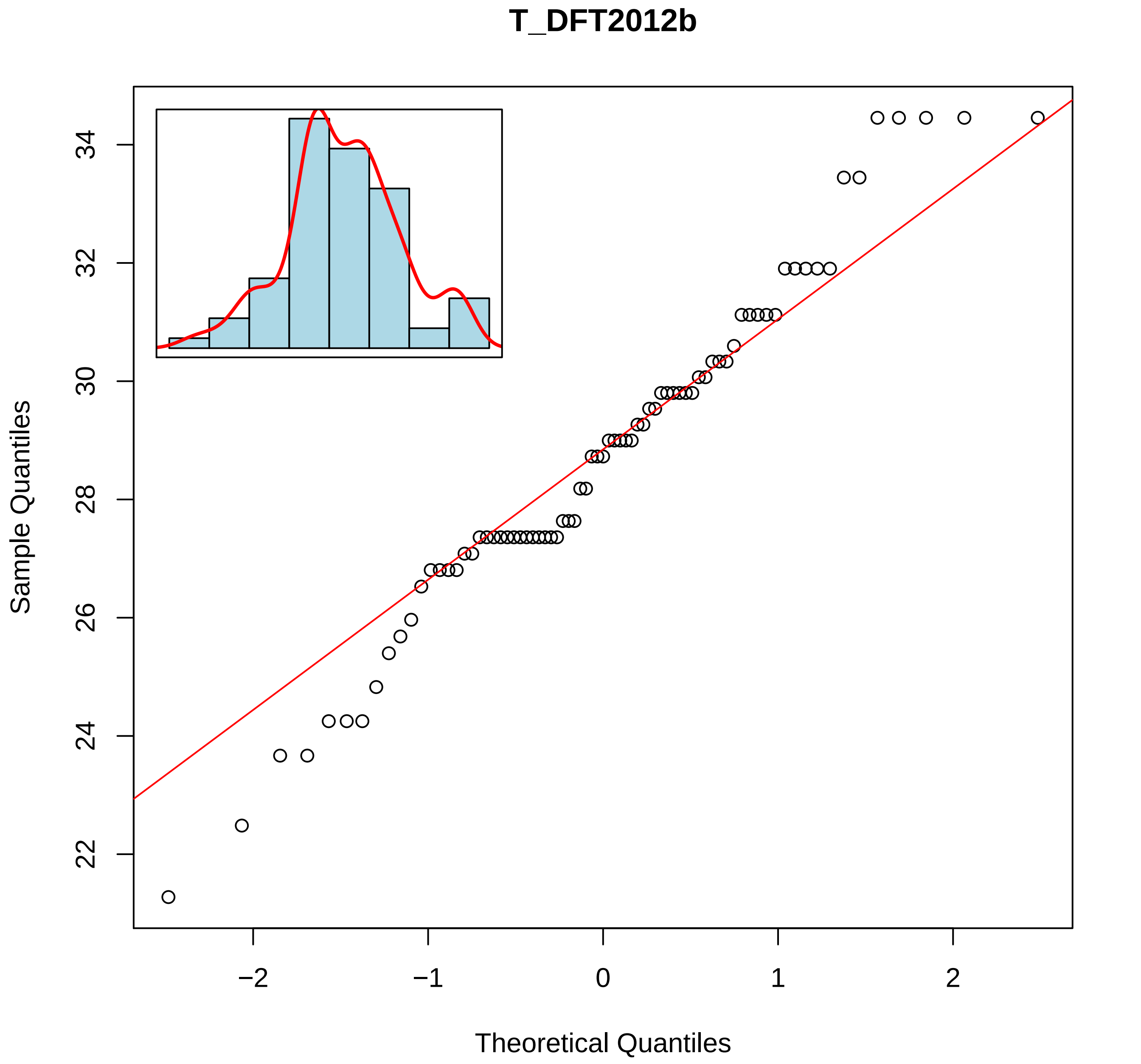

T_DFT2012b
Sample Quantiles
Theoretical Quantiles
22
24
26
28
30
32
34
−2
−1
0
1
2

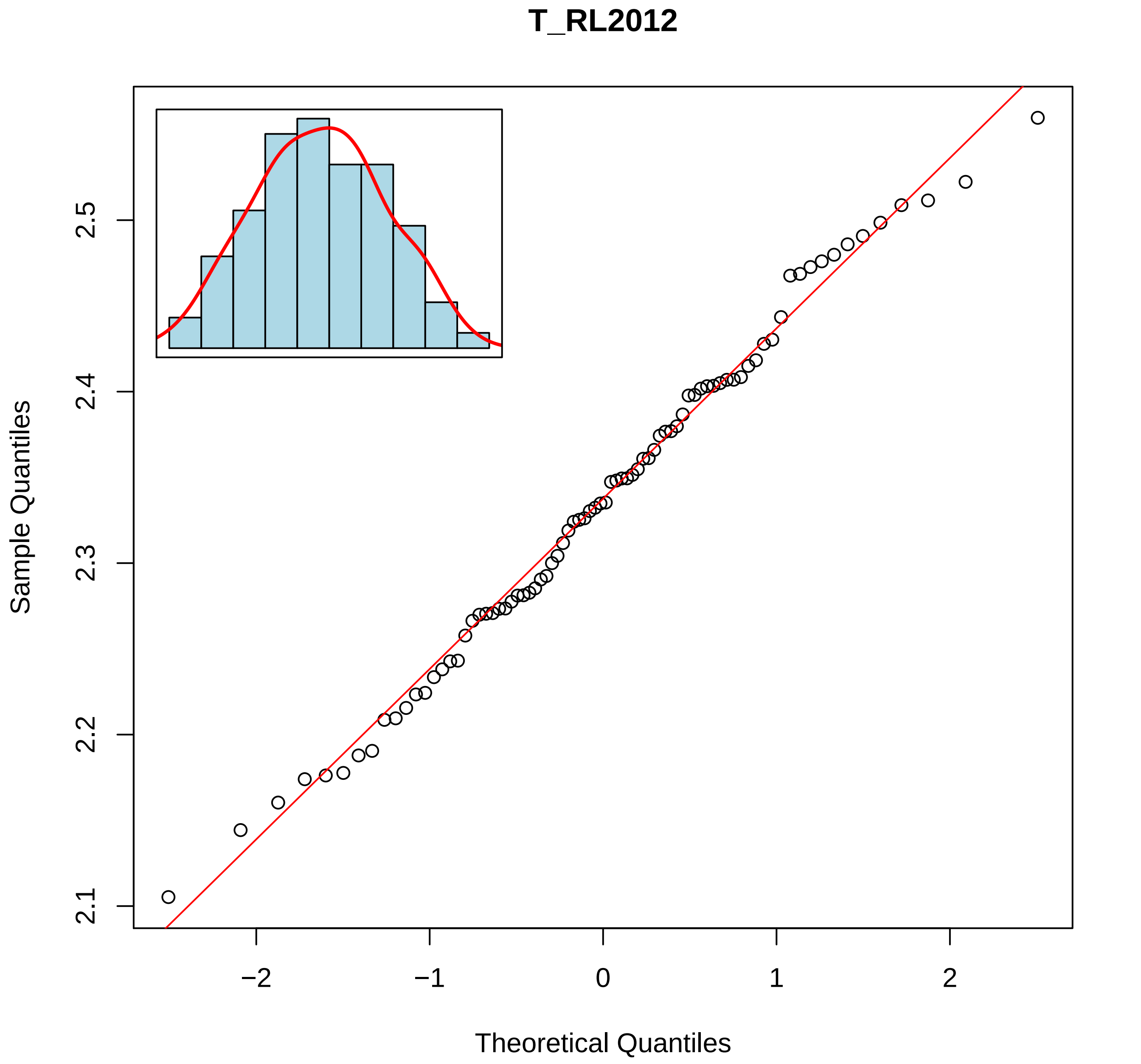
T_RL2012
Sample Quantiles
Theoretical Quantiles
2.1
2.2
2.3
2.4
2.5
−2
−1
0
1
2

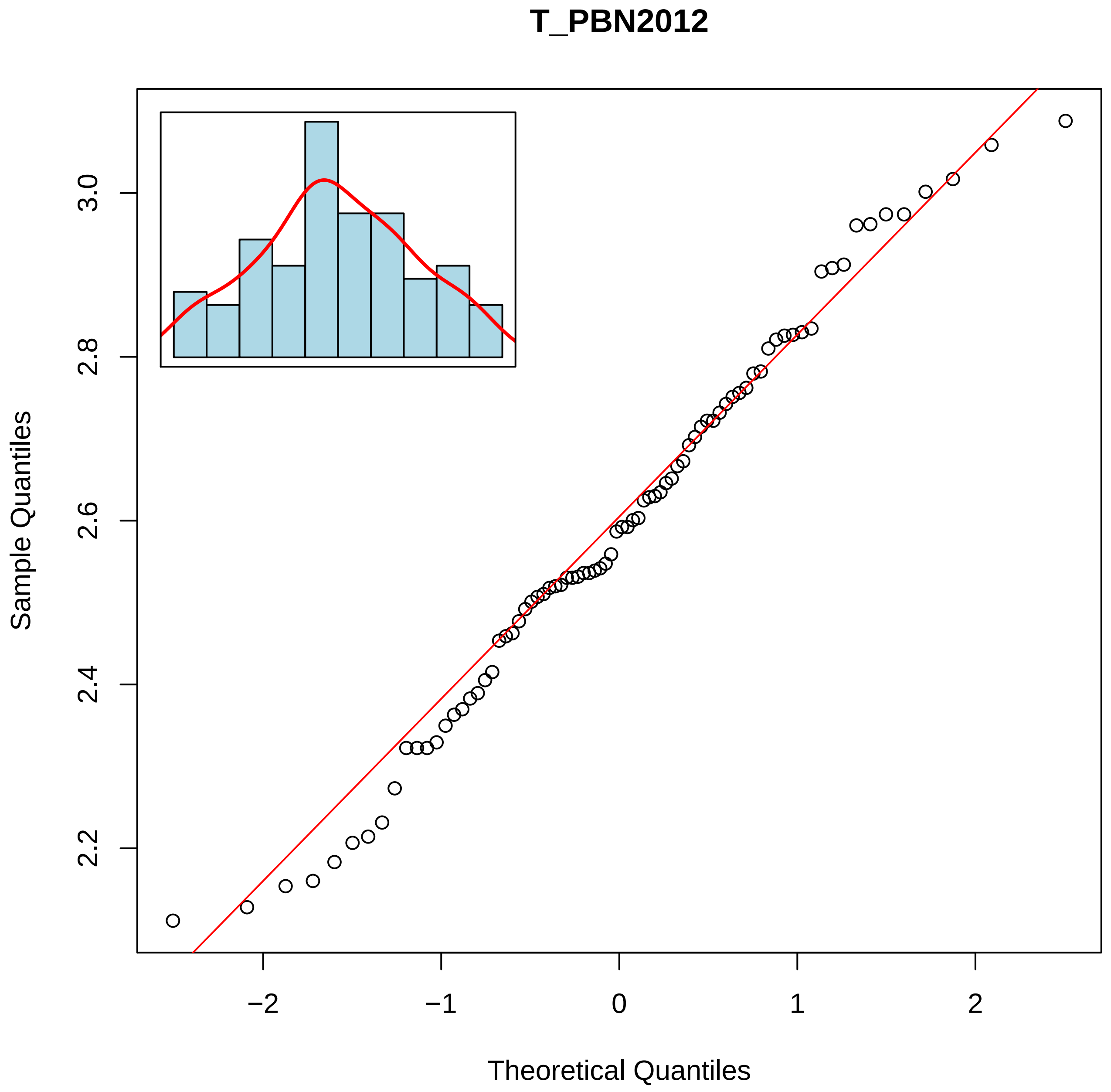
T_PBN2012
Sample Quantiles
Theoretical Quantiles
2.2
2.4
2.6
2.8
3.0
−2
−1
0
1
2

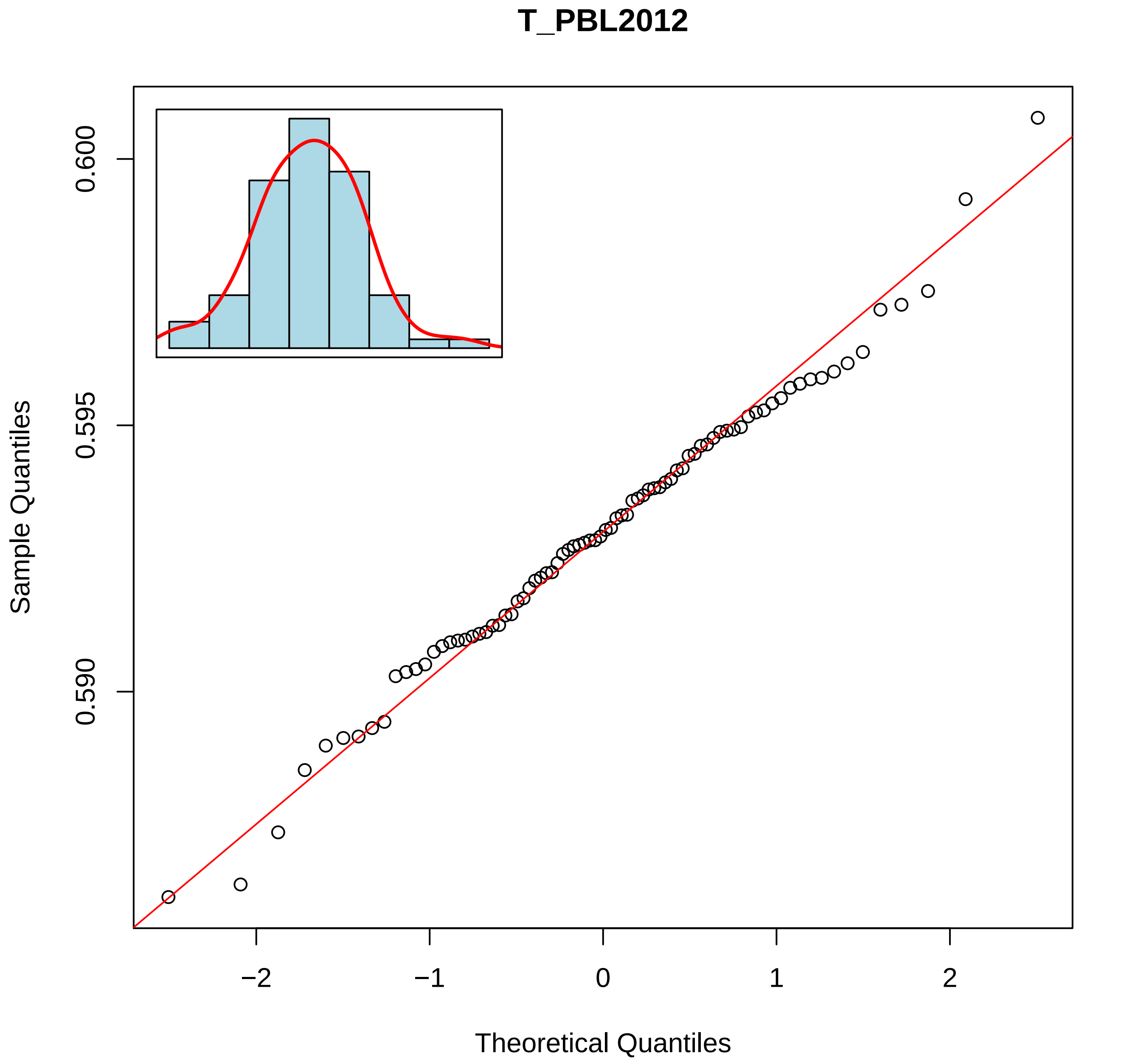
T_PBL2012
Sample Quantiles
0.590
0.595
0.600
Theoretical Quantiles
−2
−1
0
1
2

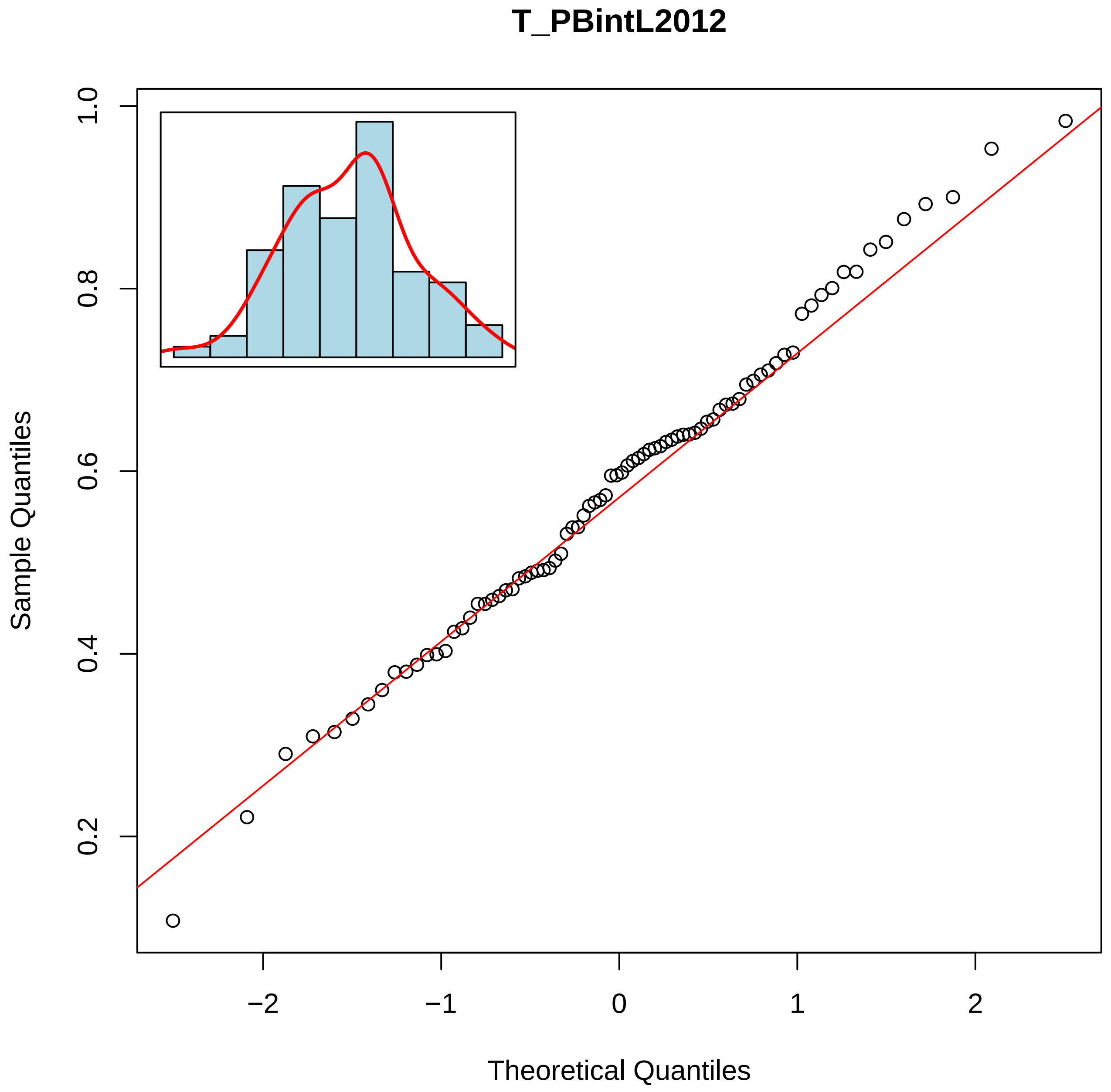

T_PBintL2012
Sample Quantiles
Theoretical Quantiles
0.2
0.4
0.6
0.8
1.0
−2
−1
0
1
2

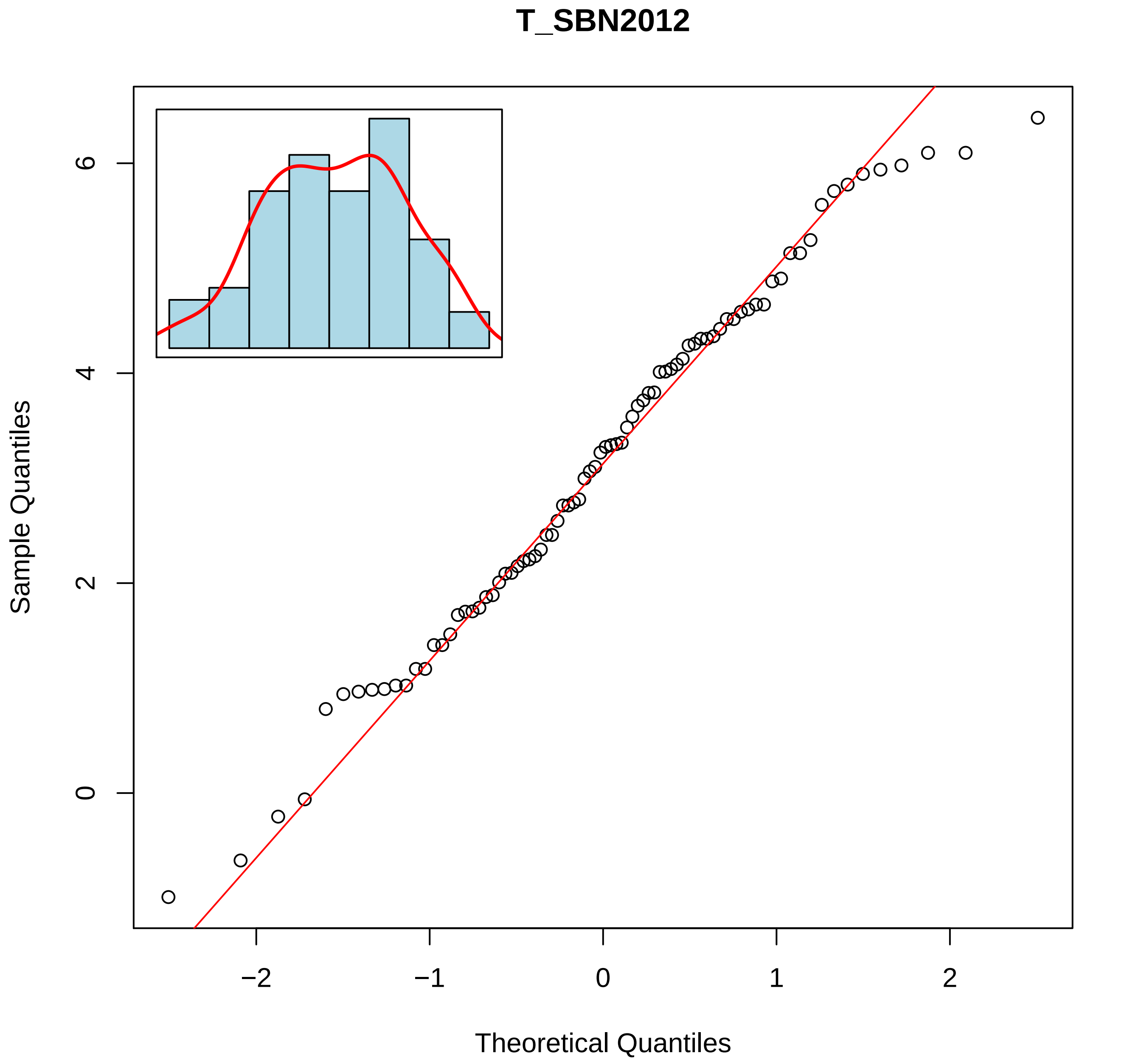

T_SBN2012
Sample Quantiles
Theoretical Quantiles
0
2
4
6
−2
−1
0
1
2

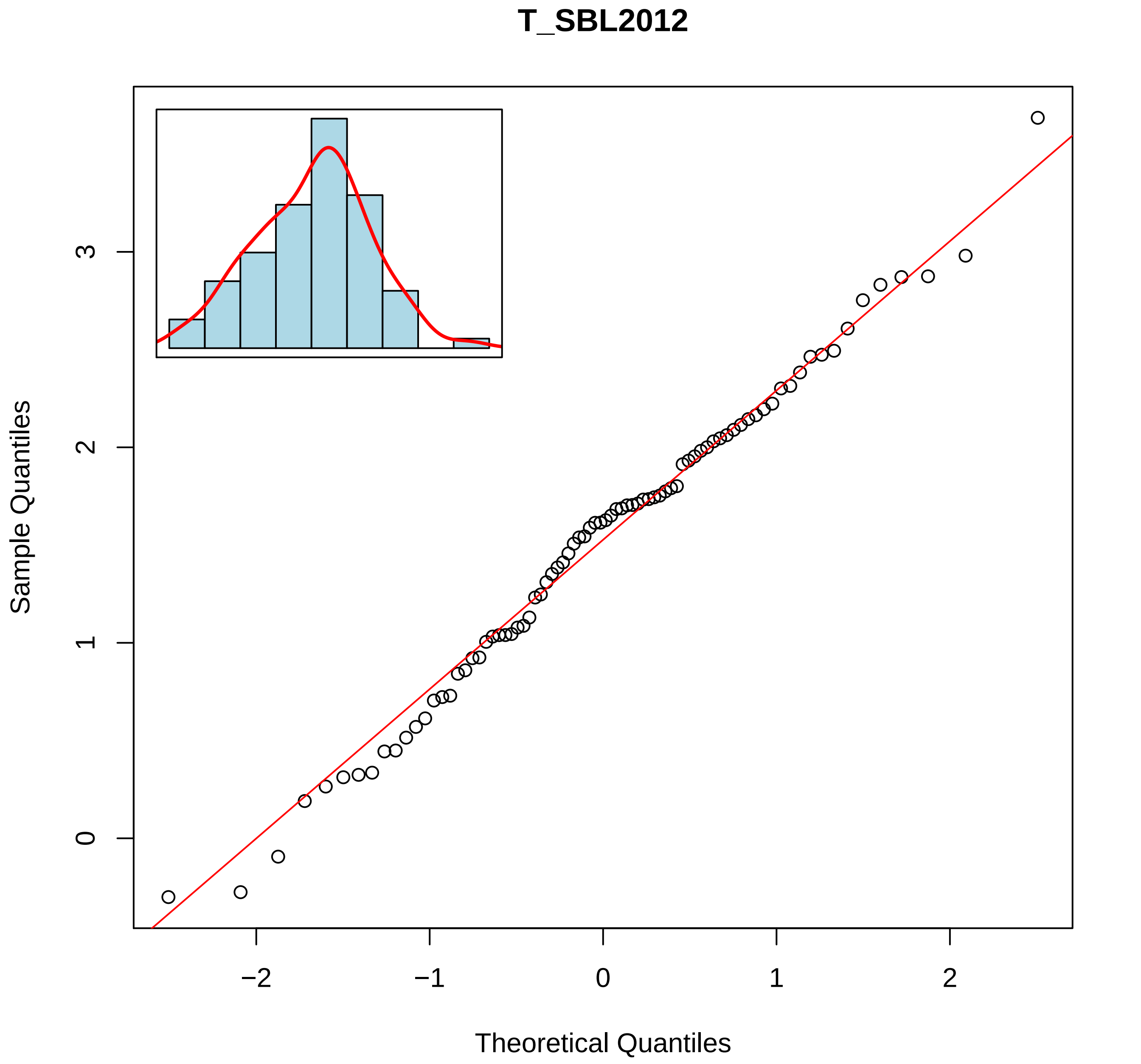

T_SBL2012
Sample Quantiles
Theoretical Quantiles
0
1
2
3
−2
−1
0
1
2

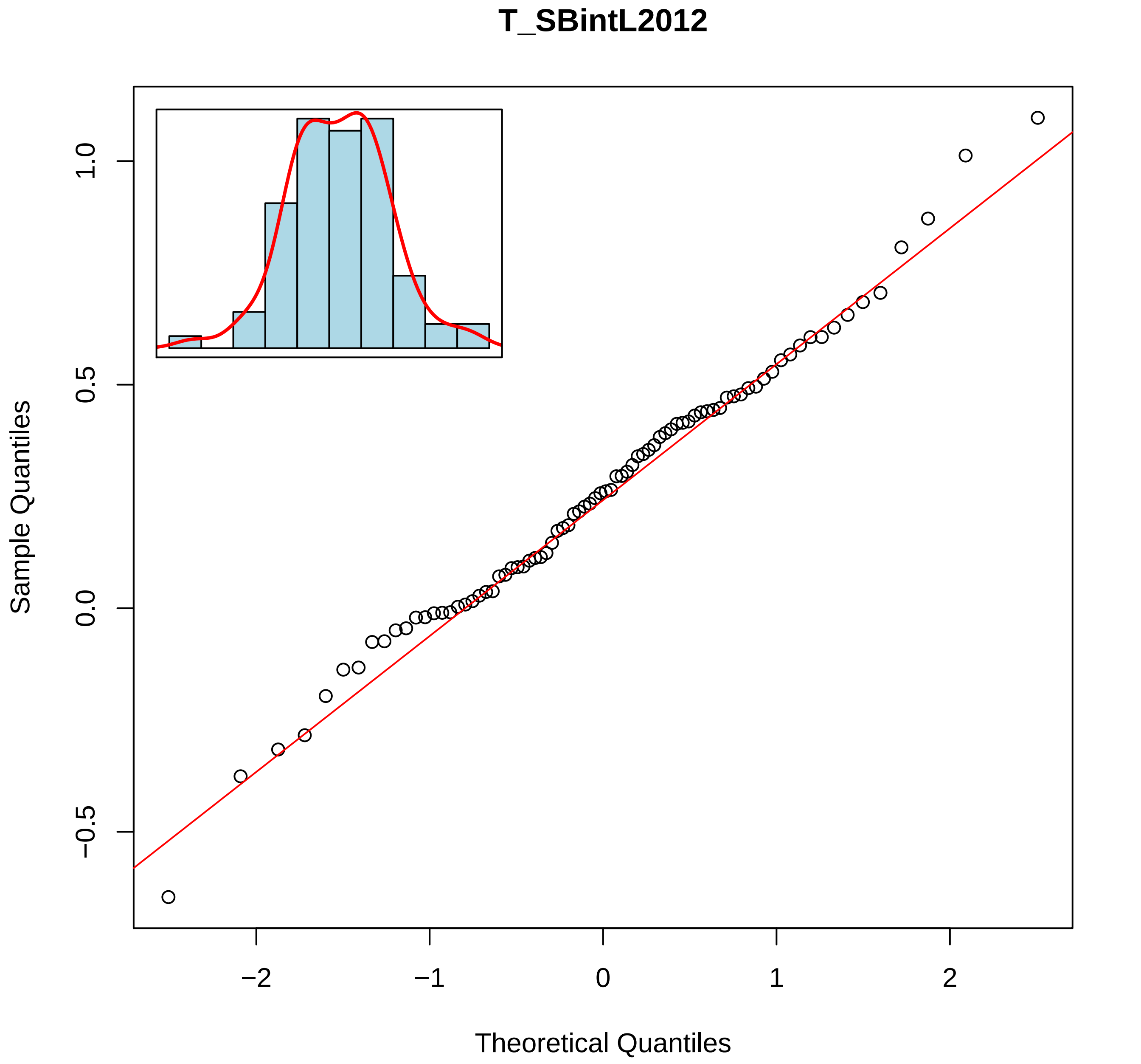
T_SBintL2012
Sample Quantiles
Theoretical Quantiles
−0.5
0.0
0.5
1.0
−2
−1
0
1
2

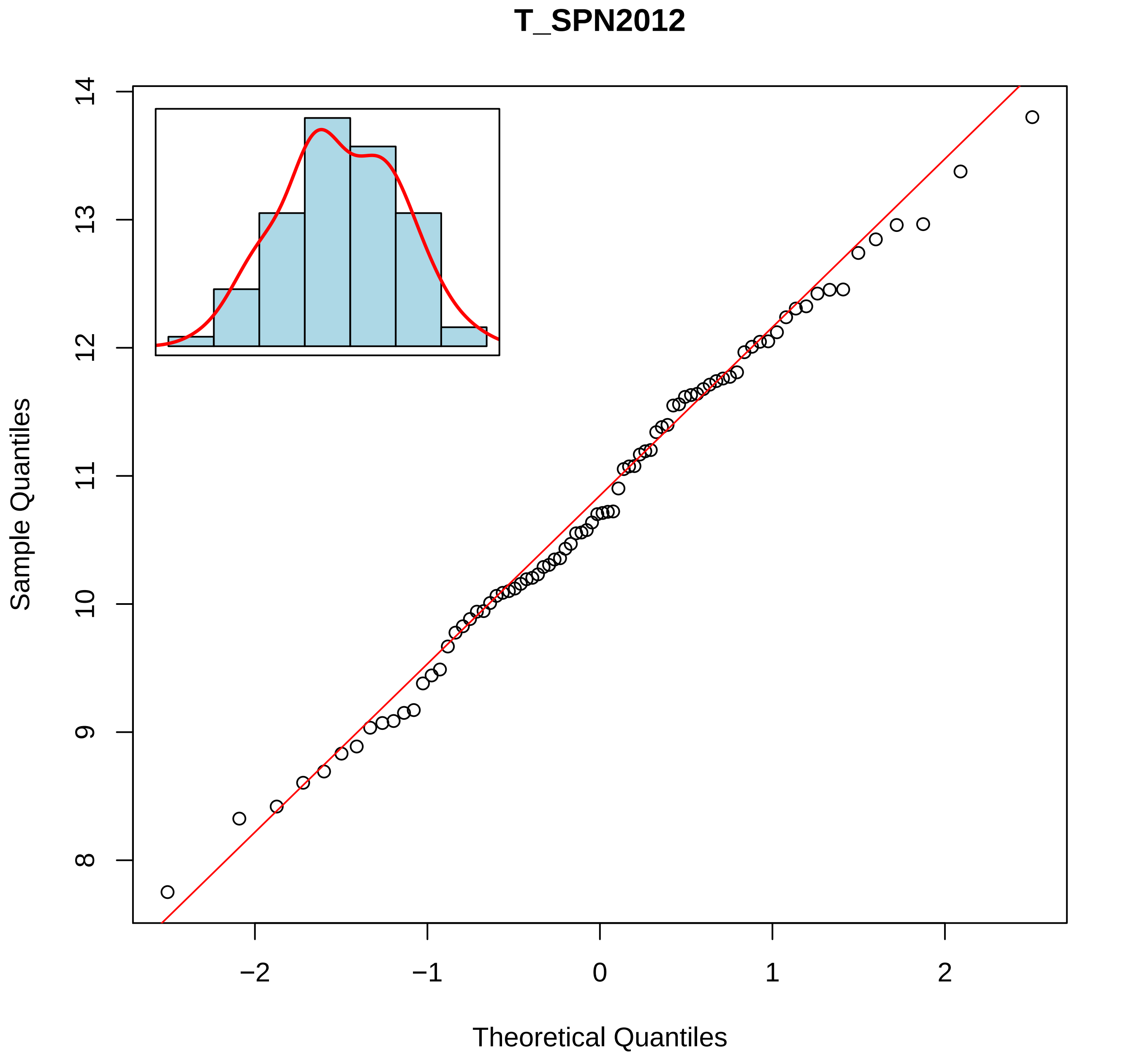
T_SPN2012
Sample Quantiles
Theoretical Quantiles
14
13
12
11
10
9
8
−2
−1
0
1
2

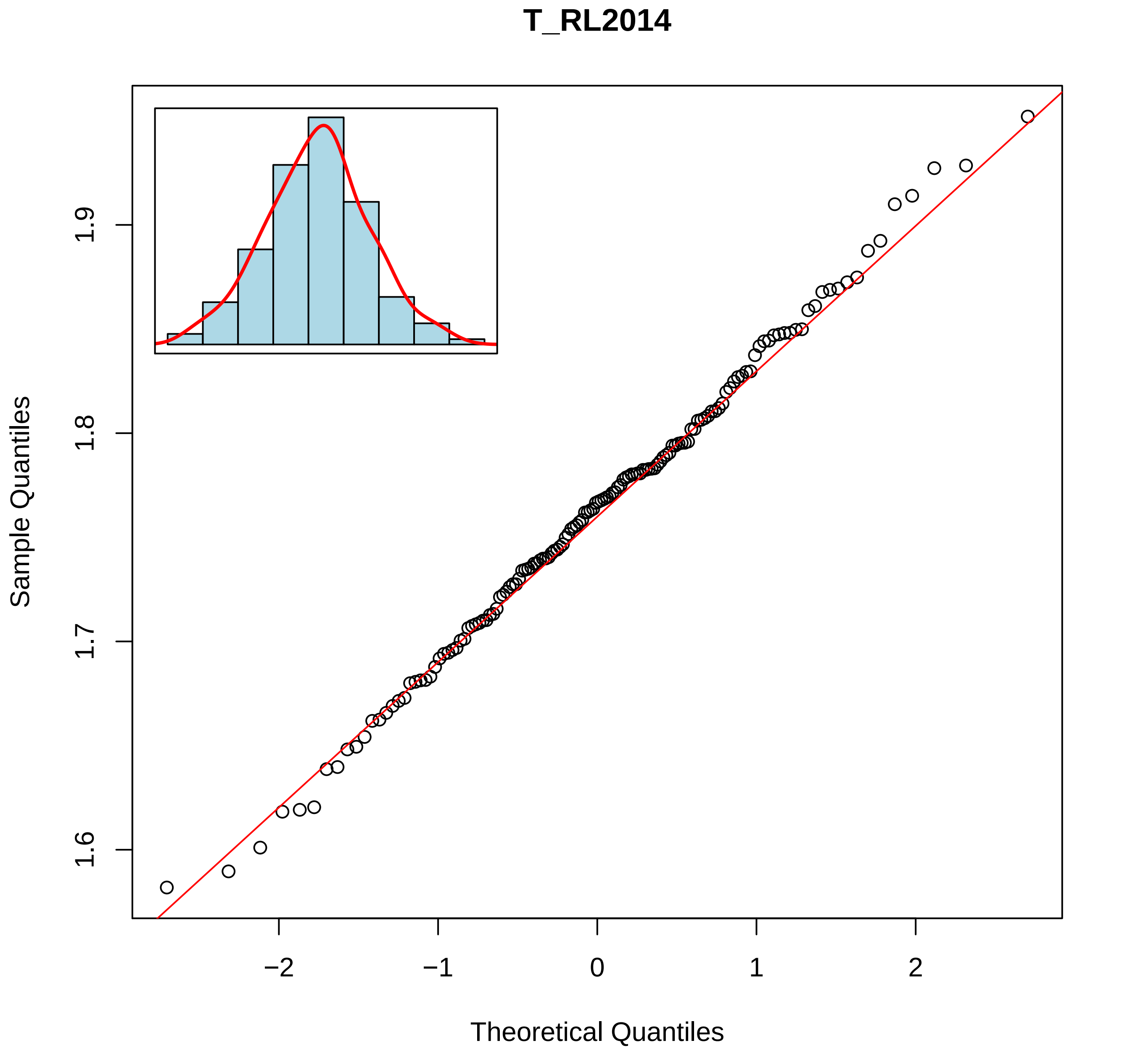
T_RL2014
Sample Quantiles
1.6
1.7
1.8
1.9
Theoretical Quantiles
−2
−1
0
1
2

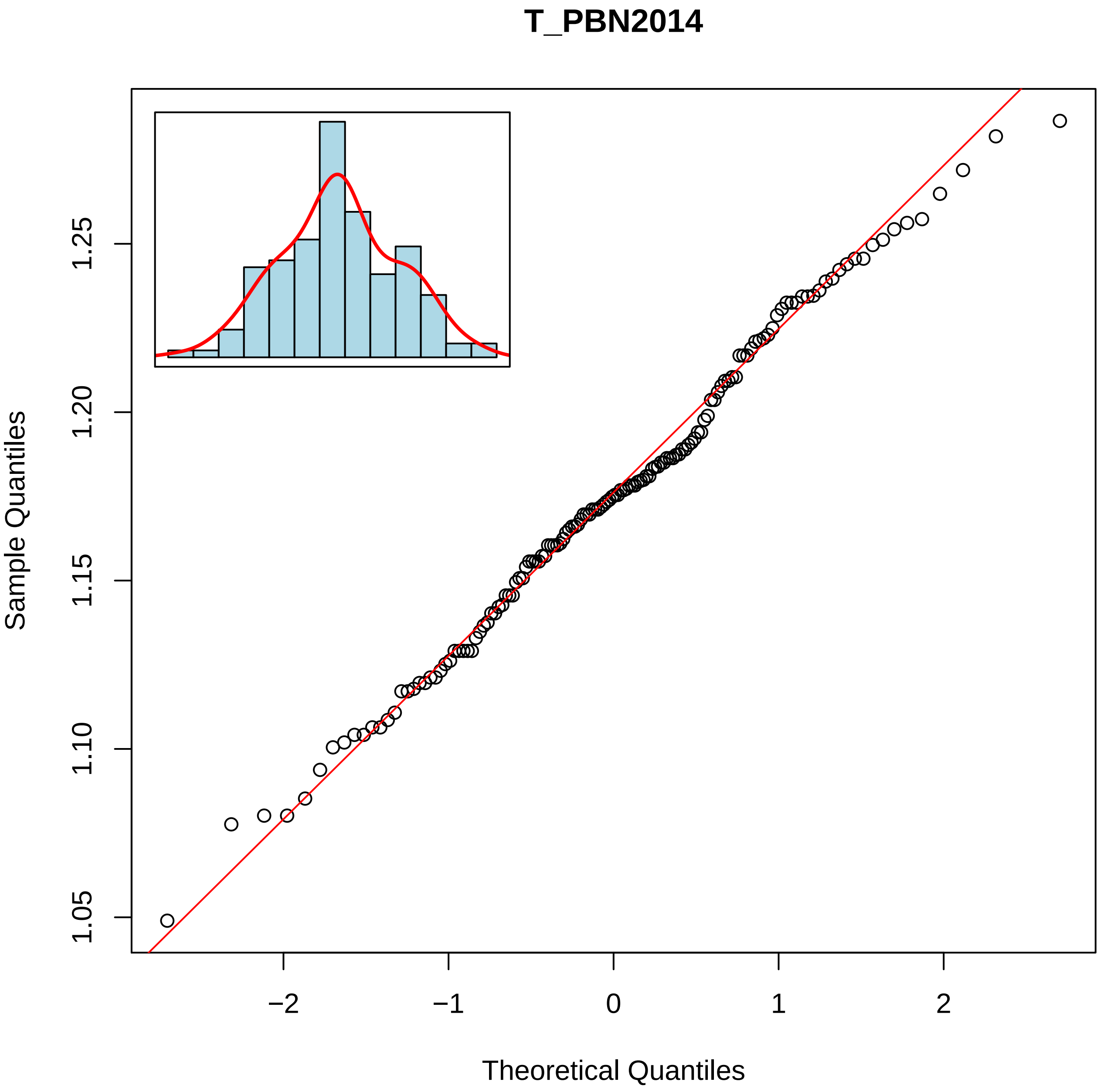
T_PBN2014
Sample Quantiles
Theoretical Quantiles
1.05
1.10
1.15
1.20
1.25
−2
−1
0
1
2

T_PBL2014

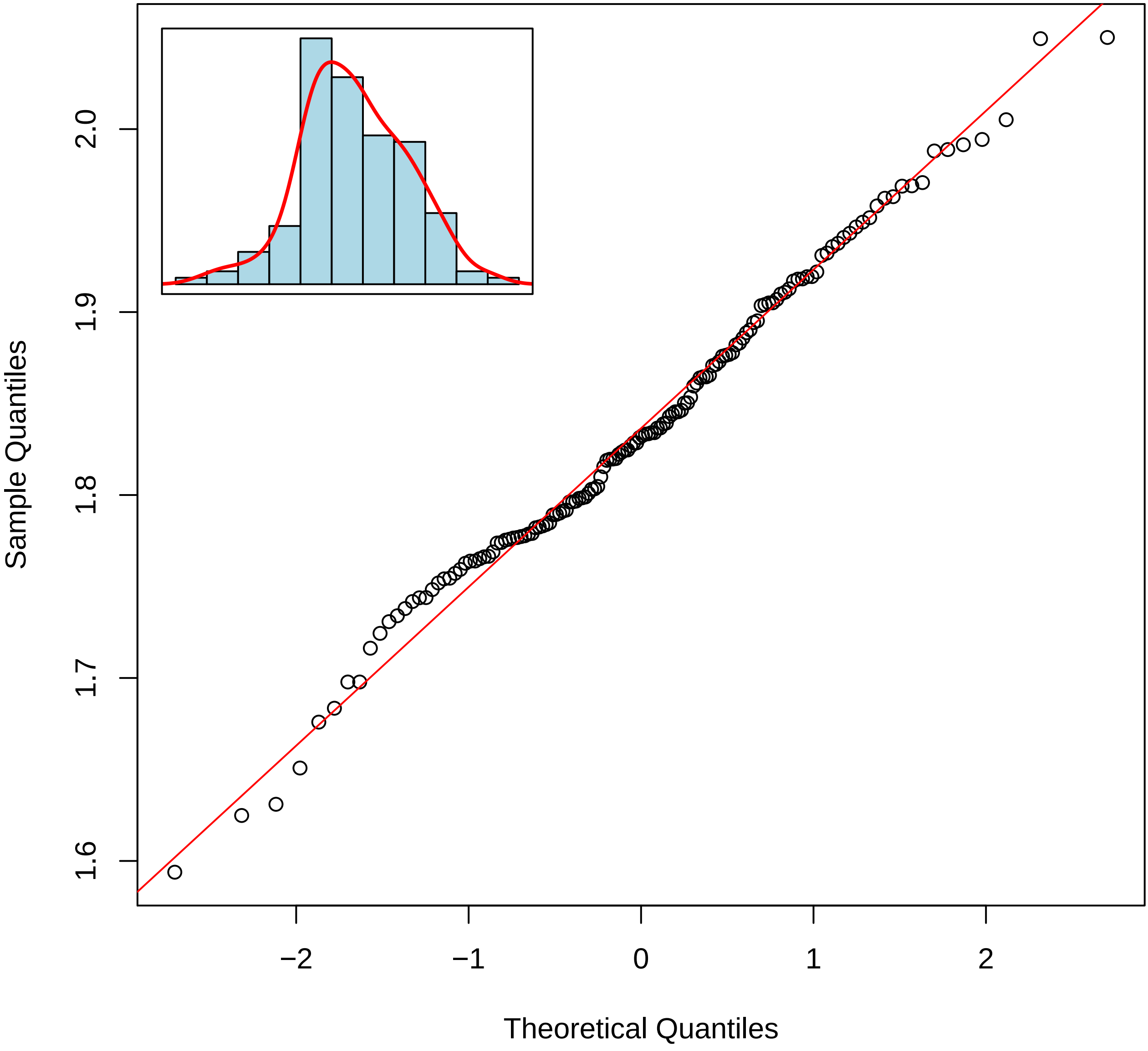

2.0
1.9
1.8
1.7
1.6
Sample Quantiles
−2
−1
0
1
2
Theoretical Quantiles

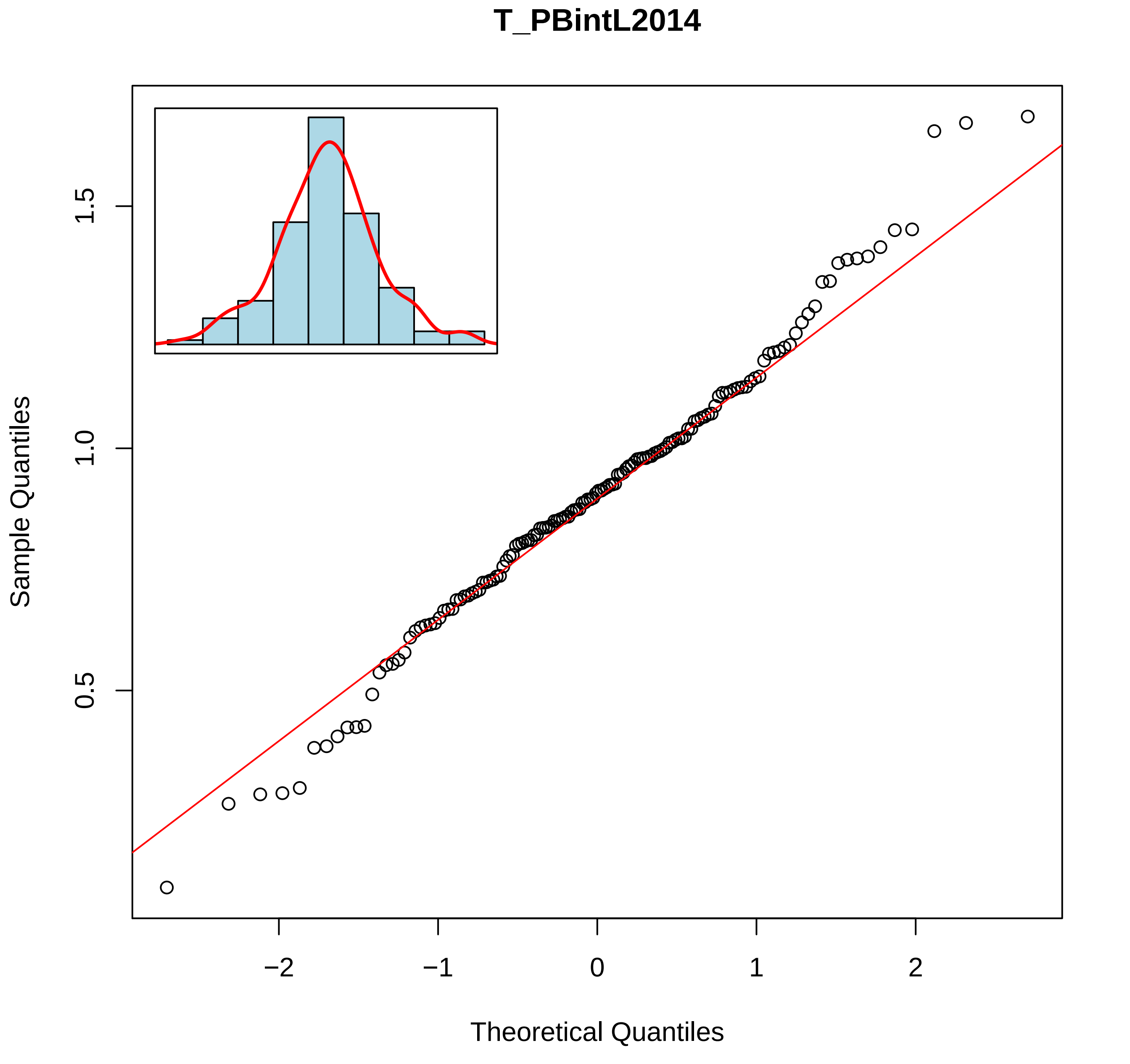
T_PBintL2014
Sample Quantiles
Theoretical Quantiles
0.5
1.0
1.5
−2
−1
0
1
2

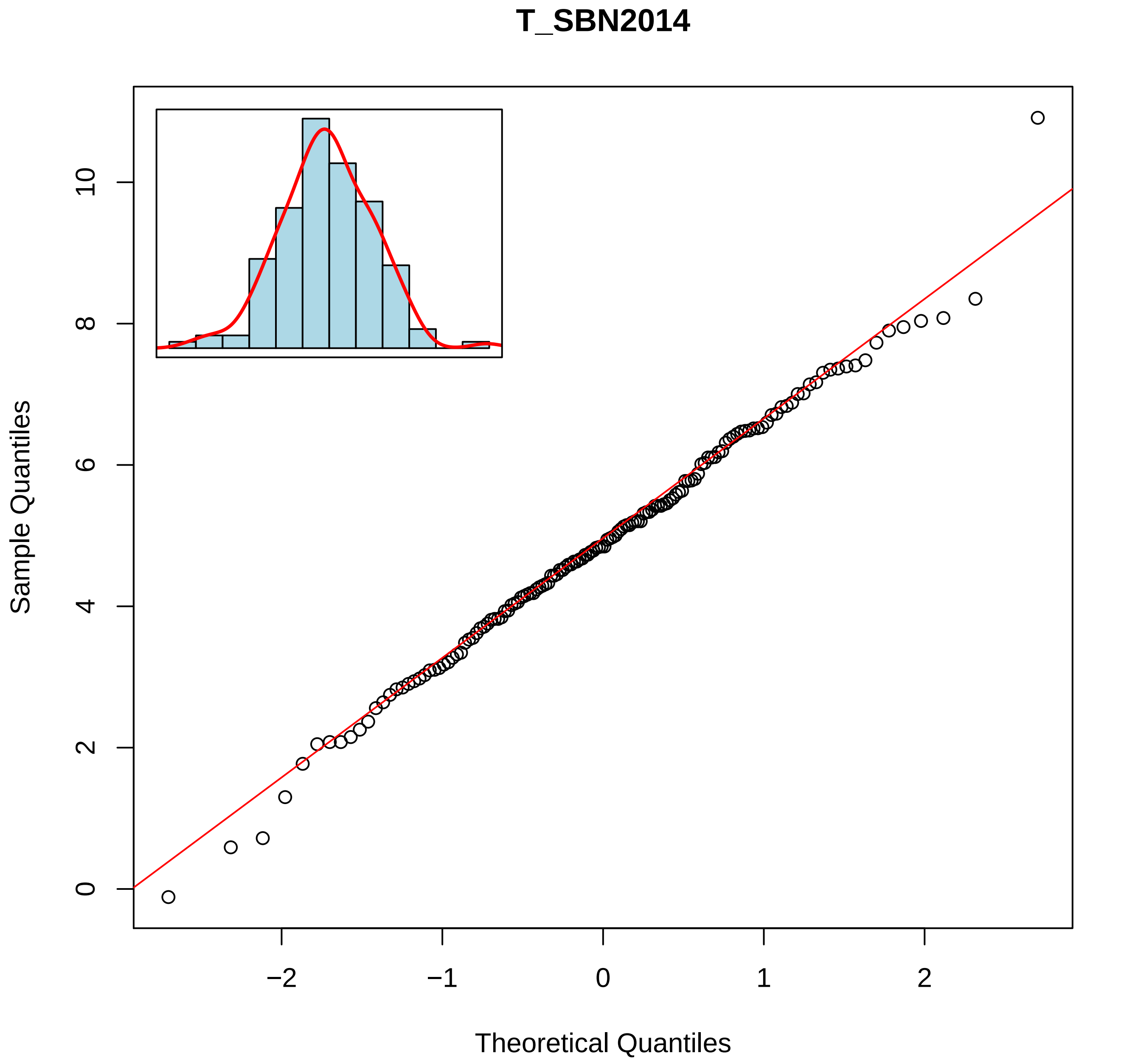
T_SBN2014
Sample Quantiles
Theoretical Quantiles
0
2
4
6
8
10
−2
−1
0
1
2

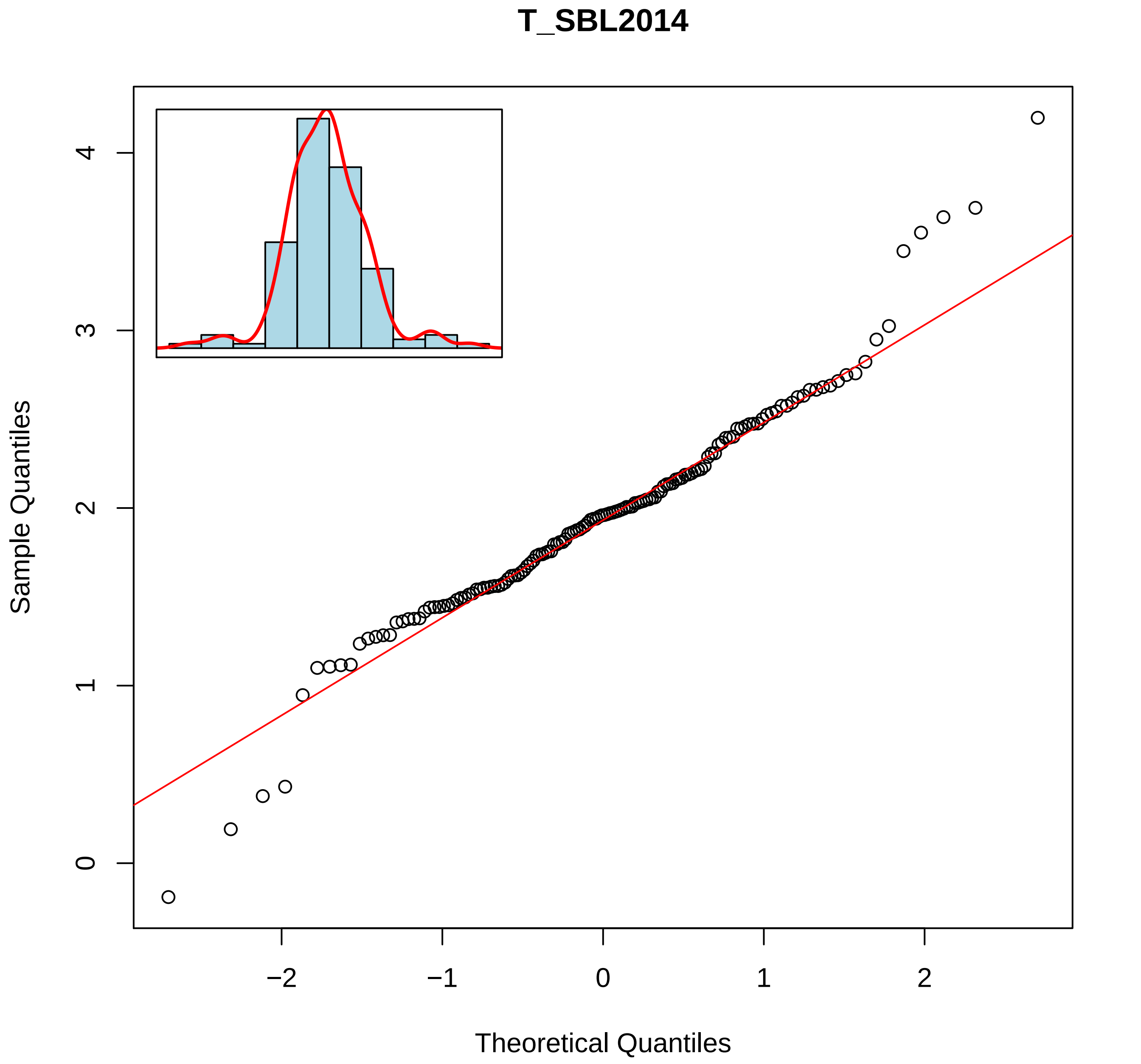
T_SBL2014
Sample Quantiles
Theoretical Quantiles
0
1
2
3
4
−2
−1
0
1
2

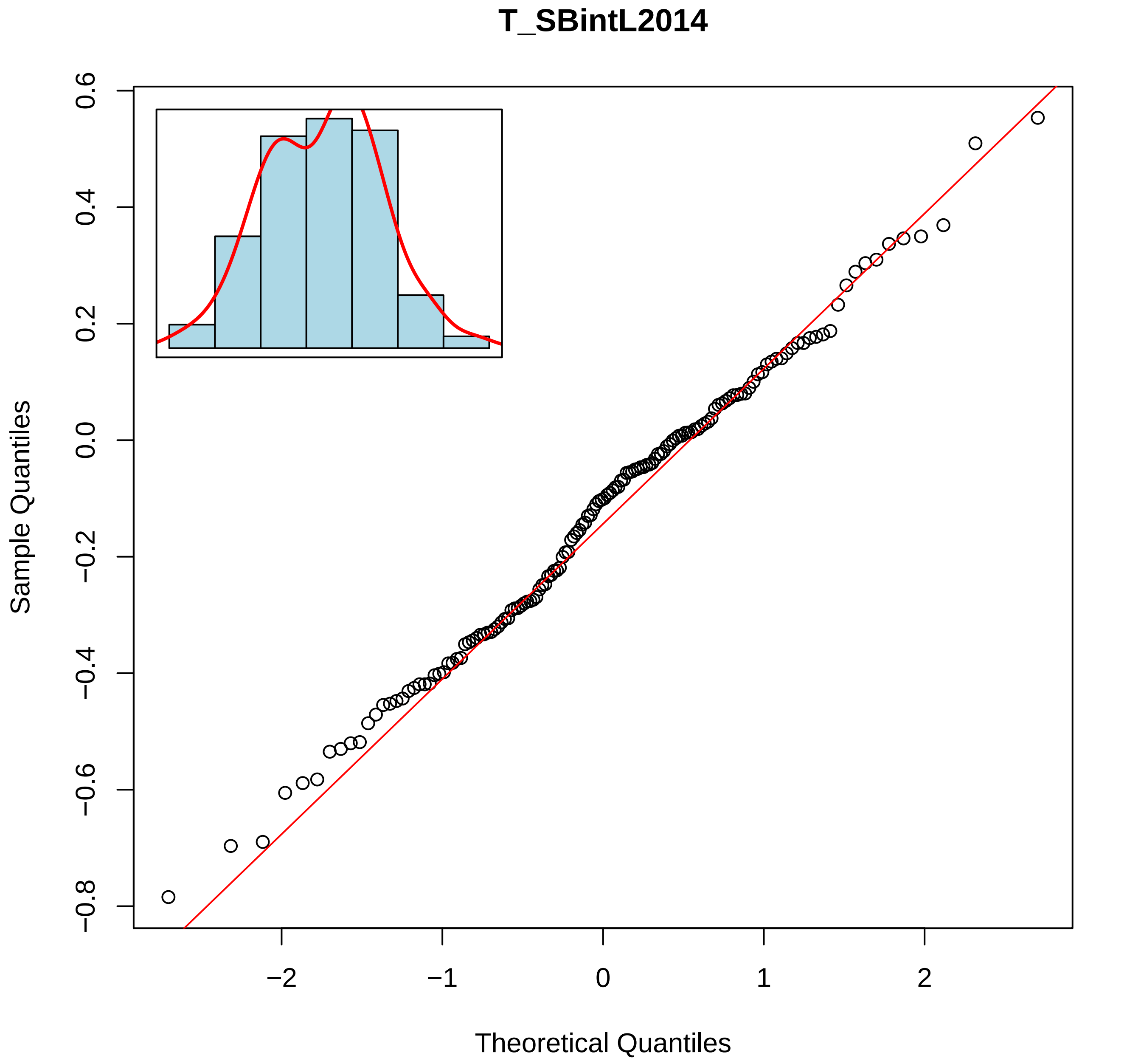
T_SBintL2014
Sample Quantiles
Theoretical Quantiles
0.6
0.4
0.2
0.0
−0.2
−0.4
−0.6
−0.8
−2
−1
0
1
2

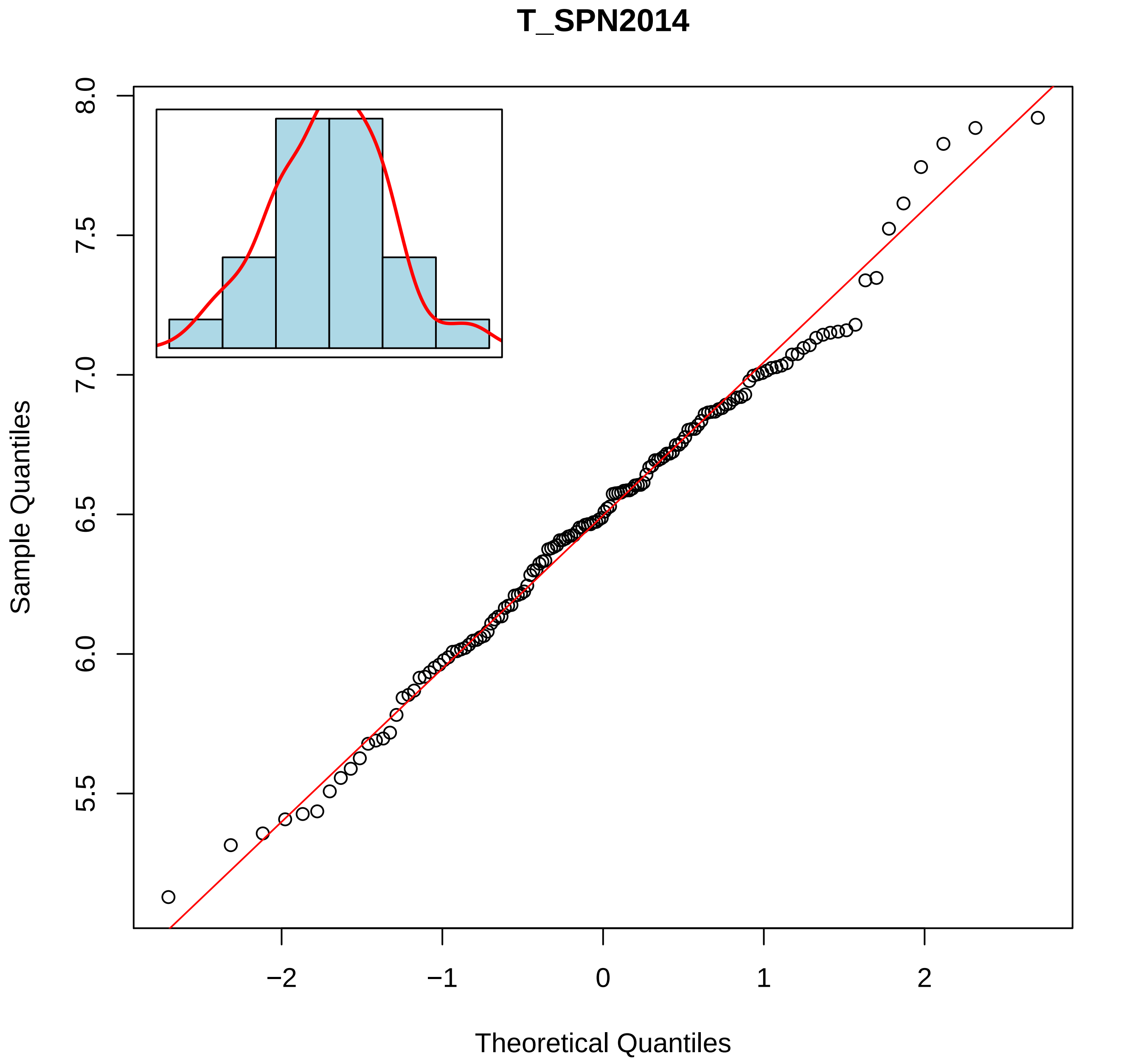
T_SPN2014
Sample Quantiles
Theoretical Quantiles
8.0
7.5
7.0
6.5
6.0
5.5
−2
−1
0
1
2

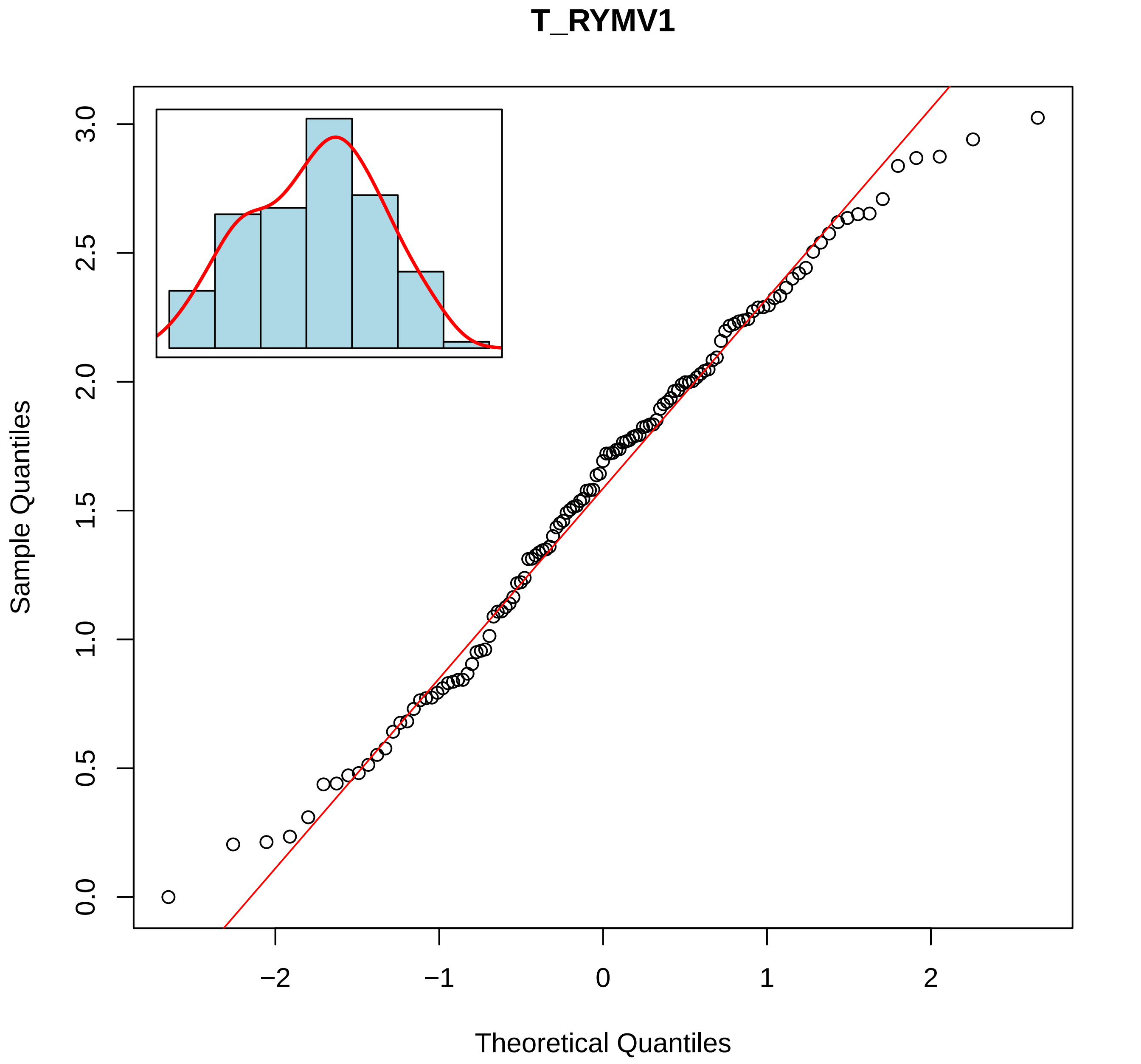
T_RYMV1
Sample Quantiles
Theoretical Quantiles
0.0
0.5
1.0
1.5
2.0
2.5
3.0
−2
−1
0
1
2

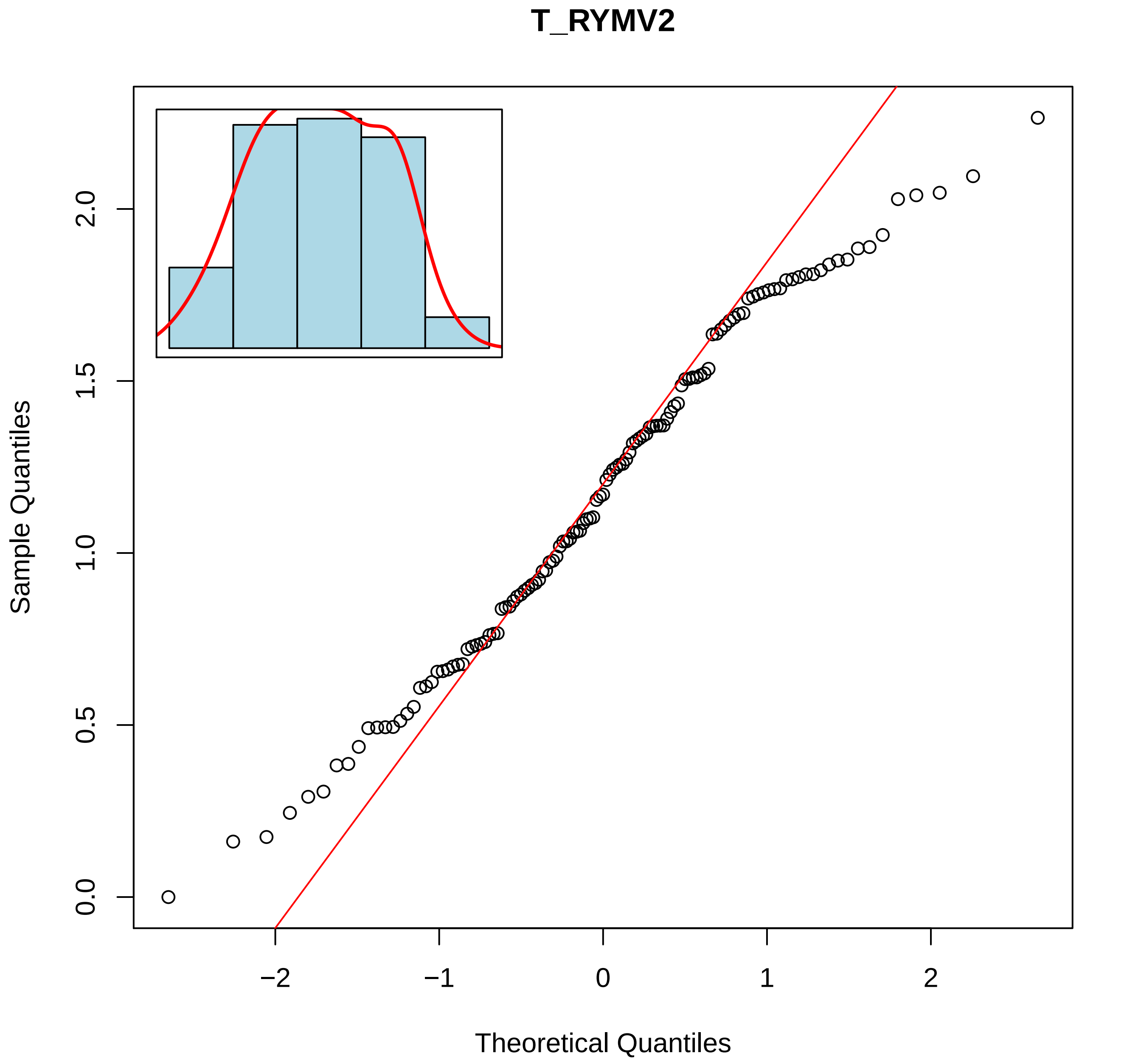
T_RYMV2
Sample Quantiles
Theoretical Quantiles
0.0
0.5
1.0
1.5
2.0
−2
−1
0
1
2

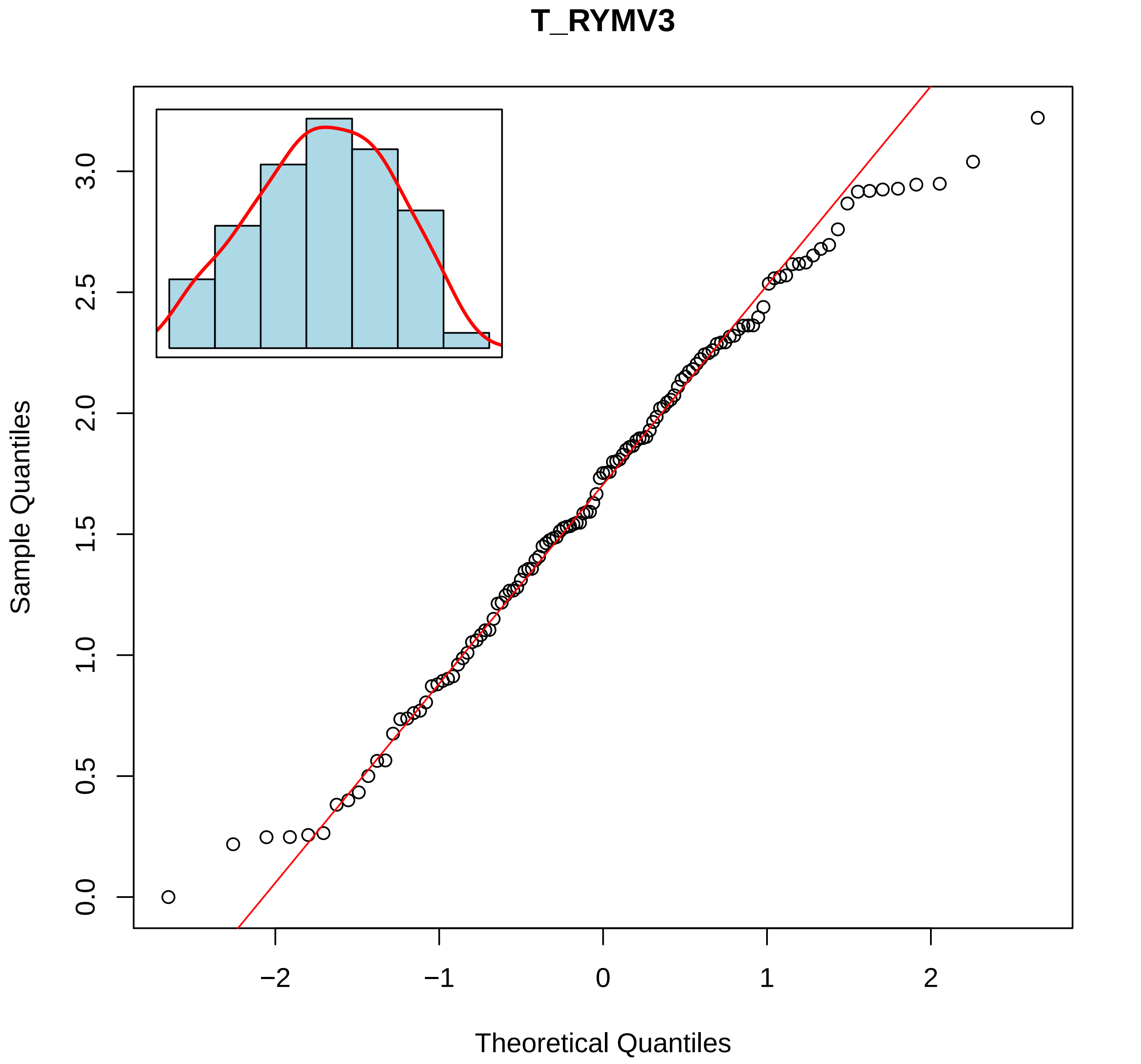

T_RYMV3
Sample Quantiles
0.0
0.5
1.0
1.5
2.0
2.5
3.0
−2
−1
0
1
2
Theoretical Quantiles
