# Supplementary figures and images for: Genome Wide Association Study Pinpoints Key Agronomic QTLs in African Rice Oryza glaberrima
Source: Rice (N Y). 2020 Sep 16;13:66. doi: 10.1186/s12284-020-00424-1 (PMC7494698; doi:10.1186/s12284-020-00424-1)

## LD decay

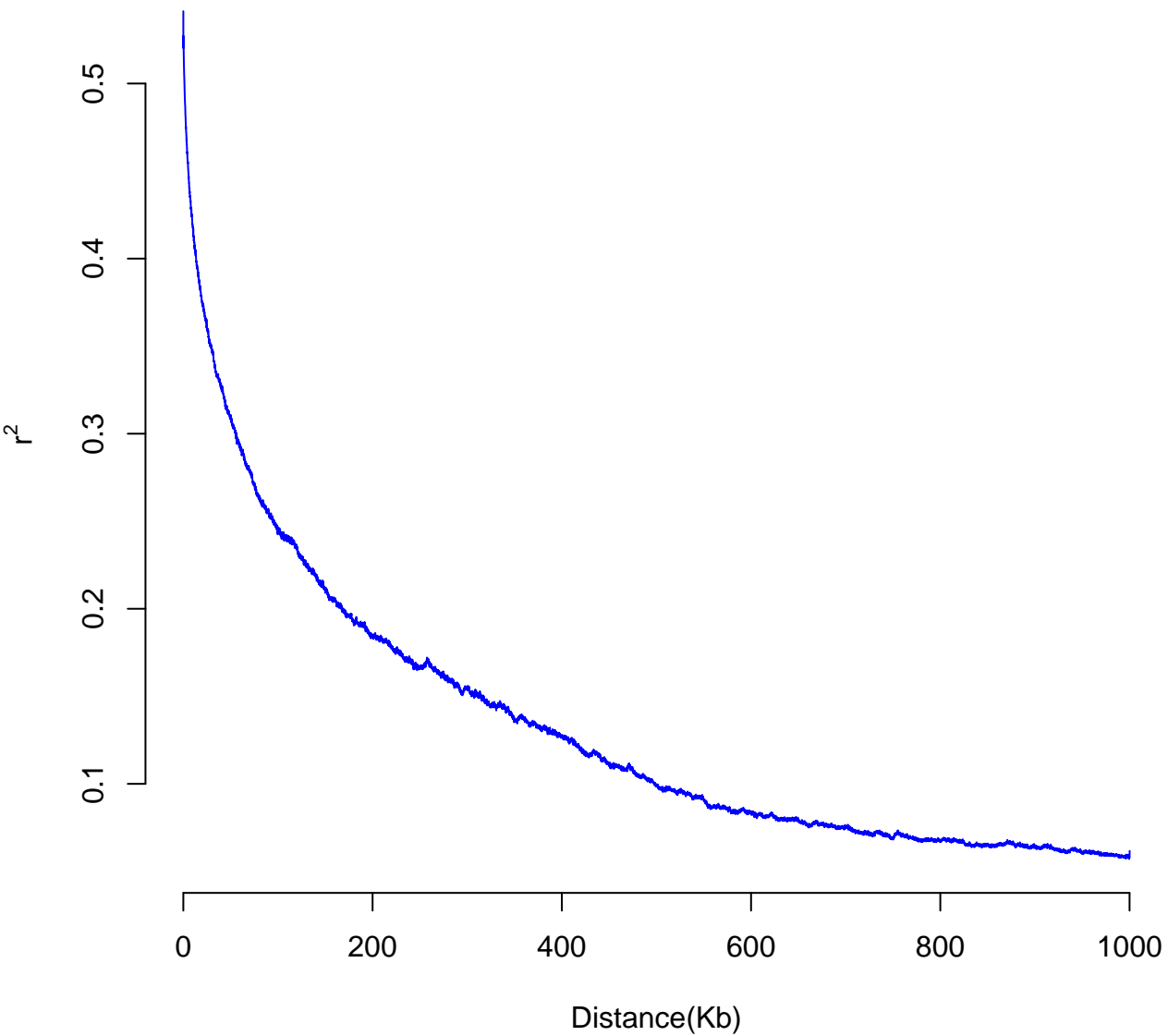

Supplement: Supplementary file 5 — Additional file 5: Figure S2. Genome wide Linkage disequilibrium (LD) decay. [file 12284_2020_424_MOESM5_ESM.pdf]

**a)****Cross entropy**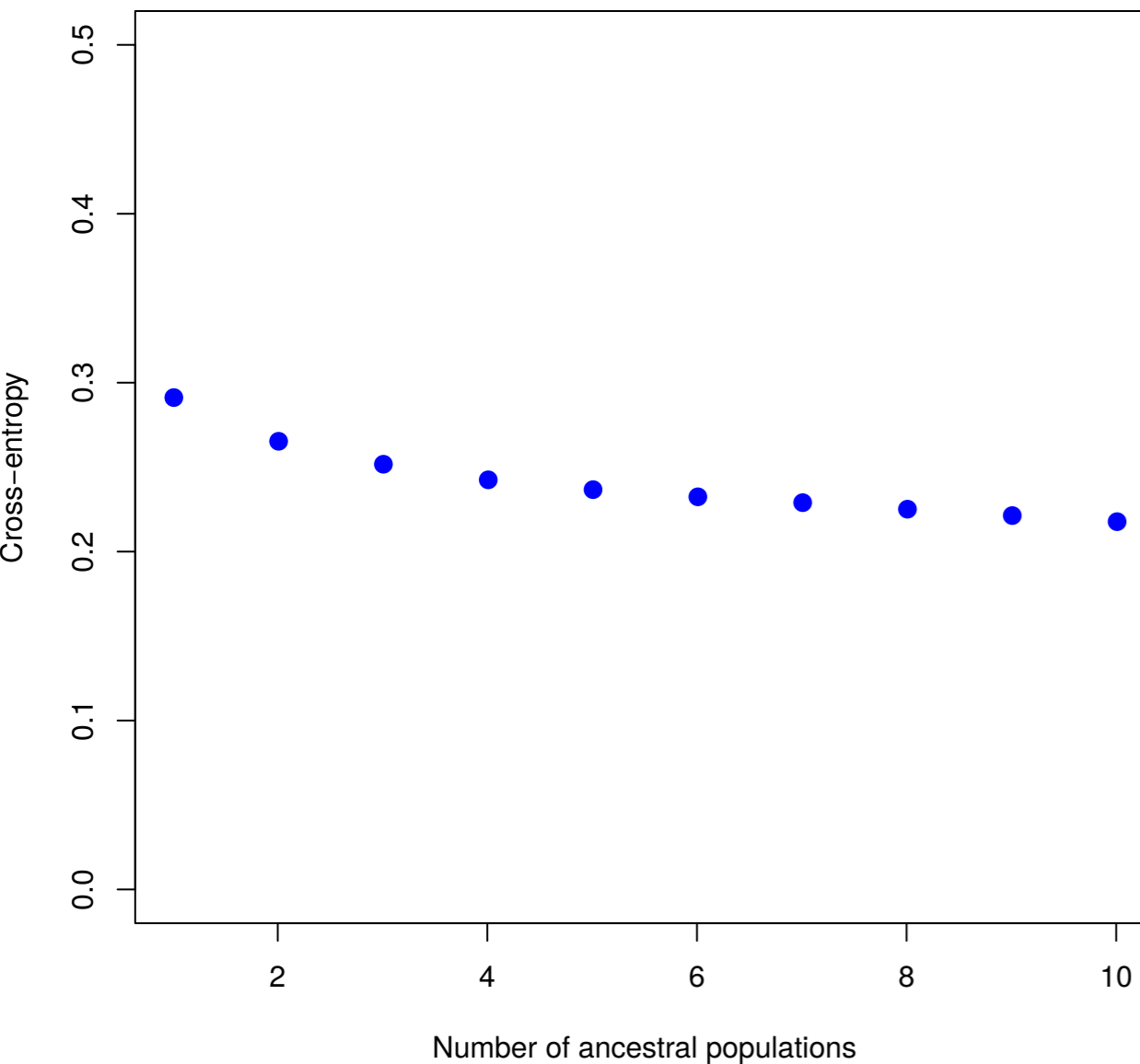**b)****Ancestry matrix**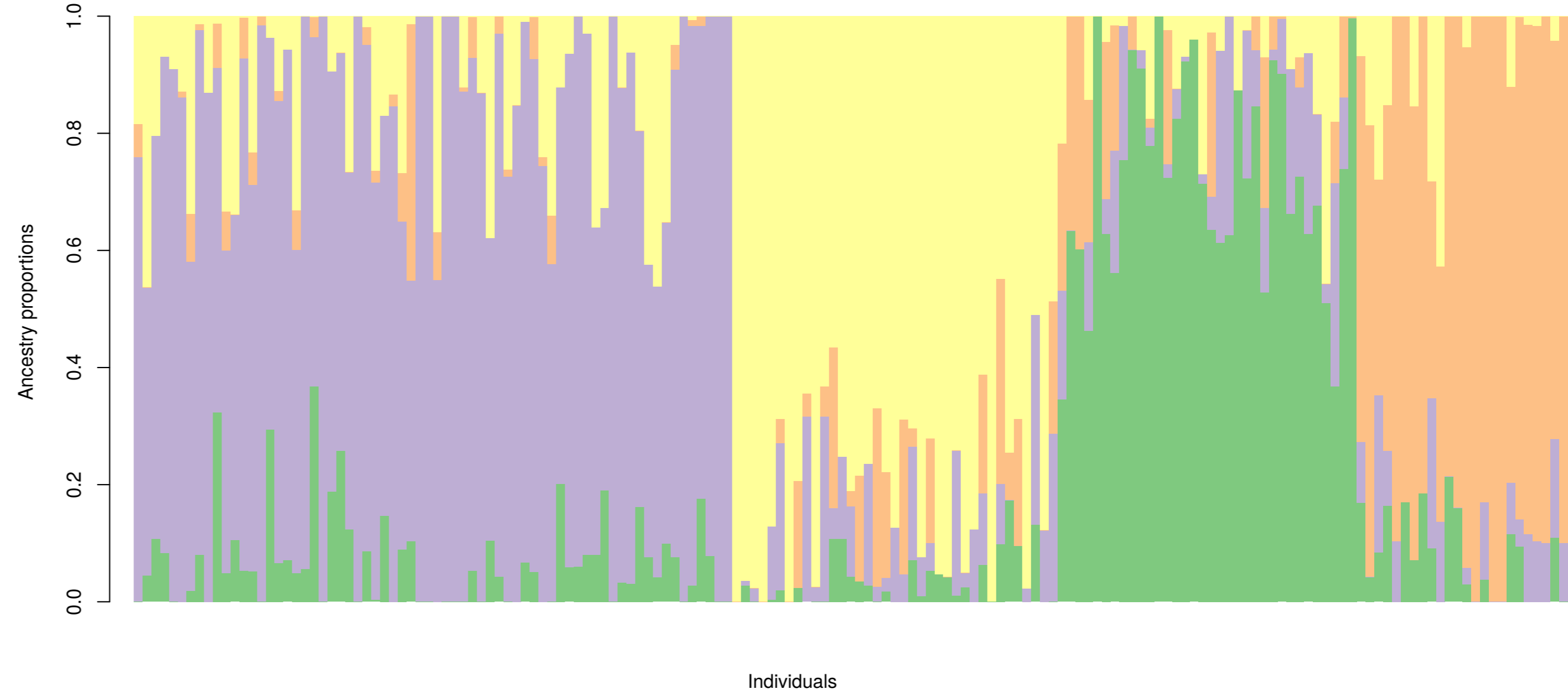

Supplement: Supplementary file 6 — Additional file 6: Figure S3. Structure of the population. (a) evolution of the cross-entropy criterion with increasing K, (b) bar plot of ancestries membership considering K = 4 ancestral population. [file 12284_2020_424_MOESM6_ESM.pdf]

# Geographic repartition of trait values

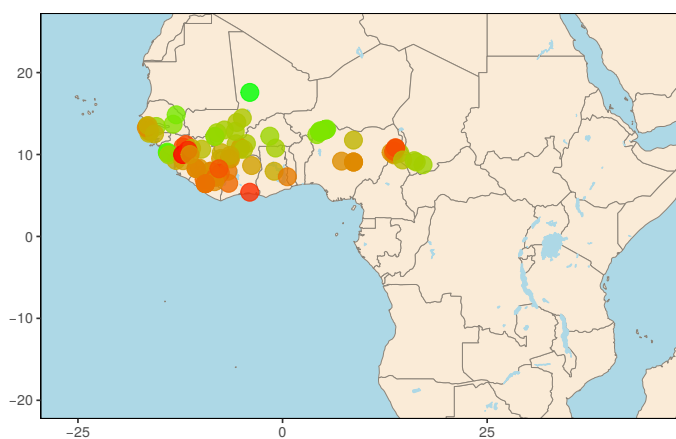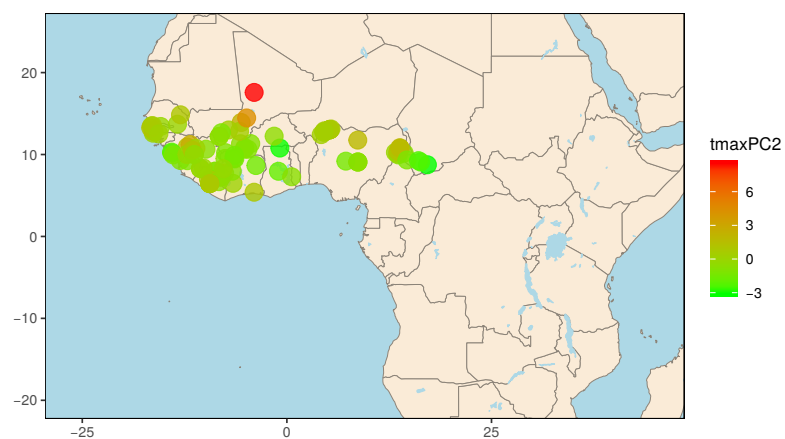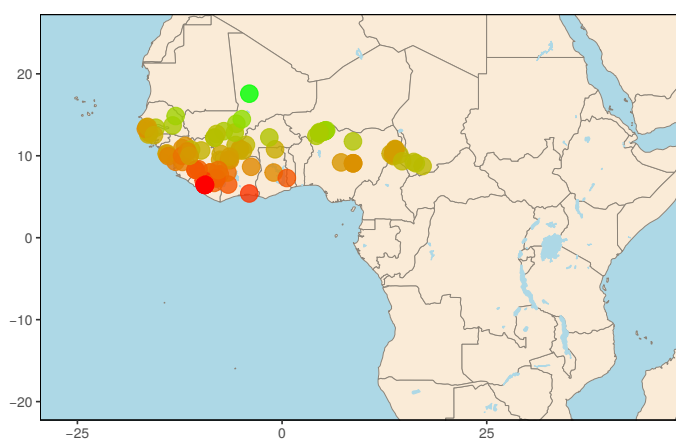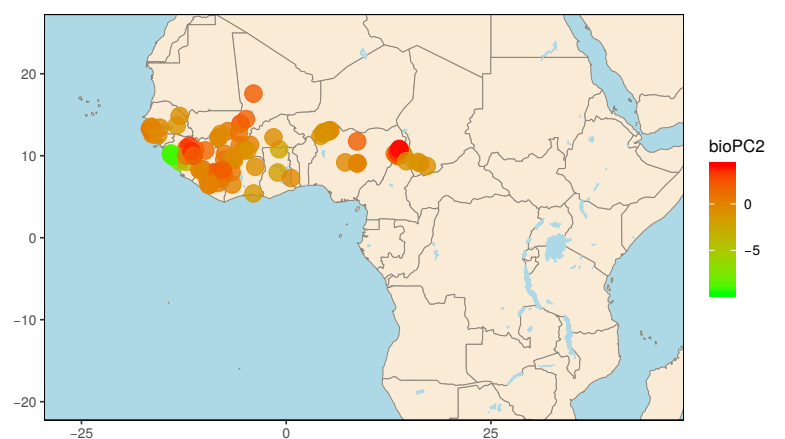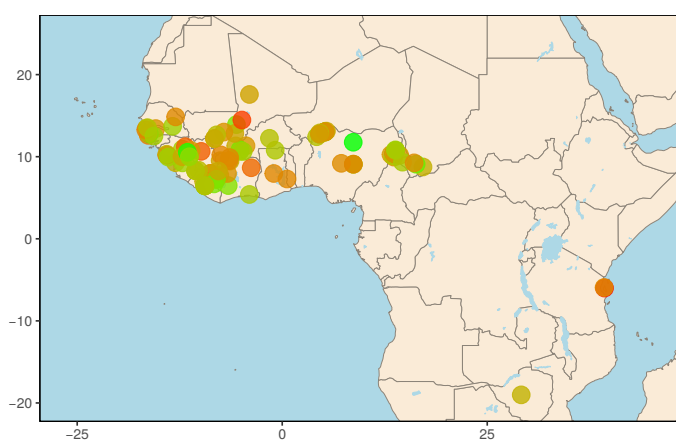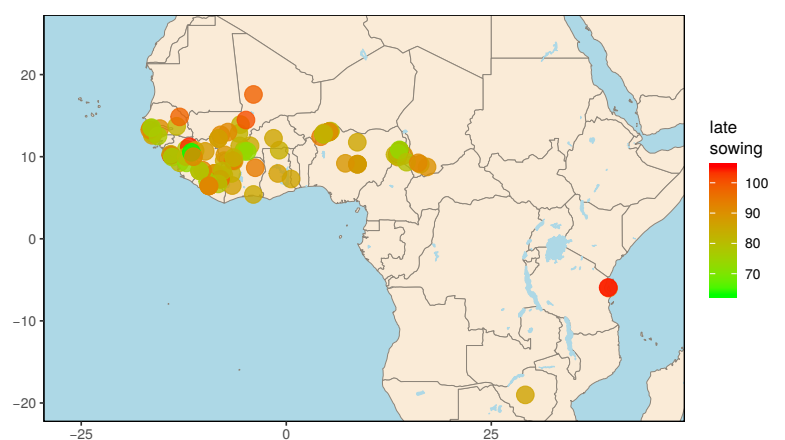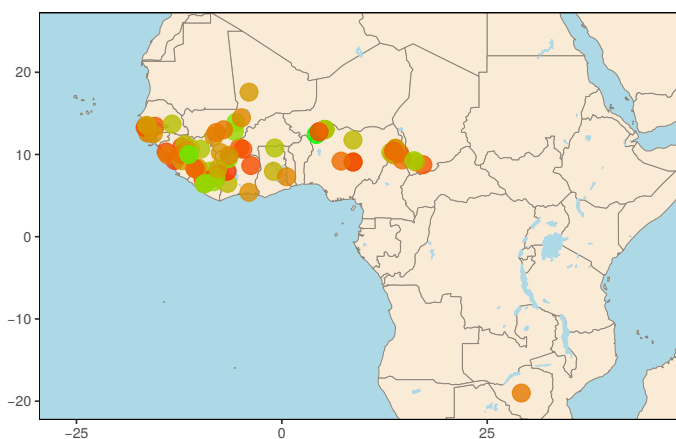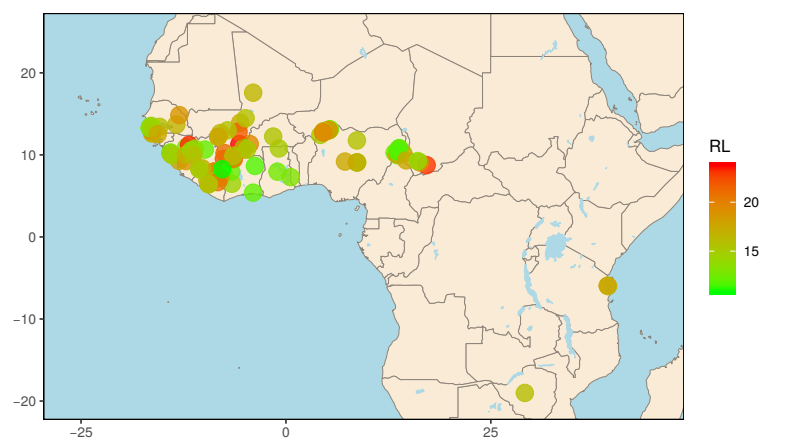

# Geographic repartition of trait values

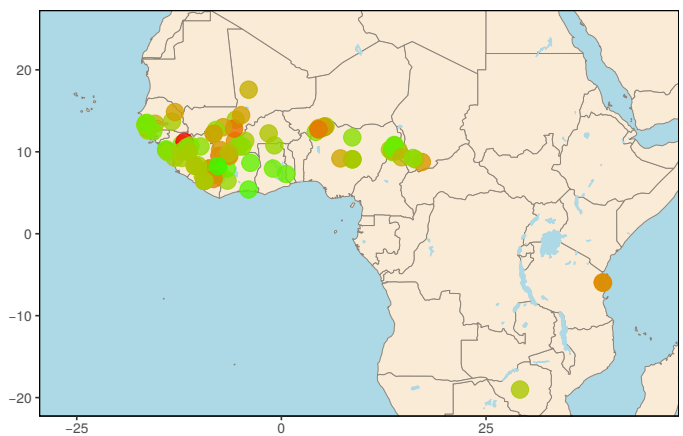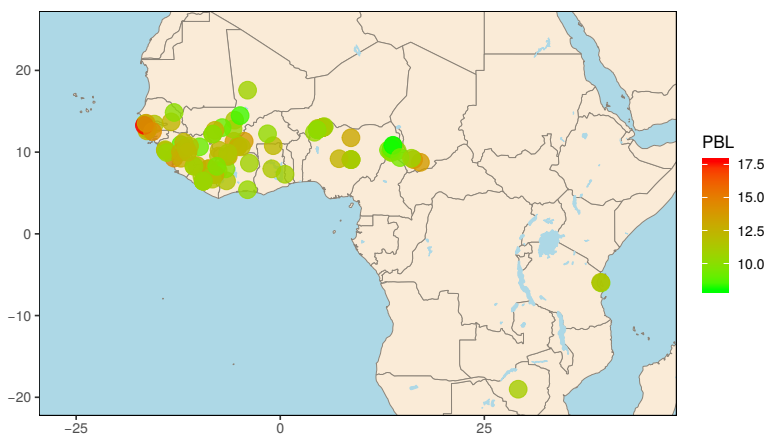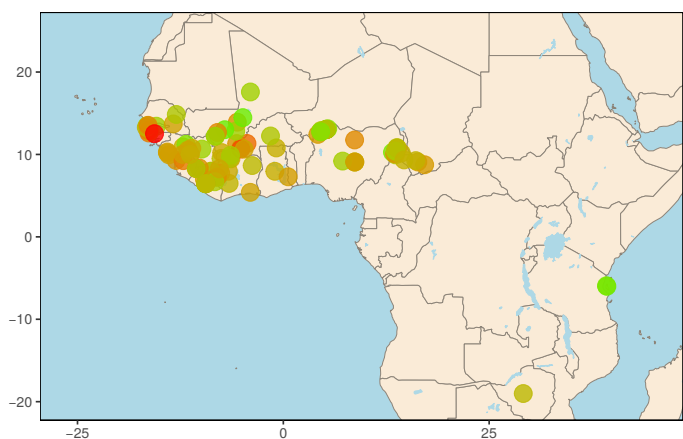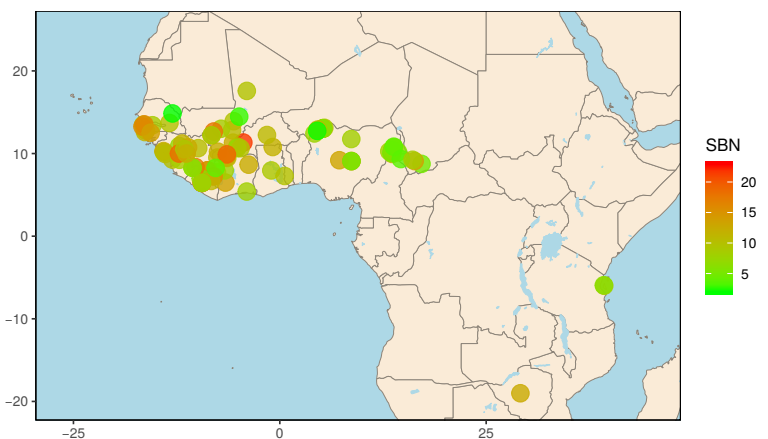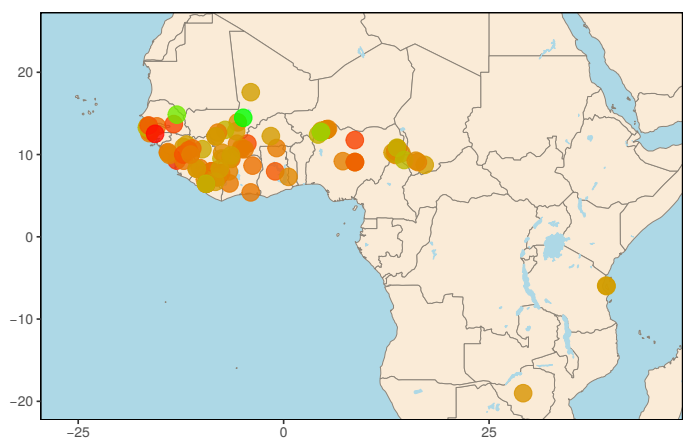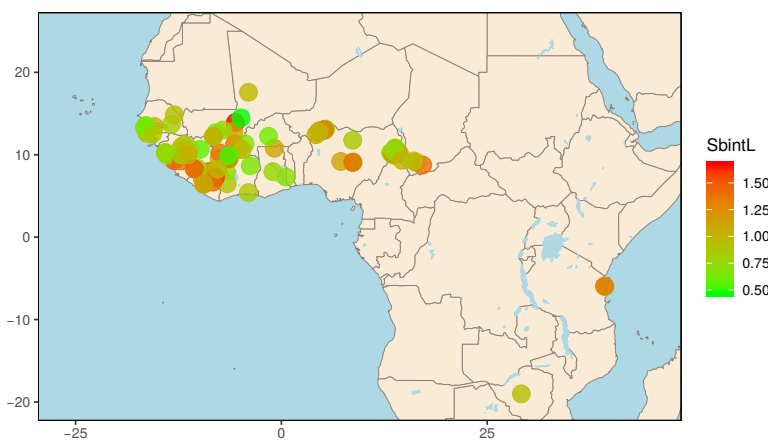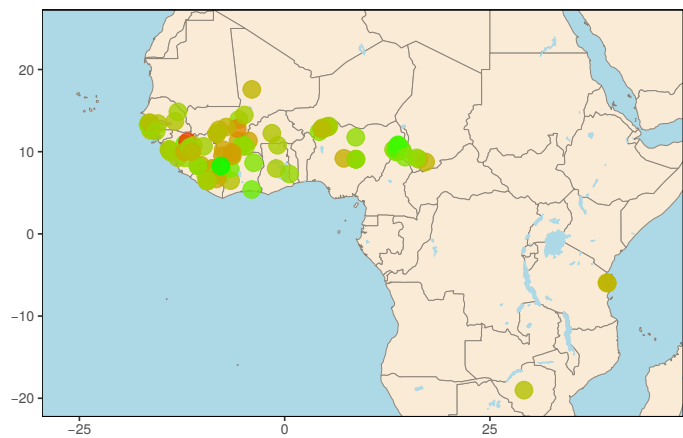

Supplement: Supplementary file 7 — Additional file 7: Figure S4. Geographic distribution of traits. We plotted the mean value of each trait for accessions having sampling location in their passport data. [file 12284_2020_424_MOESM7_ESM.pdf]

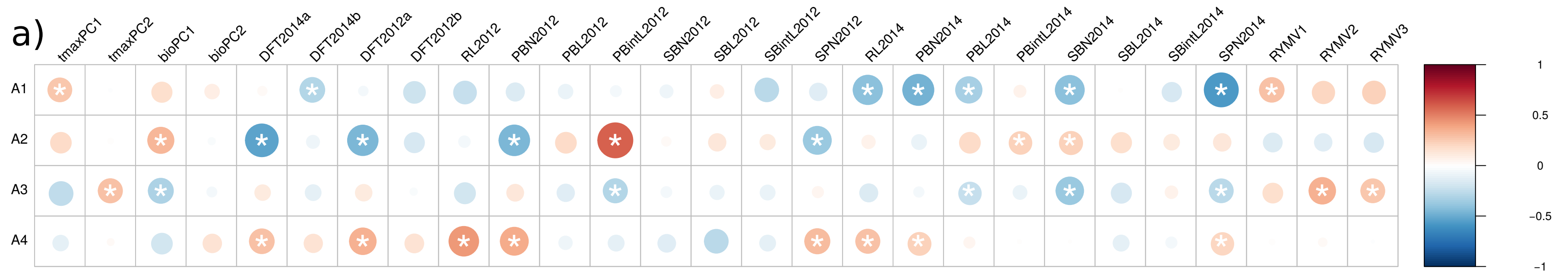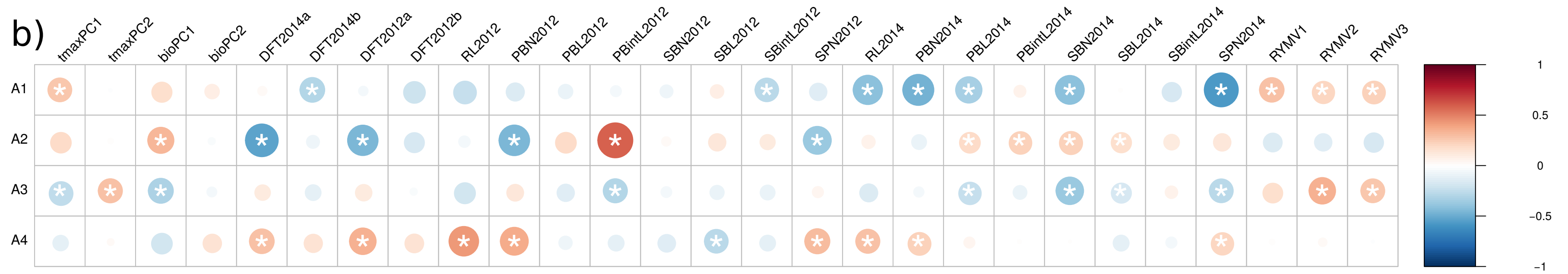

Supplement: Supplementary file 8 — Additional file 8: Figure S5. Correlations between traits and structure. For each ancestry group (A1 to A4), we made a Spearman’s rank correlation test and plotted it as a correlogram. Colors sign the intensity of the correlation and white stars were added when the correlation was significant given the threshold retained, i.e. either a p-value cutoff of 0.01 (a) or a False Discovery Rate of 5% (b). [file 12284_2020_424_MOESM8_ESM.pdf]
