# Supplementary material for: Genome Wide Association Study Pinpoints Key Agronomic QTLs in African Rice Oryza glaberrima
Source: Rice (N Y). 2020 Sep 16;13:66. doi: 10.1186/s12284-020-00424-1 (PMC7494698; doi:10.1186/s12284-020-00424-1)

# Early sowing

## emma

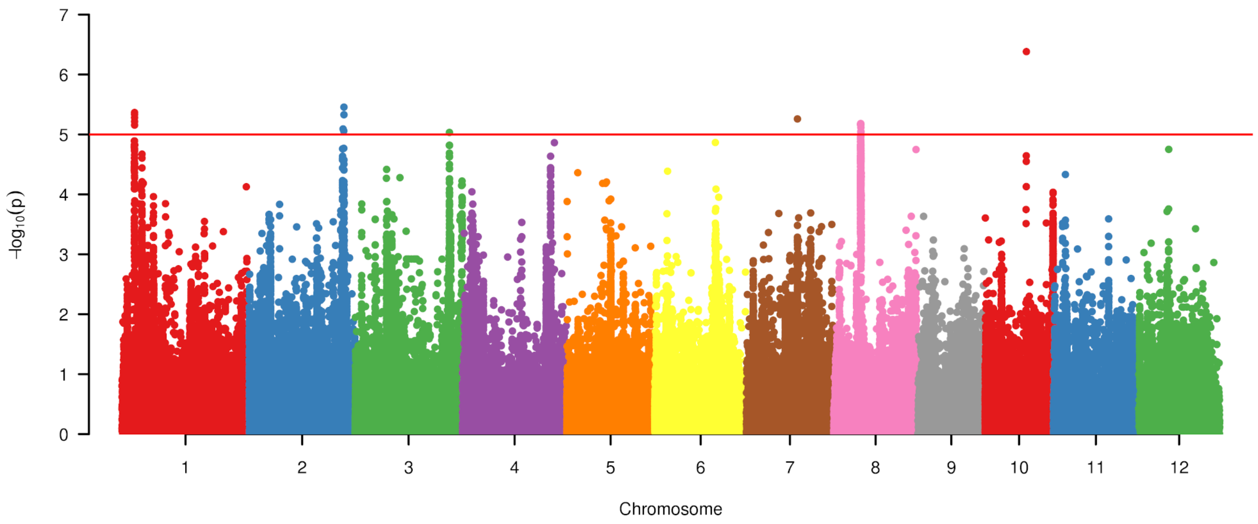


## gapit.mlm

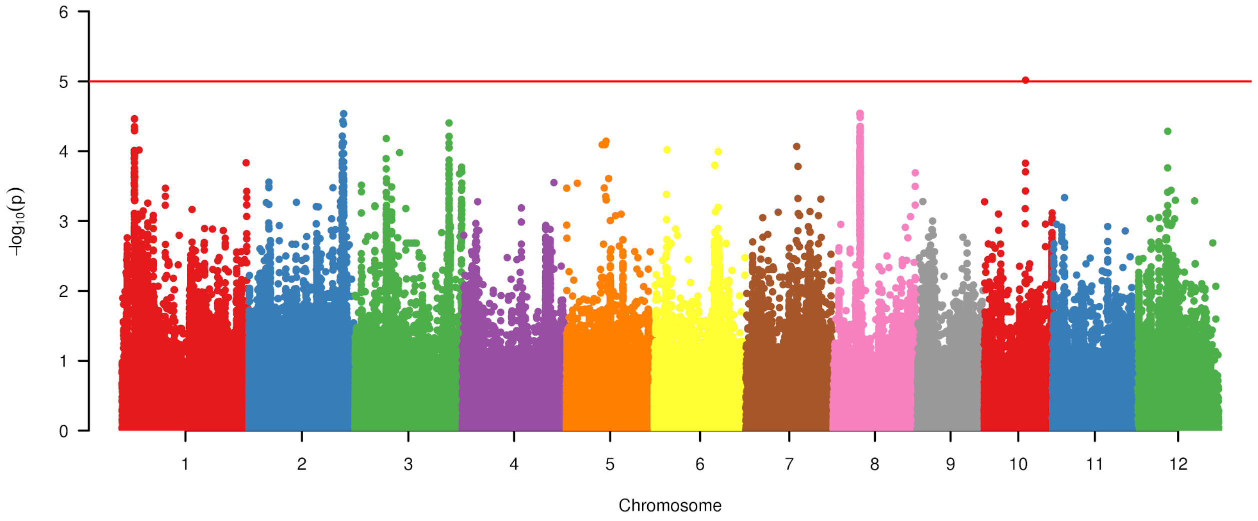


## lfmm

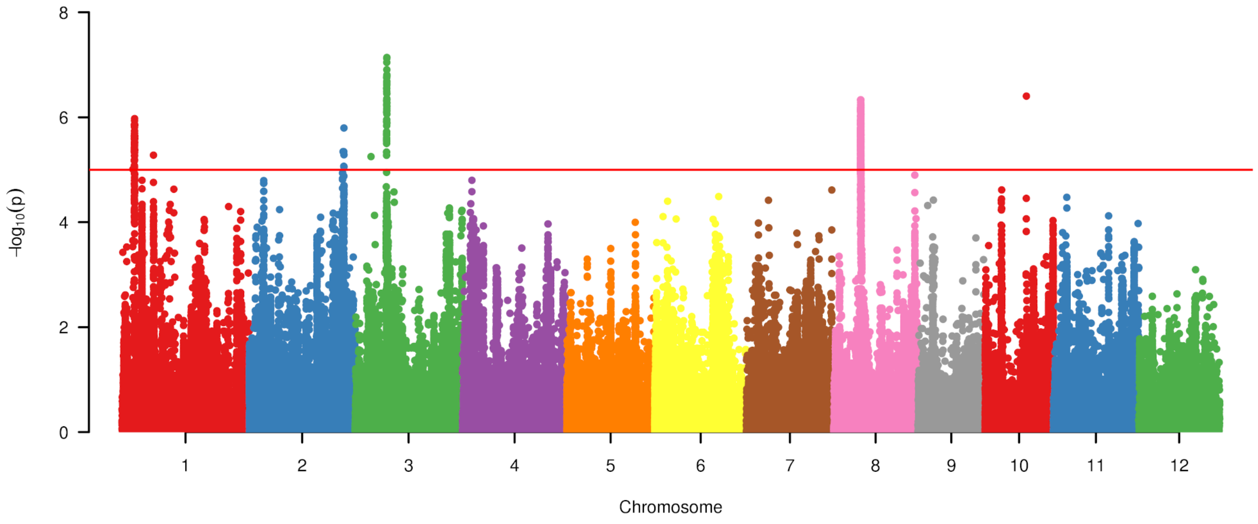

# Late sowing

## emma

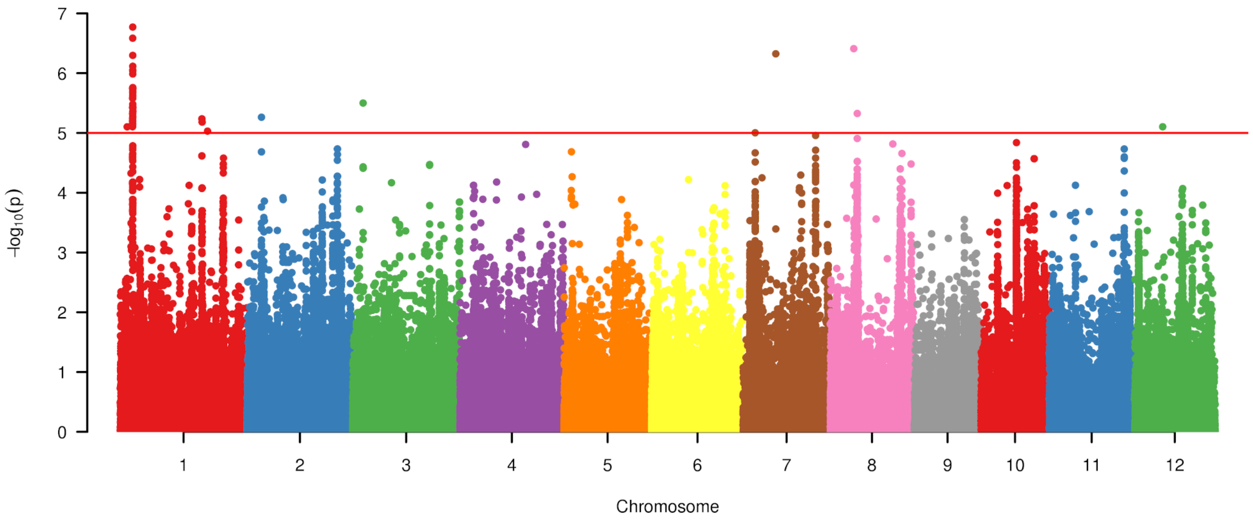


## gapit.mlm

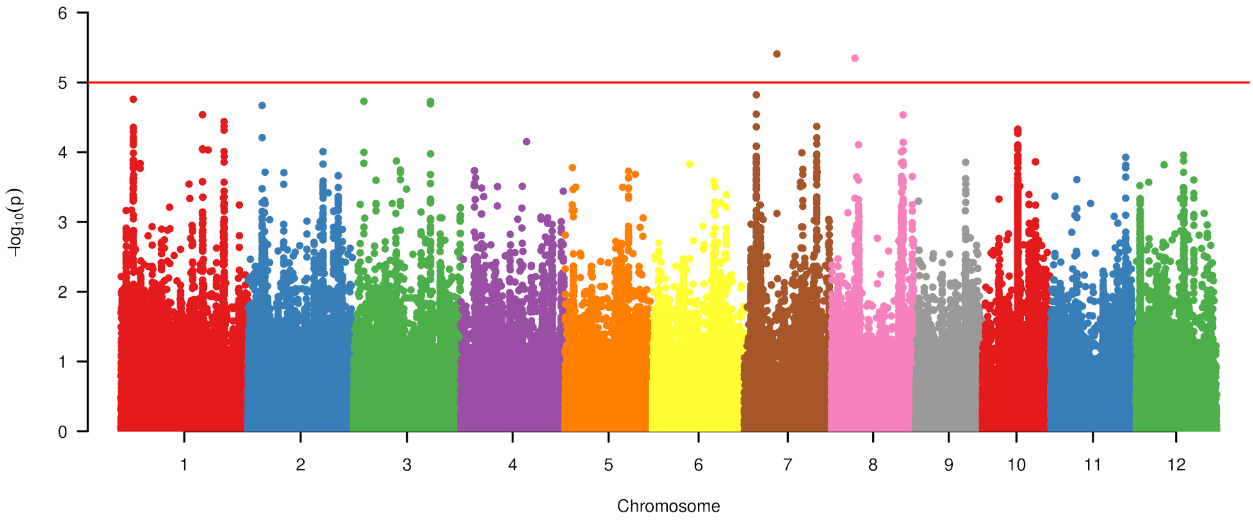


## lfmm

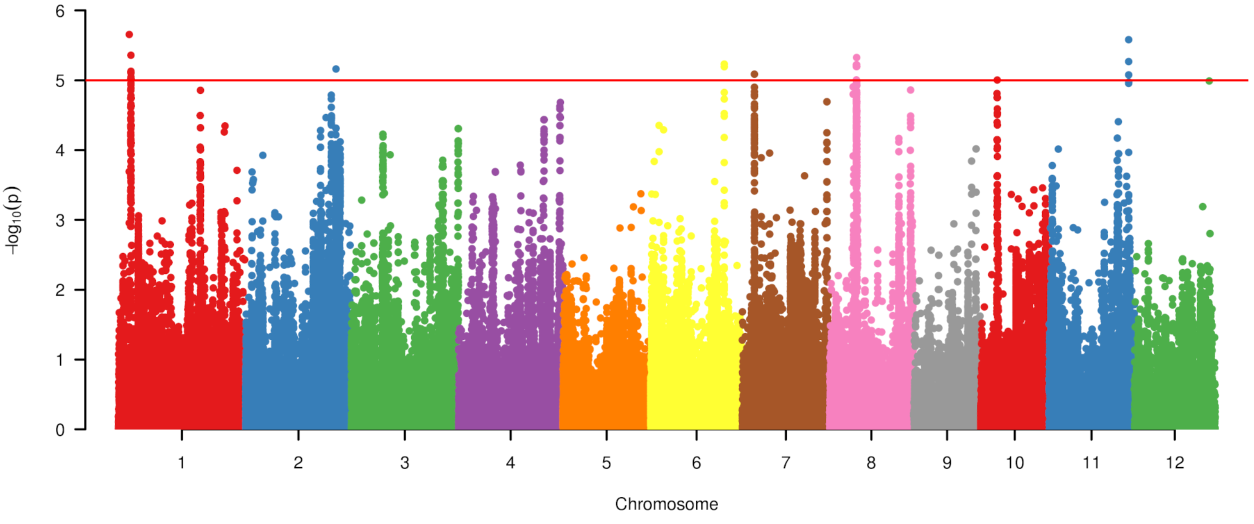

# Rachis length (RL)

emma

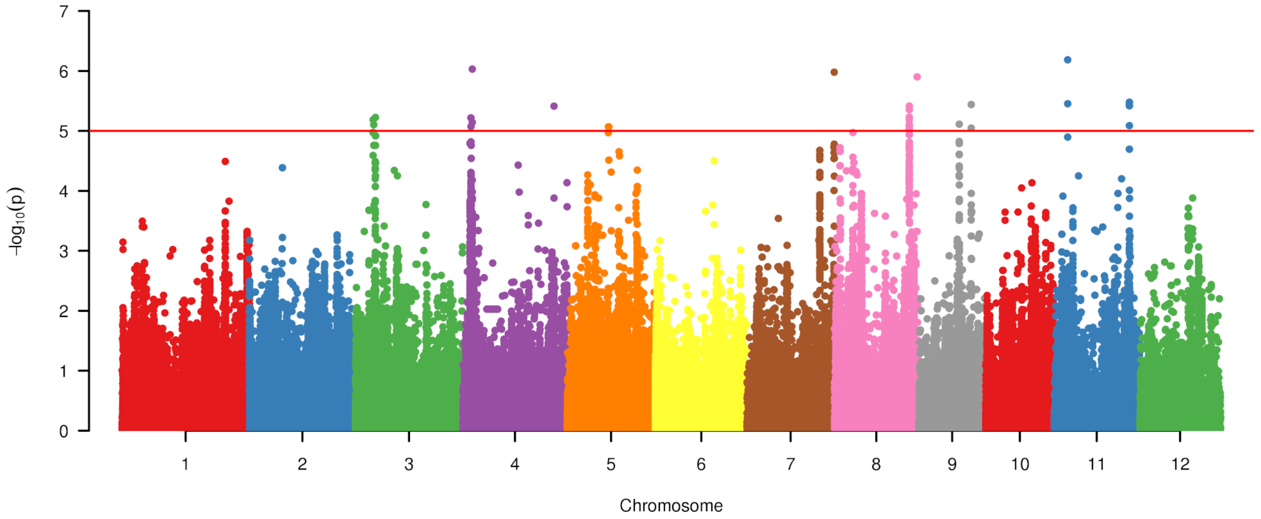


gapit.mlm

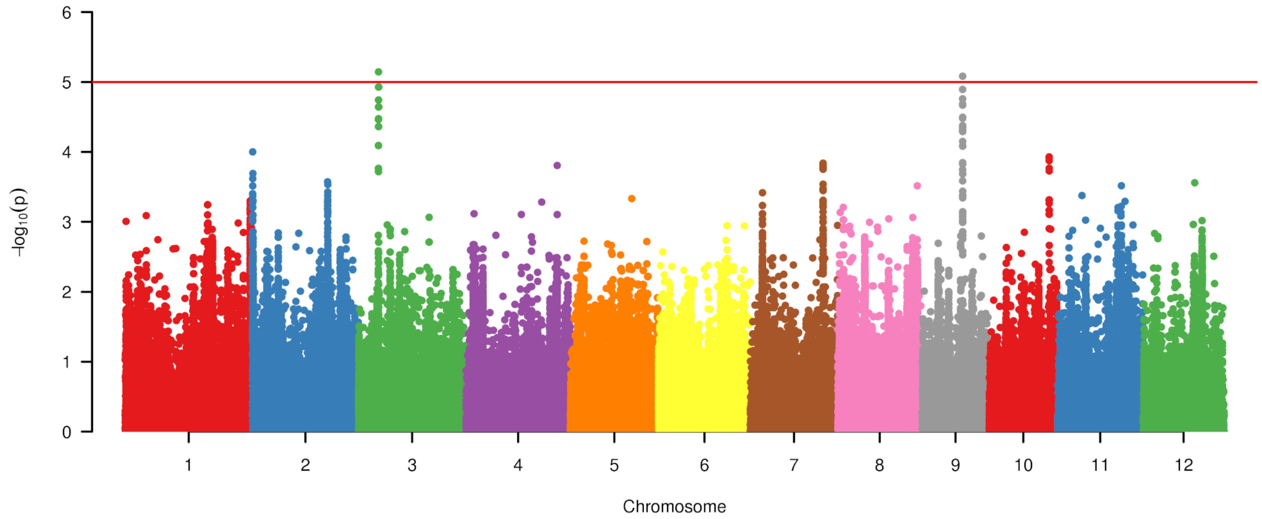


lfmm

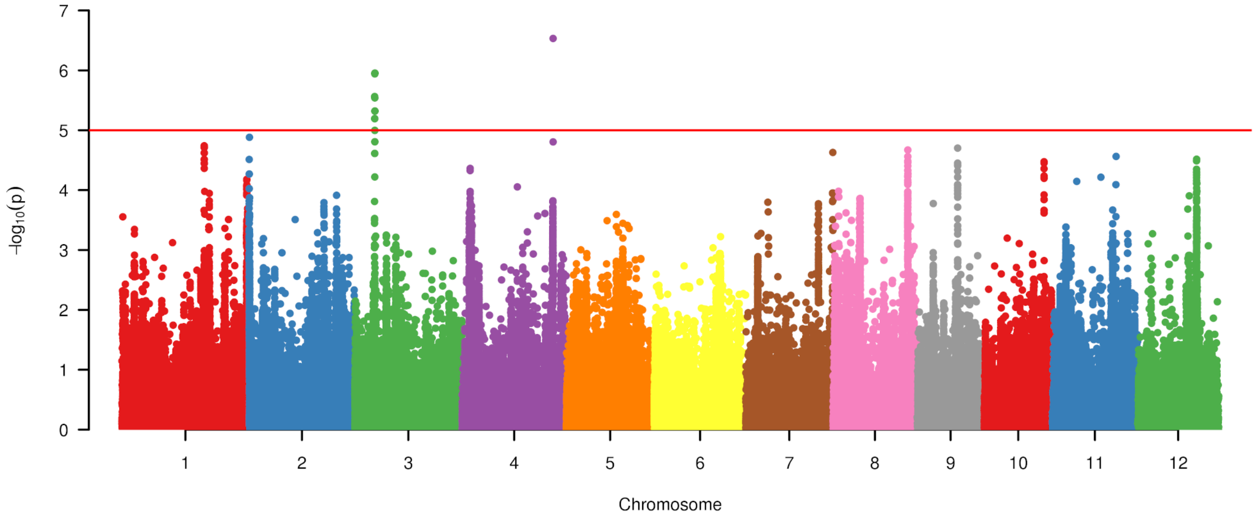

# Spikelet number (SpN)

emma

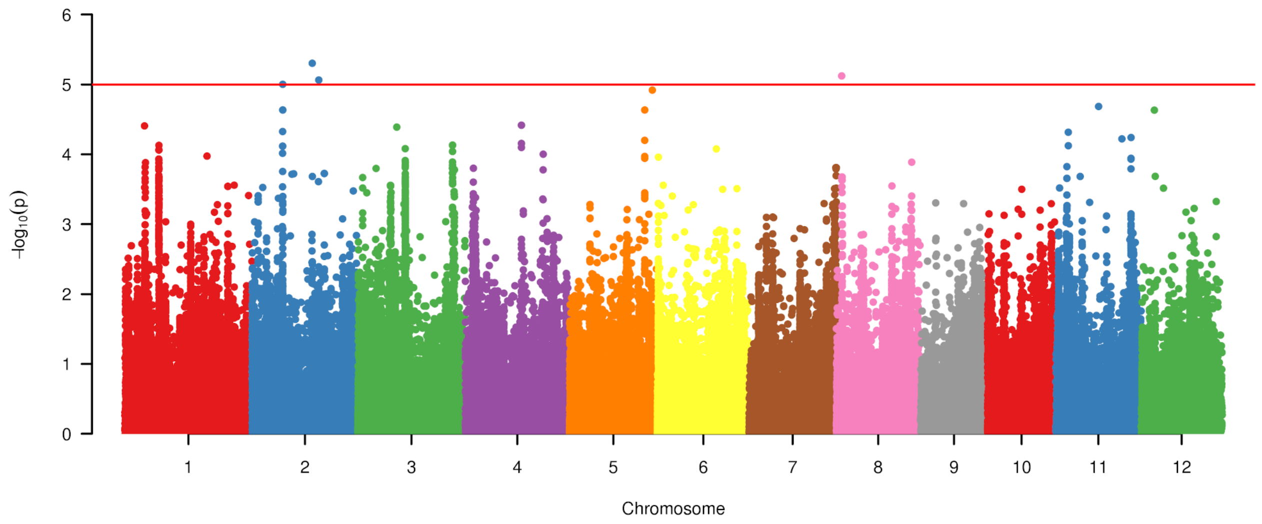


gapit.mlm

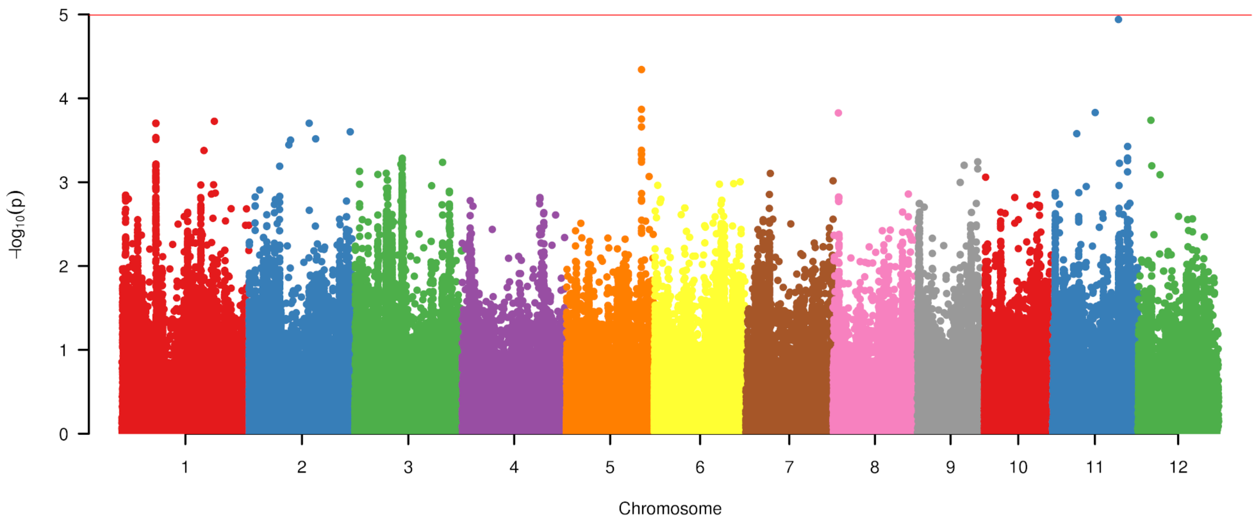


lfmm

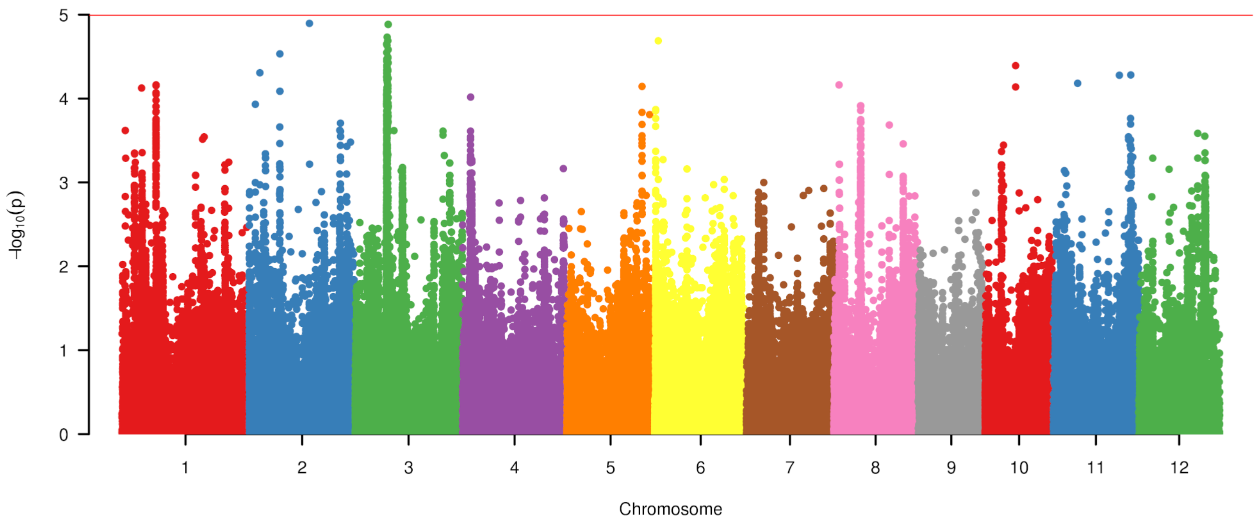

# Primary branch number (PBN)

emma

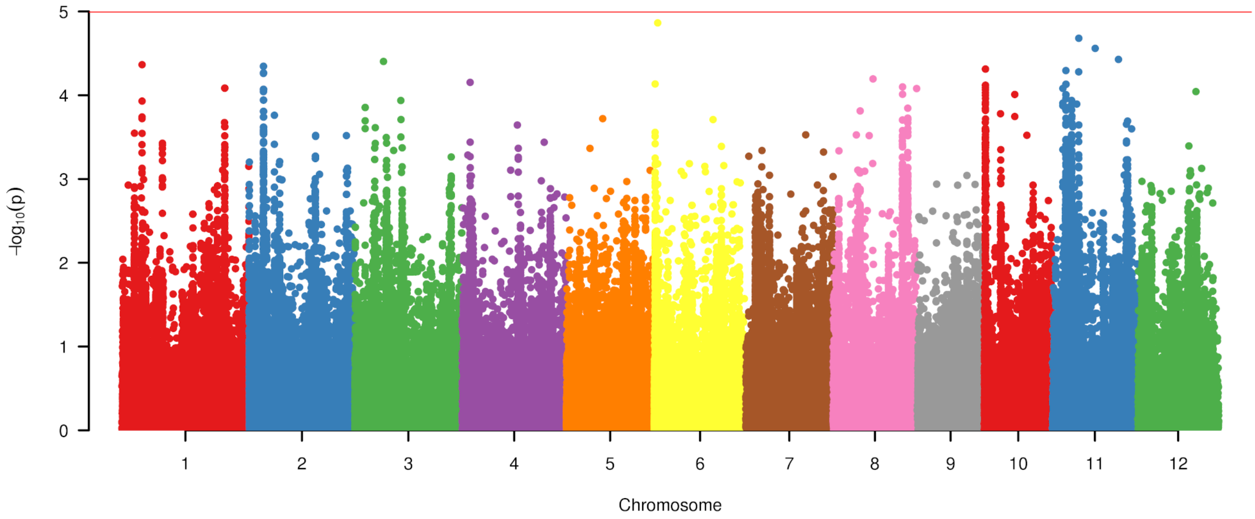


gapit.mlm

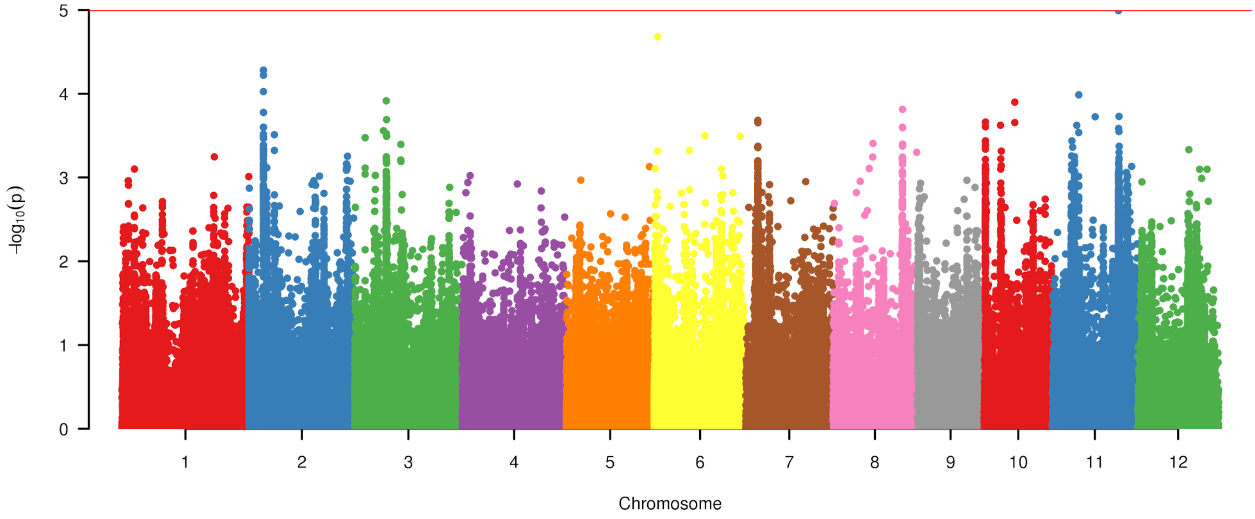


lfmm

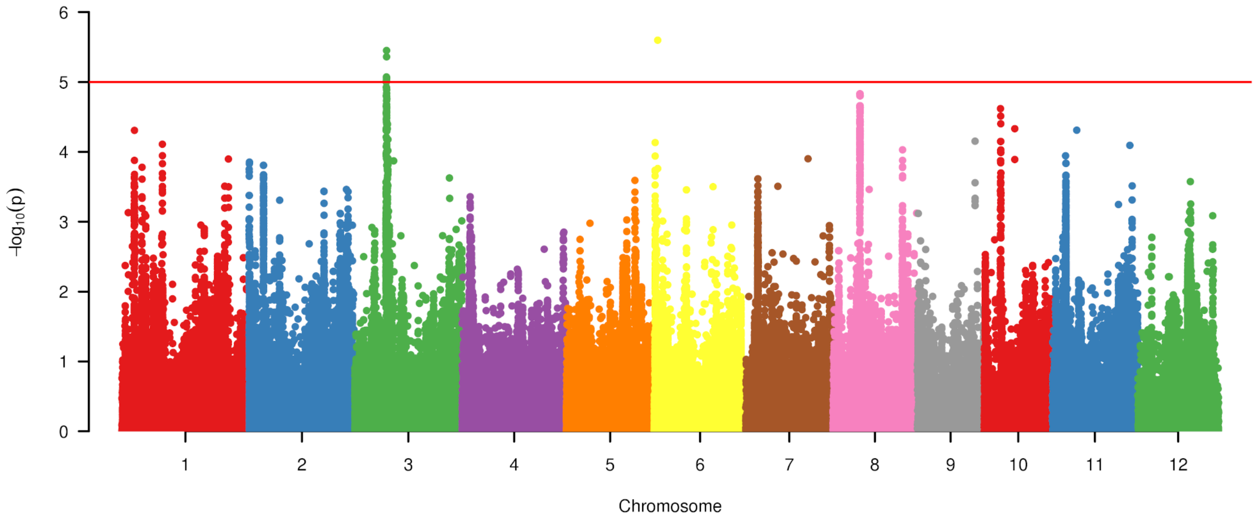

# Secondary branch number (SBN)

## emma

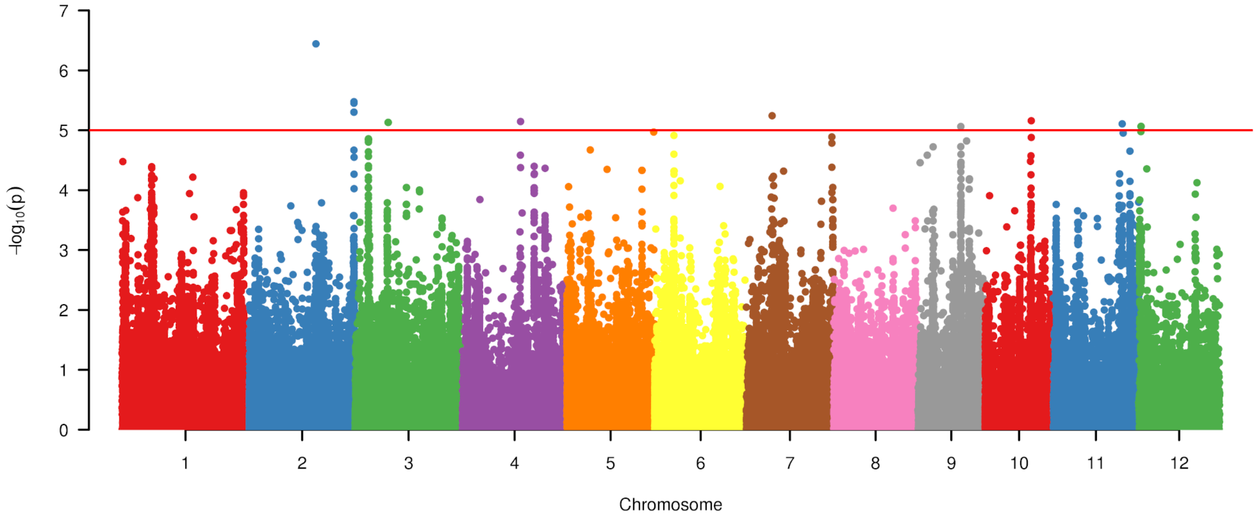


## gapit.mlm

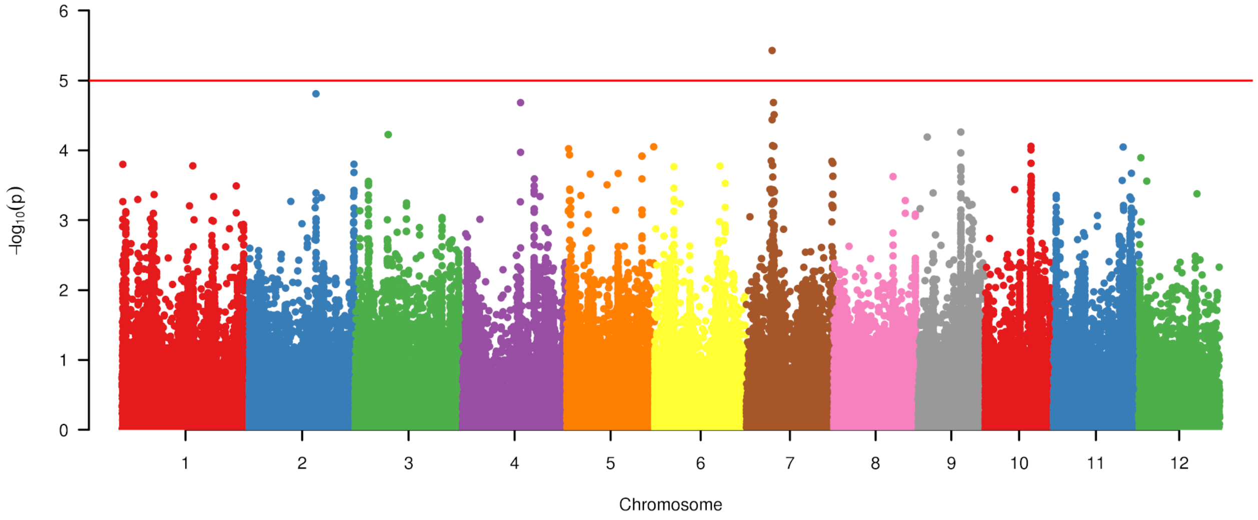


## lfmm

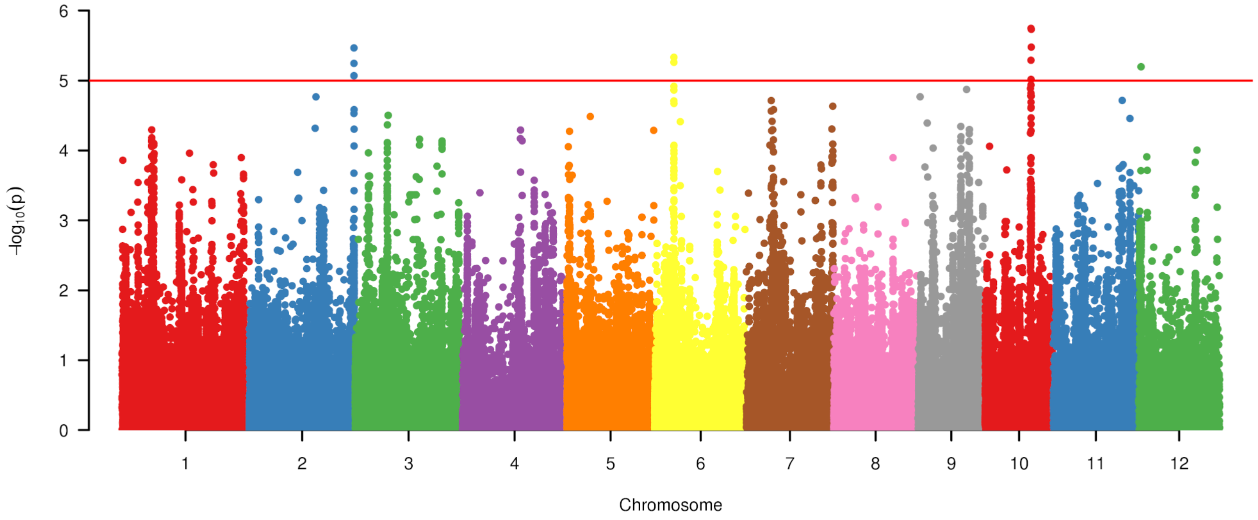

# Primary branch average length (PBL)

emma

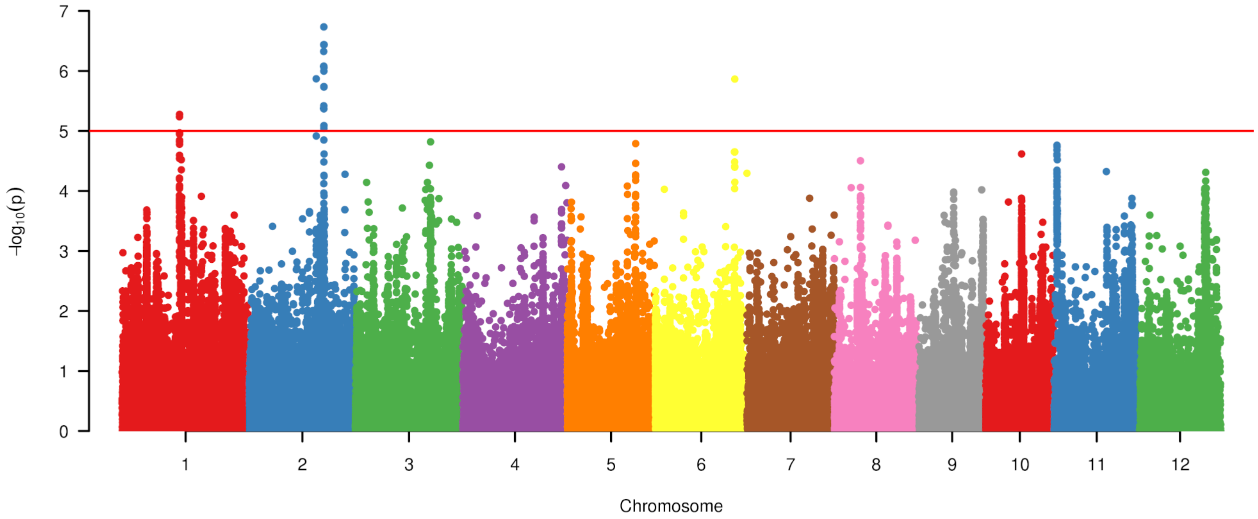


gapit.mlm

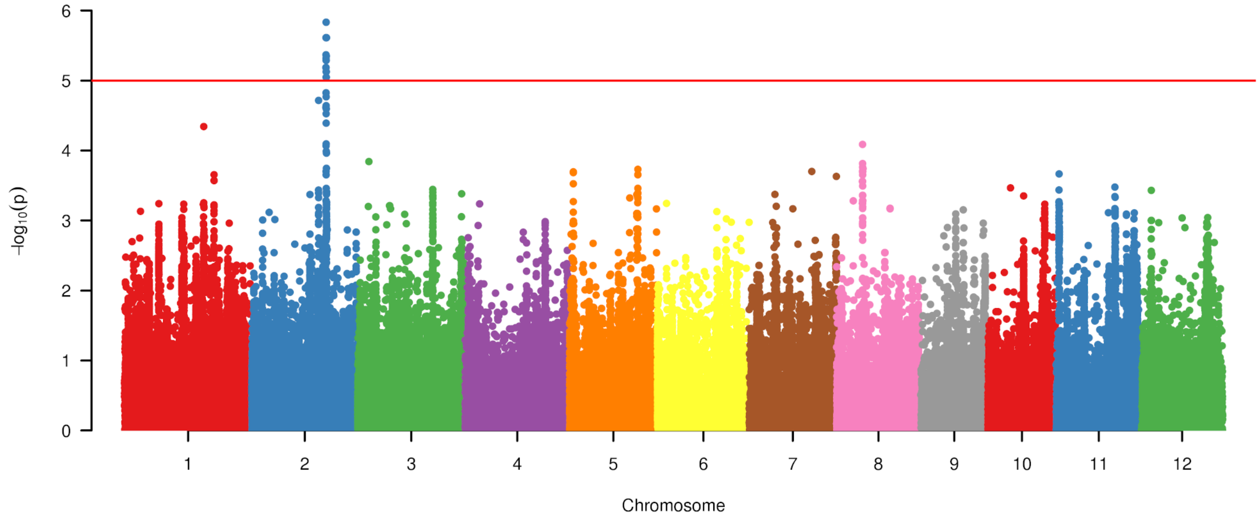


lfmm

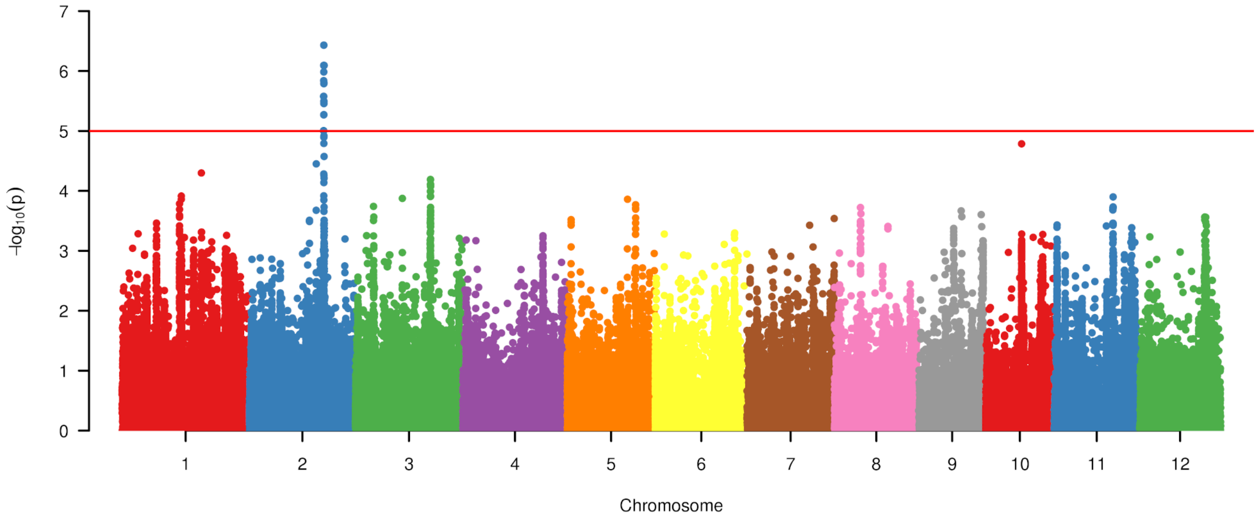

# Secondary branch average length (SBL)

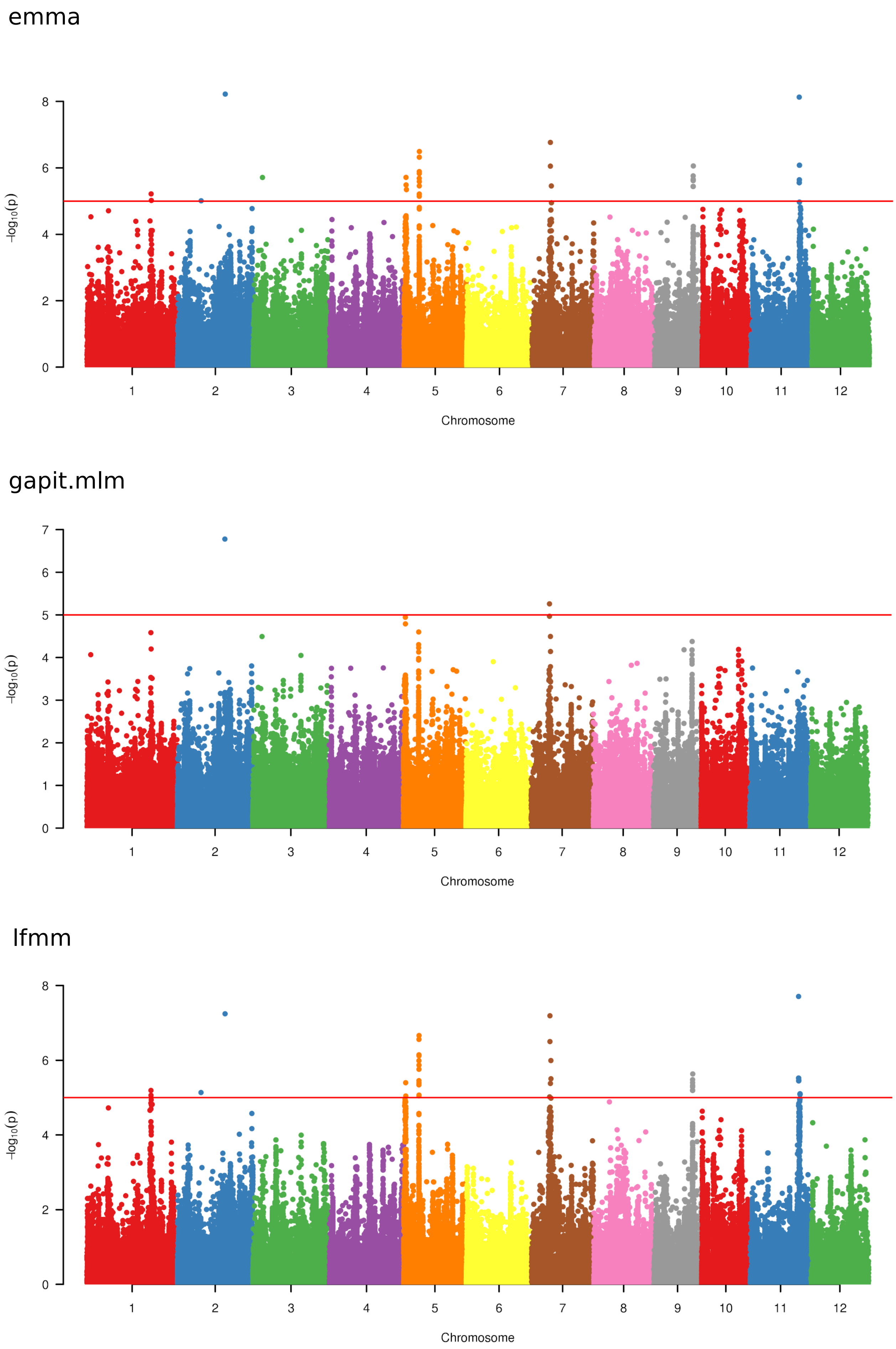

# Primary branch internode average length (PBintL)

emma

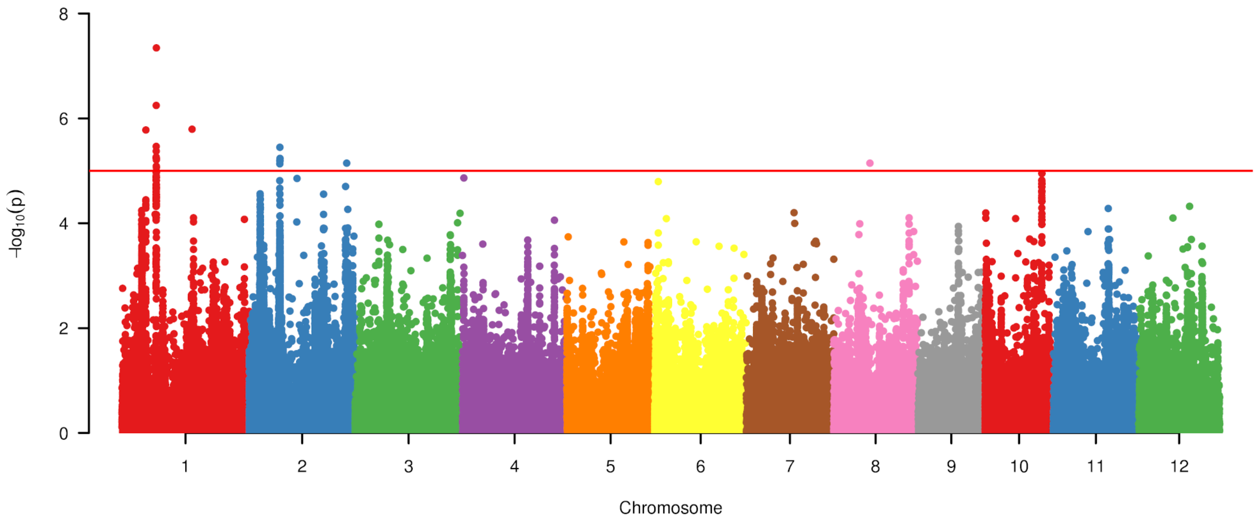


gapit.mlm

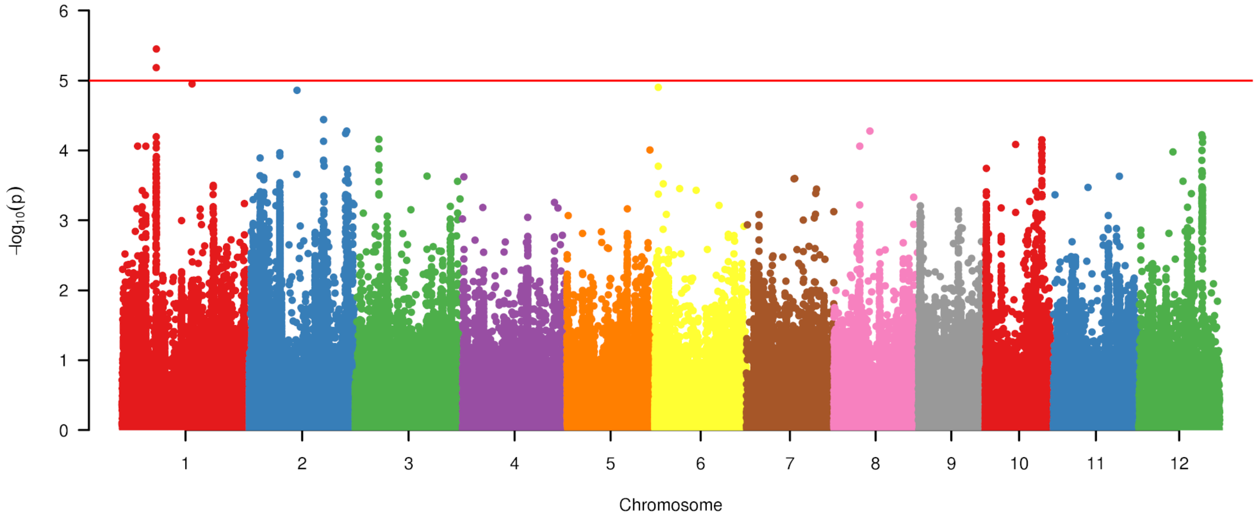


lfmm

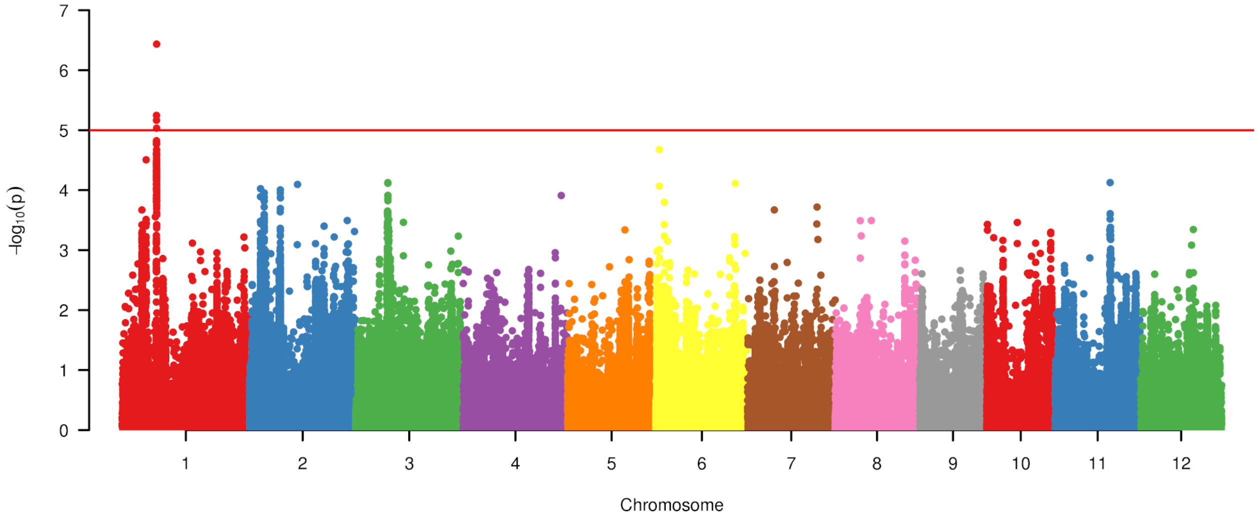

# Secondary branch internode average length (SBintL)

emma

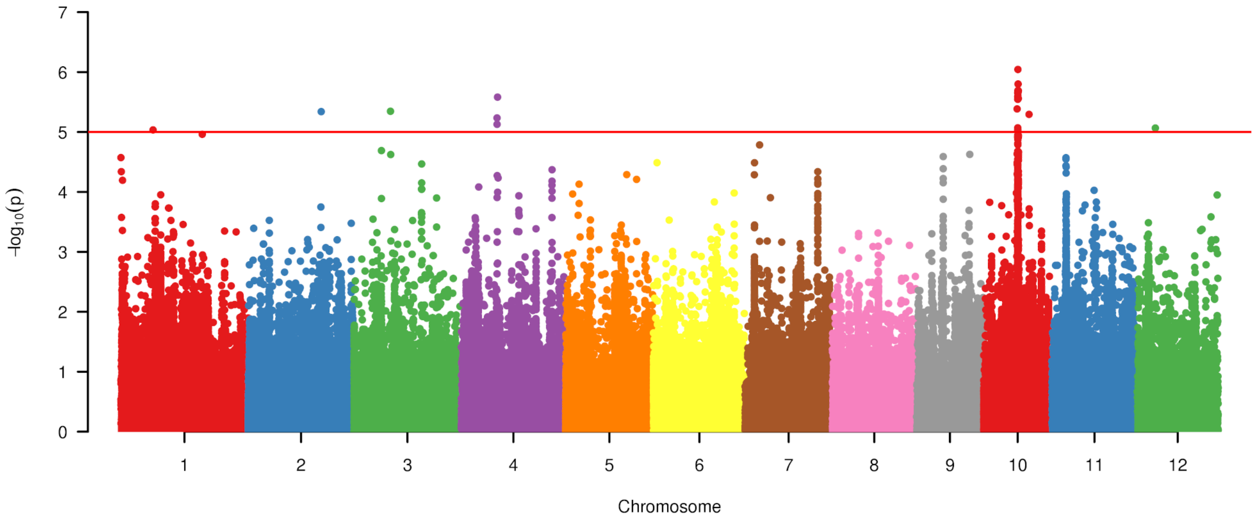


gapit.mlm

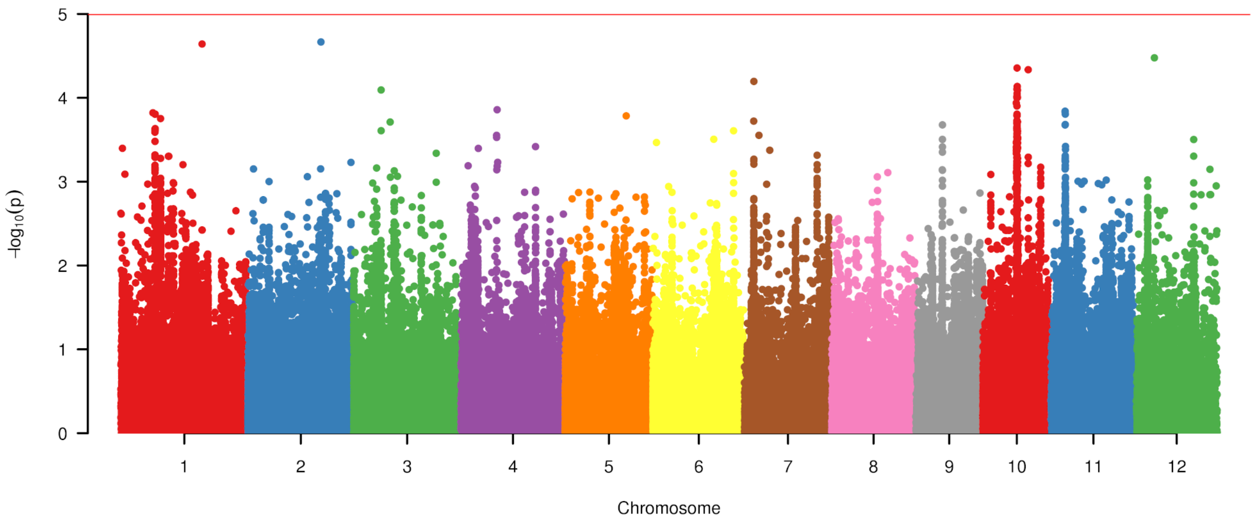


lfmm

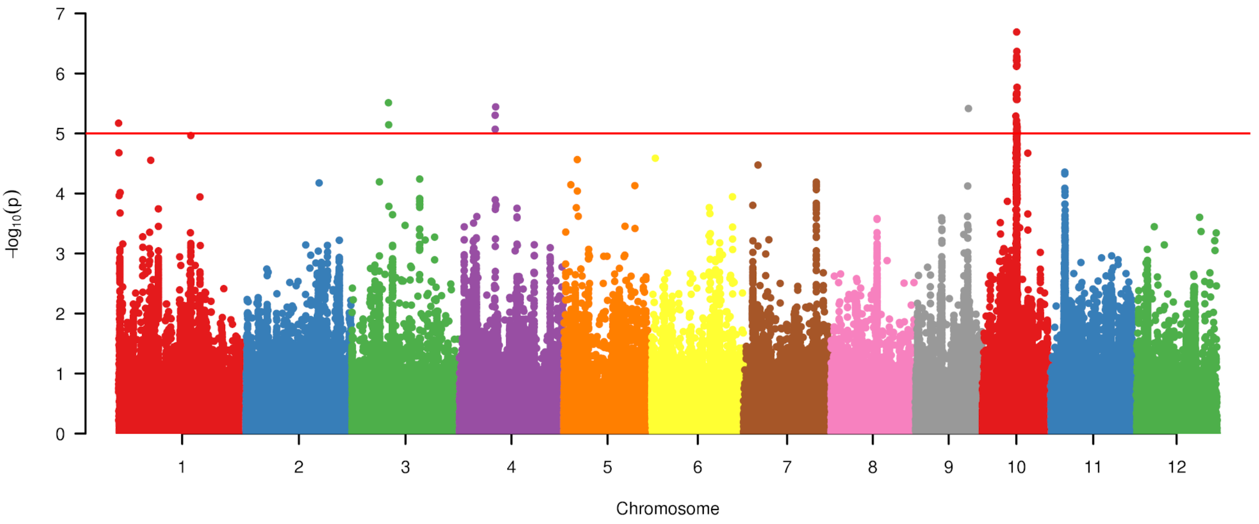

# Resistance to Rice yellow mottle virus (RYMV)

emma

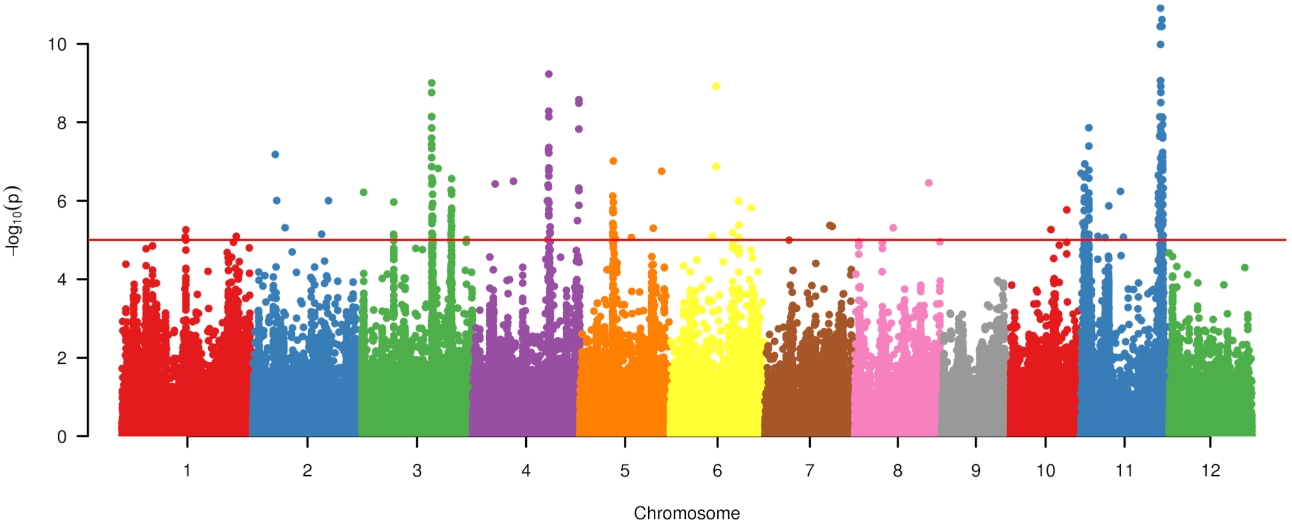


gapit.mlm

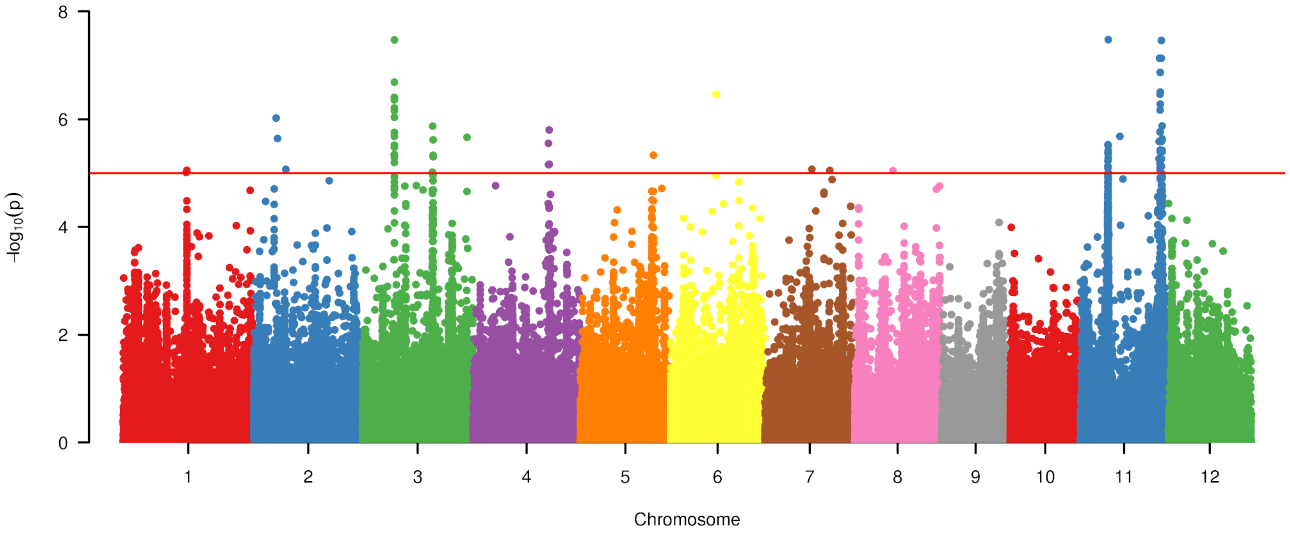


lfmm

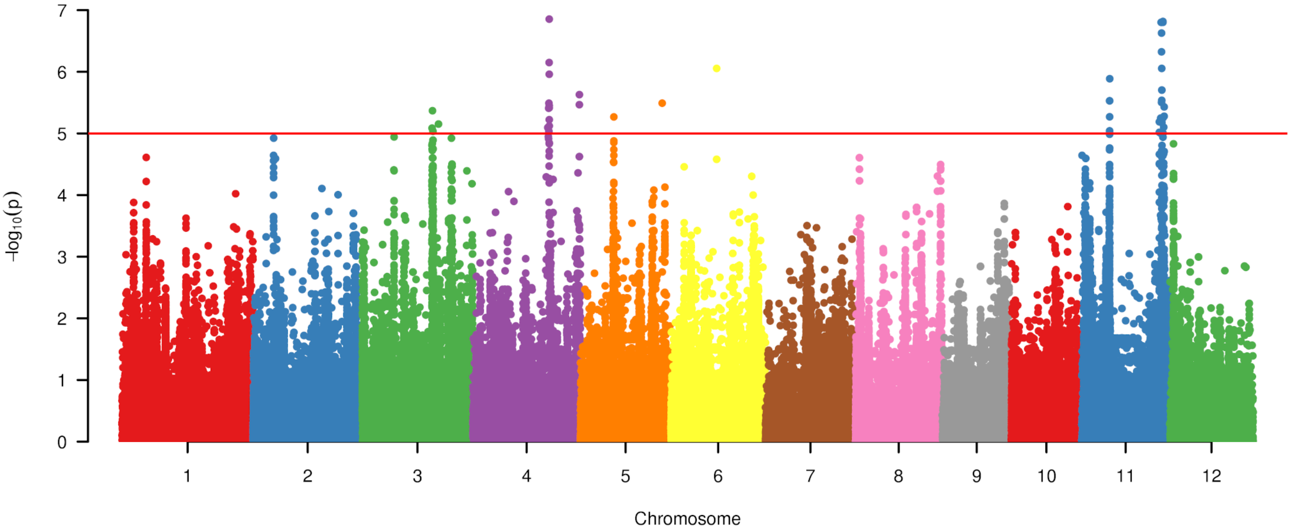

# bioPC1

emma

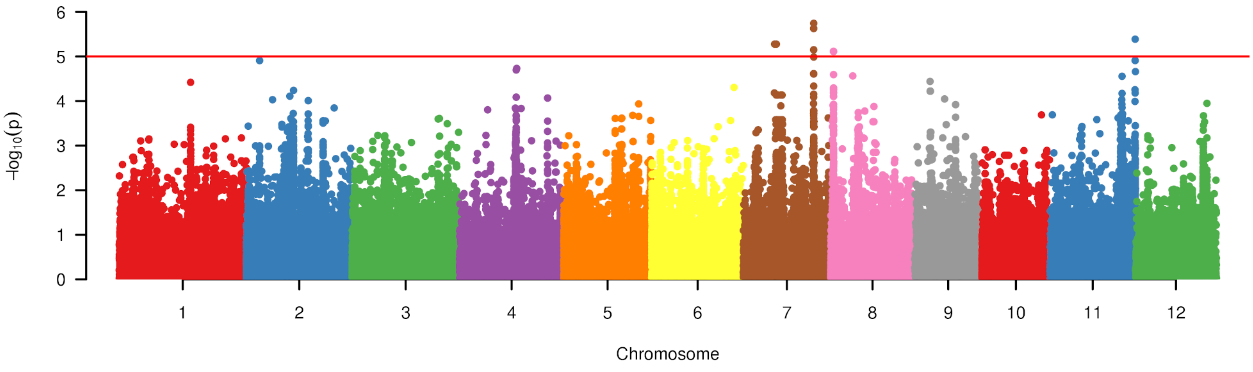


gapit.mlm

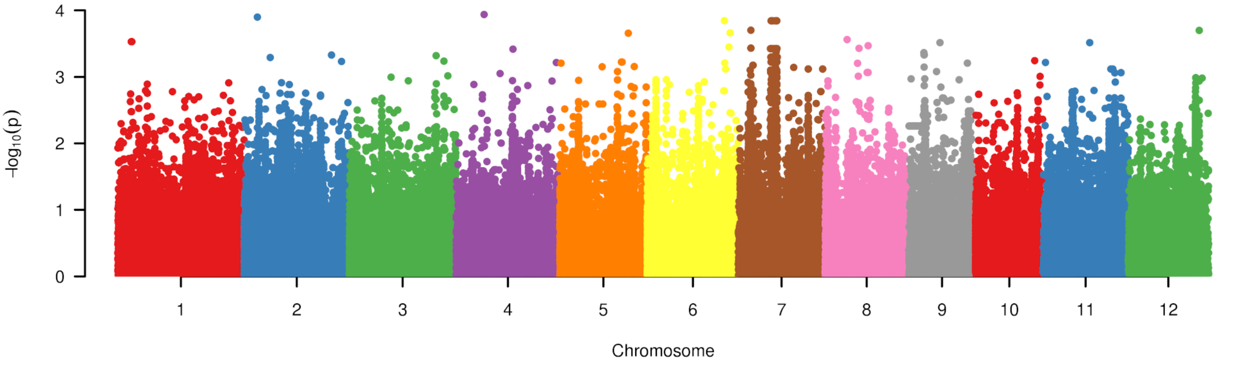


lfmm

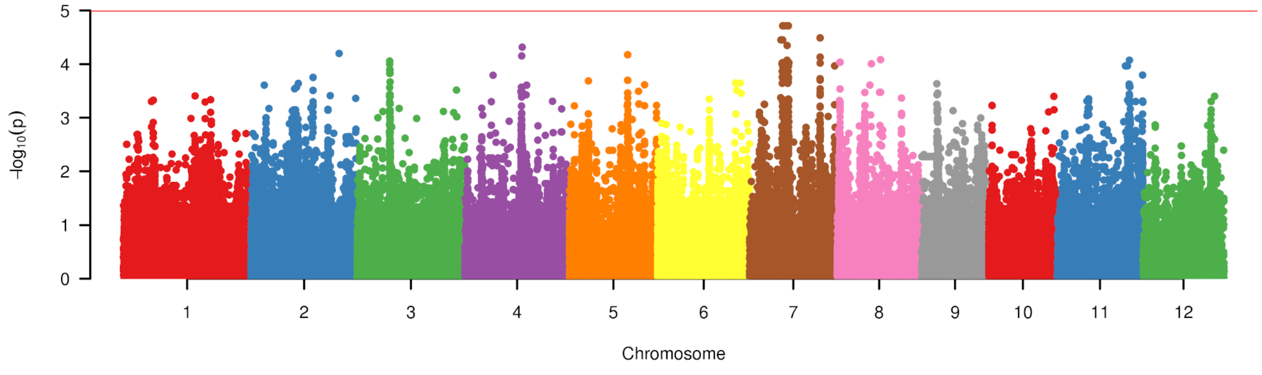

# bioPC2

## emma

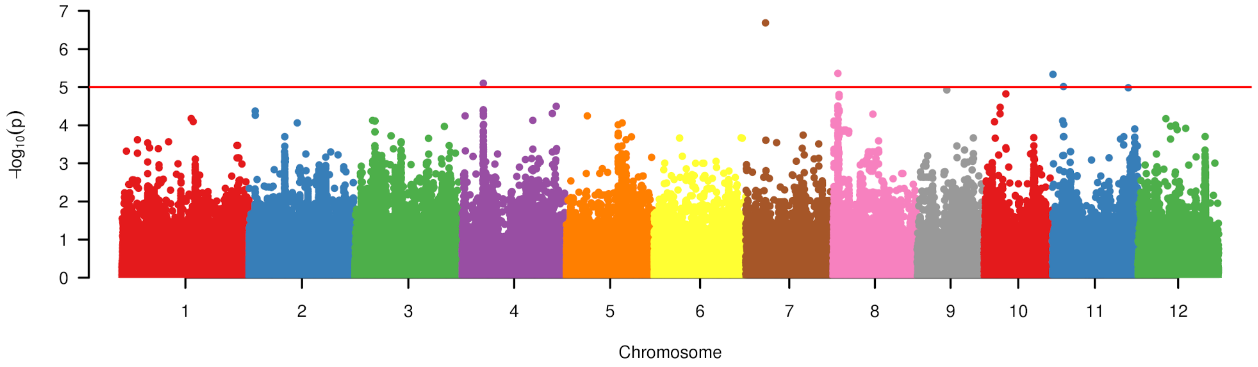


## gapit.mlm

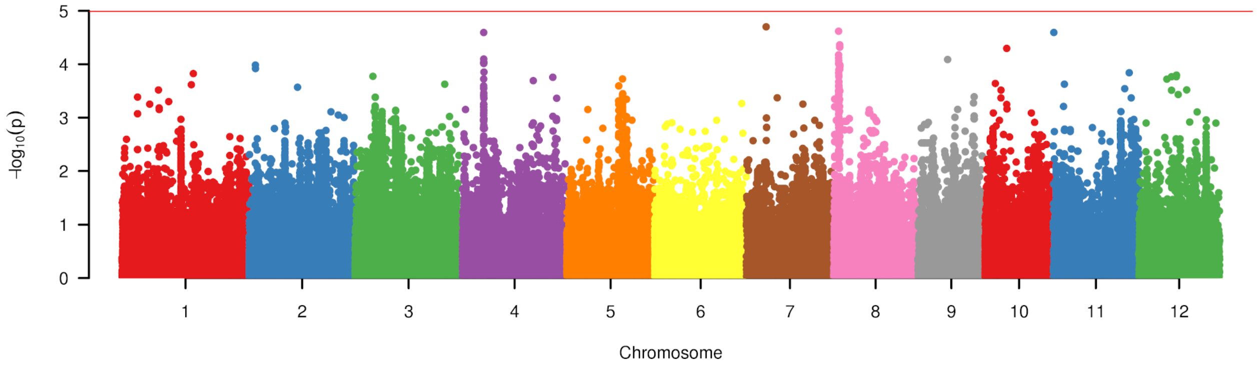


## lfmm

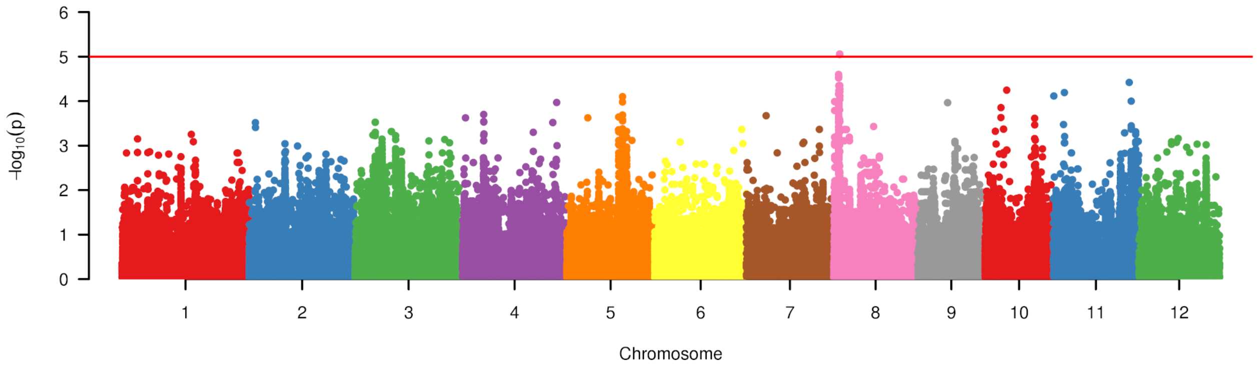

# tmaxPC1

## emma

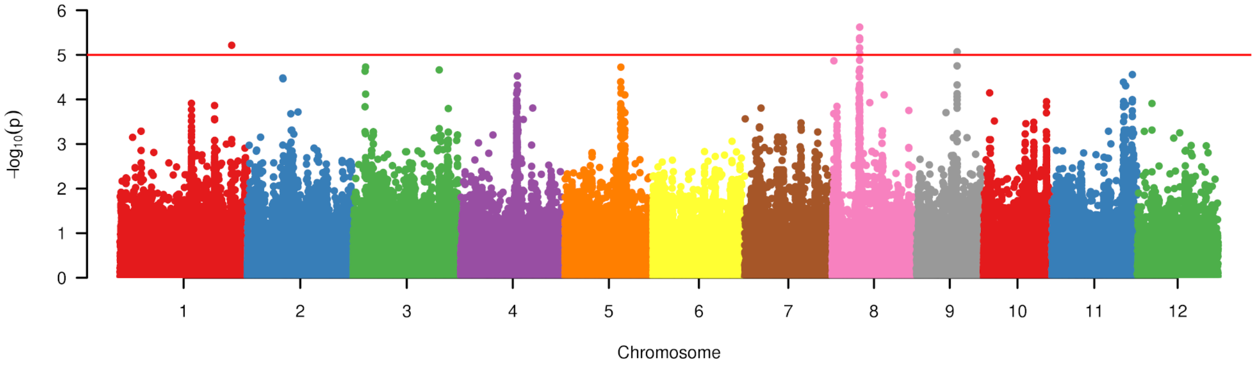


## gapit.mlm

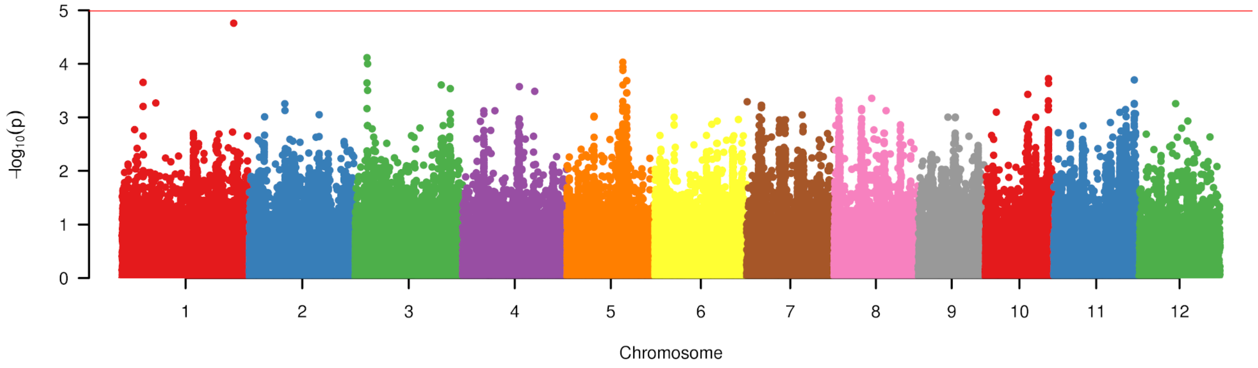


## lfmm

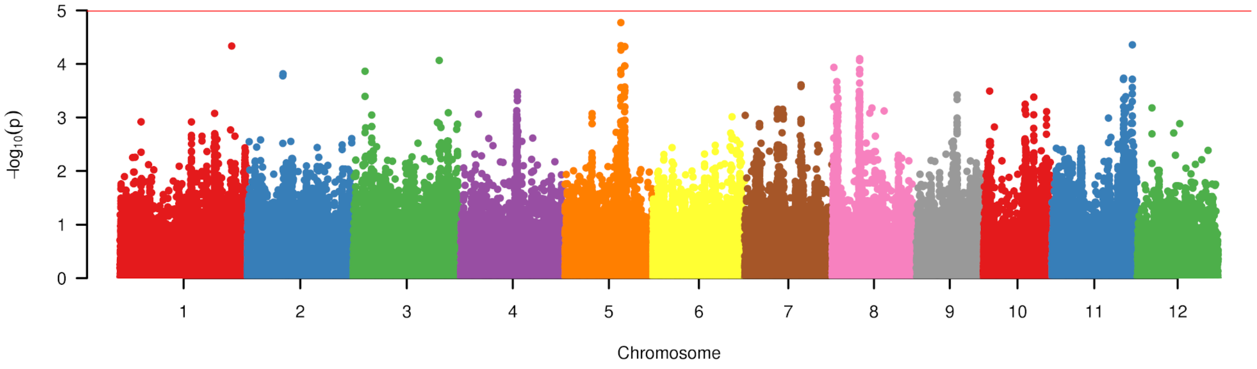

# tmaxPC2

emma

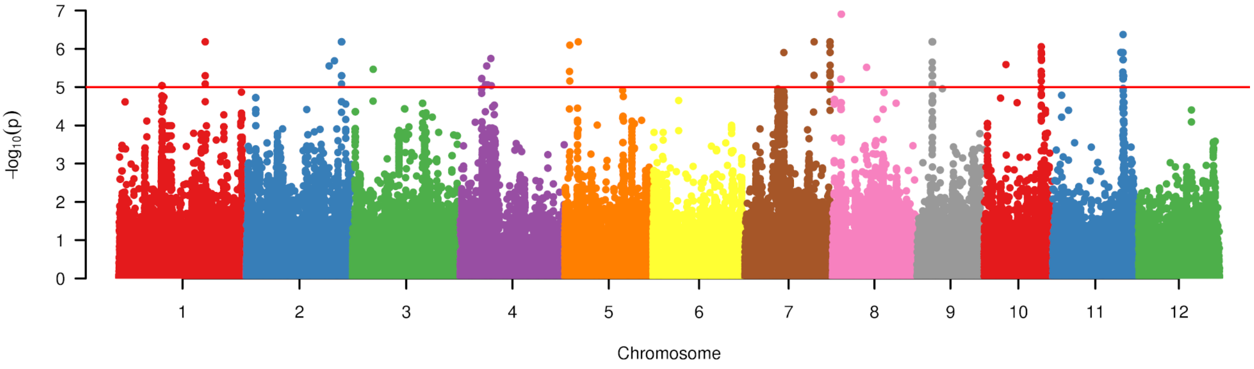


gapit.mlm

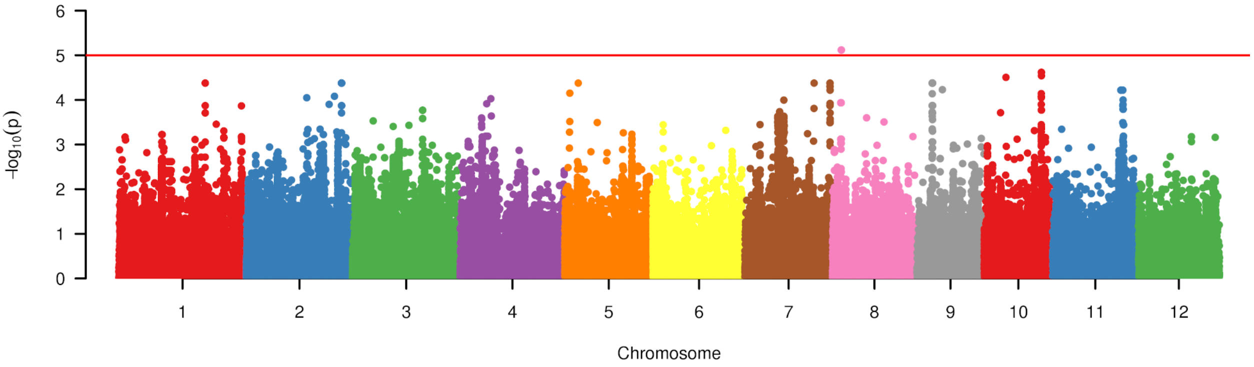


lfmm

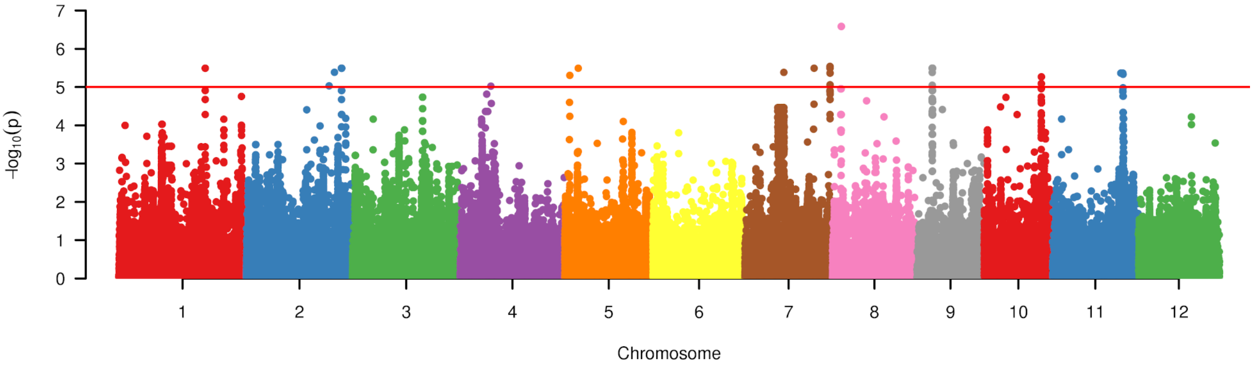

Supplement: Supplementary file 10 — Additional file 10: Figure S7. Manhattan plots. Association analysis were performed independently for each trait and repetition and based on three different methods (EMMA, LFMM, MLM). The transformed data were used if at least one of the replicates failed to reach normality. P-values obtained for each replicate were then combined using a Fisher combined probability test method to obtain the final p-values represented in this Manhattan plots. The 10− 5 thresholds are indicated by red lines. [file 12284_2020_424_MOESM10_ESM.pdf]
