# Supplementary material for: Genome Wide Association Study Pinpoints Key Agronomic QTLs in African Rice Oryza glaberrima
Source: Rice (N Y). 2020 Sep 16;13:66. doi: 10.1186/s12284-020-00424-1 (PMC7494698; doi:10.1186/s12284-020-00424-1)
Supplement: Supplementary file 11 — Additional file 11: Figure S8. Log scale QQ-plots corresponding to association analysis performed independently on each trait and repetition. Three different models (i.e. EMMA, LFMM, MLM) taking into account relatedness and/or structure were used for association and ANOVA was used as a benchmark. For a given trait, the transformed data were used if at least one of the replicates failed to reach normality, otherwise non-transformed data were used. [file 12284_2020_424_MOESM11_ESM.pdf]

T_bioPC1

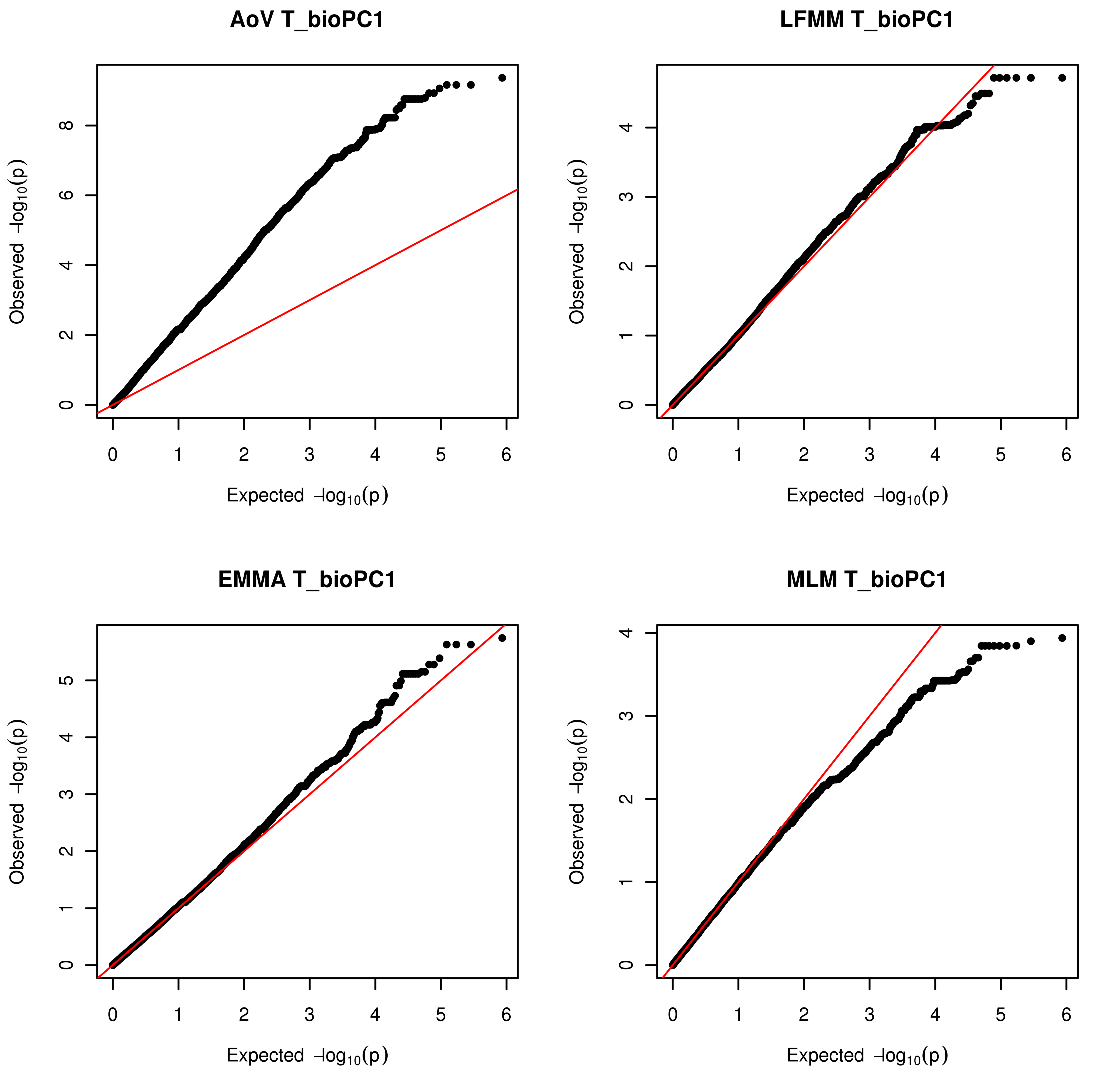

AoV T_bioPC1
LFMM T_bioPC1
EMMA T_bioPC1
MLM T_bioPC1
Observed $-\log_{10}(p)$
Expected $-\log_{10}(p)$
0
1
2
3
4
5
6
8

T_bioPC2

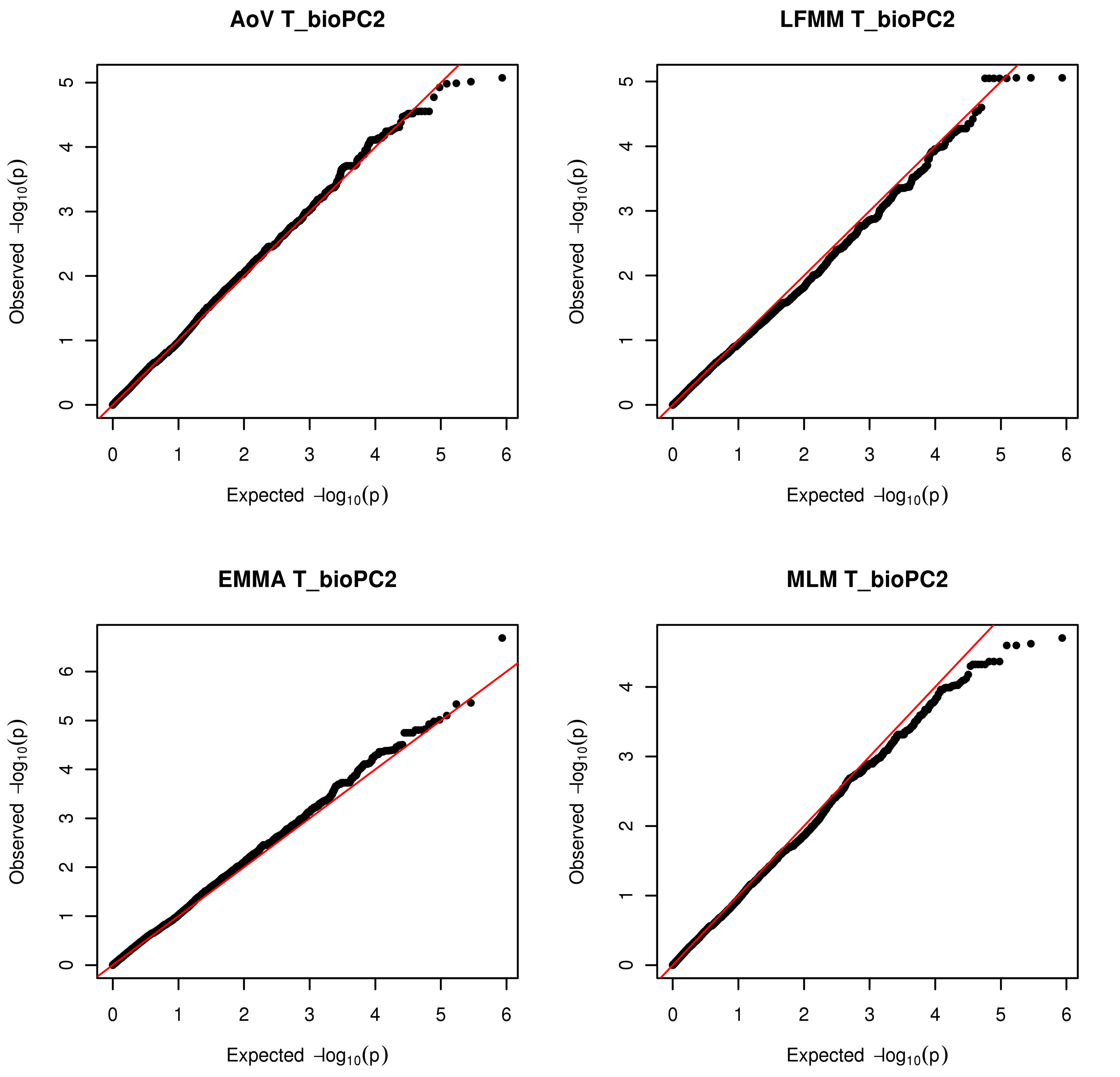
AoV T_bioPC2
Observed −log10(p)
Expected −log10(p)
LFMM T_bioPC2
Observed −log10(p)
Expected −log10(p)
EMMA T_bioPC2
Observed −log10(p)
Expected −log10(p)
MLM T_bioPC2
Observed −log10(p)
Expected −log10(p)

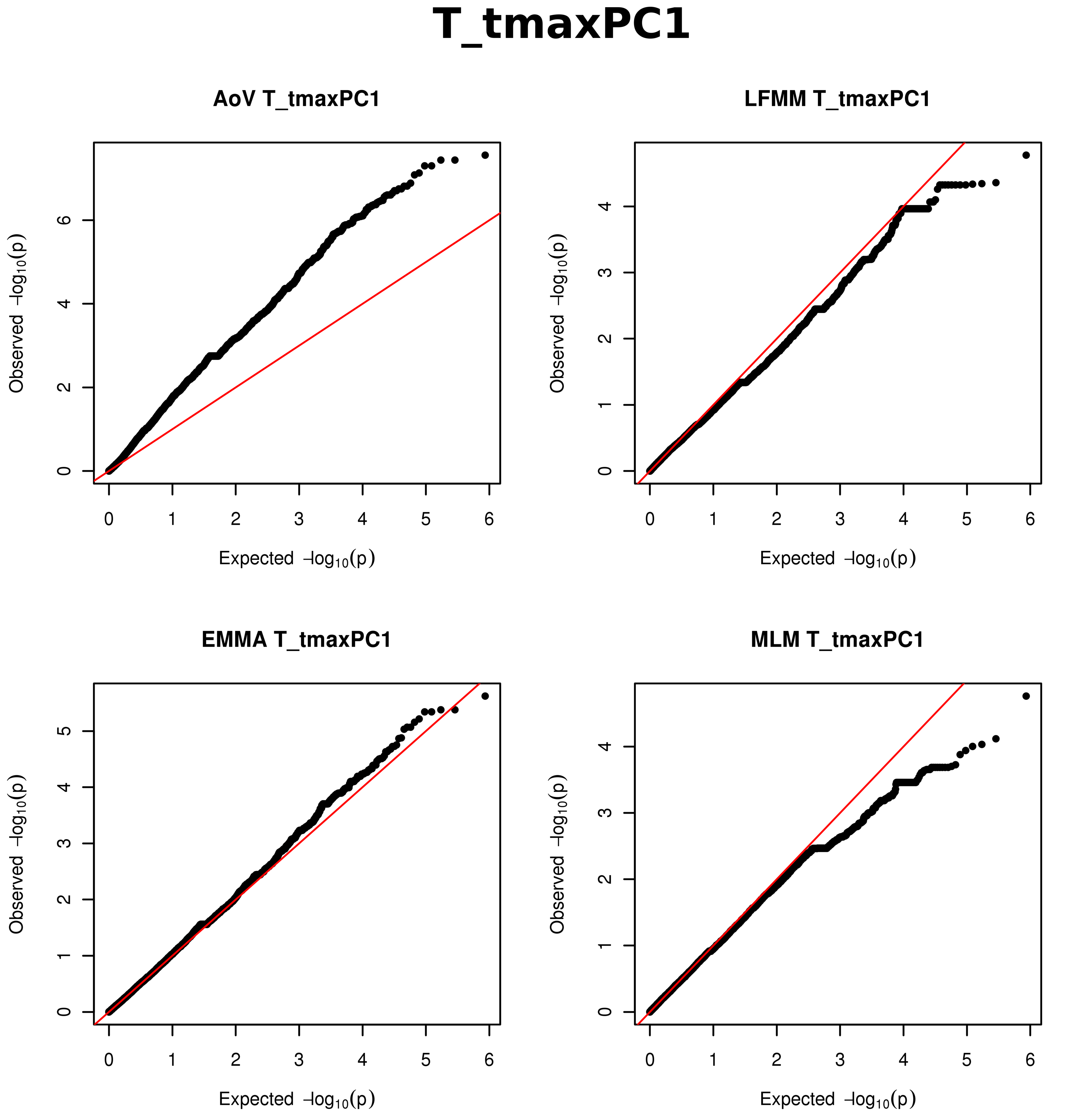
T_tmaxPC1
AoV T_tmaxPC1
LFMM T_tmaxPC1
EMMA T_tmaxPC1
MLM T_tmaxPC1
Observed −log10(p)
Expected −log10(p)
0
1
2
3
4
5
6

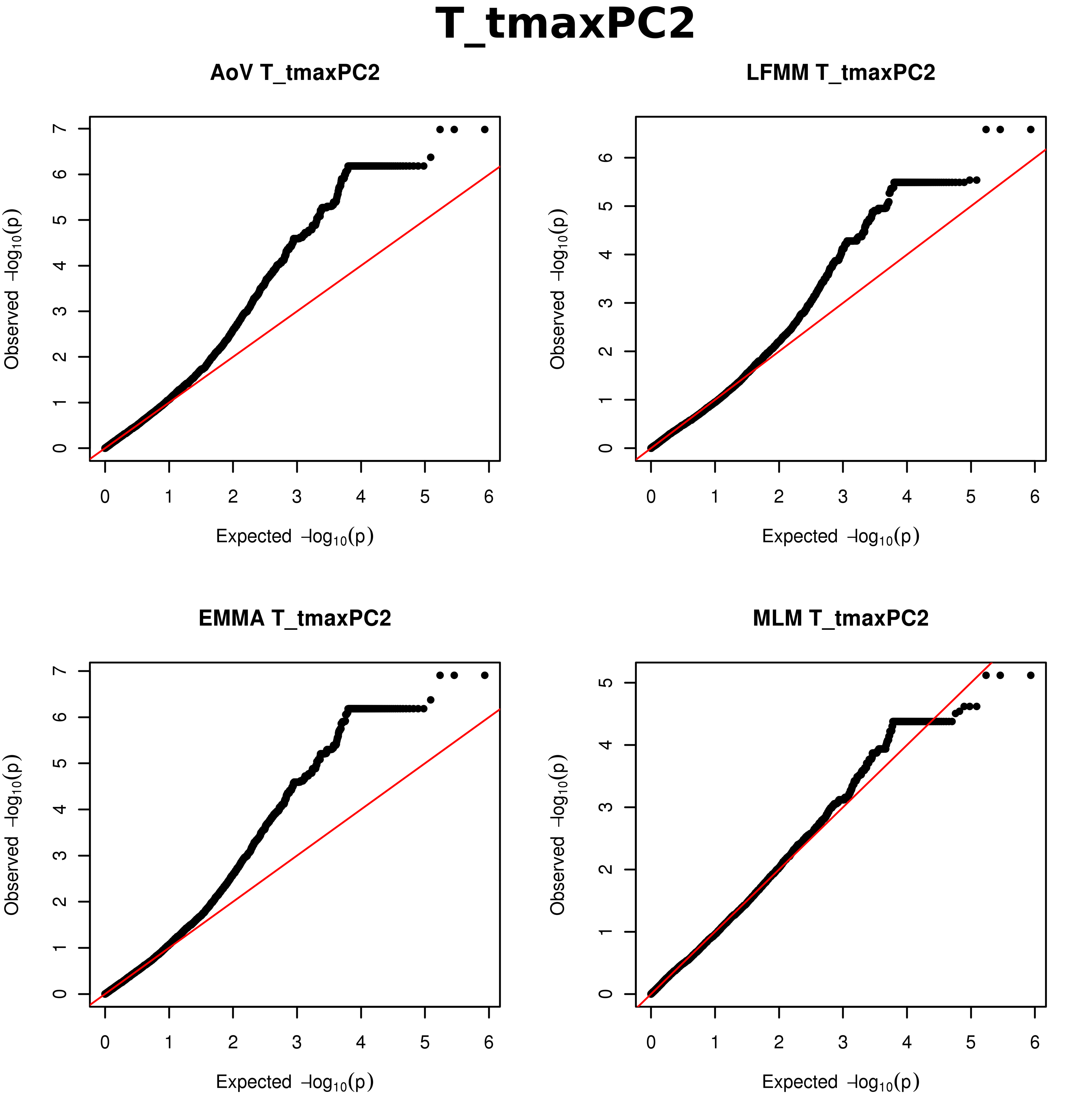
T_tmaxPC2
AoV T_tmaxPC2
LFMM T_tmaxPC2
EMMA T_tmaxPC2
MLM T_tmaxPC2
Observed −log10(p)
Expected −log10(p)
0
1
2
3
4
5
6
7

DFT2012a

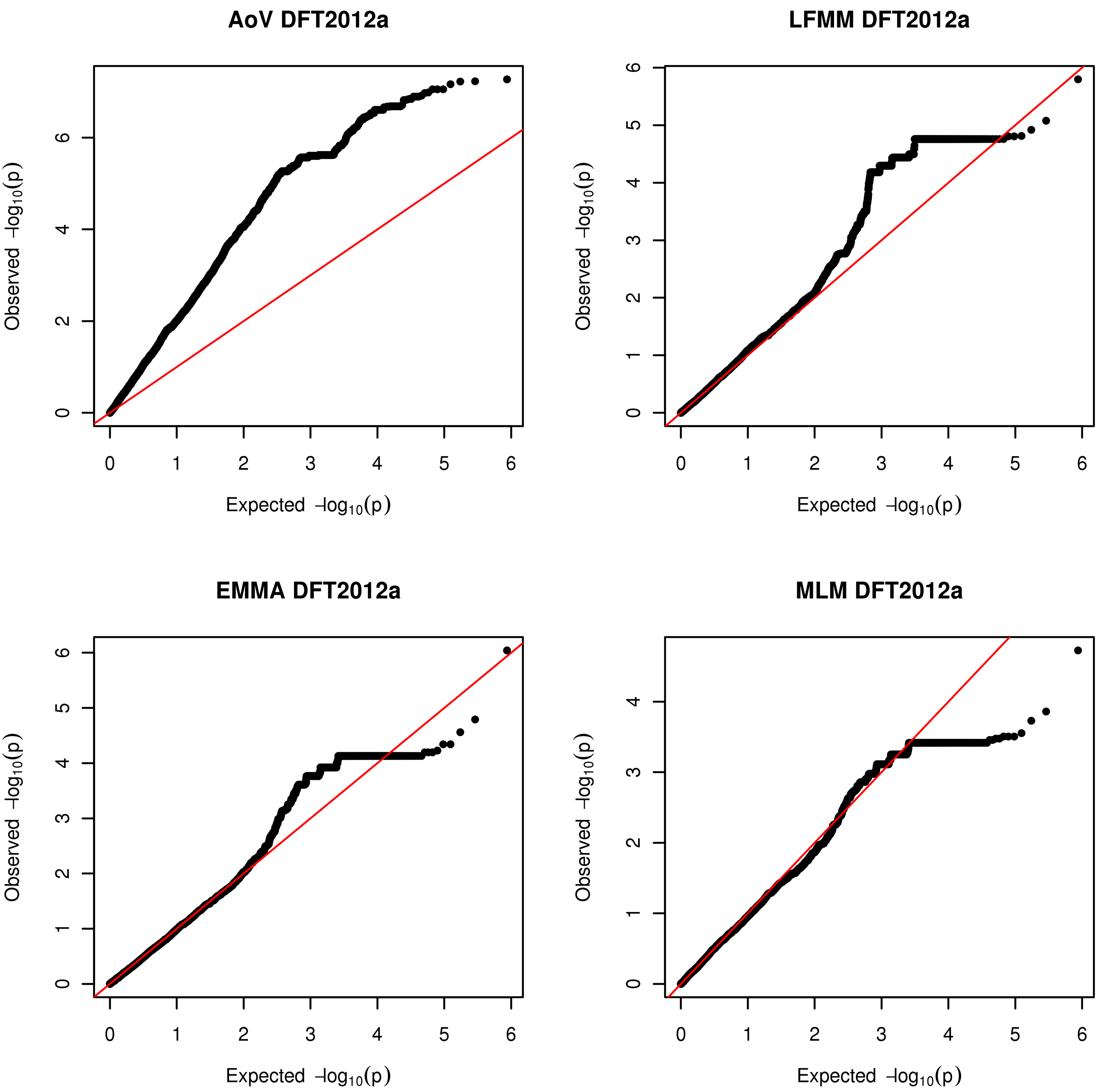
AoV DFT2012a
LFMM DFT2012a
EMMA DFT2012a
MLM DFT2012a
Observed −log10(p)
Expected −log10(p)

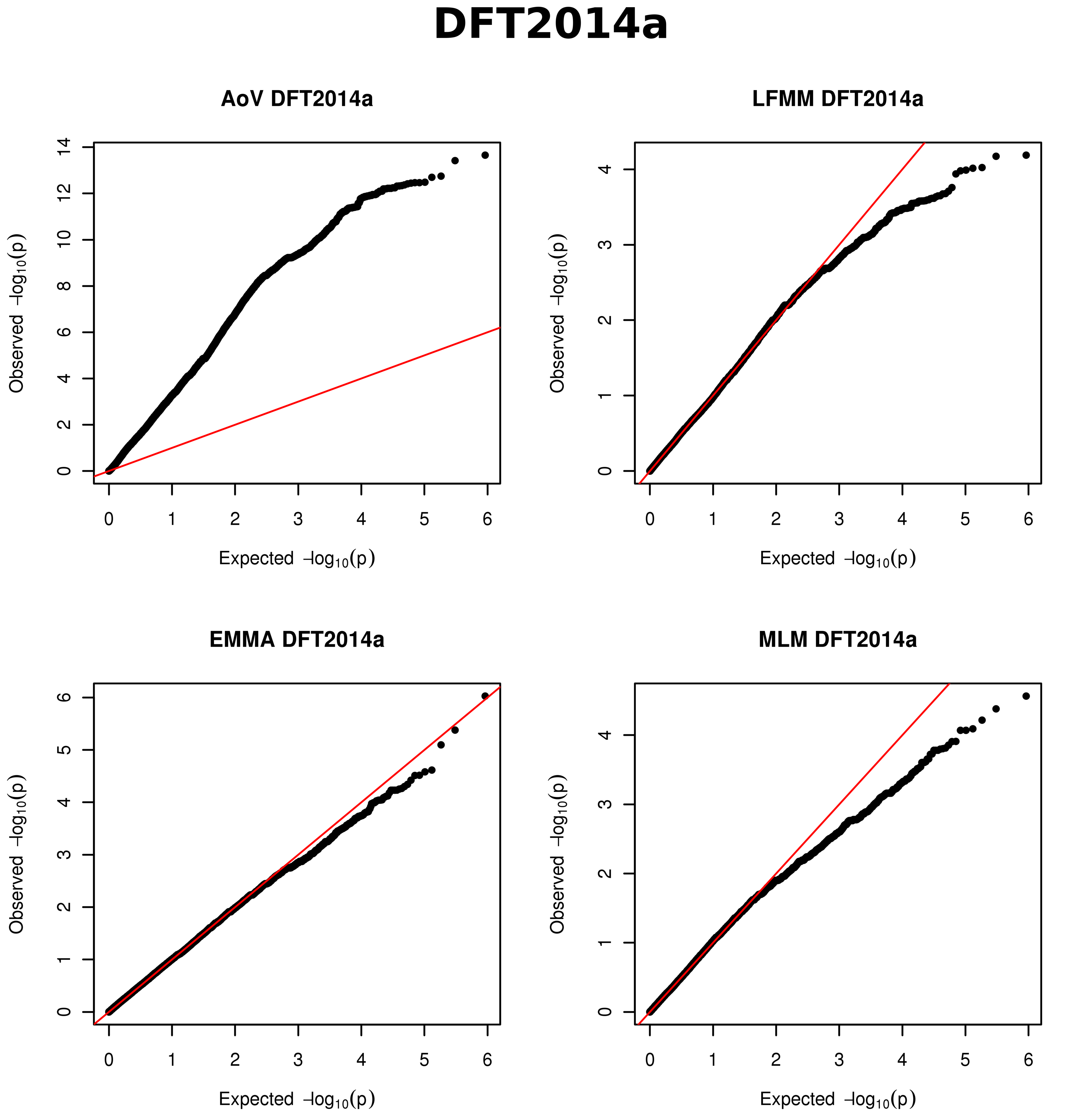
DFT2014a
AoV DFT2014a
LFMM DFT2014a
EMMA DFT2014a
MLM DFT2014a
Observed −log10(p)
Expected −log10(p)

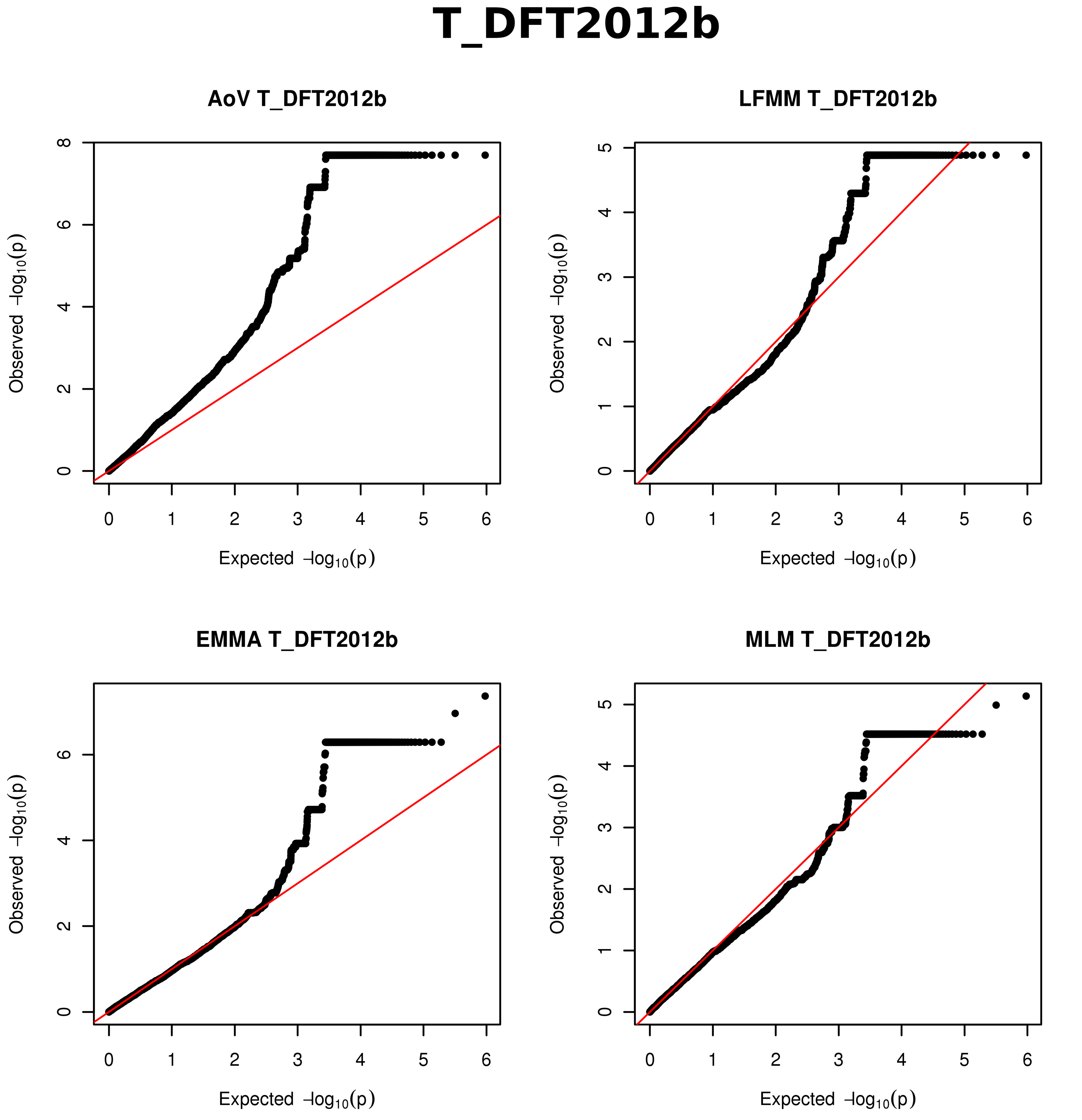
T_DFT2012b
AoV T_DFT2012b
LFMM T_DFT2012b
EMMA T_DFT2012b
MLM T_DFT2012b
Observed −log10(p)
Expected −log10(p)
0
1
2
3
4
5
6
8

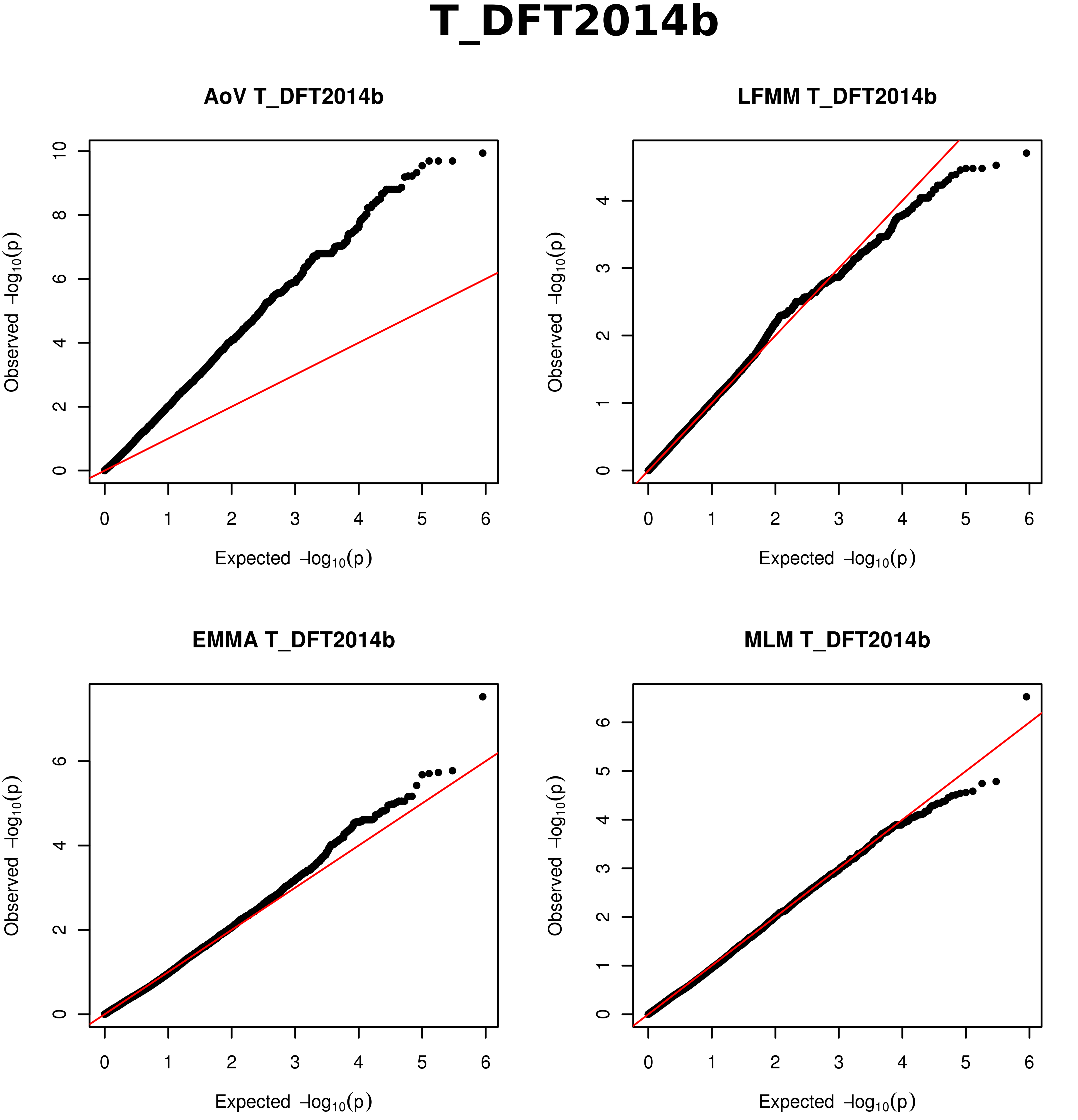
T_DFT2014b
AoV T_DFT2014b
LFMM T_DFT2014b
EMMA T_DFT2014b
MLM T_DFT2014b
Observed $-\log_{10}(p)$
Expected $-\log_{10}(p)$

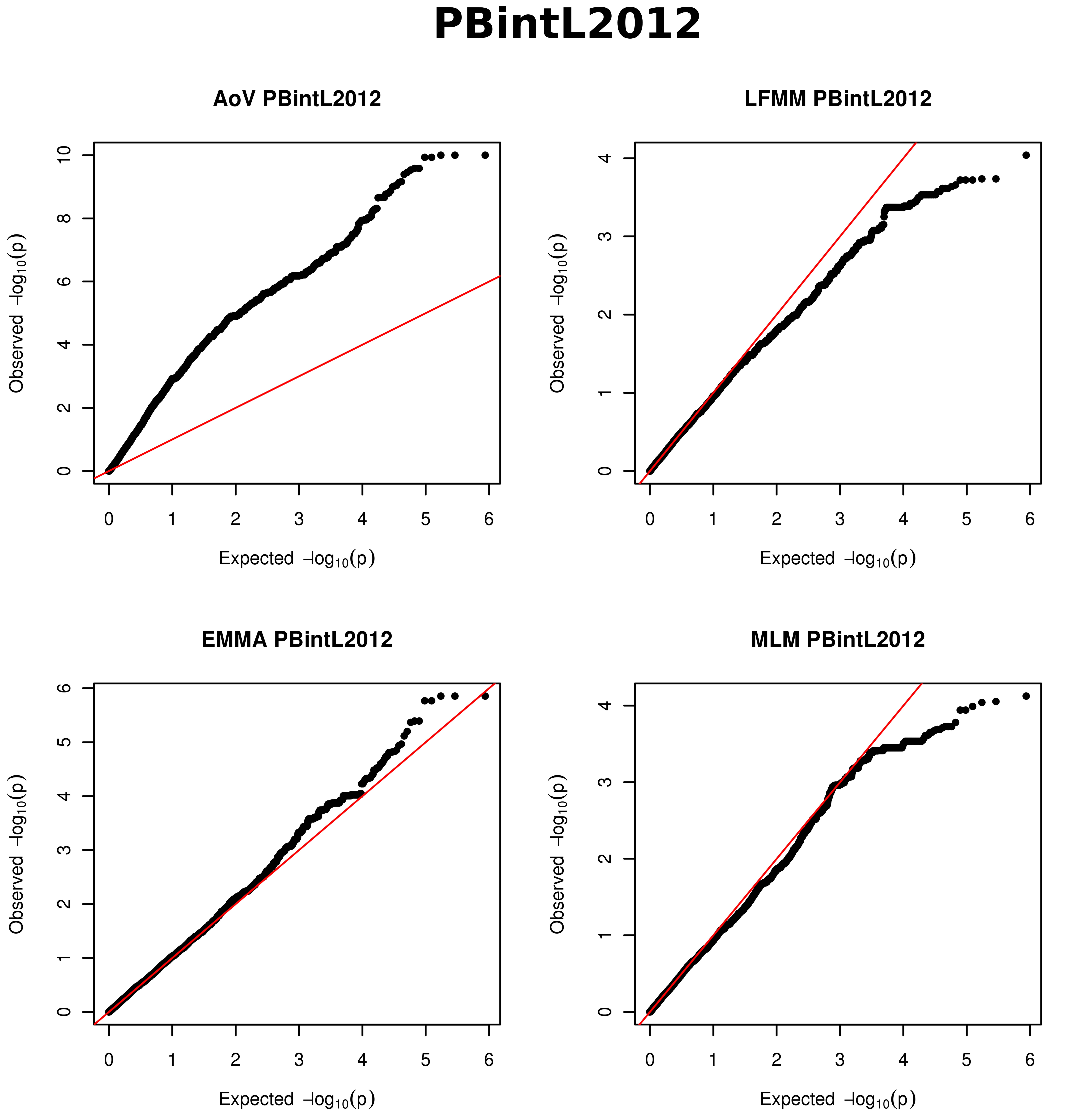
PBintL2012
AoV PBintL2012
LFMM PBintL2012
EMMA PBintL2012
MLM PBintL2012
Observed −log10(p)
Expected −log10(p)
0
1
2
3
4
5
6
8
10

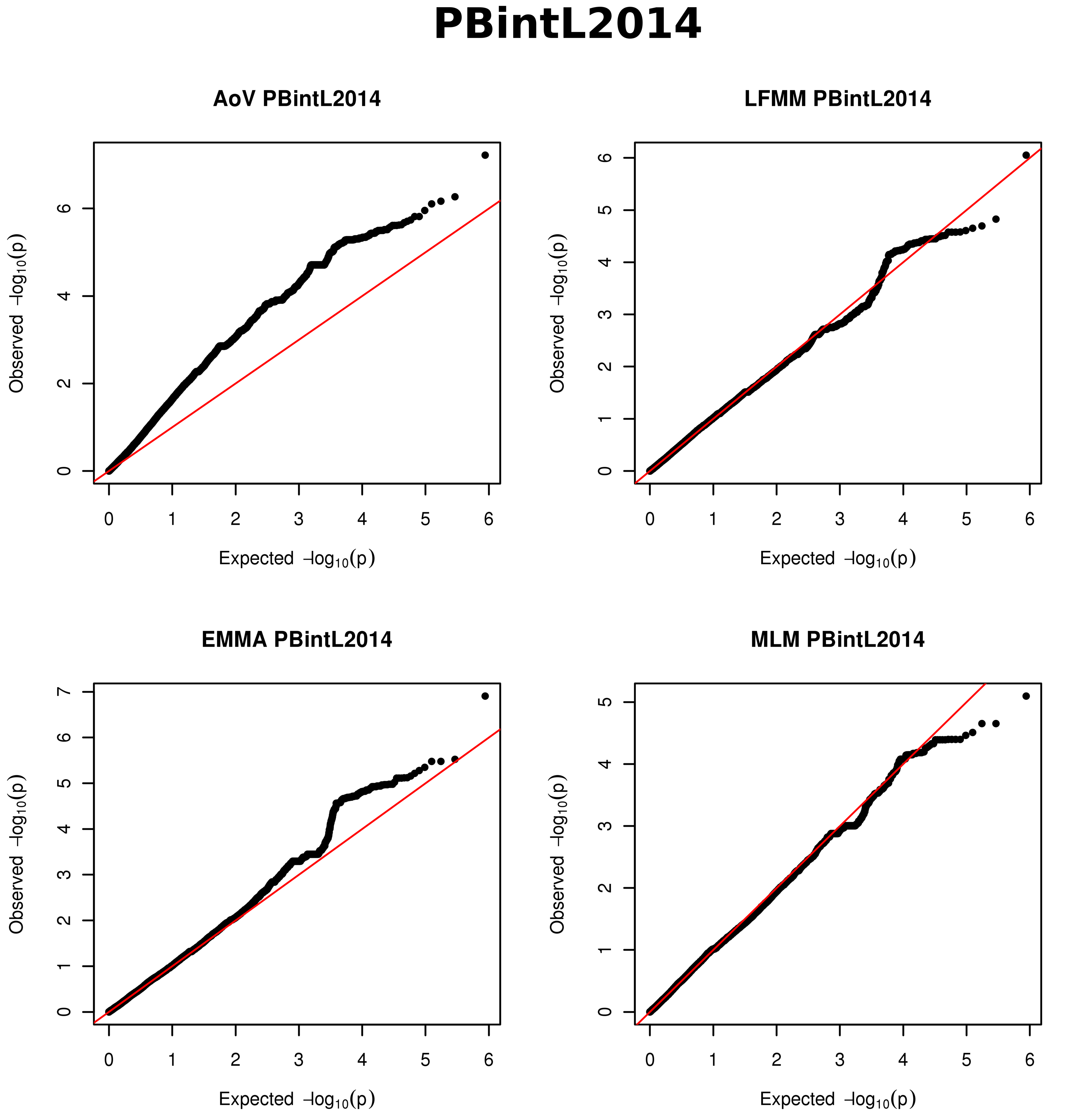
PBintL2014
AoV PBintL2014
LFMM PBintL2014
EMMA PBintL2014
MLM PBintL2014
Observed $-\log_{10}(p)$
Expected $-\log_{10}(p)$
0
1
2
3
4
5
6
7

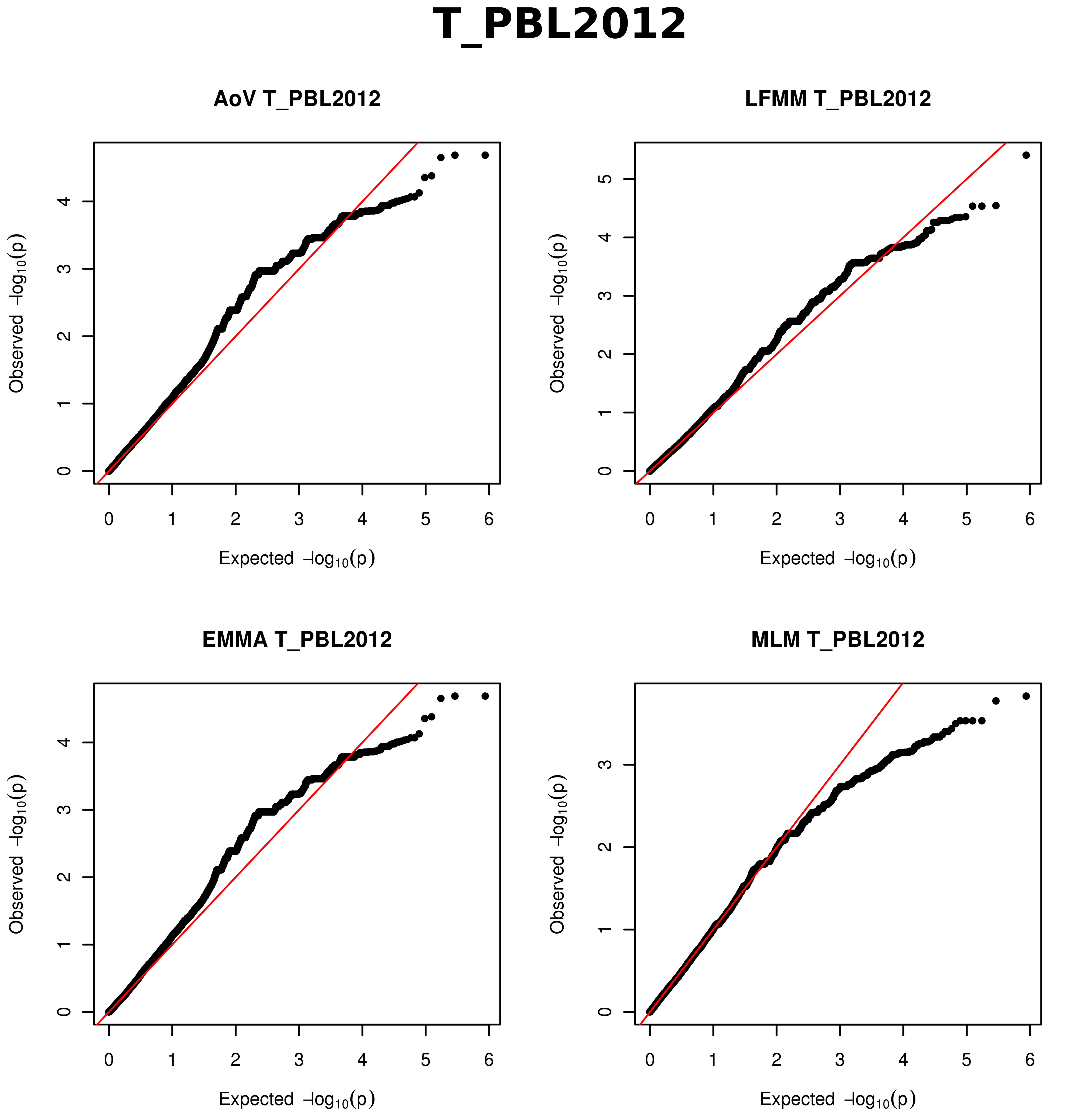
T_PBL2012
AoV T_PBL2012
LFMM T_PBL2012
EMMA T_PBL2012
MLM T_PBL2012
Observed −log10(p)
Expected −log10(p)
0
1
2
3
4
5
6

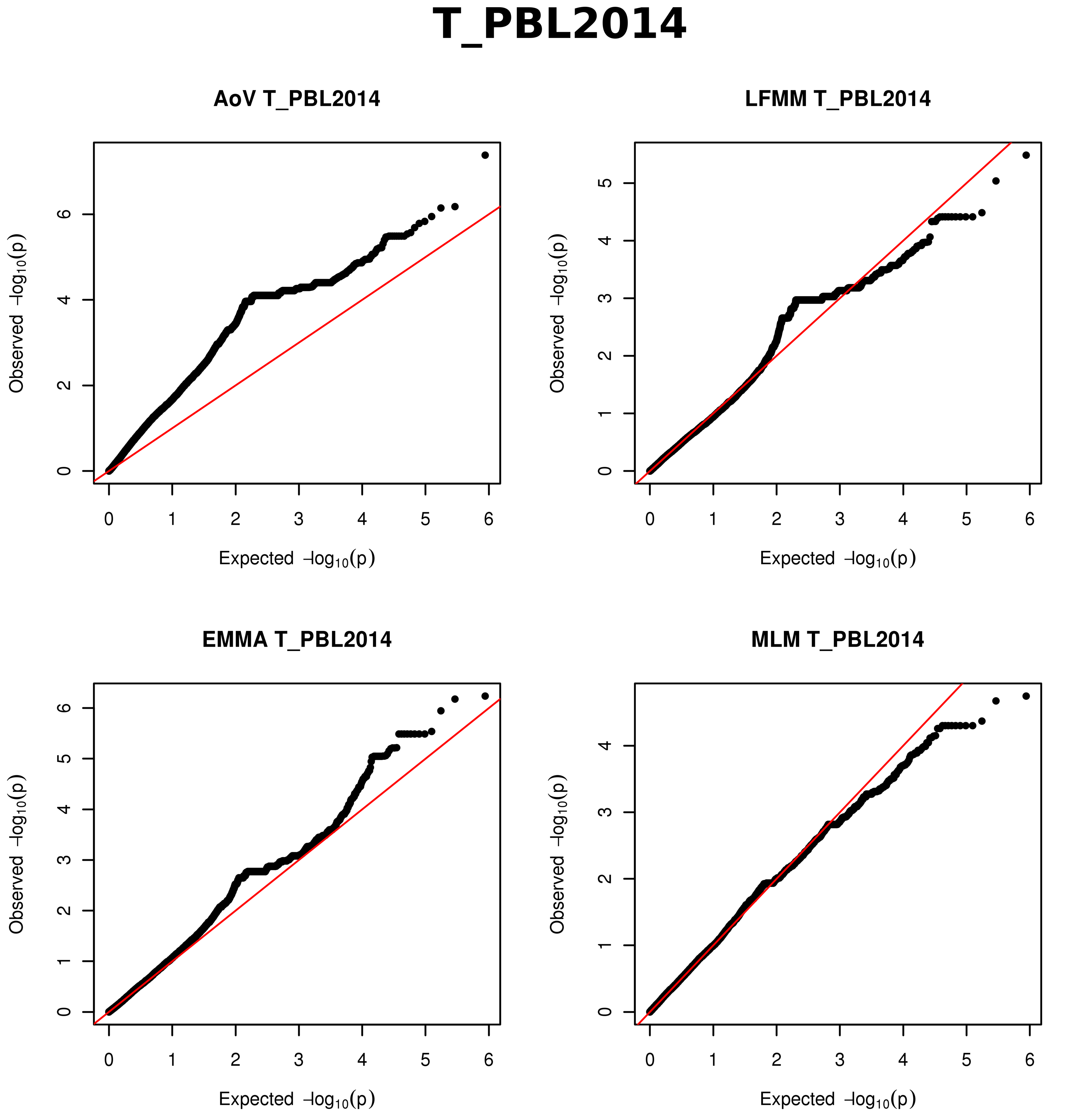
T_PBL2014
AoV T_PBL2014
LFMM T_PBL2014
EMMA T_PBL2014
MLM T_PBL2014
Observed -log10(p)
Expected -log10(p)
0
1
2
3
4
5
6

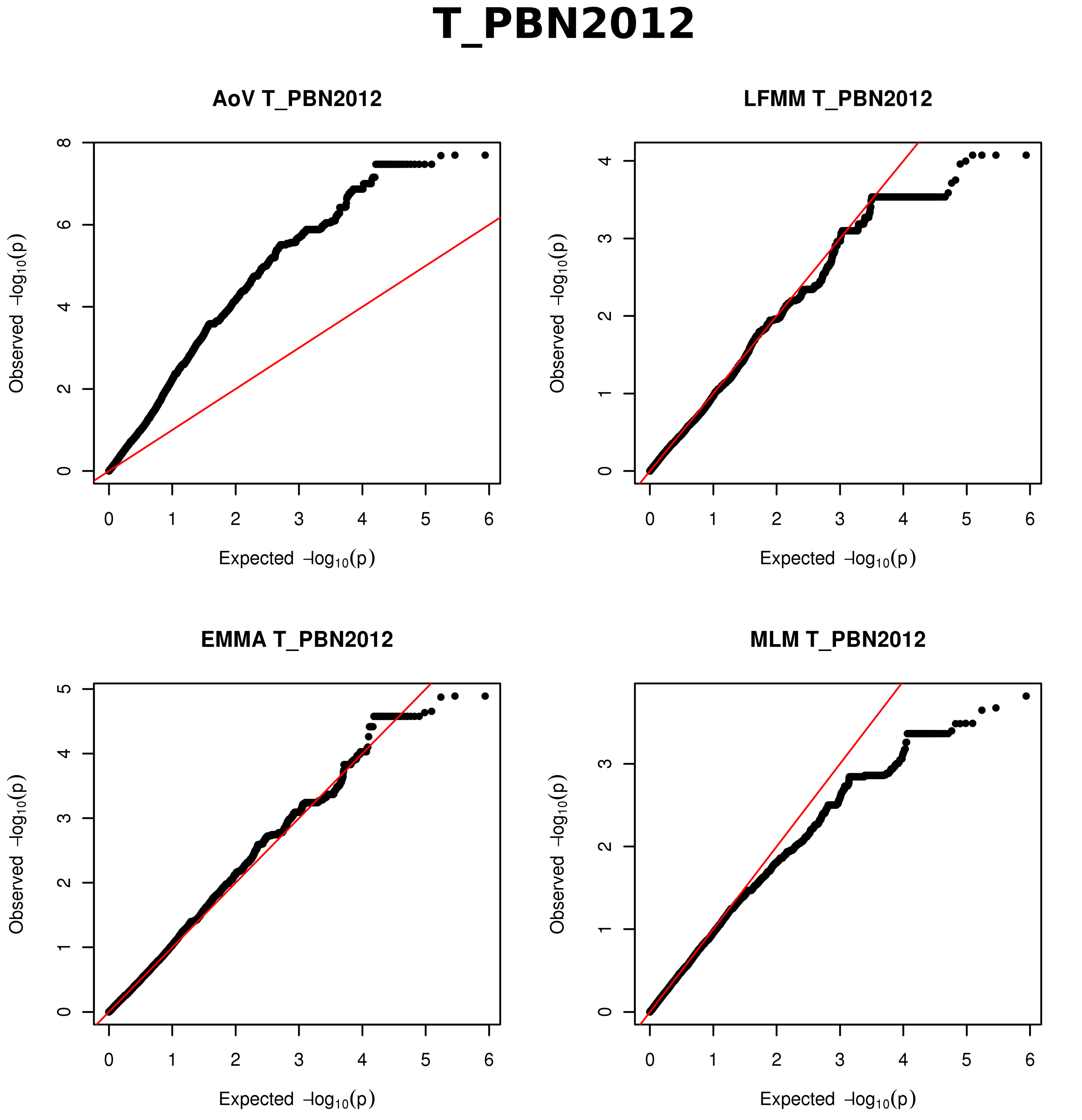
T_PBN2012
AoV T_PBN2012
LFMM T_PBN2012
EMMA T_PBN2012
MLM T_PBN2012
Observed −log10(p)
Expected −log10(p)
0
1
2
3
4
5
6
7
8

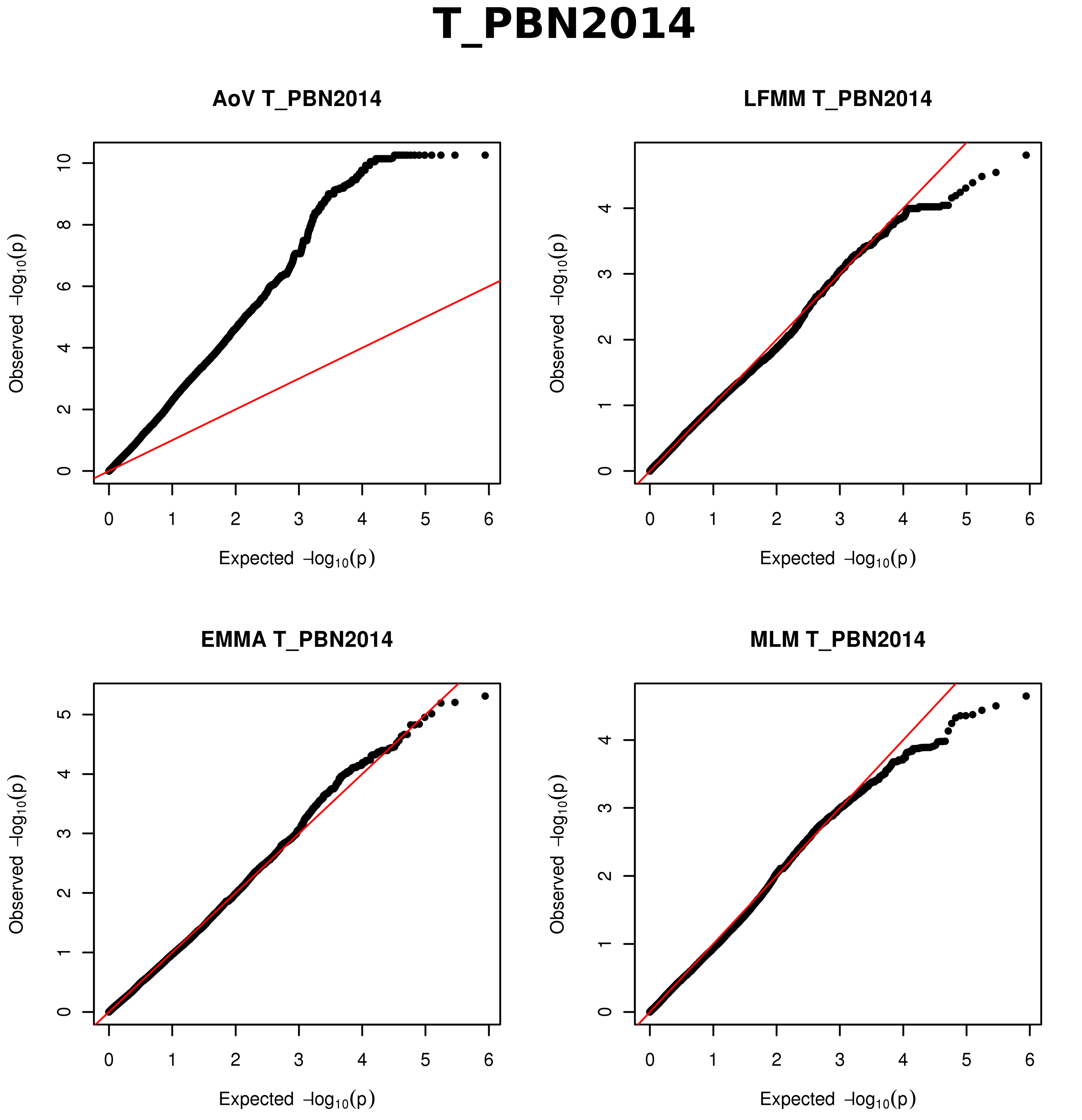
T_PBN2014
AoV T_PBN2014
LFMM T_PBN2014
EMMA T_PBN2014
MLM T_PBN2014
Expected −log10(p)
Observed −log10(p)
0
1
2
3
4
5
6
8
10

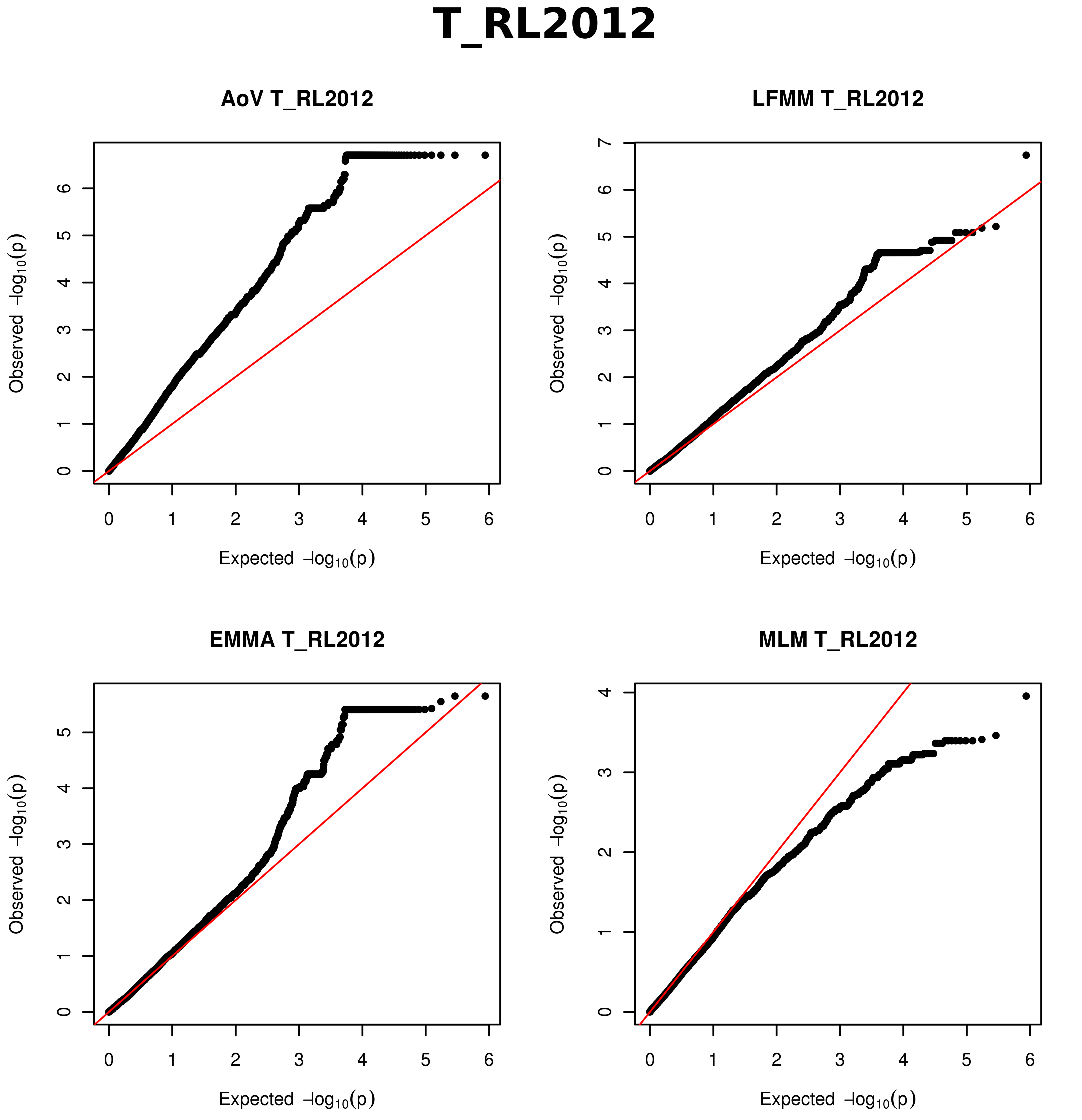
T_RL2012
AoV T_RL2012
LFMM T_RL2012
EMMA T_RL2012
MLM T_RL2012
Observed −log10(p)
Expected −log10(p)
0
1
2
3
4
5
6
7

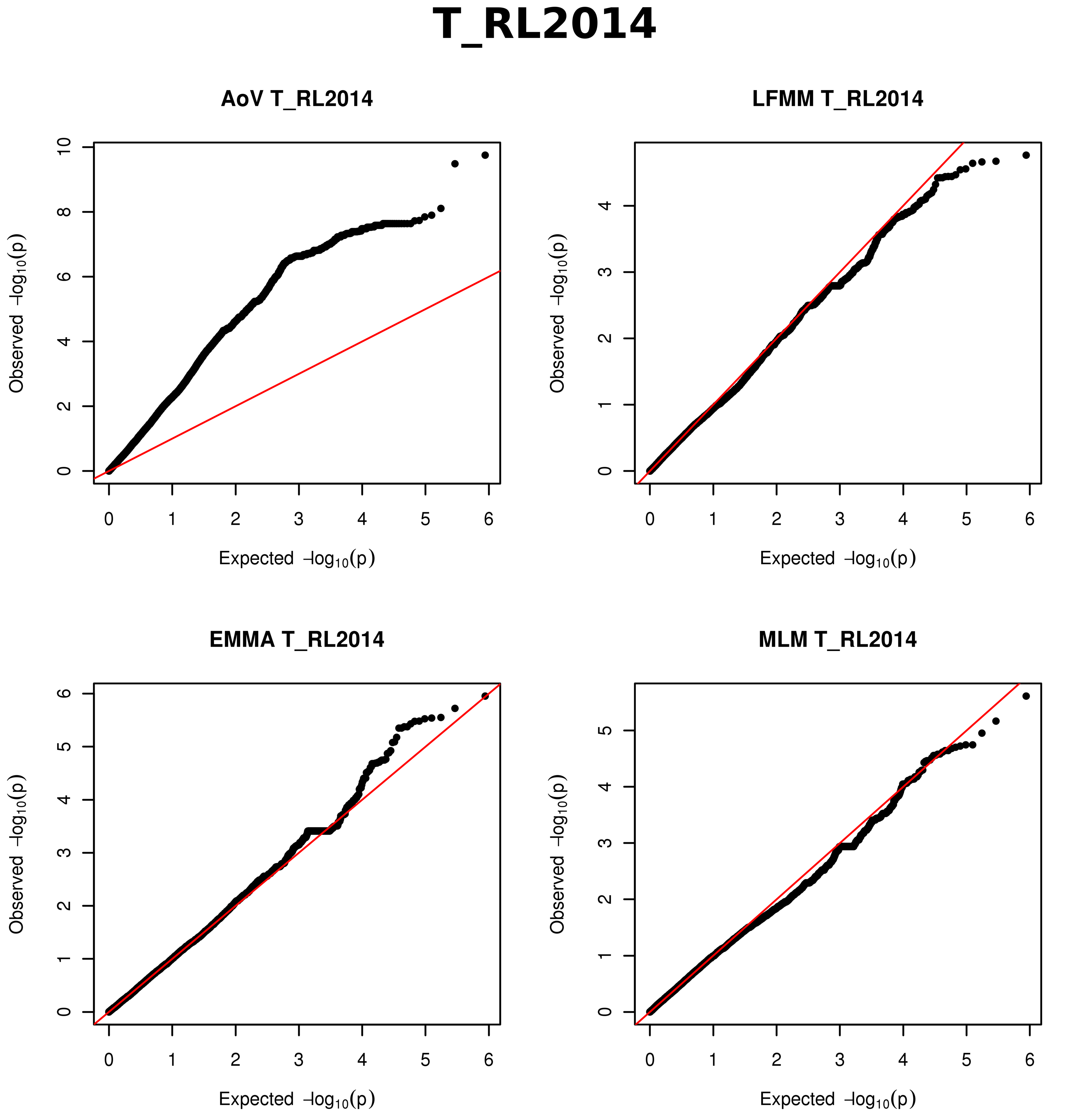
T_RL2014
AoV T_RL2014
LFMM T_RL2014
EMMA T_RL2014
MLM T_RL2014
Observed −log10(p)
Expected −log10(p)
0
2
4
6
8
10
0
1
2
3
4
5
6

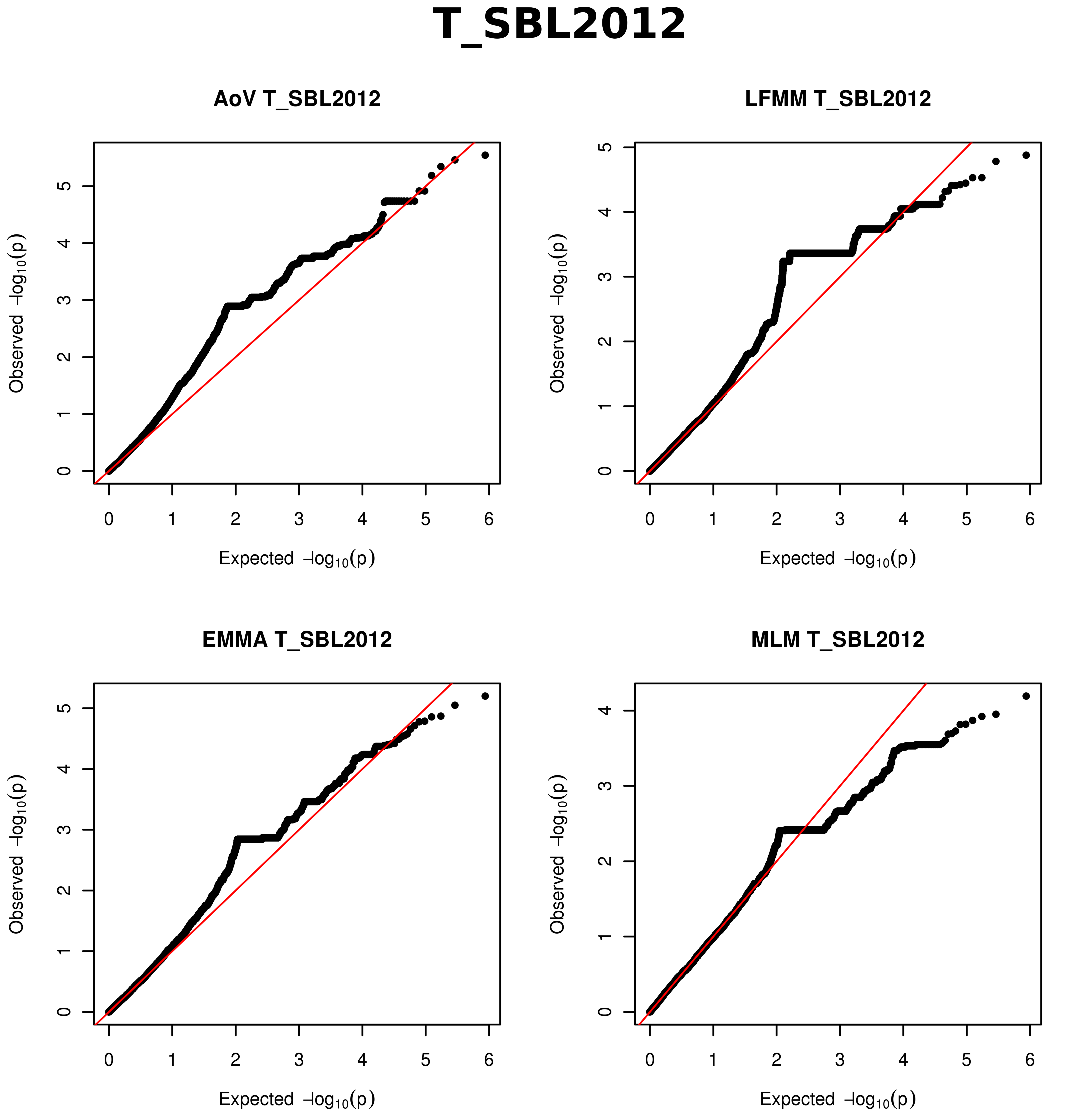
T_SBL2012
AoV T_SBL2012
LFMM T_SBL2012
EMMA T_SBL2012
MLM T_SBL2012
Observed $-\log_{10}(p)$
Expected $-\log_{10}(p)$
0
1
2
3
4
5
6

T_SBL2014

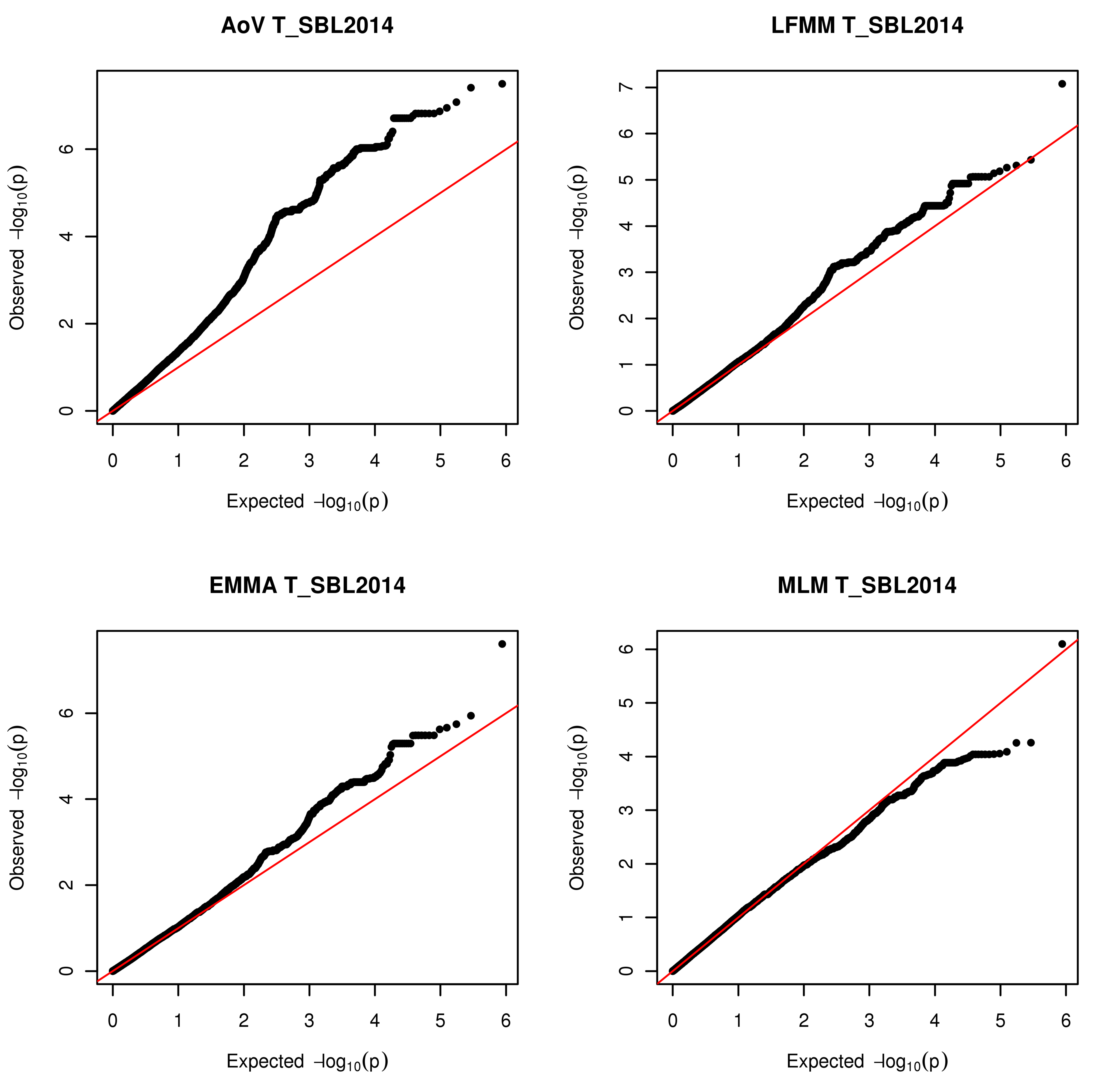

AoV T_SBL2014
LFMM T_SBL2014
EMMA T_SBL2014
MLM T_SBL2014
Observed −log10(p)
Expected −log10(p)
0
1
2
3
4
5
6
7

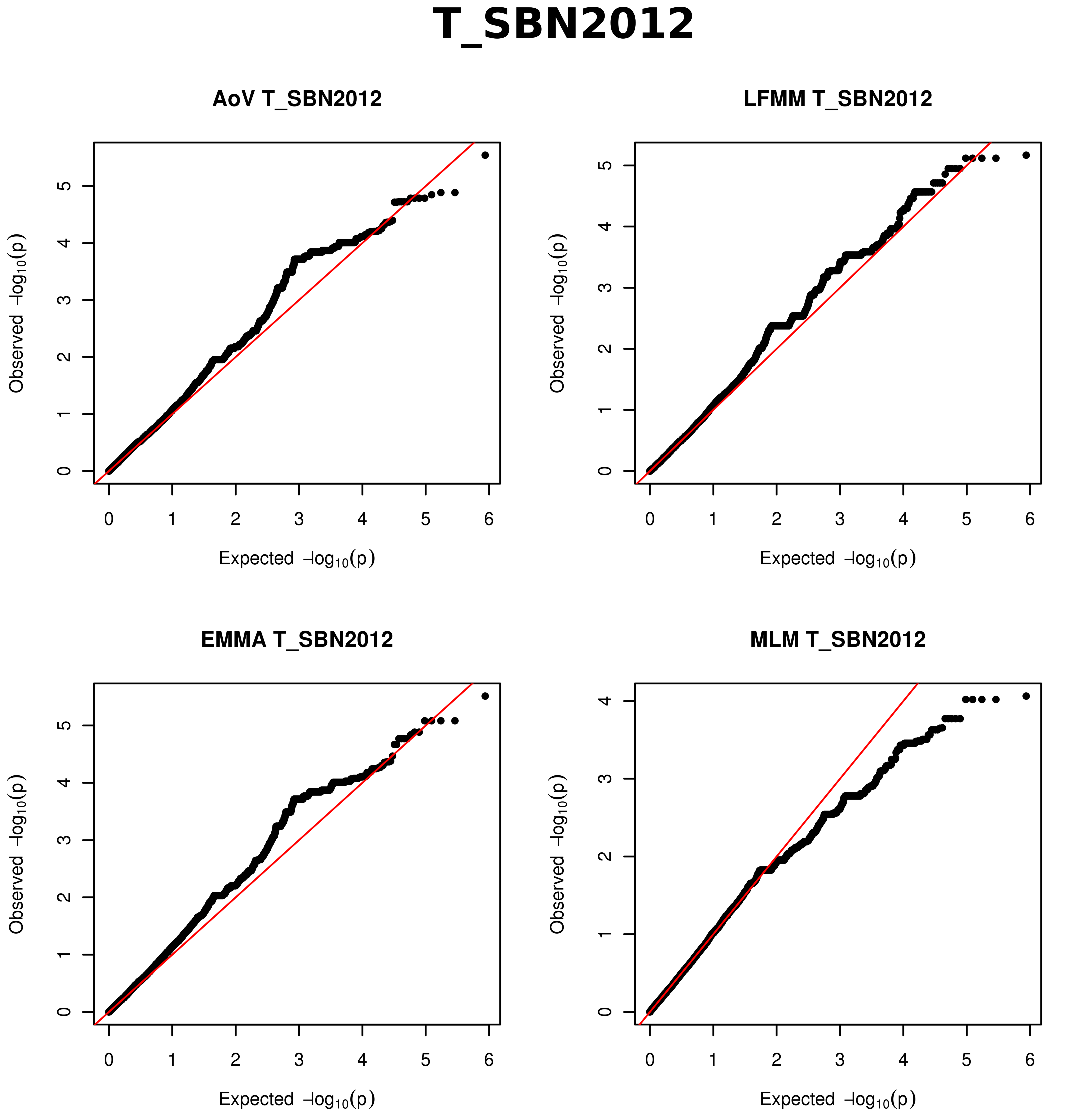

T_SBN2012
AoV T_SBN2012
LFMM T_SBN2012
EMMA T_SBN2012
MLM T_SBN2012
Observed −log10(p)
Expected −log10(p)
0
1
2
3
4
5
6

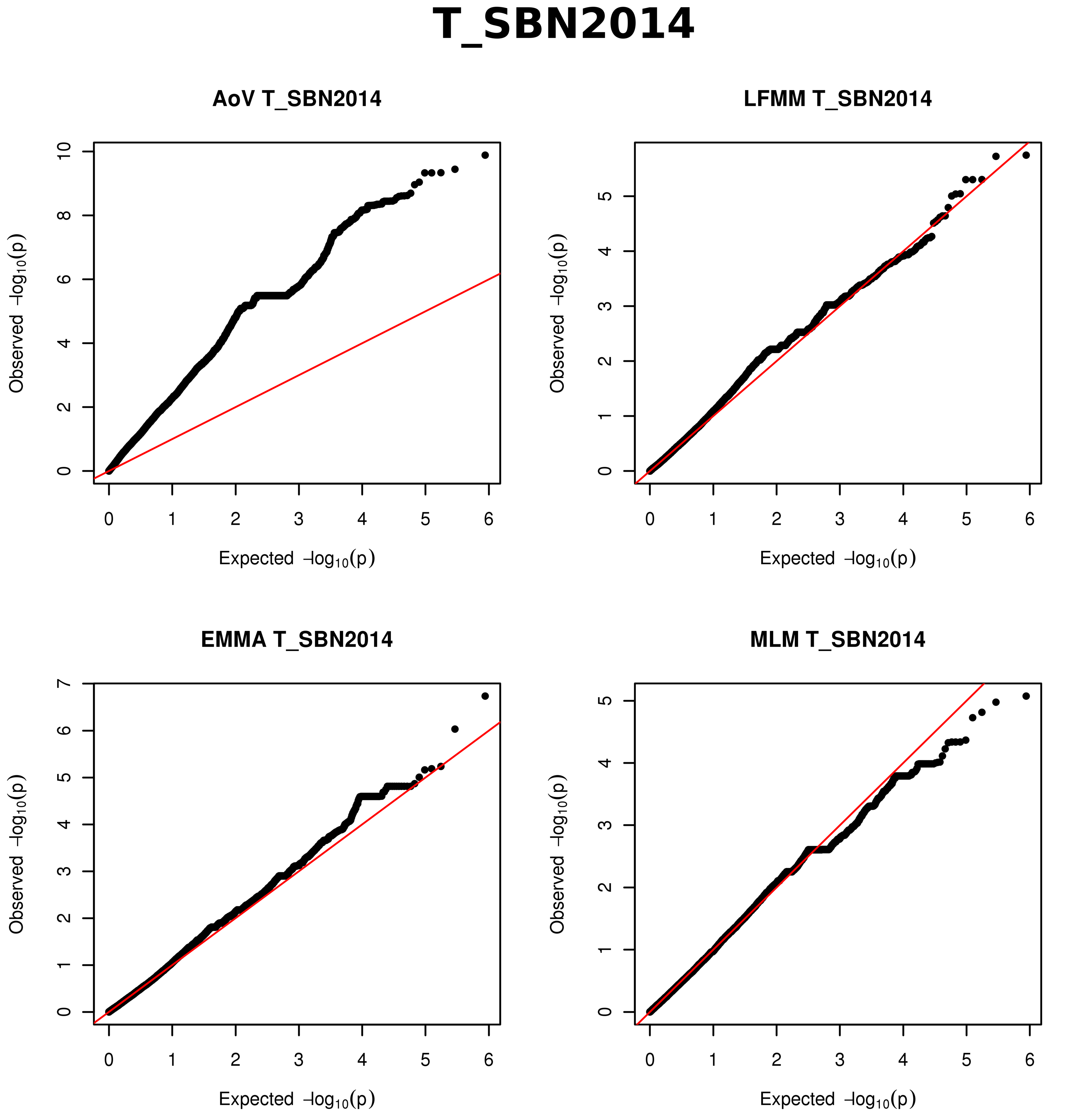
T_SBN2014
AoV T_SBN2014
LFMM T_SBN2014
EMMA T_SBN2014
MLM T_SBN2014
Observed −log10(p)
Expected −log10(p)

T_SBintL2012

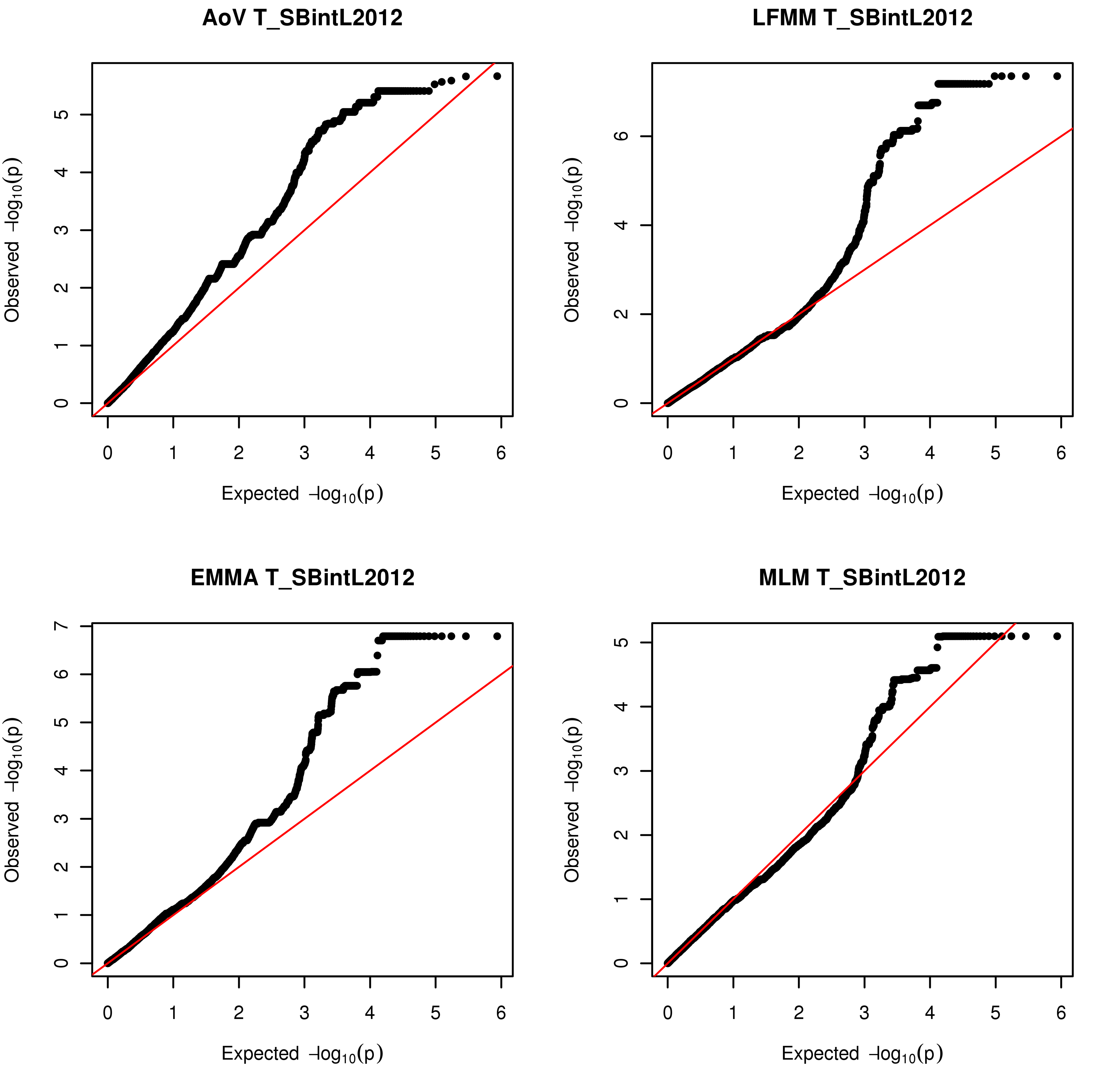

T_SBintL2014

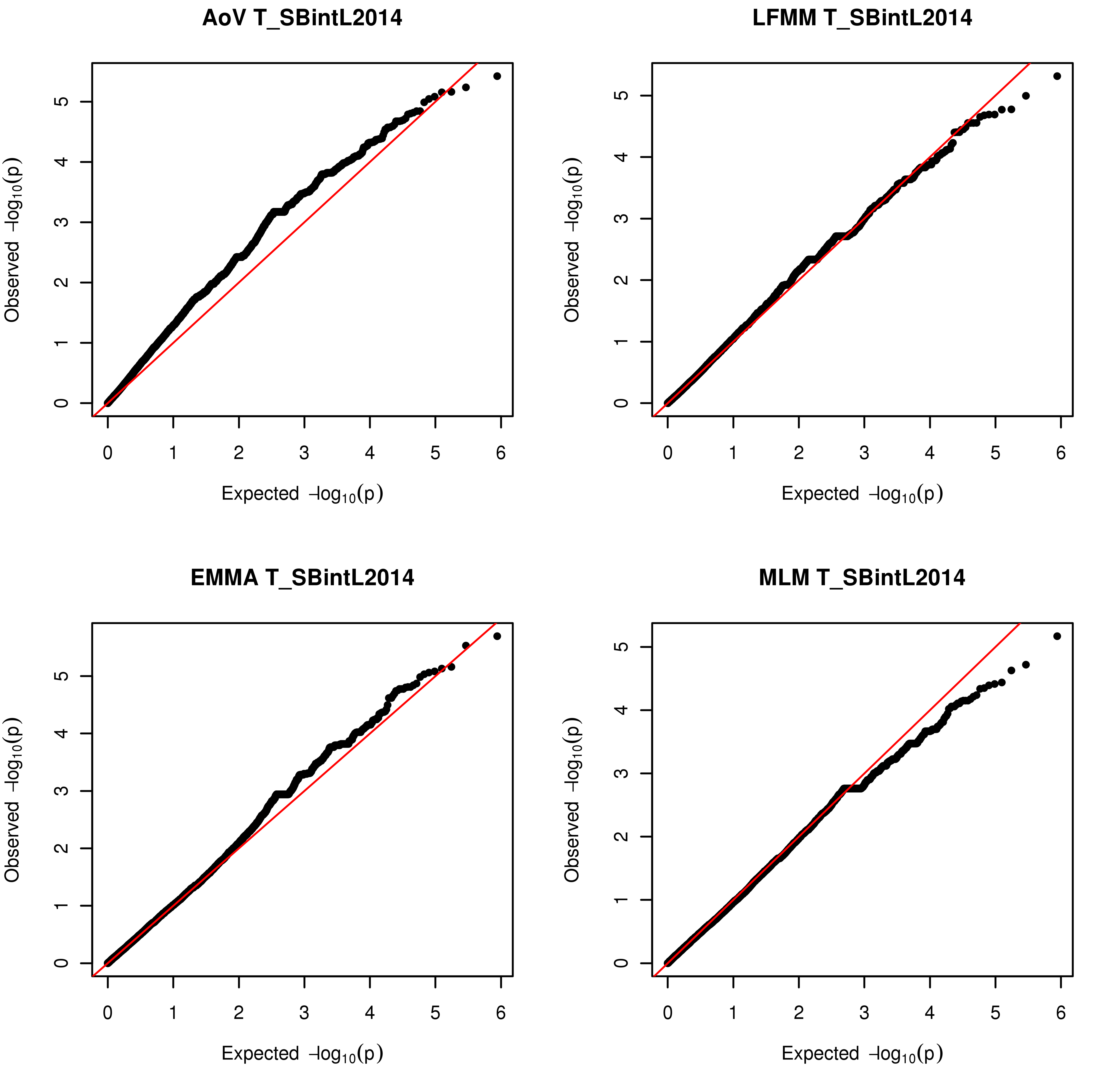

T_SPN2012

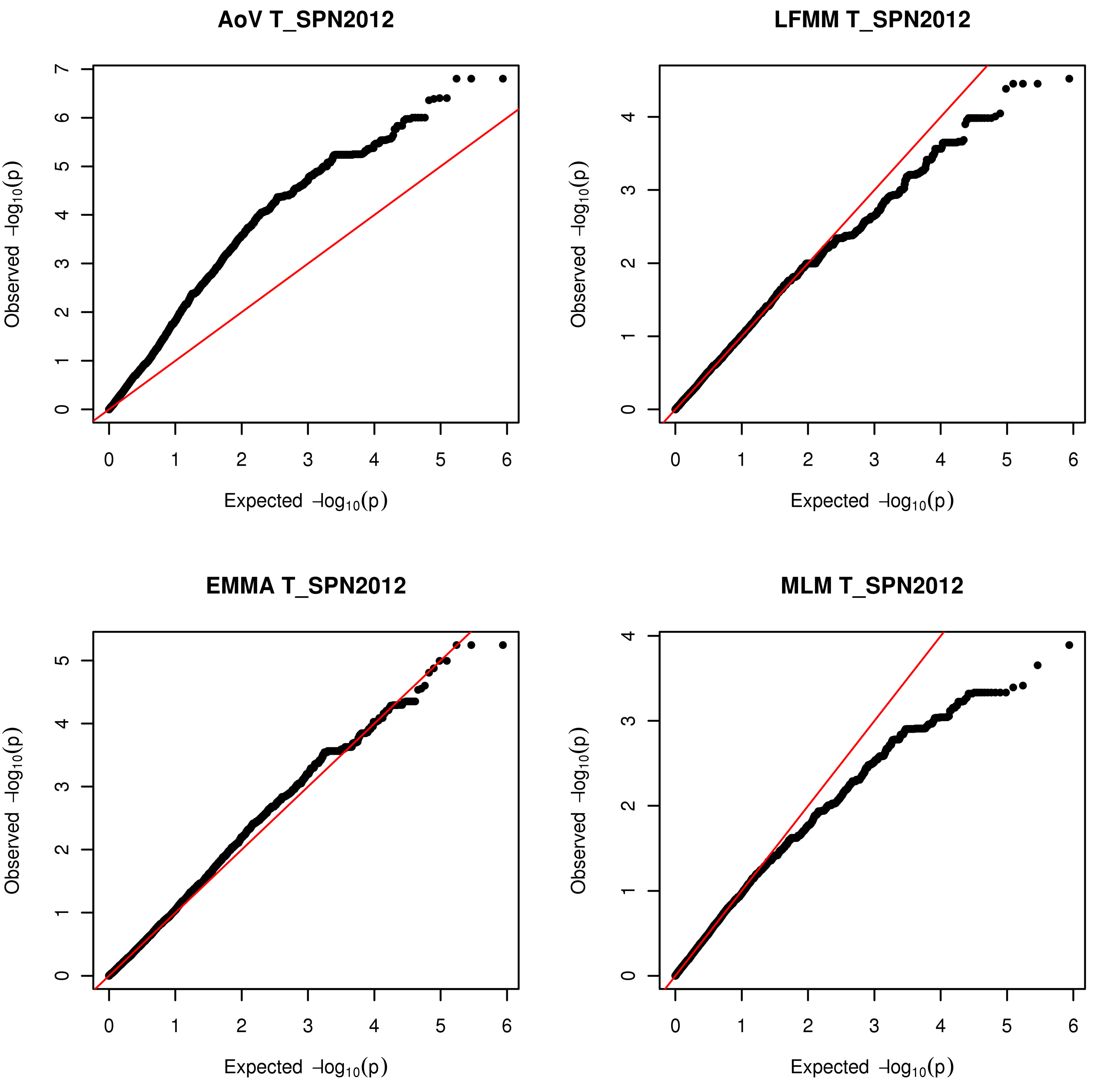

AoV T_SPN2012
LFMM T_SPN2012
EMMA T_SPN2012
MLM T_SPN2012
Observed −log10(p)
Expected −log10(p)
0
1
2
3
4
5
6
7

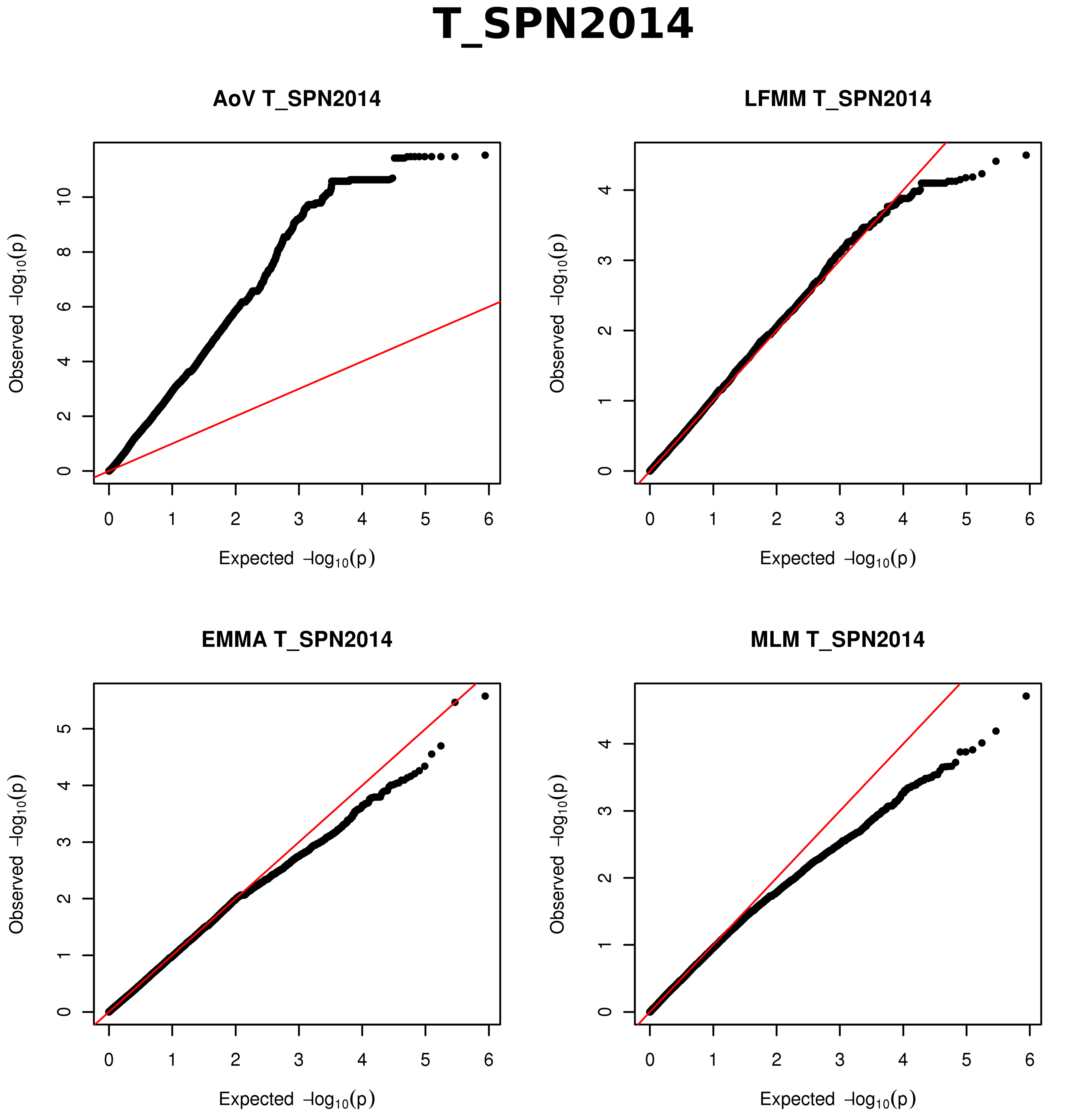

T_SPN2014
AoV T_SPN2014
LFMM T_SPN2014
EMMA T_SPN2014
MLM T_SPN2014
Observed −log10(p)
Expected −log10(p)

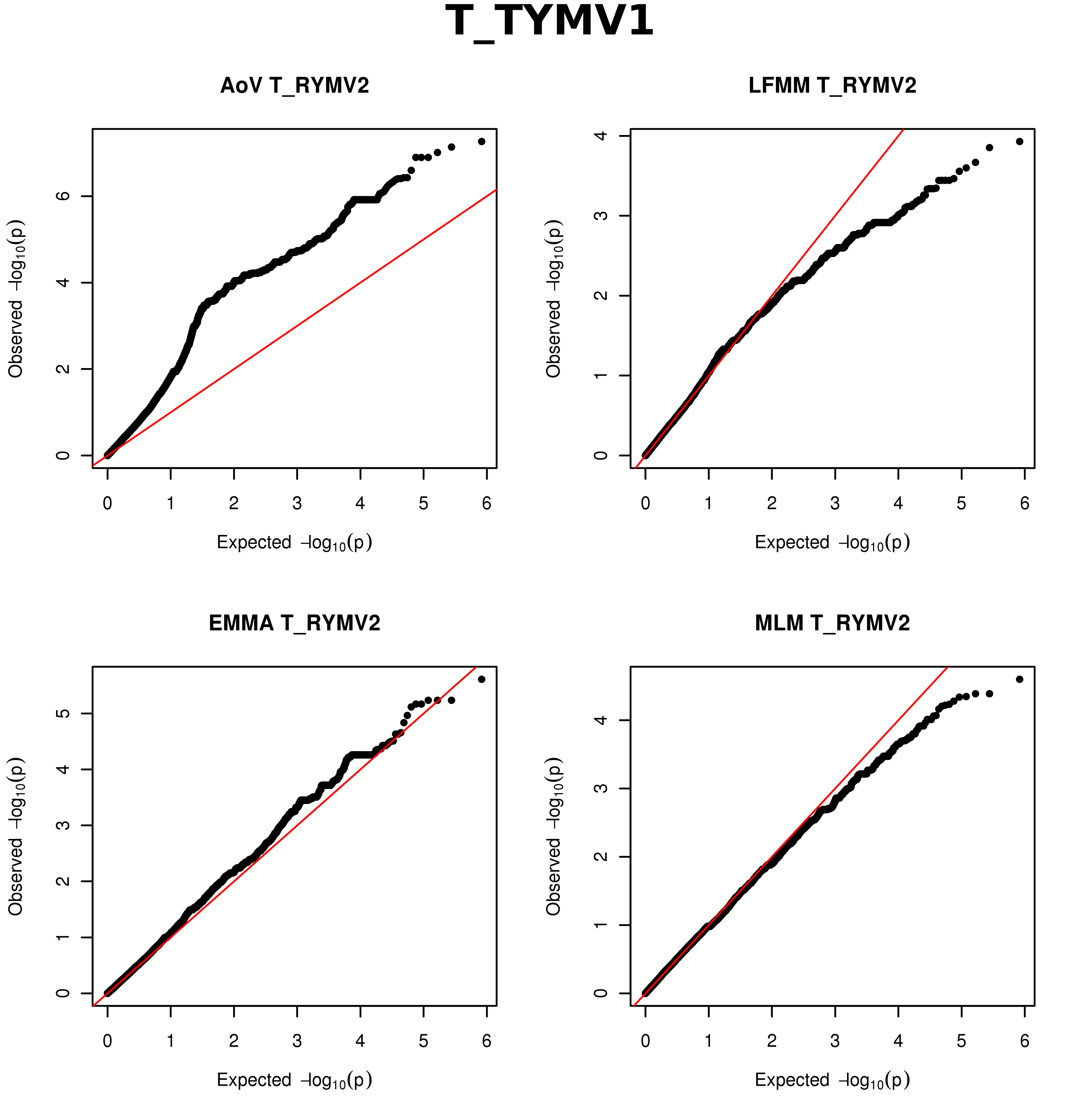
T_TYMV1
AoV T_RYMV2
LFMM T_RYMV2
EMMA T_RYMV2
MLM T_RYMV2
Observed -log10(p)
Expected -log10(p)
0
1
2
3
4
5
6

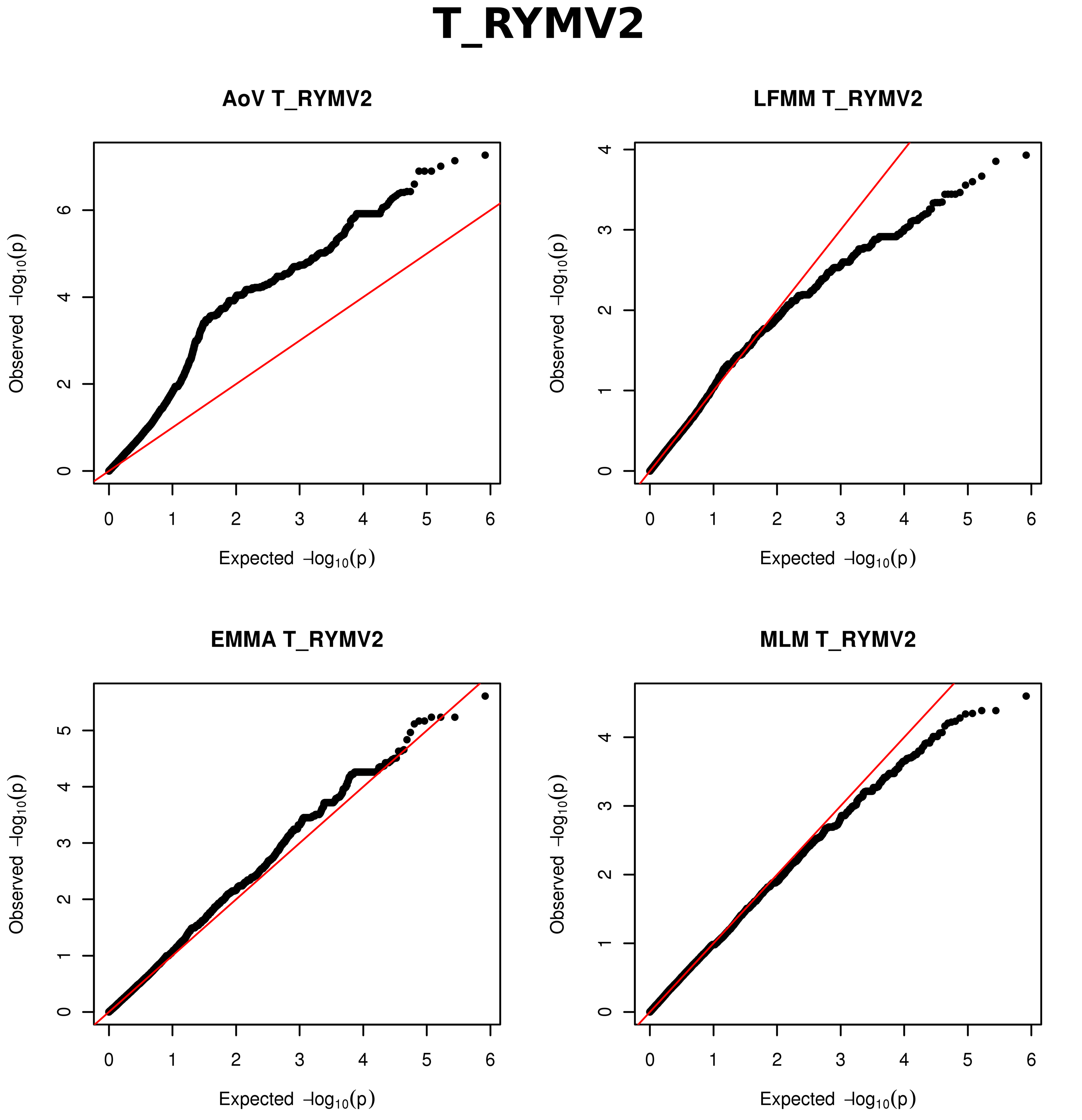
T_RYMV2
AoV T_RYMV2
LFMM T_RYMV2
EMMA T_RYMV2
MLM T_RYMV2
Observed −log10(p)
Expected −log10(p)
0
1
2
3
4
5
6

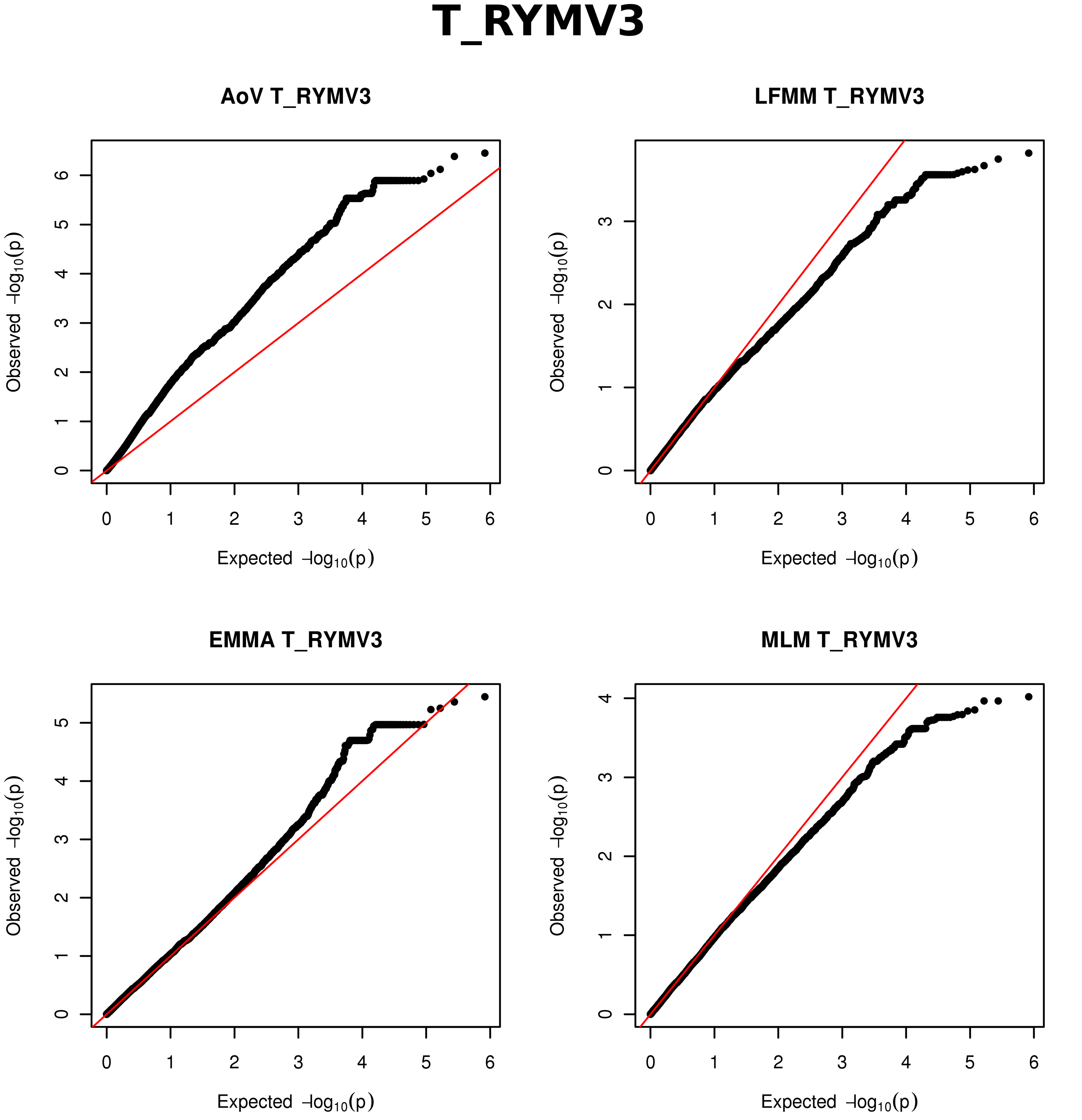
T_RYMV3
AoV T_RYMV3
LFMM T_RYMV3
EMMA T_RYMV3
MLM T_RYMV3
Observed −log10(p)
Expected −log10(p)
0
1
2
3
4
5
6
