# Supplementary material for: Genome Wide Association Study Pinpoints Key Agronomic QTLs in African Rice Oryza glaberrima
Source: Rice (N Y). 2020 Sep 16;13:66. doi: 10.1186/s12284-020-00424-1 (PMC7494698; doi:10.1186/s12284-020-00424-1)
Supplement: Supplementary file 9 — Additional file 9: Figure S6. Linear scale QQ-plots corresponding to association analysis performed independently on each trait and repetition. Three different methods (EMMA, LFMM, MLM) taking into account relatedness and/or structure were used for association and ANOVA was used as a benchmark. For a given trait, the transformed data were used if at least one of the replicates failed to reach normality, otherwise non-transformed data were used. [file 12284_2020_424_MOESM9_ESM.pdf]

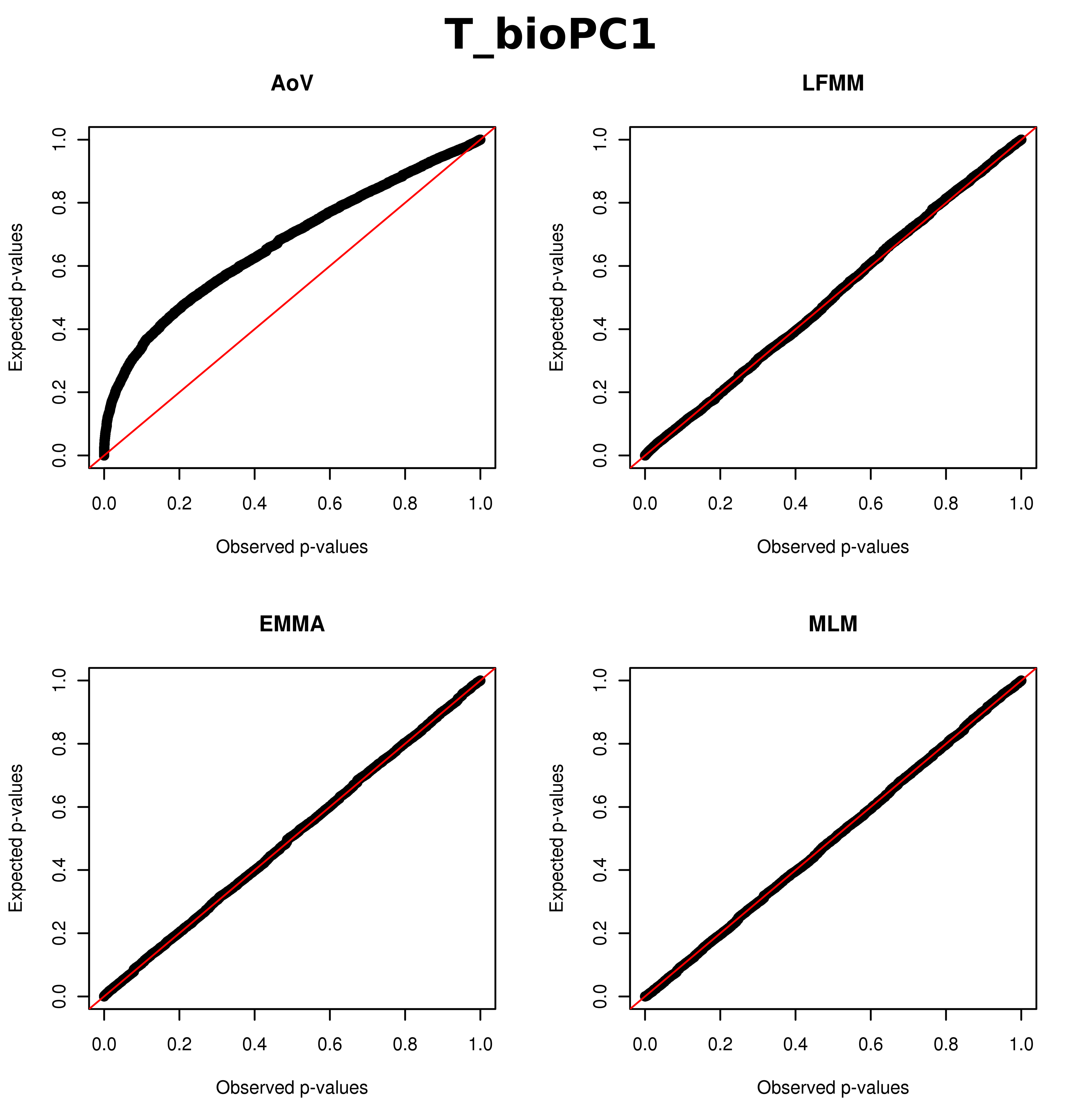
T_bioPC1
AoV
LFMM
EMMA
MLM
Expected p-values
Observed p-values
0.0
0.2
0.4
0.6
0.8
1.0

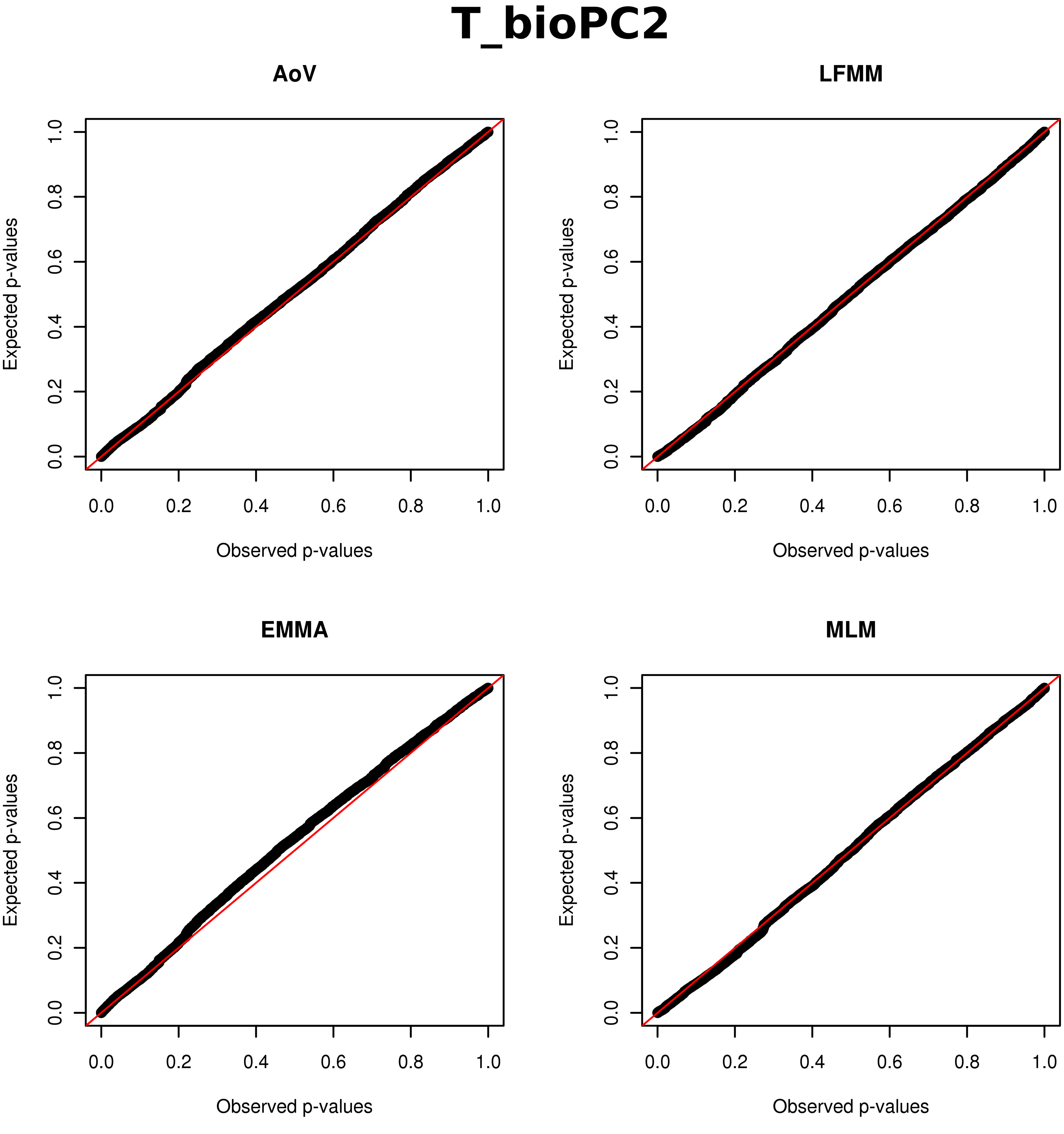
T_bioPC2
AoV
LFMM
EMMA
MLM
Expected p-values
Observed p-values
0.0
0.2
0.4
0.6
0.8
1.0

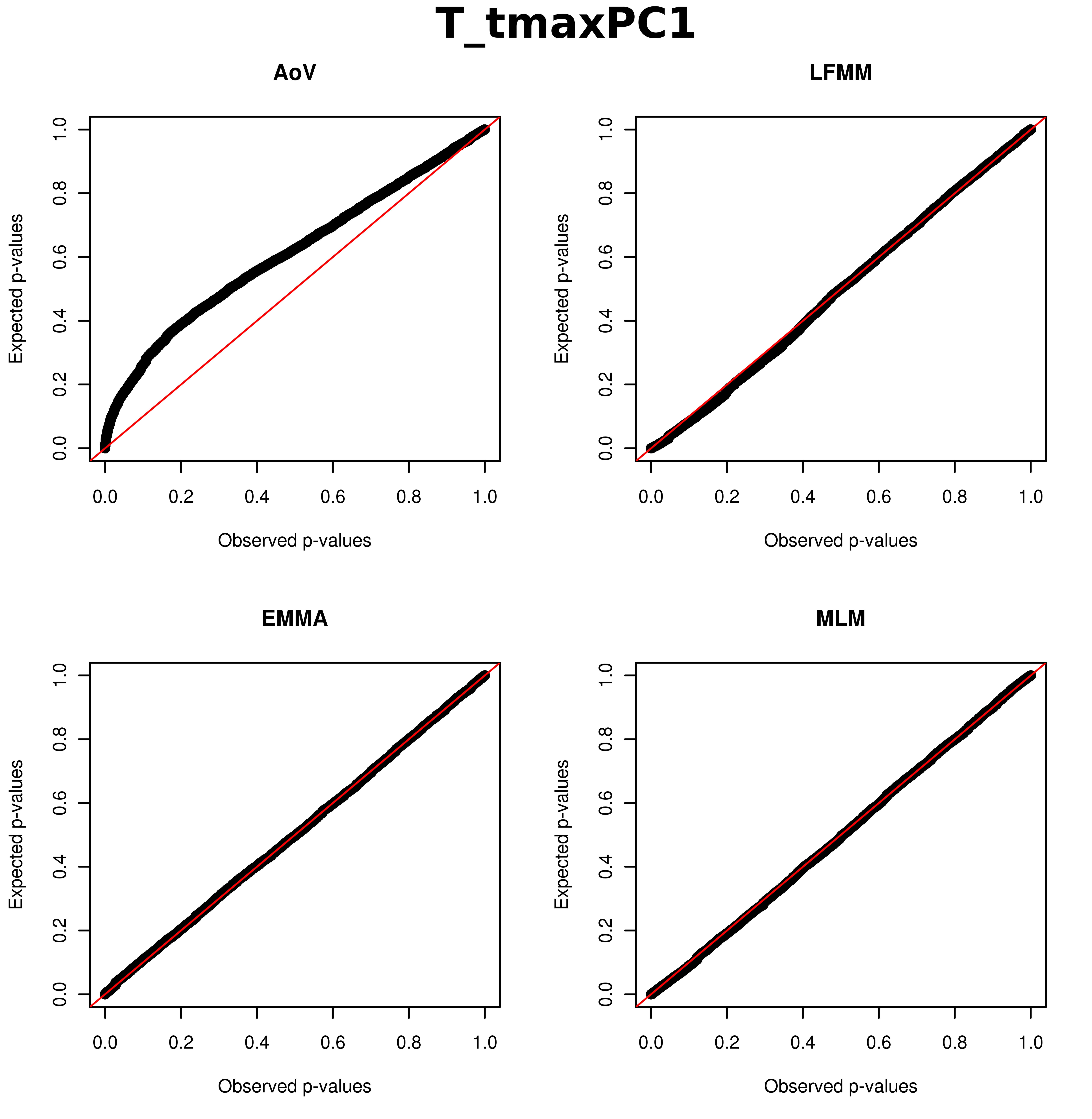
T_tmaxPC1
AoV
LFMM
EMMA
MLM
Expected p-values
Observed p-values
0.0
0.2
0.4
0.6
0.8
1.0

T_tmaxPC2

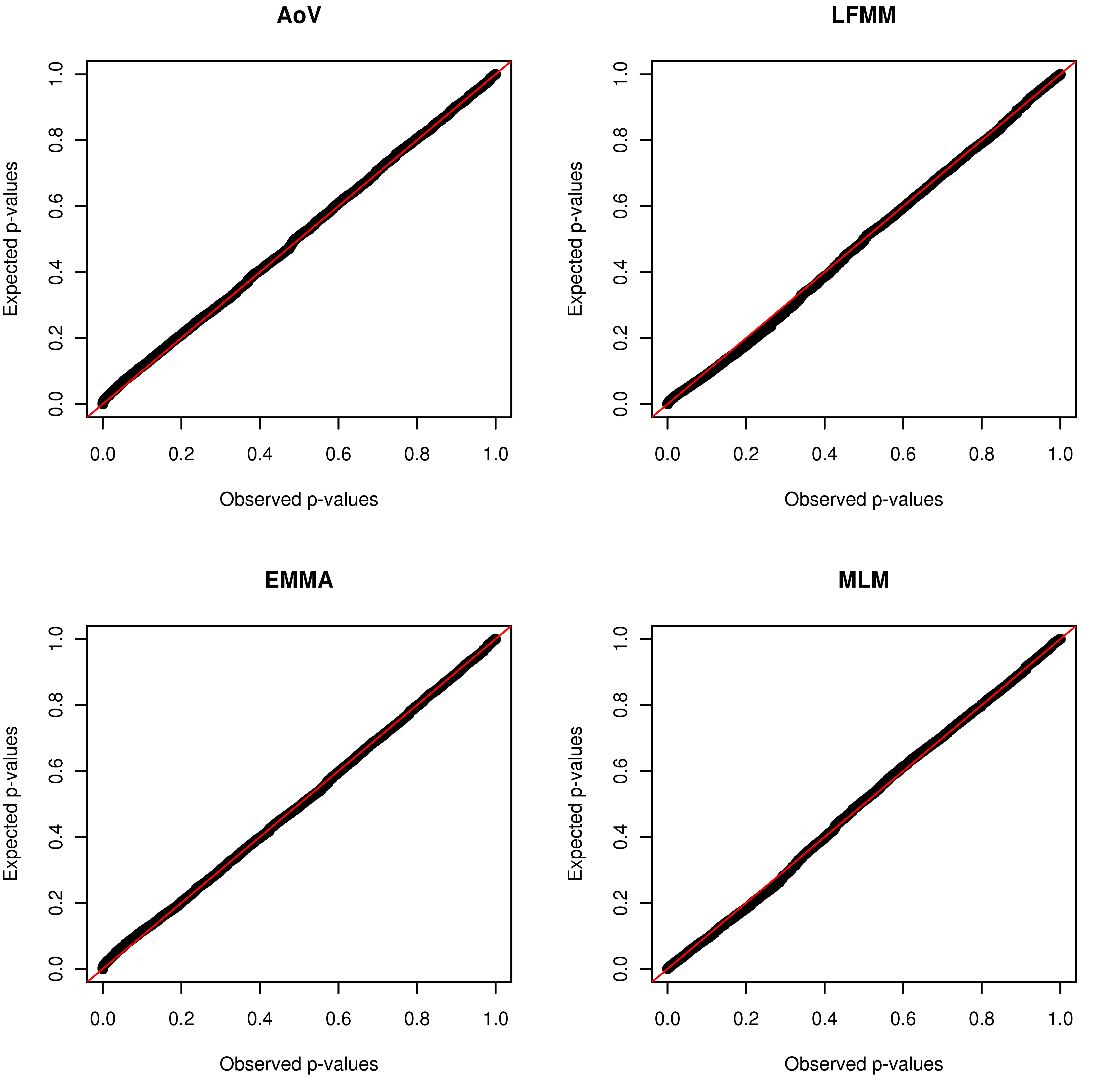

AoV
LFMM
EMMA
MLM
Expected p-values
Observed p-values
0.0
0.2
0.4
0.6
0.8
1.0

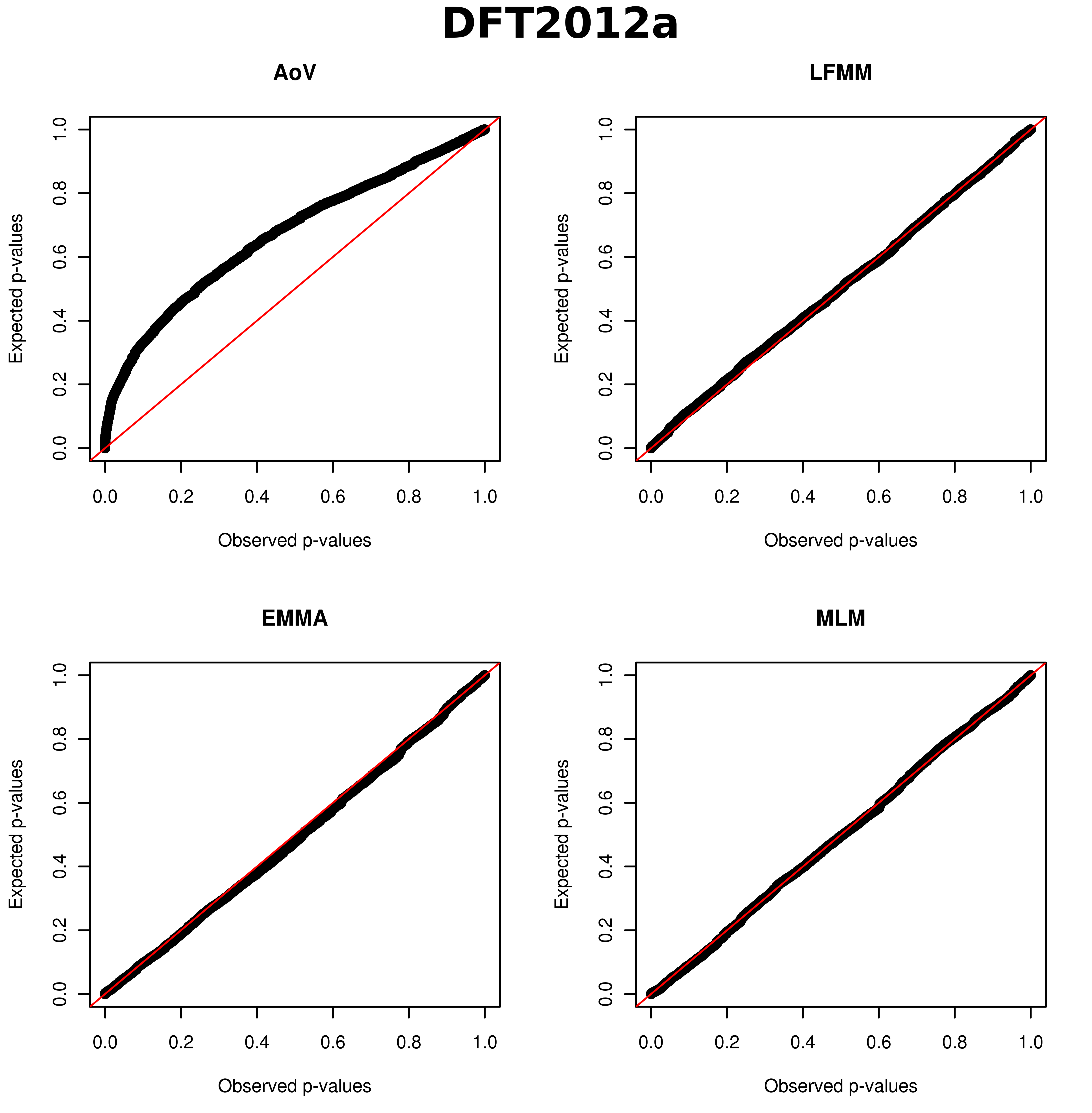
DFT2012a
AoV
LFMM
EMMA
MLM
Expected p-values
Observed p-values
0.0
0.2
0.4
0.6
0.8
1.0

DFT2014a

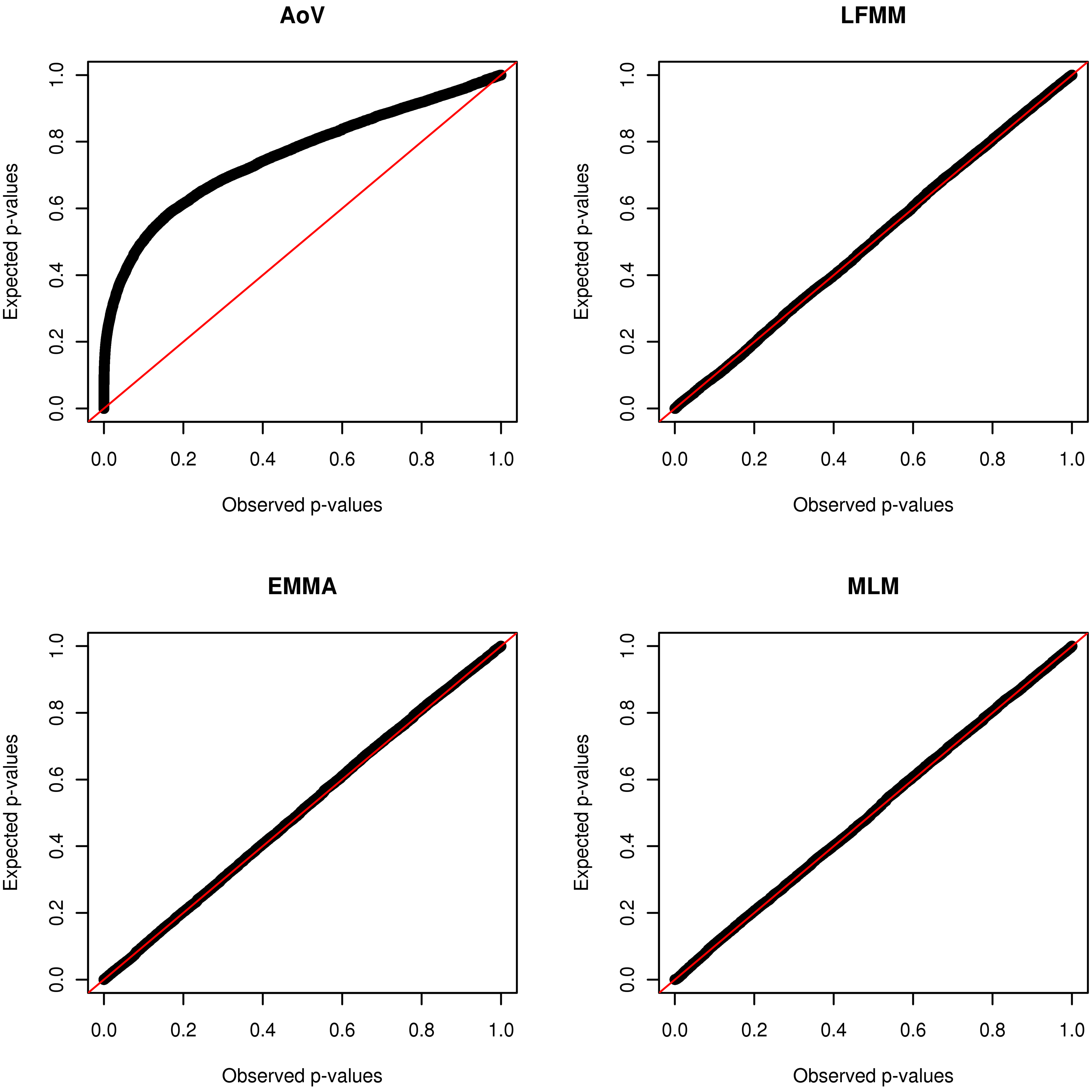
AoV
LFMM
EMMA
MLM
Expected p-values
Observed p-values
0.0
0.2
0.4
0.6
0.8
1.0

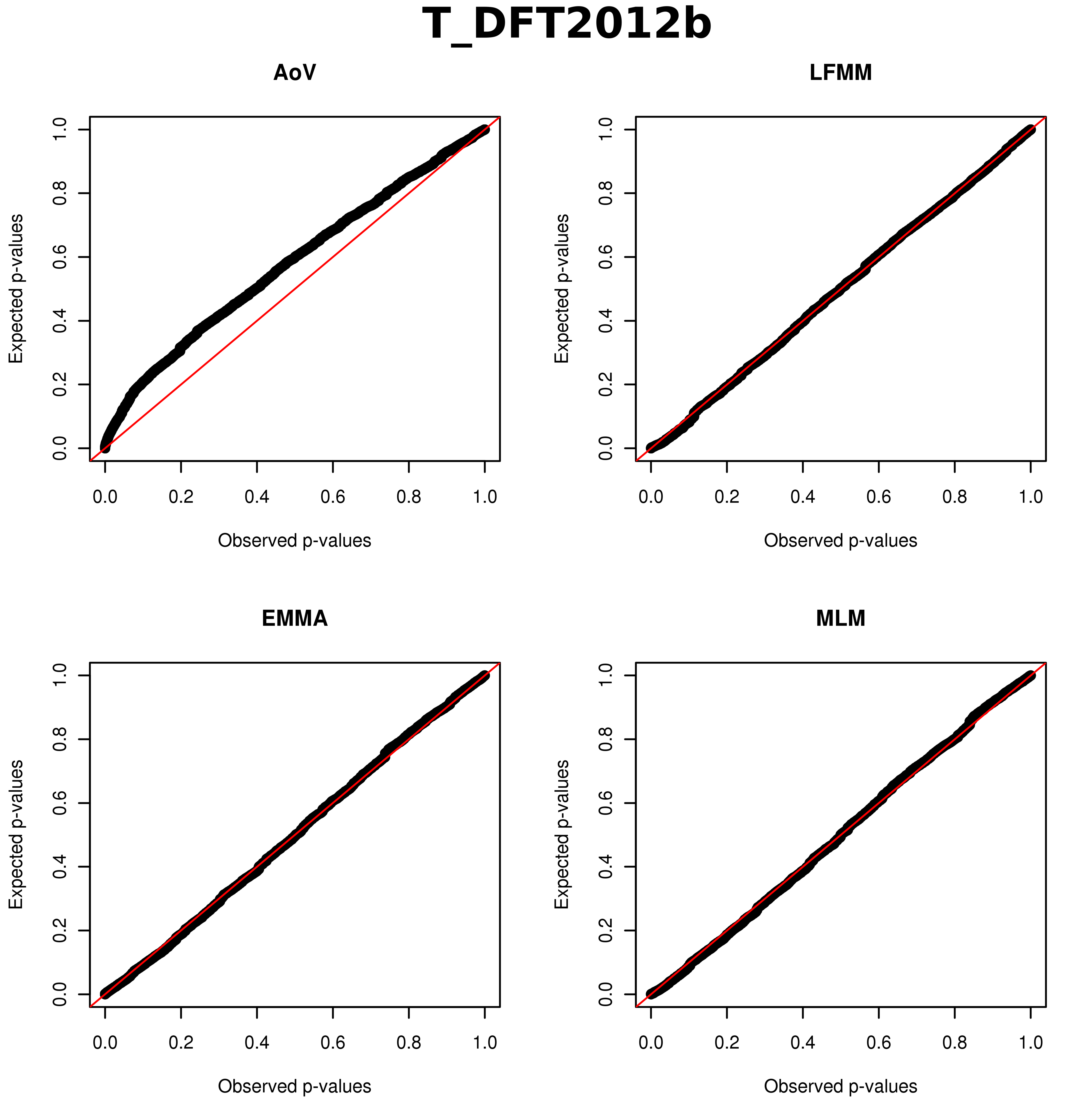
T_DFT2012b
AoV
LFMM
EMMA
MLM
Expected p-values
Observed p-values
0.0
0.2
0.4
0.6
0.8
1.0

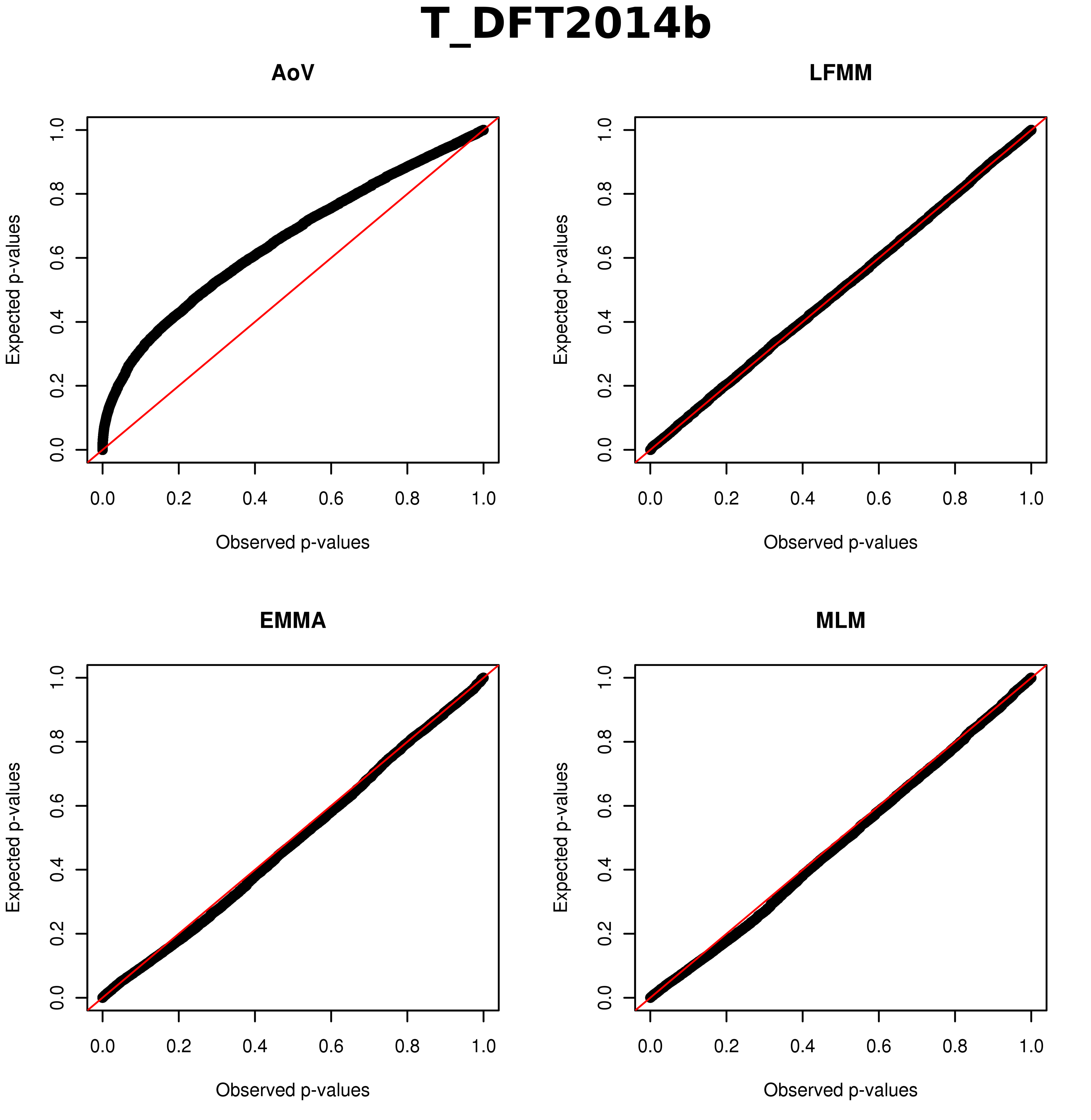
T_DFT2014b
AoV
LFMM
EMMA
MLM
Expected p-values
Observed p-values
0.0
0.2
0.4
0.6
0.8
1.0

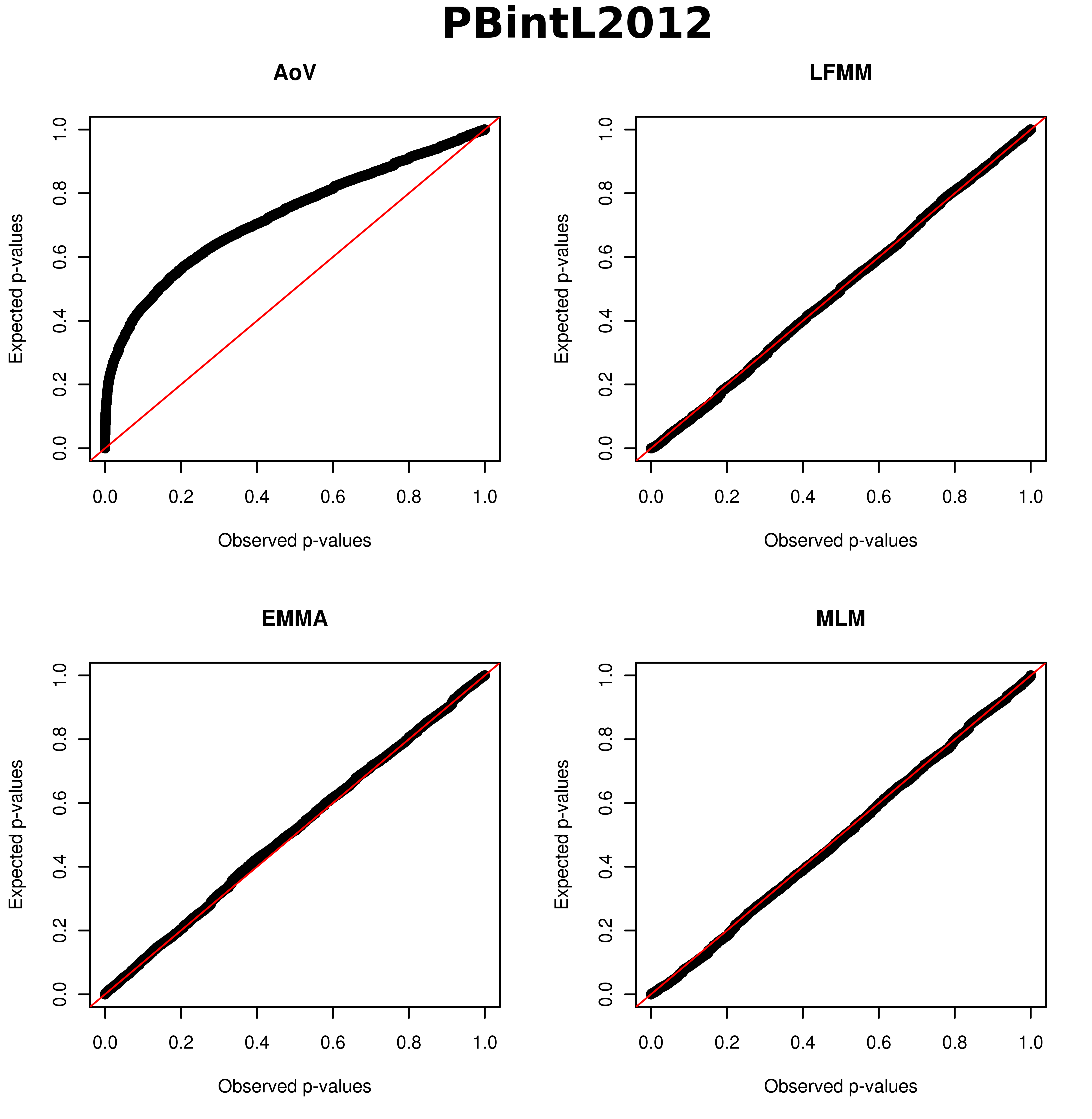
PBintL2012
AoV
LFMM
EMMA
MLM
Expected p-values
Observed p-values
0.0
0.2
0.4
0.6
0.8
1.0

PBintL2014

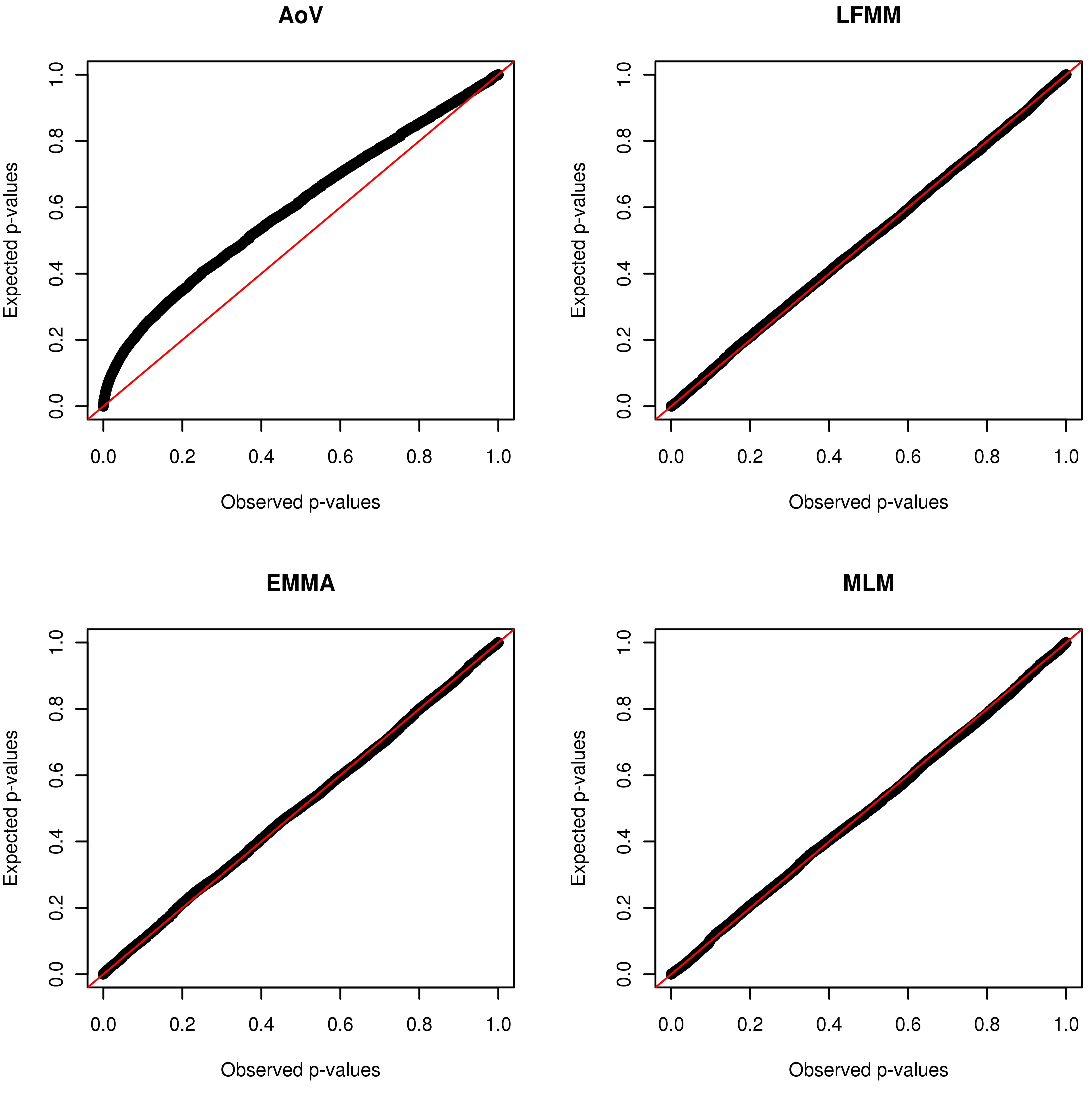
AoV
LFMM
EMMA
MLM
Expected p-values
Observed p-values
0.0
0.2
0.4
0.6
0.8
1.0

T_PBL2012

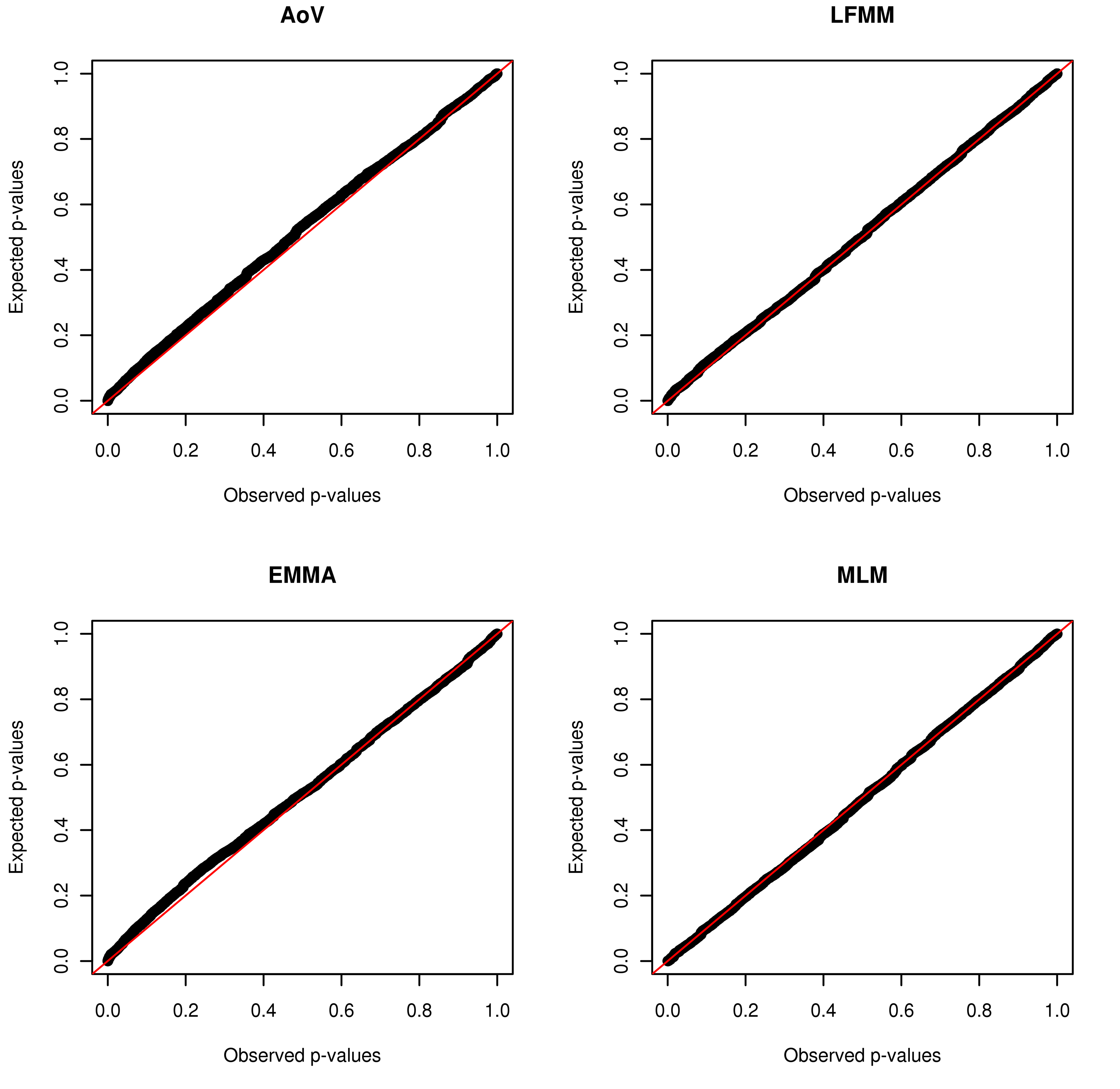
AoV
LFMM
EMMA
MLM
Expected p-values
Observed p-values
0.0
0.2
0.4
0.6
0.8
1.0

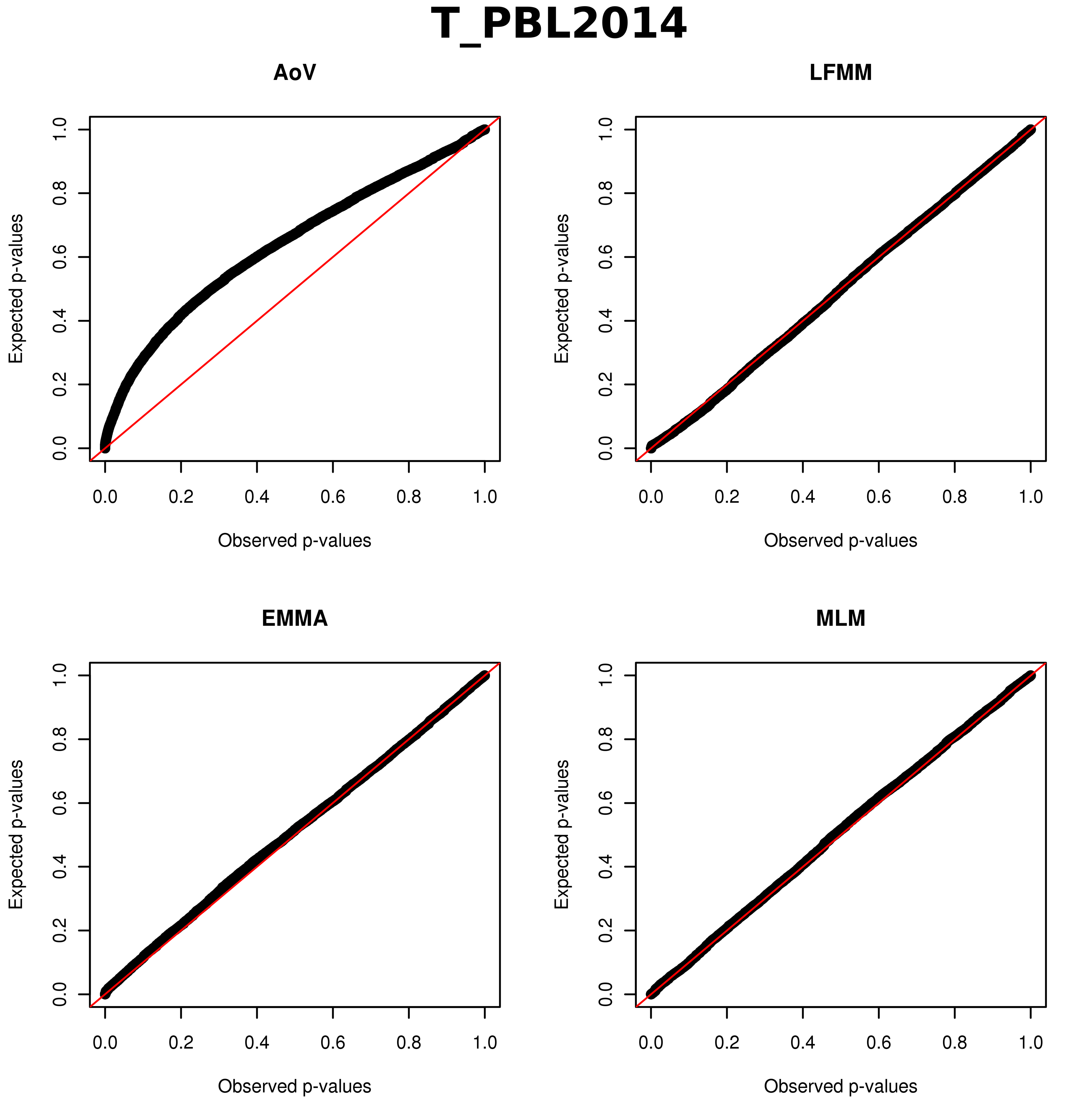
T_PBL2014
AoV
LFMM
EMMA
MLM
Expected p-values
Observed p-values
0.0
0.2
0.4
0.6
0.8
1.0

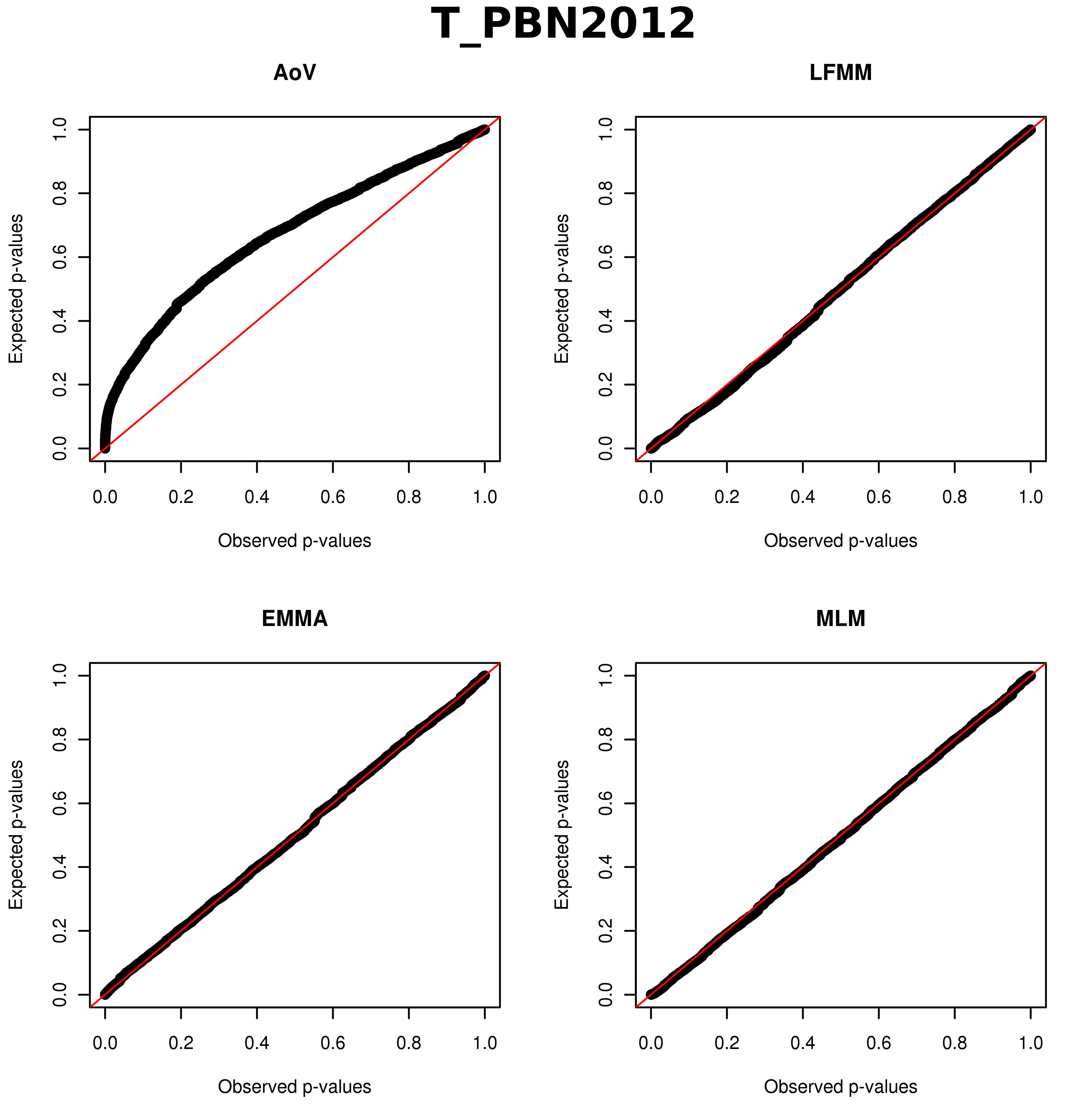

T_PBN2012
AoV
LFMM
EMMA
MLM
Expected p-values
Observed p-values
0.0
0.2
0.4
0.6
0.8
1.0

T_PBN2014

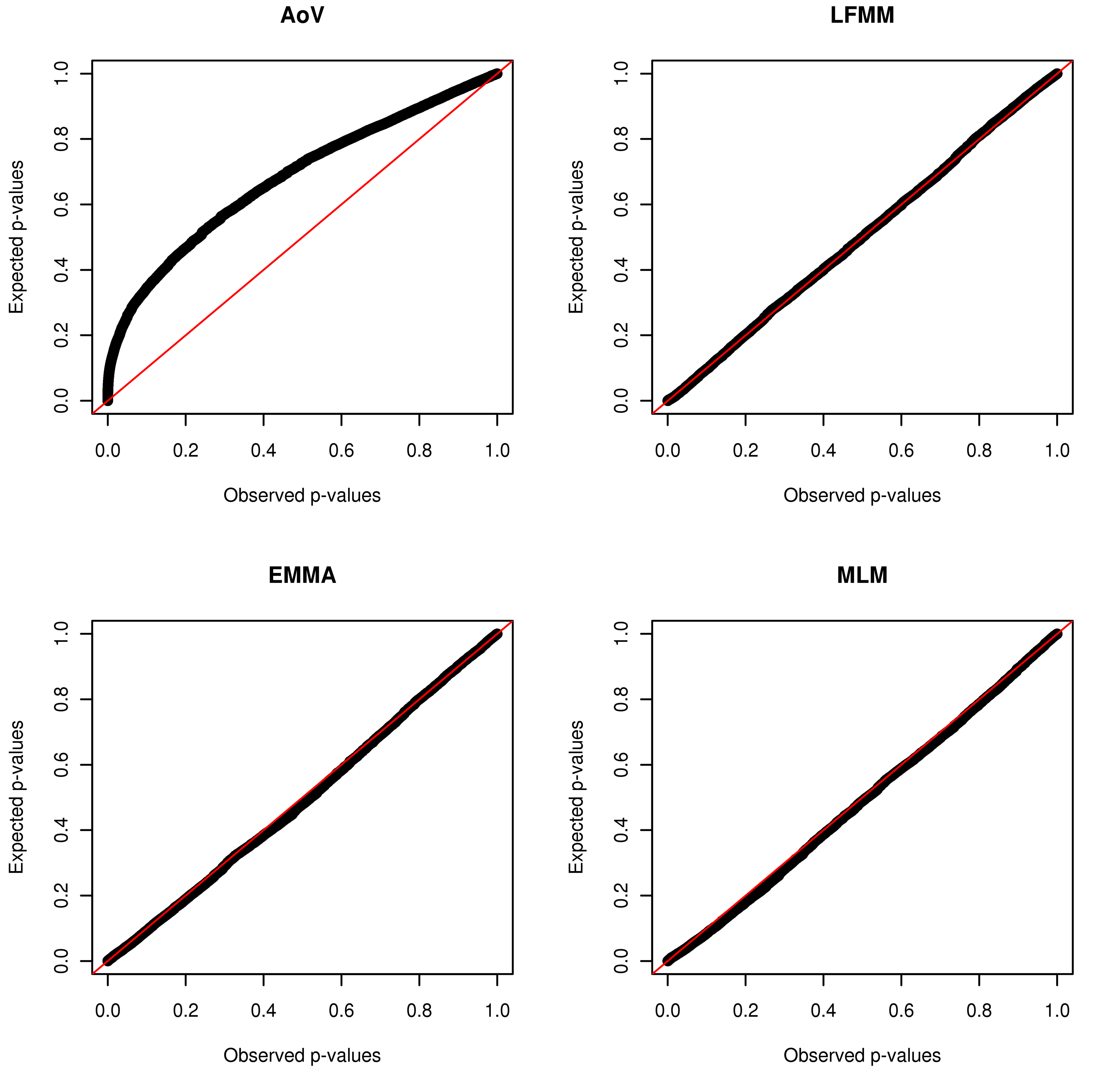
AoV
LFMM
EMMA
MLM
Expected p-values
Observed p-values
0.0
0.2
0.4
0.6
0.8
1.0

T_RL2012

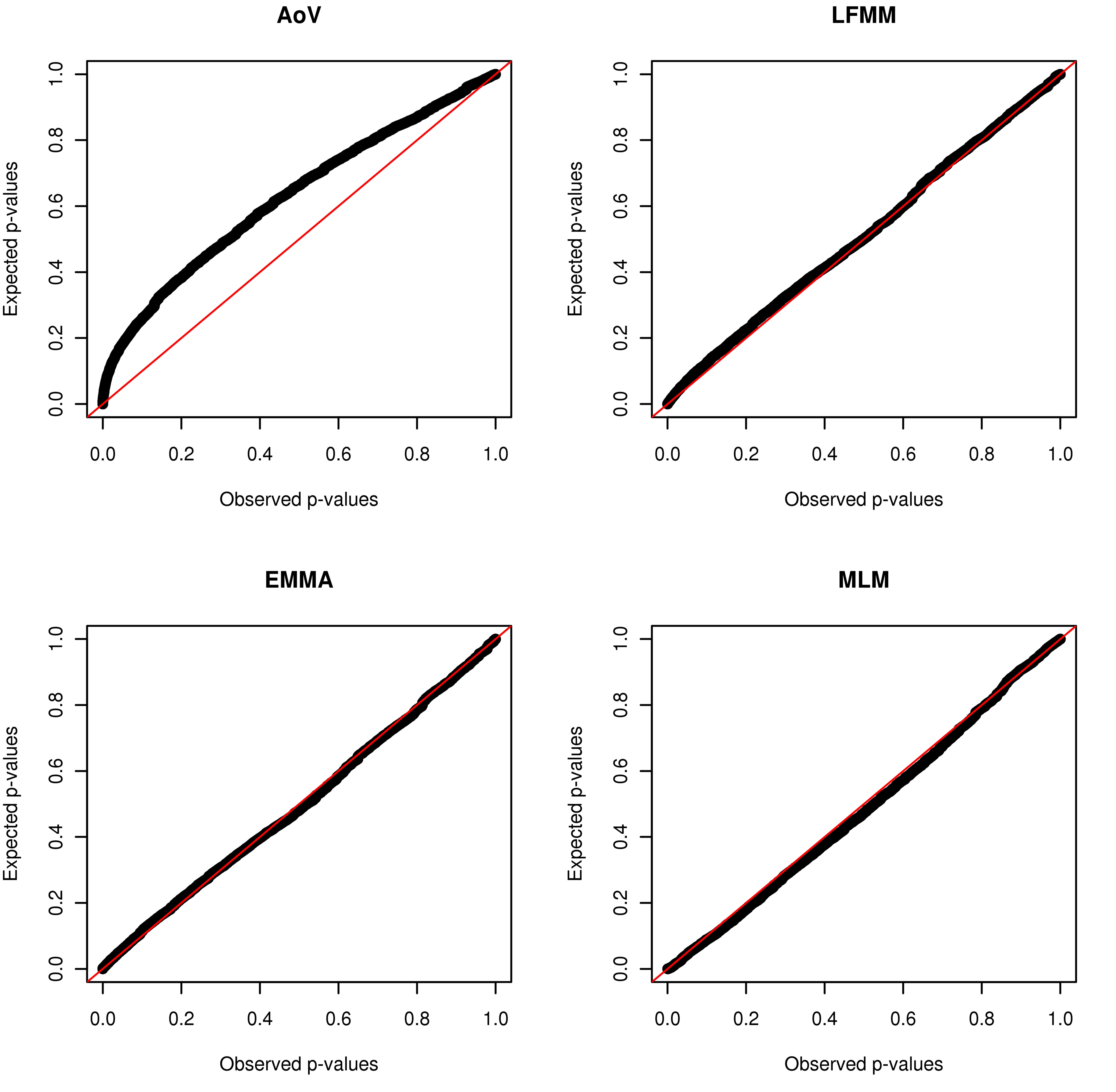

AoV
LFMM
EMMA
MLM
Expected p-values
Observed p-values
0.0
0.2
0.4
0.6
0.8
1.0

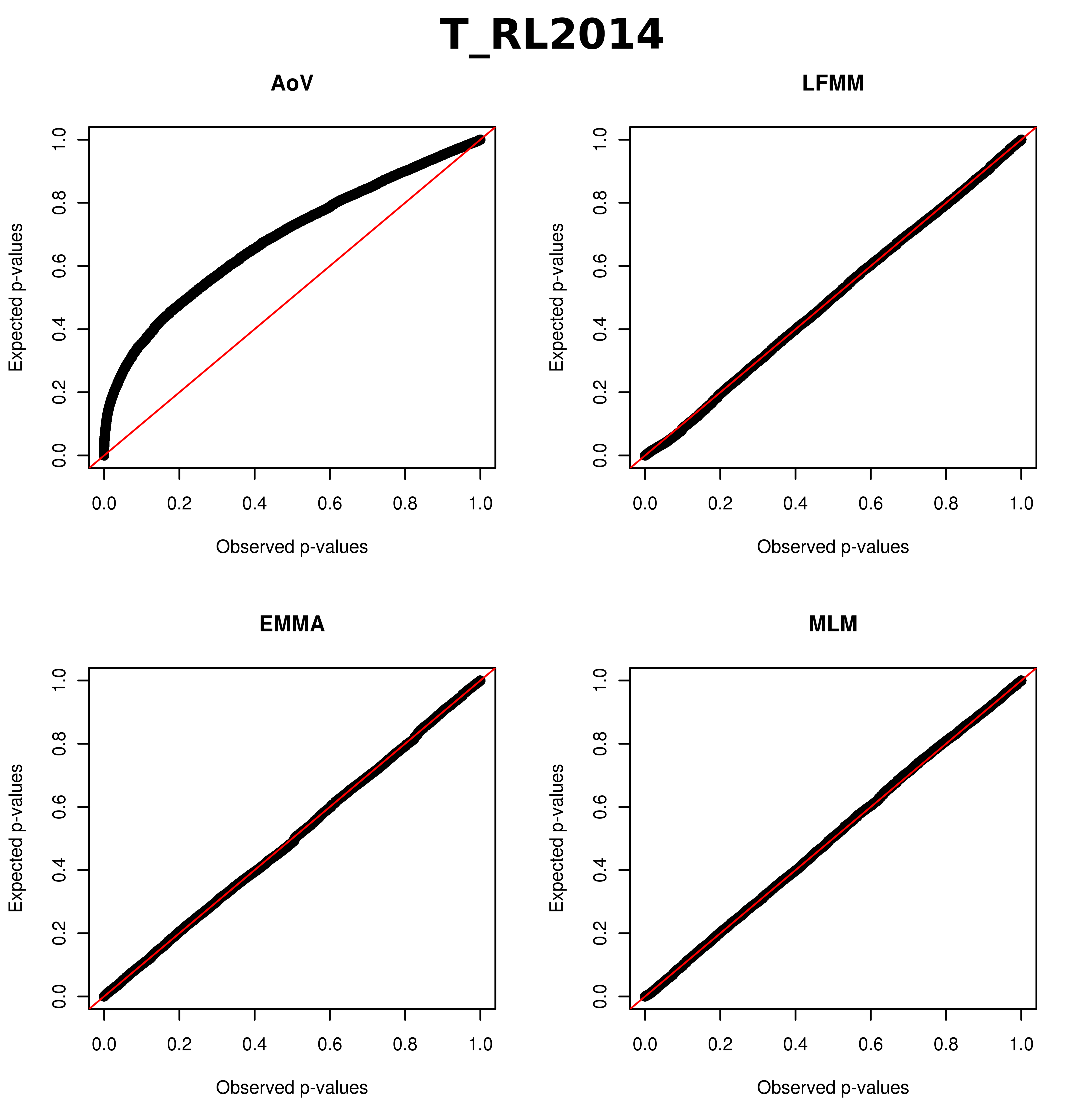
T_RL2014
AoV
LFMM
EMMA
MLM
Expected p-values
Observed p-values
0.0
0.2
0.4
0.6
0.8
1.0

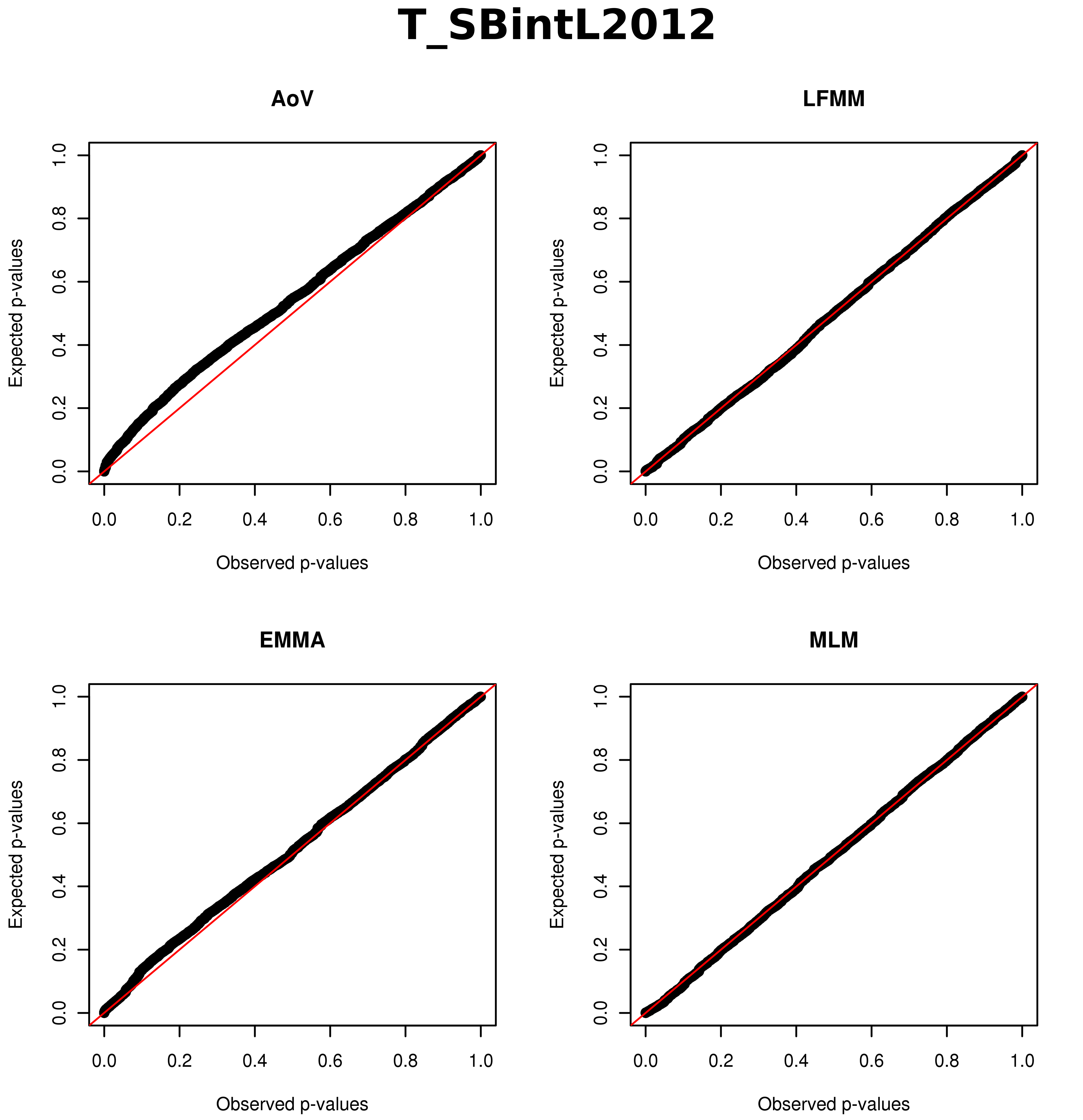
T_SBintL2012
AoV
LFMM
EMMA
MLM
Expected p-values
Observed p-values
0.0
0.2
0.4
0.6
0.8
1.0

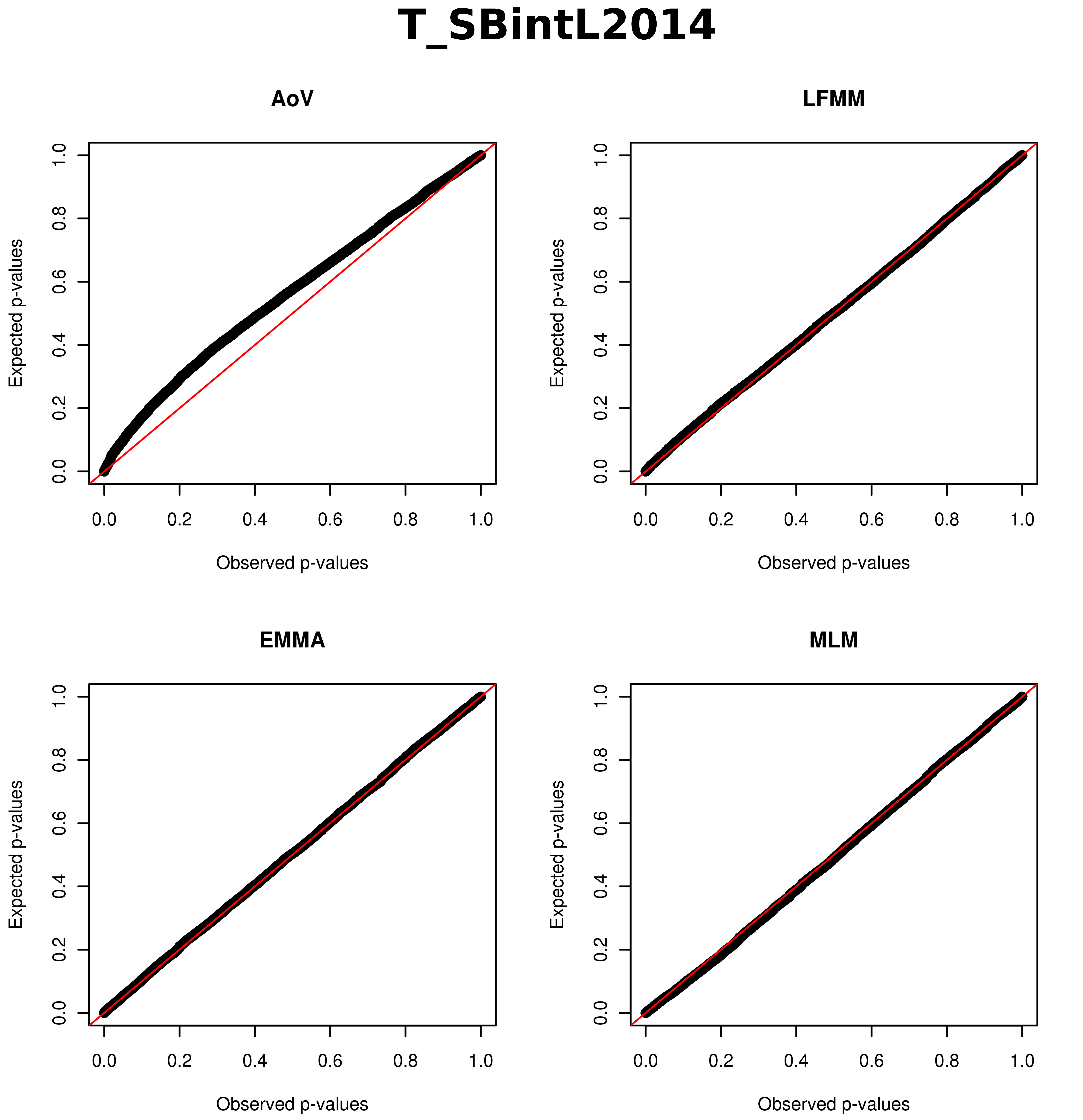
T_SBintL2014
AoV
LFMM
EMMA
MLM
Expected p-values
Observed p-values
0.0
0.2
0.4
0.6
0.8
1.0

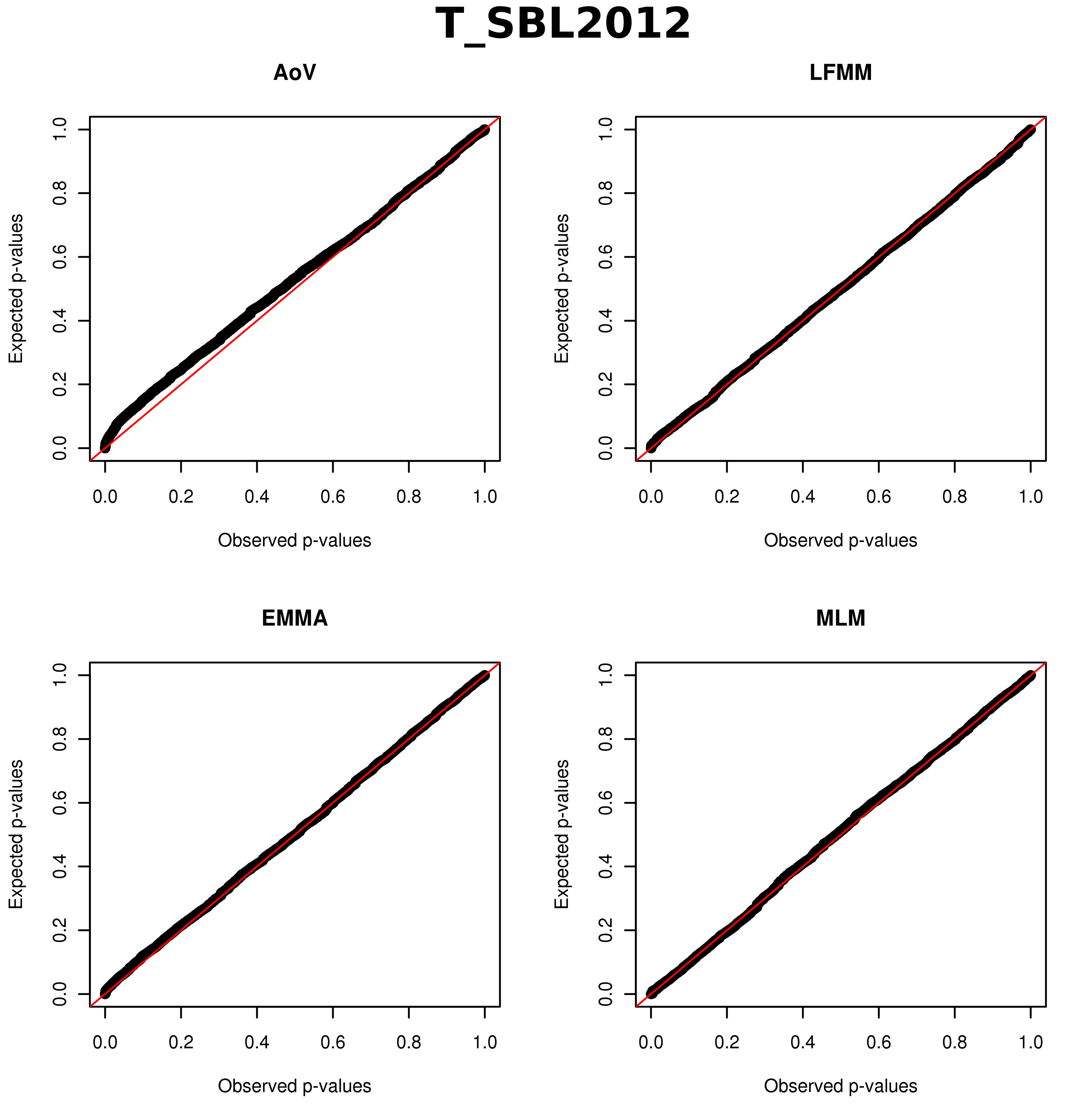
T_SBL2012
AoV
LFMM
EMMA
MLM
Expected p-values
Observed p-values
0.0
0.2
0.4
0.6
0.8
1.0

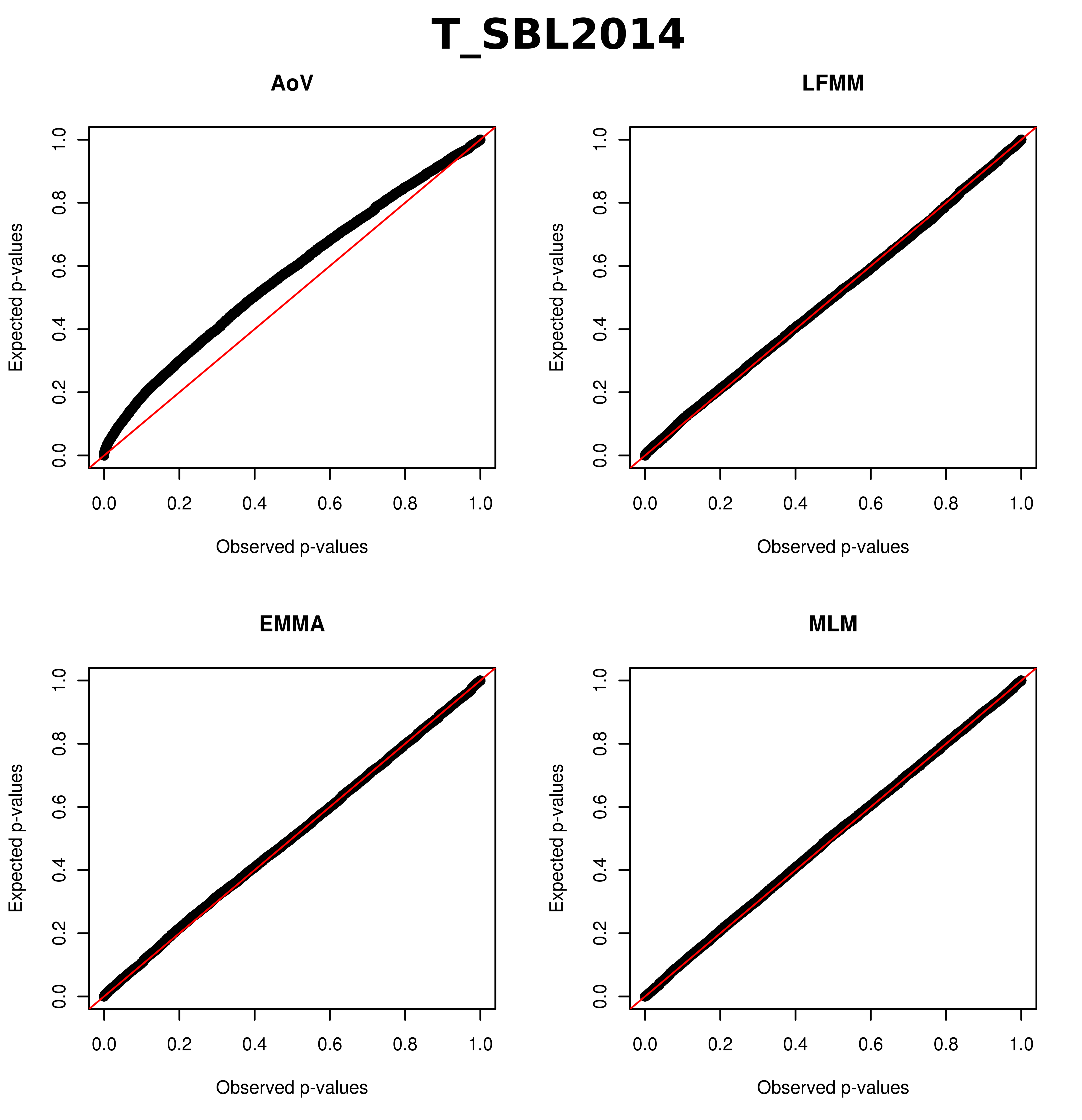
T_SBL2014
AoV
LFMM
EMMA
MLM
Expected p-values
Observed p-values
0.0
0.2
0.4
0.6
0.8
1.0

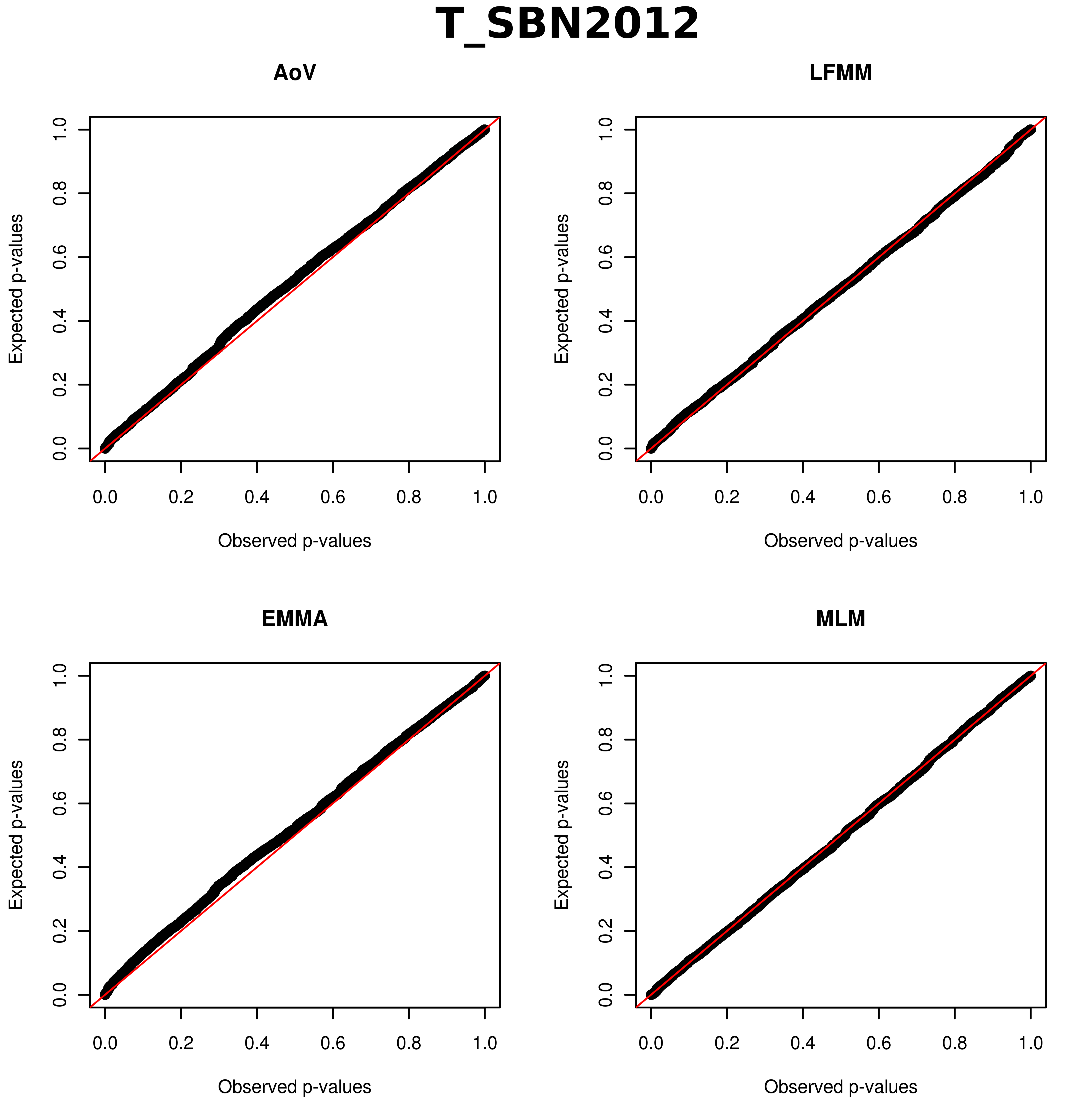
T_SBN2012
AoV
LFMM
EMMA
MLM
Expected p-values
Observed p-values
0.0
0.2
0.4
0.6
0.8
1.0

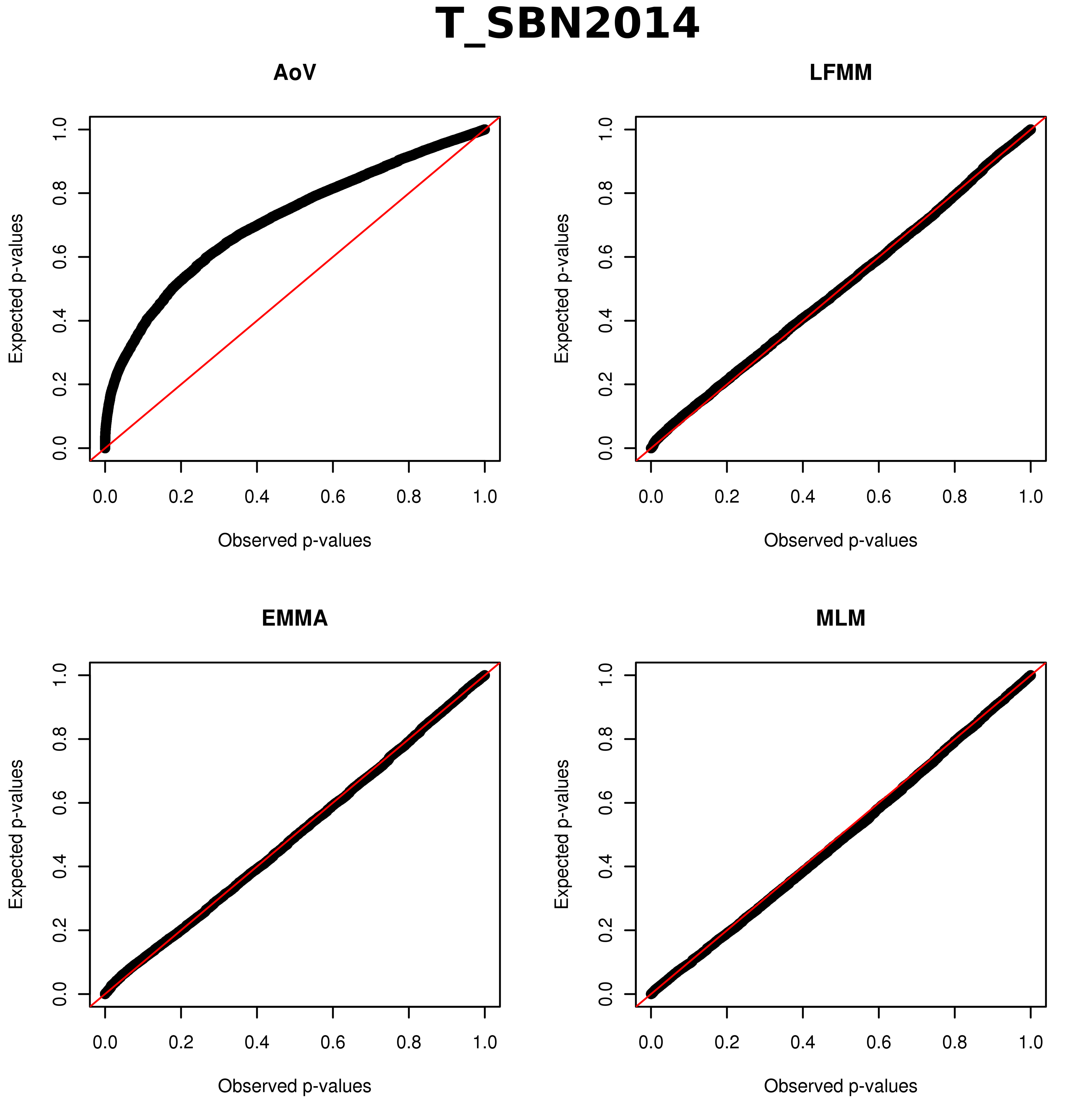

T_SBN2014
AoV
LFMM
EMMA
MLM
Expected p-values
Observed p-values
0.0
0.2
0.4
0.6
0.8
1.0

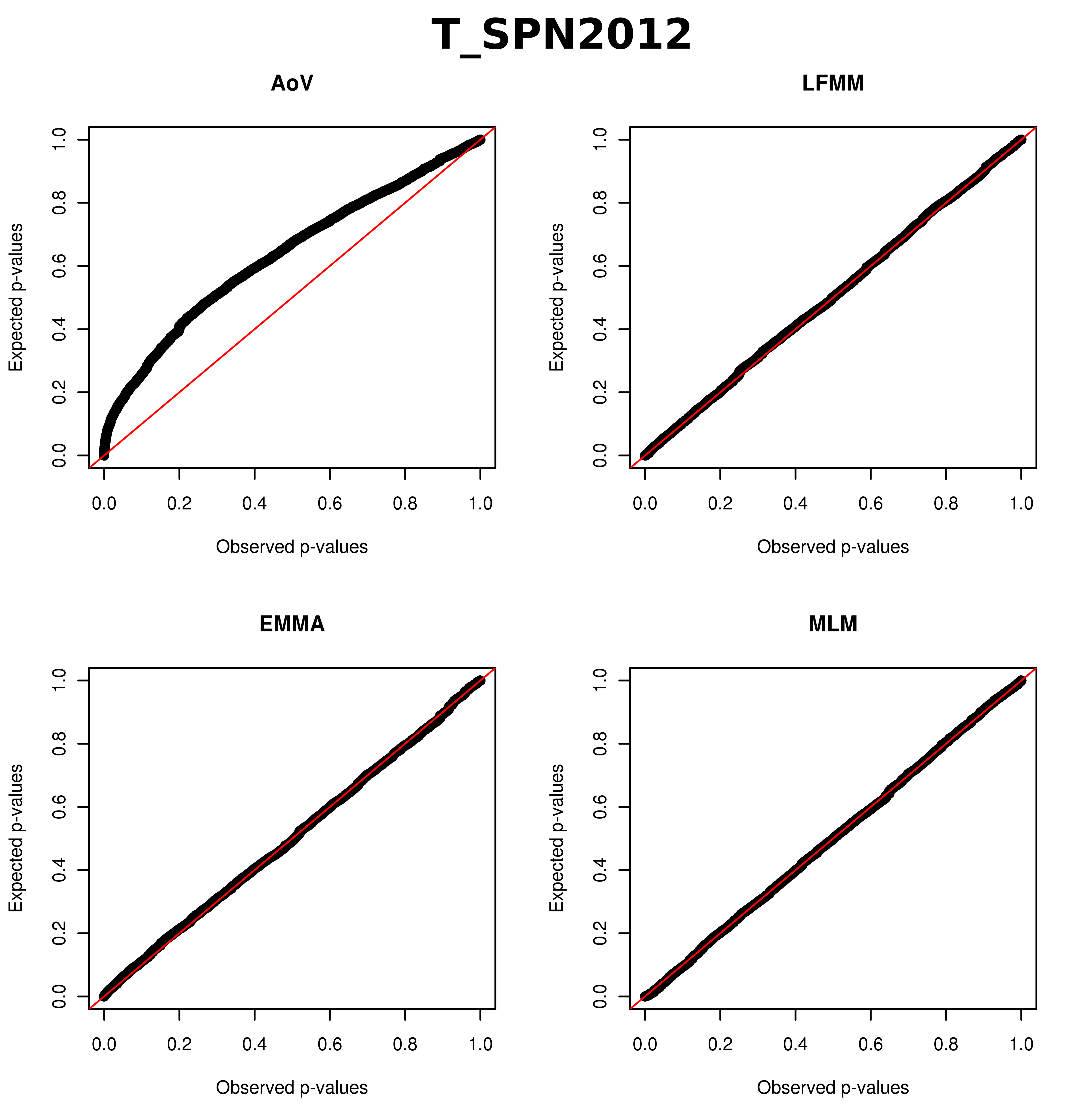
T_SPN2012
AoV
LFMM
EMMA
MLM
Expected p-values
Observed p-values
0.0
0.2
0.4
0.6
0.8
1.0

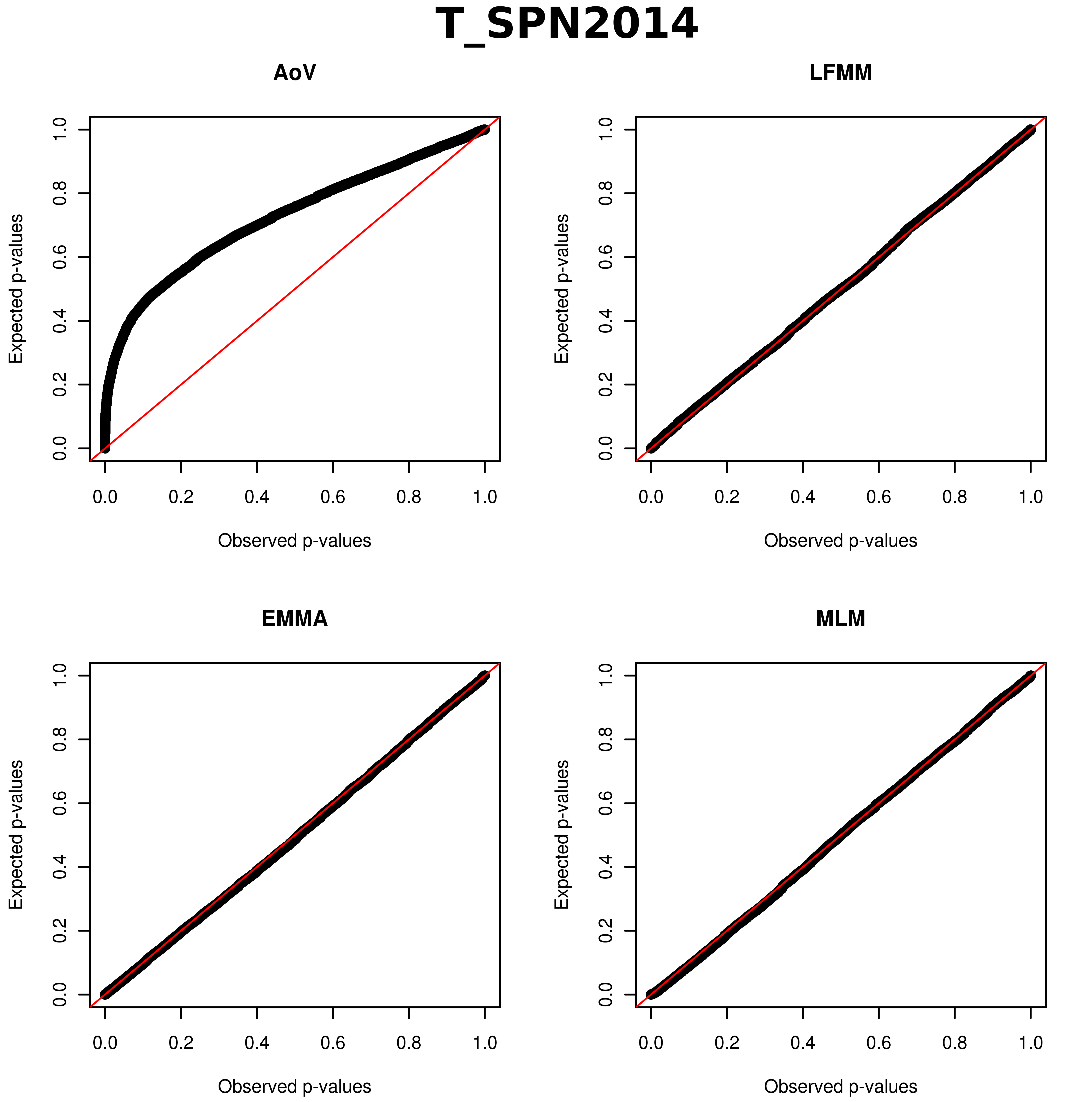
T_SPN2014
AoV
LFMM
EMMA
MLM
Expected p-values
Observed p-values
0.0
0.2
0.4
0.6
0.8
1.0

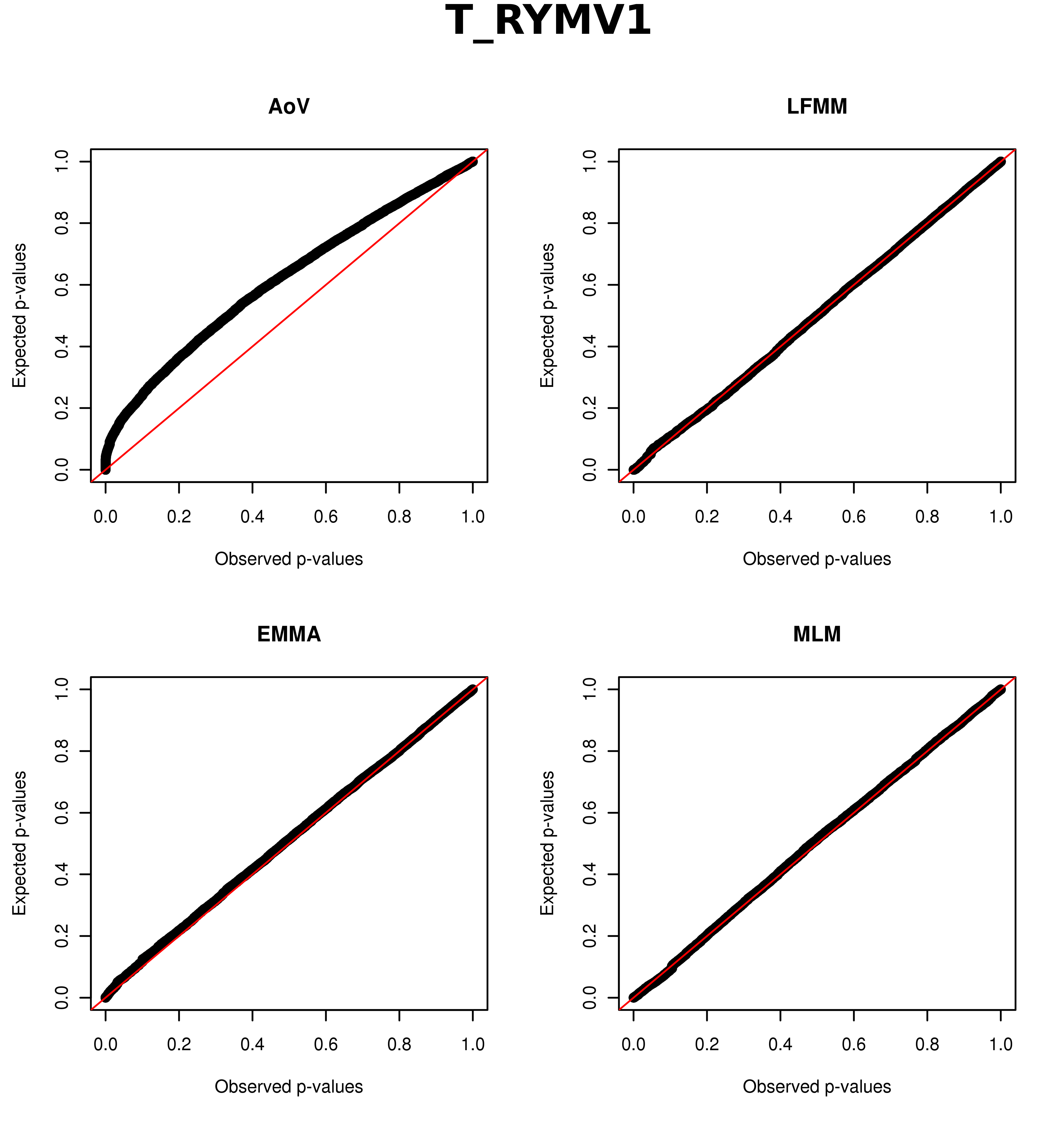
T_RYMV1
AoV
LFMM
EMMA
MLM
Expected p-values
Observed p-values
0.0
0.2
0.4
0.6
0.8
1.0

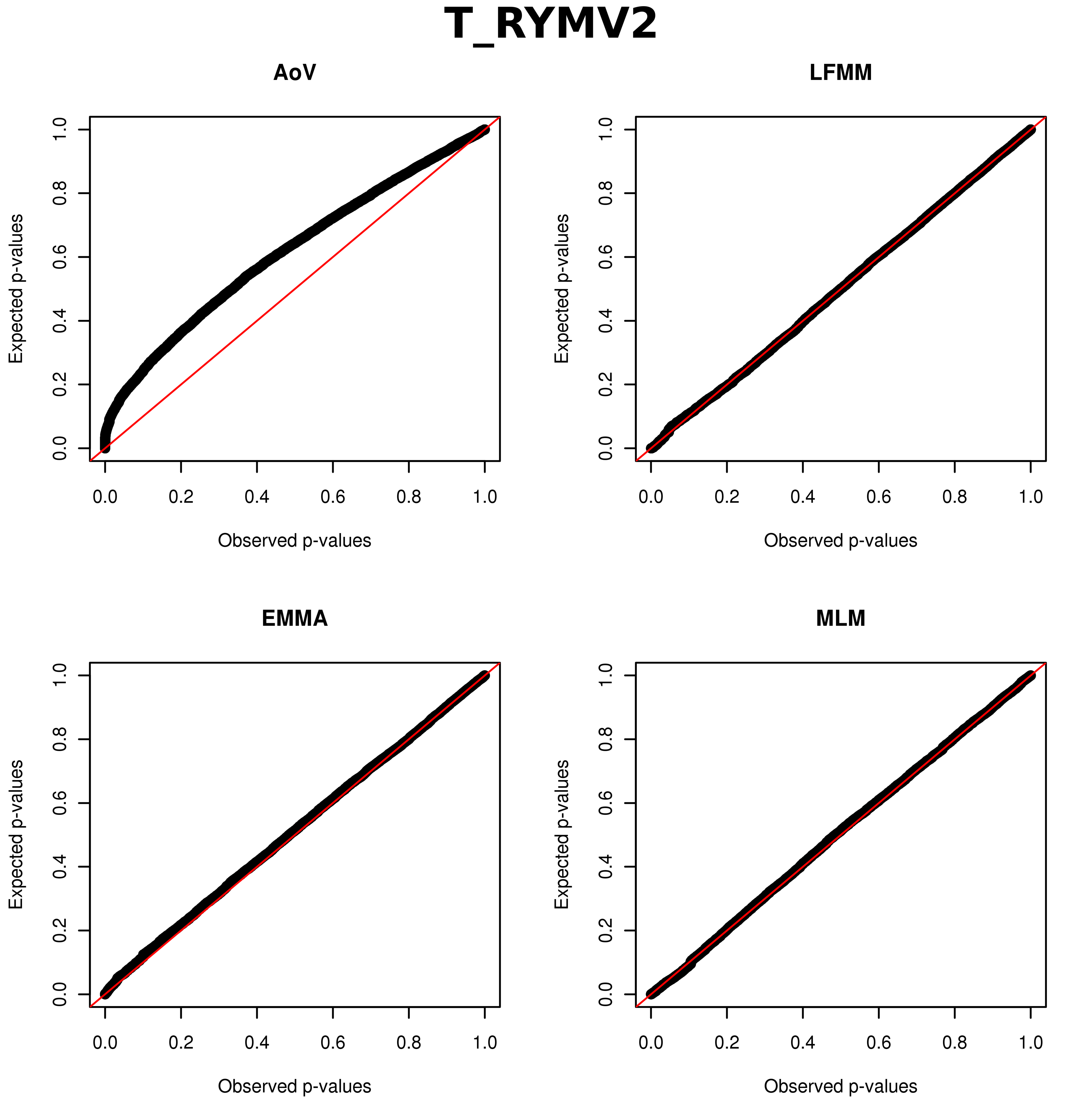
T_RYMV2
AoV
LFMM
EMMA
MLM
Expected p-values
Observed p-values
0.0
0.2
0.4
0.6
0.8
1.0

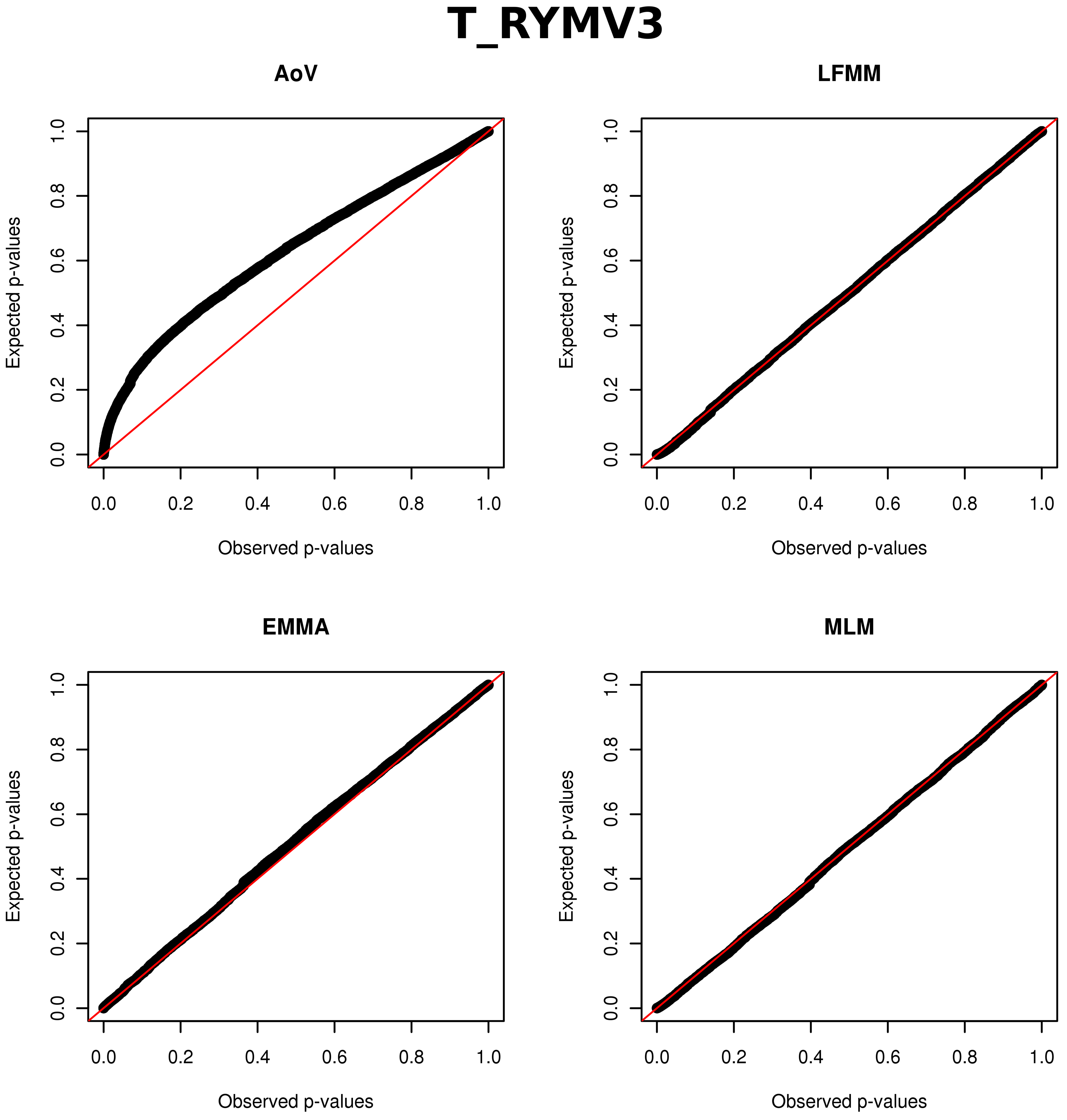
T_RYMV3
AoV
LFMM
EMMA
MLM
Expected p-values
Observed p-values
0.0
0.2
0.4
0.6
0.8
1.0
